# Absence of *Wdr13* Gene Predisposes Mice to Mild Social Isolation – Chronic Stress, Leading to Depression-Like Phenotype Associated With Differential Expression of Synaptic Proteins

**DOI:** 10.3389/fnmol.2018.00133

**Published:** 2018-04-25

**Authors:** Shiladitya Mitra, Ghantasala S. Sameer Kumar, B. Jyothi Lakshmi, Suman Thakur, Satish Kumar

**Affiliations:** ^1^Council of Scientific and Industrial Research-Centre for Cellular and Molecular Biology, Hyderabad, India; ^2^Laboratory of Neurobiology, Nencki Institute of Experimental Biology, Warsaw, Poland; ^3^Biopharma Division, Vimta Labs Ltd., Hyderabad, India

**Keywords:** *Wdr13*, gene environment interaction, depression, synaptic genes, *Gata1*

## Abstract

We earlier reported that the male mice lacking the *Wdr13* gene (*Wdr13*^-/0^) showed mild anxiety, better memory retention, and up-regulation of synaptic proteins in the hippocampus. With increasing evidences from parallel studies in our laboratory about the possible role of *Wdr13* in stress response, we investigated its role in brain. We observed that *Wdr13* transcript gets up-regulated in the hippocampus of the wild-type mice exposed to stress. To further dissect its function, we analyzed the behavioral and molecular phenotypes of *Wdr13*^-/0^ mice when subjected to mild chronic psychological stress, namely; mild (attenuated) social isolation. We employed iTRAQ based quantitative proteomics, real time PCR and western blotting to investigate molecular changes. Three weeks of social isolation predisposed *Wdr13*^-/0^ mice to anhedonia, heightened anxiety-measured by Open field test (OFT), increased behavior despair- measured by Forced swim test (FST) and reduced dendritic branching along with decreased spine density of hippocampal CA1 neurons as compared to wild-type counterparts. This depression-like-phenotype was however ameliorated when treated with anti-depressant imipramine. Molecular analysis revealed that out of 1002 quantified proteins [1% False discovery rate (FDR), at-least two unique peptides], strikingly, a significant proportion of synaptic proteins including, SYN1, CAMK2A, and RAB3A were down-regulated in the socially isolated *Wdr13*^-/0^ mice as compared to its wild-type counterparts. This was in contrast to the elevated levels of these proteins in non-stressed mutants as compared to the controls. We hypothesized that a de-regulated transcription factor upstream of the synaptic genes might be responsible for the observed phenotype. Indeed, in the socially isolated *Wdr13*^-/0^ mice, there was an up-regulation of GATA1 – a transcription factor that negatively regulates synaptic genes and has been associated with Major Depression (MD) in humans. The present study demonstrates significant genotype × enviornment interaction for *Wdr13* gene as shown by the reversal in the expression levels of several synaptic proteins in the mutant vis-à-vis wild-type mouse when exposed to social isolation stress.

## Introduction

The *Wdr13* gene, located on the X-chromosome, transcribes a 485 amino acids WD (tryptophan-aspartate) repeat containing protein (Singh et al., 2003; Suresh et al., 2005). The mouse WDR13 is 95% identical to its human counterpart indicating functional conservation of this protein across these species (Suresh et al., 2005). WDR13 is a nuclear protein and interacts with multiple nuclear receptors. To elucidate its function, we had earlier generated and analyzed a mutant mice lacking this gene (Singh et al., 2012). *Wdr13* deficient male mice (*Wdr13*^-/0^) showed age dependant changes in the metabolic parameters, namely; mild obesity and the increased insulin levels. This phenotype could be advanced to an early age if the mice were fed with high-fat diet. In another study we show that there was reduced liver regeneration in *Wdr13*^-/0^ mice, upon insult with hepatotoxin CCl_4_ (Mishra et al., communicated). The absence of *Wdr13* on the other hand led to reduction in progression of cancer in AOM/DSS induced colon cancer primarily through reduction in cell proliferation (Singh et al., 2017).

It may be emphasized that the mutant mice didn’t exhibit any strong overt phenotypes, including mortality under standard laboratory animal management conditions. Since insults in terms of chemical toxins or high fat diet accentuated the phenotypes observed in the absence of *Wdr13*, we hypothesized that WDR13 might have an important physiological function when the system/homeostasis was challenged. We had shown earlier that *Wdr13* has a brain specific function with the mutant mice exhibiting mild anxiety, better memory retention and increased expression of synaptic genes (Mitra et al., 2016). There were other reports that had predicted that *Wdr13* might have a neuro-protective role (Price et al., 2003). Hence, we proceeded to understand whether subjection to psychological insult like stress might affect the homeostasis in the absence of *Wdr13.* Considering the similarity between human and mouse *Wdr13* gene, this investigation would provide us with better understanding of the possible role of this gene in humans and prognosis and treatment of patients carrying mutations in this gene.

There are multiple paradigms for inducing stress in rodents namely social defeat, learned helplessness, social isolation, chronic unpredictable stress (CUS) and psychosocial stress (Campos et al., 2013). Social defeat is a well-validated paradigm for inducing stress in mice but it is limited by the requirement of an aggressor strain. CUS is also an effective method but may not be fully replicable and is sensitive to multiple experimental variables (Argyropoulos and Nutt, 1997; Willner, 2005; Bergström et al., 2007). On the other hand, the prolonged social isolation during adulthood is a potent stressor and results in anhedonia and changes in reward behavior. Though this paradigm has been employed less frequently, it displays excellent construct validity and requires minimal sophistication (Wallace et al., 2009; Wilkinson et al., 2009). Further, this paradigm is akin to social isolation in humans which is an ever increasing risk factor for Major Depression (MD) (Compton et al., 2006). Also, since the duration of stress paradigm in social isolation is for 8 weeks, the number of days can be altered to regulate the effect of stress and study of the onset of depression. Social isolation paradigm also gives the opportunity to analyze multiple strains (in our case CD1 and C57Bl/6J) unlike social defeat. Hence, we decided to employ a protocol for attenuated social isolation as mild chronic stress to study the role of *Wdr13* in brain and behavior.

## Materials and Methods

### Animal Experiments and Ethics Statement

All animal experiments were approved by CCMB Institutional Animal Ethics Committee (Reg. No. CPCSEA 20/1999). We have used *Wdr13*^-/0^ and wild-type male mice in CD1 outbred genetic background for phenotype, histological and molecular analysis. Mice in C57Bl/6J genetic background were also utilized to determine whether the observed phenotype was strain dependant. Mice were housed in central Animal House Facility (CCMB) in polypropylene cages with a 12-h light and dark cycle (6am–6pm light cycle). Shredded corn-cob bedding was used and food and water were provided *ad libitum*. Matings between wild-type male and heterozygous mutant female (heterozygous, *Wdr13*^+/-^) mice provided the experimental animals (littermates and age matched). A schematic representation for all the experiments may be found in **Supplementary Figure S1**.

### Behavioral Paradigms

Normal and socially isolated *Wdr13*^-/0^ and wild-type mice were assessed for any changes in their behavior using sucrose preference test, open field test (OFT), elevated plus maze test and forced swim tests (FSTs). We used video tracking software Noldus EthoVision 3.1 for recording and analysis of the experiments. All behavioral experiments were carried out on 2 months old mice unless otherwise mentioned (**Table 1**).

**Table 1:** Details of cohorts used for experiment.

Cohort (CD1 background)	Number of mice and procedures
Cohort#1	*N* = 6, 2 months age, *Wdr13*^+/0^ and *Wdr13*^-/0^ Social isolation, Sucrose preference, Open field Test (OFT), Forced swim test (FST). OFT and FST were conducted before subjection to social isolation and after social isolation. There was a week of group housing between the OFT and FST and beginning of social isolation
Cohort#2	*N* = 6, 2 months age, *Wdr13*^+/0^ and *Wdr13*^-/0^ non-stressed controls, OFT, FST
Cohort#3	*N* = 6, 2 months age, *Wdr13*^+/0^ and *Wdr13*^-/0^ social isolation, OFT and FST conducted after social isolation
Cohort#4	*N* = 10, 2 months age, *Wdr13*^+/0^ and *Wdr13*^-/0^ non-stressed controls, Sucrose preference, Elevated plus maze
Cohort#5	*N* = 6, 2 months age, *Wdr13*^+/0^ and *Wdr13*^-/0^ social isolation, Elevated plus maze, and FST conducted after social isolation
Cohort#6	*N* = 8, 4 months age, *Wdr13*^+/0^ and *Wdr13*^-/0^ social isolation, FST conducted after social isolation
Cohort#7	*N* = 6, 7 months age, *Wdr13*^+/0^ and *Wdr13*^-/0^ social isolation, FST after social isolation
Cohort#8	*N* = 7, 2 months age, *Wdr13*^+/0^ and *Wdr13*^-/0^ non-stressed control for imipramine experiment, FST
Cohort#9	*N* = 7, 2 months age, *Wdr13*^+/0^ and *Wdr13*^-/0^ social isolation group for imipramine experiment-sham treatment, FST after social isolation
Cohort#10	*N* = 7, 2 months age, *Wdr13*^+/0^ and *Wdr13*^-/0^ social isolation group for imipramine experiment – Imipramine treatment, FST after social isolation and imipramine treatment

#### Open Field and Forced Swim Tests

##### Open field test (OFT)

The OFT was carried out by placing the mice in an open square box of dimensions, 50 cm × 50 cm in a brightly lit room (around 120–150 lux; white light). The duration the mice spent in the virtually marked central area of the box was measured. The total duration of the trial was for 5 min.

##### Forced swim test (FST)

Forced swim test or Porsolt swim test (Porsolt et al., 1977) was conducted in mice by placing them in a beaker filled with 20–25 cm deep water and the total duration of immobility was measured. The temperature of the water was around 25**°**C. Experiments were video-recorded for 5 min (300 s) and analyzed later.

#### Elevated Plus Maze (EPM) Test

In this test, the mice were placed in an elevated platform having two closed arms and two open arms in a brightly lit room (around 120–150 lux; white light). The duration mice spent in the arms were analyzed. The total duration of trial was 5 min.

#### Sucrose Preference Test

Mice have an inborn preference for sweet foods and solutions. In depressed mice, brain reward pathway is not activated despite stimulation by sweet solutions (Strekalova et al., 2004). Thus, decrease in preference for sucrose solution over water is taken as an indicator of depression in mice. All mice were kept with two bottles per cage. Mice were first habituated for 2 days with water in both bottles followed by 2 days with 2% sucrose solution in both bottles. There after one bottle of water and one with sucrose solution was given to them. To avoid place preference of the mouse positions of bottles of water and sucrose were switched daily during the course of the stress period. Bottle weights were taken every day to determine sucrose preference. Sucrose preference was measured for 4 days for non-stressed groups and for 5 days of the final period of social isolation for socially isolated group.

#### Social Isolation Experiment (SI)

Social isolation experiment was adapted with modifications from that described by Wilkinson et al. (2009). In the present study, a mouse was kept solitary in a non-transparent cage for a period of 3 weeks instead of longer durations of 8 weeks as described in original protocol. Two bottles of water and feed *ad libitum* were added prior to start of the experiment. At the end of 3 weeks, OFT and FST were conducted to ascertain the level of anxiety/depression in these mice.

### Imipramine Treatment

Treatment group mice (seven each) were injected 20 mg/kg Imipramine (SIGMA; dissolved in 0.9% saline) I.P. for 5 days after social isolation (short treatment) (Aulakh et al., 1987; Kumar and Garg, 2009; Jangra et al., 2016). Control group mice (seven each) received saline I.P. injections after social isolation.

### Proteomic Analysis

8plex iTRAQ based quantitative proteomics analysis was carried out on proteins isolated from pre-frontal cortex (PFC) of normal and socially isolated *Wdr13*^-/0^ and wild-type mice. The mass spectrometry proteomics raw data along with the list of the quantified proteins and peptide lists have been deposited with ProteomeXchange Consortium via the PRIDE partner repository with the dataset identifier PXD004545. 4plex iTRAQ based quantitative proteomics analysis was carried out on proteins isolated from hippocampus of socially isolated *Wdr13*^-/0^ and wild-type mice. Dissection of desired brain regions were done 4 days after behavioral procedures.

#### iTRAQ 8-Plex

Pre-frontal cortices were dissected from 1mm thick sections derived from the brain of experimental mice using a matrix (slicer). We pooled pre-frontal cortices (PFC) from three normal and three socially isolated mice from each of the two groups, viz.; wild-type (*Wdr13*^+/0^) and knockout (*Wdr13*^-/0^). Protein was extracted with 0.5% SDS and quantified using BCA protein estimation kit. 200 μg of protein was digested using trypsin as described previously (Mitra et al., 2016). 80 μg of samples were split into two groups and each group was labeled with iTRAQ-8plex (Applied Biosystems, Foster City, CA, United States) for technical duplicates and as per manufacturers protocol. Peptides from wild-type mice were labeled with 113 and 114 tags, from knockout with 115 and 116 tags, from socially isolated wild-type with 117 and 118 tags and from socially isolated knockout with 119 and 121 tags. We carried out 8-plex labeling and desalting experiments as per manufacturer’s protocols. To increase the number of detections of peptides and proteins, fractionation was carried out using Strong Cation Exchange (SCX) before de-salting using C_18_ column (Pierce/ThermoFisher).

#### LC-MS/MS and Data Analysis

Peptides were separated using linear gradient from 5 to 98% of buffer B (95% acetonitrile and 0.1% formic acid) at a flow rate of 300 nl/min followed by a column re-equilibration reaching 5% of buffer B for a few minutes. Gradient length was adjusted to 90 min. Data acquisition was done using Xcalibur 2.1 (Thermo Fisher Scientific, Bremen, Germany). MS spectra, mass range, scan resolution for MS, MS/MS, Normalized collision energy (NCE), precursor ion selection, the dynamic exclusion parameters were kept the same as described in iTRAQ 4-plex experiments (Mitra et al., 2016). The nano source spray was set at 2.2 KV and the capillary temperature at 250°C without sheath gas. Isolation width was adjusted. Data analysis for 8-plex experiments was done using Proteome Discoverer 1.3 (Thermo Fisher Scientific, Bremen, Germany) software. We acquired total 80900 MS/MS scans. False discovery rate (FDR) was calculated by enabling the peptide sequence analysis using decoy database and top ranked hit based on peptide score, XCorr for Sequest. 1% FDR was applied in our analysis and only proteins with at-least 1 unique peptide were considered. For further bioinformatics analysis proteins with at-least 2 unique peptides were selected. While performing analysis only 116 labels was considered for *Wdr13*^-/0^ under non-stress condition. ±1.2-fold change in protein level was taken as cut-off (Parker et al., 2015; Chen et al., 2016).

#### iTRAQ 4-Plex

We pooled hippocampus from three socially isolated mice from each of the two groups, viz.; wild-type (*Wdr13*^+/0^) and knockout (*Wdr13*^-/0^). The protocol followed was similar to the iTRAQ 8-plex and as previously described (Mitra et al., 2016). Socially isolated wild-types were labeled with 114, 115 and socially isolated mutant mice were labeled with 116, 117 labels.

### Golgi Cox Staining

Golgi Cox staining was carried out on brain sections of 100 μm thickness using a previous protocol (Chakravarty et al., 2015). A total of six each of unstressed wild-type and *Wdr13*^-/0^ mice, and, five wild-type and six *Wdr13*^-/0^ socially isolated mice were studied. A minimum of six CA1 neurons from 3-5 hippocampal sections for each mouse (Magariños et al., 1996) were analyzed for dendritic branching. CA1 neurons were selected as described in previous reports (Krugers et al., 2010; Wang and Michaelis, 2010). Sholl analysis was carried out using NeuronJ plugin and Sholl Analysis plugin (v1.0) of ImageJ software.

### Cell Culture and Transduction

Human neuroblastoma cell line IMR32 was cultured using DMEM media supplemented with 10% Fetal Bovine Serum and antibiotics. Overexpression of WDR13 in IMR32 cell line was achieved through transduction with adenovirus AdWdr13 (100MOI) (Singh et al., 2012) in BSLII Cell Culture Facility.

### Primer Designing, Real Time Analysis

Primers were designed using Primer3 software or selected from Primer Bank (**Table 2**). RNA was isolated from PFC, hippocampus and nucleus accumbens using Trizol (Thermo Fisher Scientific) and cDNAs were prepared using protocol listed elsewhere (Sambrook and Russell, 2001). ABI Prism SDS 7000 and ABI 3900 HT were employed to perform real time PCR using SYBR green 2X mix (Invitrogen and Thermo Fisher Scientific) as per manufacturer’s protocol and described previously (Singh et al., 2012, 2015).

**Table 2:** List of primers.

Gene	Forward (5′–3′)	Reverse (3′–5′)
*Mapt*	TGAGGGACTAGGGCAGCTAA	CAGTCCACCCATCCATCTCT
*Mapt2*	TCCGATGCTAAGAGCACTCC	GAGCTTGAGTCACATGCCCA
*Kcnh2*	CTCATGACACCAACCACAGG	GTTGTCCATGGCAGAAACCT
*Cdca3*	TTCTCAAAGCTGGAGGAGGA	AGAGTTGGTGCCTGAGAGGA
*Gfap*	GGGGCAAAAGCACCAAAGAAG	GGGACAACTTGTATTGTGAGCC
*Pax2*	AAGCCCGGAGTGATTGGTG	CAGGCGAACATAGTCGGGTT
*Crebbp*	GGCTTCTCCGCGAATGACAA	GTTTGGACGCAGCATCTGGA
*Bdnf*	TCATACTTCGGTTGCATGAAGG	AGACCTCTCGAACCTGCCC
*Ngf(a)*	CCAGTGAAATTAGGCTCCCTG	CCTTGGCAAAACCTTTATTGGG
*Tgfb1*	CTCCCGTGGCTTCTAGTGC	GCCTTAGTTTGGACAGGATCTG
*Grin2a*	ACGTGACAGAACGCGAACTT	TCAGTGCGGTTCATCAATAACG
*Grin2b*	GCCATGAACGAGACTGACCC	GCTTCCTGGTCCGTGTCATC
*Syn1*	AGCTCAACAAATCCCAGTCTCT	CGGATGGTCTCAGCTTTCAC
*Syn2*	TGACAAATGCGTTCAGCTTC	TAGATGCCTTTCCTGGTTGG
*Fos*	CGGGTTTCAACGCCGACTA	TTGGCACTAGAGACGGACAGA
*Nrxn1*	AACGGACTGATGCTTCACACA	CCTGAGTGCTGACGCAGATT
*Nrxn2*	GCTCTGCATCCTCATTCTCC	TGTTCTTCTTGGCCTTGCTT
*Camk2a*	TGCCTGGTGTTGCTAACCC	CCATTAACTGAACGCTGGAACT
*Rab3a*	GTGGGCAAAACCTCGTTCCT	TCCTCTTGTCGTTGCGGTAGA
*Sv2a*	GTCTGGTTTCCCGACATGAT	CCTCAAACAGGGAATCCTCA
*Calm2*	ACGGGGATGGGACAATAACAA	TGCTGCACTAATATAGCCATTGC
*Rab4b*	CATCGTGGTCATCCTCTGTG	ACTGAATGCCTGAACCCATC
*Gata1*	TGGGGACCTCAGAACCCTTG	GGCTGCATTTGGGGAAGTG
*Mzf1*	GAAGAGACGTCCAGGAGTGC	AGTCCTCCAGCTTCACCAGA
*Nf-1*	TGCTGATGCTGTCCTTCAAC	GAGCCTCAAAACTTGCTTGG
*Pur1*	ATCCGCCAGACAGTCAACC	TCCACTCCATAGTCGTCGATG
*Ikzf1*	AGACAAGTGCCTGTCAGACAT	CCAGGTAGTTGATGGCATTGTTG
*Klf4*	CCACCAGGACTACCCCTACA	GACCTTCTTCCCCTCTTTGG
*Areb*	GGGGCATCTCACACTTTTGT	AACGGCTGTGAACCAAAAAC

### Western Blotting

Brain tissues were homogenized and lysed in SDS-Lysis buffer and western blotting was performed using a protocol described in our previous work (Singh et al., 2012). Samples (50 μg protein) were blotted using antibodies against GATA1 (ab28839, Abcam; 1:500 in 5% BSA), NRXN2 (ABN97, Milipore; 1:400 in 5% BSA), CAMK2A (Prestige Antibody, Sigma, 1:300 in 5% BSA), and β-ACTIN (sc47778, SantaCruz; 1:1000 in 5% BSA).

### Experimental Design

A schematic of the design of the experiments have been included in **Table 1** and **Supplementary Figure S1**.

### Statistical Analysis

Students unpaired *t*-test and one or two-way analysis of variance (ANOVA) were applied to understand statistical significance. For samples with *n* > 5, data are presented as mean ± SEM. For samples with *n* < 5, non-parametric tests like Mann–Whitney have been performed and data are represented as mean ± SD.

## Results

**FIGURE 1:**
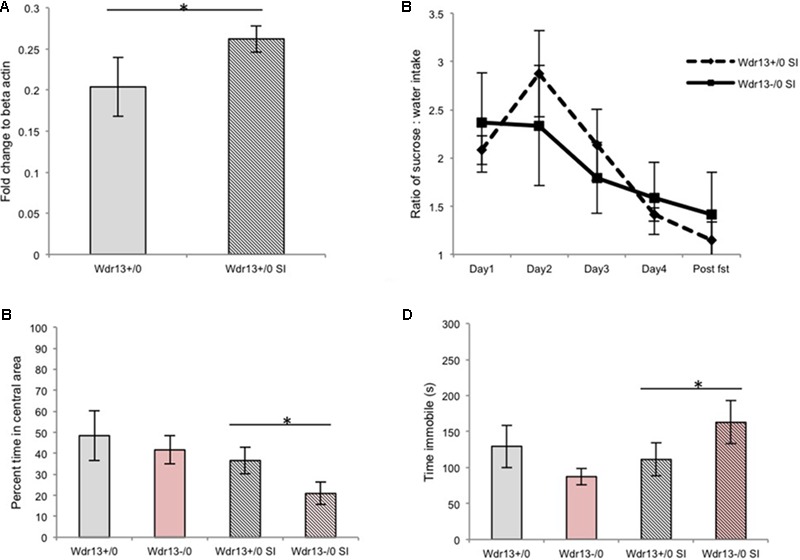
Effect of social isolation for 3 weeks on sucrose preference, open field and forced swim tests (FSTs) in absence of *Wdr13* gene in mice. **(A)** *Wdr13* expression showed increase (Mann–Whitney; *p* < 0.05) in the hippocampus of wild-type mice upon social isolation (*n* = 4). Data represented as ±SD. **(B)** Sucrose preference test (Cohort#1). After social isolation, both *Wdr13*^-/0^ and wild-type mice showed anhedonia [Two-way repeated ANOVA, effect of days on both genotype *F*(4,40) = 3.775; *p* = 0.0107| interaction *F*(4,40) = 0.47; *p* > 0.05| between genotypes *F*(1,40) = 0.017; *p* > 0.05|*n* = 6]. **(C)** Open field test (OFT) (Cohort#1). *Wdr13*^-/0^ mice showed significant decrease [individual *t*-test; *p* < 0.05|| Two-way repeated ANOVA, effect of stress on genotypes *F*(1,20) = 5.757; *p* = 0.0263| interaction *F*(1,20) = 0.4292; *p* > 0.05| between genotypes *F*(1,20) = 2.736; *p* > 0.05] in exploration of central area of open field after social isolation than the wild-type mice (*n* = 6). **(D)** FST (Cohort#1). Socially isolated *Wdr13*^-/0^ mice remained immobile for more time [individual *t*-test; *p* < 0.05|| Two-way repeated ANOVA, interaction *F*(1,20) = 5.467; *p* = 0.0299] than their wild-type mice counterparts (*n* = 6). *Wdr13^+/0^*, wild-type; *Wdr13*^-/0^, *Wdr13* knockout mice SI, Social Isolation. ^∗^ denotes *p* < 0.05 and ^∗∗^ denotes *p* < 0.005.

Based on our previous experiments, we hypothesized that WDR13 might have an important role under stress conditions. In brain, *Wdr13* expresses prominently in the hippocampus, amygdala, cortex, PFC and cerebellum (Mitra et al., 2016). We had earlier studied the hippocampus for structural and molecular changes associated with the absence of *Wdr13*. The hippocampus is one of the regions of the brain which gets affected by stress and has been related to psychiatric disorders, viz.; anxiety and depression (Kim et al., 2006; Conrad, 2008). To ascertain the effect of social isolation on *Wdr13* gene expression, we analyzed the hippocampus of socially isolated wild-type male mice and found that *Wdr13* transcripts were significantly upregulated (Mann–Whitney; *p* < 0.05; **Figure 1A**). These data prompted us to undertake a detailed investigation into the possible role of WDR13 in chronic social isolation stress.

### Socially Isolated *Wdr13*^-/0^ Mice Showed Decreased Exploration of Central Area of Open Field Test (OFT) and Increased Immobility in Forced Swim Test (FST)

Earlier researches have shown that social isolation for a period of 8 weeks resulted in symptoms of major-depression like phenotype in the wild-type mice (Wilkinson et al., 2009; Volden et al., 2013). Since we intended to study whether *Wdr13*^-/0^ mice were predisposed to chronic stress as compared to the wild types, we decided to curtail the duration of the stress to 3 weeks, such that it would act as a stressor but wouldn’t induce major-depression-like phenotype associated with hightened anxiety and behavioral despair in wild-type mice. Both the *Wdr13*^-/0^ and wild-type mice subjected to 3 weeks of social isolation exhibited lower preference for sucrose in sucrose preference test (Two-way repeated ANOVA, effect of days on both genotype *F*(4,40) = 3.775; *p* = 0.0107| interaction *F*(4,40) = 0.47; *p* > 0.05 | between genotypes *F*(1,40) = 0.017; *p* > 0.05|*n* = 6) indicating anhedonia (**Figure 1B** and **Supplementary Figure S2**). Mice from Cohort#1 were analyzed for OFT and FST followed by a week of no activity before subjection to social isolation; at the end of which they were again analyzed for the same paradigms. The socially isolated *Wdr13*^-/0^ mice traversed significantly less time [individual *t*-test; *p* < 0.05|| Two-way repeated ANOVA, effect of stress on genotypes *F*(1,20) = 5.757; *p* = 0.0263| interaction *F*(1,20) = 0.4292; *p* > 0.05| between genotypes *F*(1,20) = 2.736; *p* > 0.05] in the central area of OFT (**Figure 1C**) than their wild-type counterparts. There was however no significant difference (*t*-test; *p* > 0.05) in total distance traveled between socially isolated wild-type and mutant mice (**Supplementary Figure S3A**). In addition, under FST, the mutant mice showed greater immobility as compared to the wild-type [individual *t*-test; *p* < 0.05;|| Two-way repeated ANOVA, interaction *F*(1,20) = 5.467; *p* = 0.0299; **Figure 1D**]. Consistent with our experimental design, the socially isolated wild-type mice didn’t show any significant changes in OFT and FST indicating that 3 weeks social isolation didn’t induce hightened anxiety and behavioral-despair-like-phenotype in wild-type unlike in mice lacking *Wdr13*. It may be noted that wild-type mice however did show anhedonia. In this context it may be noted that multiple researches have shown that anhedonia to be independent of anxiety and/or behavioral despair like behavioral phenotype as measured by OFT and FST respectively (Strekalova et al., 2004; Slattery and Cryan, 2014; Soga et al., 2015).

To validate our findings of effect of social isolation in *Wdr13*^-/0^ mice, we repeated the experiment twice with independent cohorts of mice (for stressed and non-stressed groups) and got similar results (**Supplementary Figures S3B–D**). Interestingly, socially isolated *Wdr13*^-/0^ mice also spent increased time in closed arm of Elevated plus maze (*t*-test; *p* < 0.05; **Supplementary Figures S3E,F**) indicating hightened anxiety. Differential response of *Wdr13*^-/0^ mice upon social isolation, i.e., increased immobility in FST was also prominent at four (*t*-test; *p* < 0.05) and seven (*t*-test; *p* < 0.005) months of age indicating that this phenotype was not age dependant (**Supplementary Figures S4A,B**). Mutant mice in C57Bl/6J background also showed similar behavior indicating that the observed phenotype was not strain specific either (*t*-test; *p* < 0.05; **Supplementary Figure S4C**). Thus, the absence of *Wdr13* predisposed these mice to hightened anxiety and behavioral despair as measured by OFT and FST upon subjection to 3 weeks social isolation.

### Downregulation of Synaptic Genes in *Wdr13*^-/0^ Brain After Social Isolation

The hippocampus, amygdala and PFC are known to be affected by stress and involved in depression (Bremner, 2006). Particularly, the PFC is vulnerable to stress, which is associated with distinct molecular and histological changes (McEwen and Morrison, 2013). We had earlier reported that *Wdr13* expression was prominent in PFC in addition to hippocampus (Mitra et al., 2016). To study the molecular changes associated with susceptibility of *Wdr13*^-/0^ mice to increased anxiety and depression, and further to determine whether such changes were similar to those found in other rodent models of depression (Wilkinson et al., 2009) or depressed human post-mortem samples (Player et al., 2013; Fuchsova et al., 2015), we investigated the molecular changes in the PFC of the socially isolated *Wdr13*^-/0^ mice using 8-plex iTRAQ quantitative proteomics.

**FIGURE 2:**
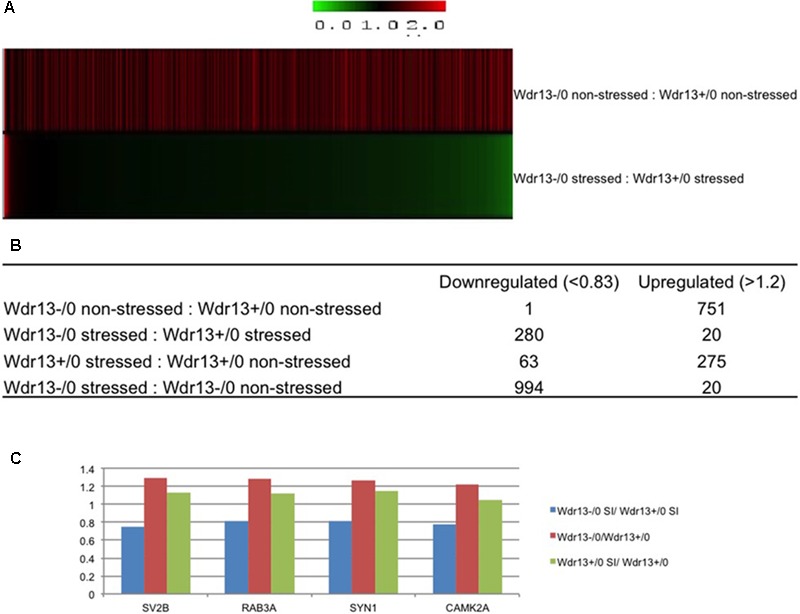
8plex ITRAQ based quantitative proteomics of pre-frontal cortex (PFC) from wild-type and *Wdr13*^-/0^ mice with and without social isolation. **(A)** Heat map representing proteomics data from PFC of *Wdr13*^-/0^ mice and wild-type mice before and after social isolation (1% FDR, at-least one unique peptide). **(B)** Table depicting changes in the proteome. Out of 1722 quantified proteins, there was a significant proportion of downregulated proteins in *Wdr13*^-/0^ mice after social isolation as compared to wild-type (1.2-fold as upregulated and less than 0.83 as downregulated). **(C)** Relative levels of four synaptic proteins that showed downregulation in socially isolated *Wdr13*^-/0^ mice than wild-type counterparts, while showed upregulation under normal conditions. It may be noted that levels of these proteins remained unchanged in socially isolated wild-type. *Wdr13*^+/0^, wild-type; *Wdr13*^-/0^, *Wdr13* knockout mice; SI, Social Isolation.

We compared both the non-stressed and stressed wild-type and *Wdr13*^-/0^ mice to examine changes in protein levels resulting from: (a) social isolation stress, (b) loss of *Wdr13*, and (c) loss of *Wdr13* plus social isolation stress (**Supplementary Table S1** – at-least one unique peptide, **Supplementary Table S2** – atleast two unique petides). Analysis of 1722 quantified proteins at 1% FDR (at-least one unique peptide) revealed very contrasting changes in the expression of proteins when *Wdr13*^-/0^ mice were subjected to stess as compared to the wild-types subjected to similar stress (**Figure 2A** and **Supplementary Figure S5**). Loss of *Wdr13* gene *per se* in mutant mice resulted in upregulation of 751 proteins as compared to their wild-type littermates. On the contrary, the loss of *Wdr13* in mice when accompanied by stress, led to down-regulation of 280 proteins, and only 20 proteins were up-regulated as compared when wild-type type mice were subjeced to stress (**Figure 2B**). Interestingly, synaptic proteins like SYN1 (**Supplementary Figure S6**), RAB3A, SV2B, and CAMK2A (**Supplementary Figure S7**) which were found to be up-regulated in the hippocampus and PFC of un-stressed *Wdr13*^-/0^ mice in our previous (Mitra et al., 2016) and present study, were downregulated in the PFC of the *Wdr13*^-/0^ mice after social isolation (**Figure 2C** and **Supplementary Figure S5B**). Real time analysis also showed that there was a decrease in the transcript levels of the synaptic genes *Rab3a* and *Nrxn2* in the socially isolated *Wdr13*^-/0^ mice PFC as compared with their wild-type counterparts (**Supplementary Figure S8A**). Additionally, Western blot analysis confirmed that the protein levels of another synaptic protein NRXN2 were accordingly down-regulated in *Wdr13*^-/0^ mice (**Supplementary Figure S8D**). This indicated that stress had a profound influence on the phentotype – behaviorally and at molecular level of *Wdr13*^-/0^ mice. Taken together, there was a strong evidence for genotype × enviornment interaction for *Wdr13* gene as revealed by the reversal in the expression of several genes in *Wdr13*^-/0^ mice as a result of the social isolation stress as compared to wild-type counterparts and in non-stressed environment.

**FIGURE 3:**
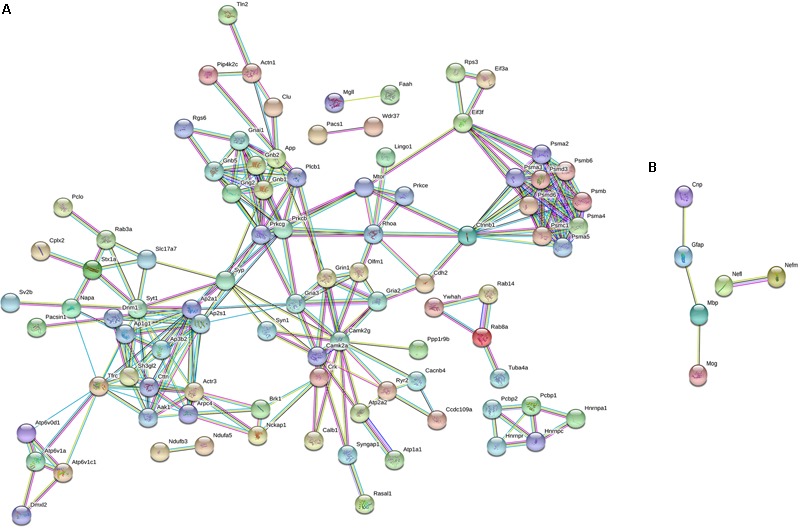
String analysis of differentially expressed proteins [1% False discovery rate (FDR) and at-least 2 unique peptides] from PFC of socially isolated *Wdr13*^-/0^ and wild-type mice. **(A)** Down-regulated proteins and **(B)** Up-regulated proteins. Parameters of high confidence have been applied and only connected nodes are displayed.

To characterize the molecular changes associated with the depression-like phenotype, we investigated in detail the downregulated and upregulated proteins in the socially isolated *Wdr13*^-/0^ mice. To further refine our analysis, we selected proteins with at-least two unique peptides (1002 proteins; **Supplementary Table S2**). We performed String analysis of the down-regulated (164) proteins as well as the upregulated (13) proteins (**Figure 3**). Multiple synaptic proteins were found among the downregulated group. Infact, delienation of downregulated proteins in Biological Processes revealed that the synaptic transmission process comprising 18 proteins from our list was affected with a FDR < 0.005 (**Supplementary Table S3**). Similarly KEGG Pathway analysis revealed that glutamatargic synapse, synaptic vesicle cycling and dopaminargic synapse were among the top 10 downregulated pathways with a FDR < 0.005 (**Supplementary Table S4**). As stated earlier these data were in contrast with upregulation of multiple synaptic genes in the non-stressed *Wdr13*^-/0^ mice as compared to wild-type conterparts as found in this study (**Figure 2A**, **Supplementary Table S1**, and **Supplementary Figure S5B**) and our previous report (Mitra et al., 2016). In addition to synaptic pathways, we also found proteasome pathways with proteins like PSMA2, PSMB6, PSMC1, etc., to be downregulated in the socially isolated *Wdr13*^-/0^ mice as compared their wild-type conterparts (**Figure 3A** and **Supplementary Table S1**).

Among the 20 up-regulated proteins, there was a significant increase in the levels of neurofilaments NEFL and NEFM along with GFAP in *Wdr13*^-/0^ mice subjected to stress (**Figure 3B**).

**FIGURE 4:**
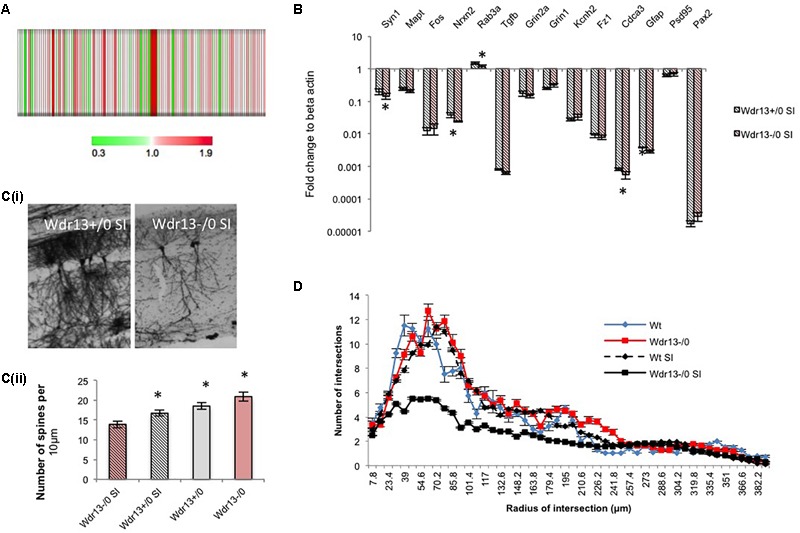
Expression analysis of synaptic genes and histological examination of hippocampus in *Wdr13*^-/0^ mice after 3 weeks social isolation. **(A)** Heat map of quantified proteins from hippocampus of socially isolated *Wdr13*^-/0^ mice using iTRAQ based proteomics. **(B)** Transcript levels of synaptic genes like *Nrxn2, Rab3a, Syn1* showed decrease in the *Wdr13*^-/0^ hippocampus (*t*-test; *p* < 0.05) than the wild-type (*n* = 5). **(C) (i)** Representative images showing CA1 neurons in wild-type and *Wdr13*^-/0^ mice after social isolation. **(ii)** Spine density of CA1 neurons in different groups (individual *t*-test; *p* < 0.05). **(D)** The dendritic branching of hippocampal CA1 neurons in *Wdr13*^-/0^ mice was significantly lesser (Two-way ANOVA, *F*(1,3900) = 8806; *p* < 0.005) than the wild-type mice after social isolation. *Wdr13^+/0^*, wild-type; *Wdr13*^-/0^, *Wdr13* knockout mice; SI, Social Isolation. ^∗^ denotes *p* < 0.05 and ^∗∗^ denotes *p* < 0.005.

To determine whether the above molecular changes seen in the PFC of the socially isolated *Wdr13*^-/0^ mice were similar to those in other regions of the brain, particularly where *Wdr13* expresses prominently, we carried out 4plex iTRAQ based proteomics comparing socially isolated wild-type and mutant hippocampus (**Figure 4A**). We also performed real time PCR analysis for multiple synaptic and synapse associated genes from hippocampus (**Figure 4B**) and nucleus accumbens (NA) (**Supplementary Figure S8C**) of the socially isolated wild-type and *Wdr13*^-/0^ mice. From our proteomics experiment we found that CAMK2A was downregulated in the hippocampus of socially isolated *Wdr13*^-/0^ mice as compared to its wild-type counterpart (**Figure 4A** and **Supplementary Table S5**). This was similar to the data obtained from analysis of proteins of PFC. We validated this with western blot against CAMK2A (**Supplementary Figure S8B**). Consistent with our proteomics data and the transcript analysis from PFC, there was a significant downregulation in expression of synaptic genes in the hippocampus and NA as well, namely, *Syn1, Rab3a*, and *Nrxn2* in the socially isolated *Wdr13*^-/0^ mice (individual *t*-test; *p* < 0.05; **Figure 4B** and **Supplementary Figure S8C**). However, we found no significant changes (*t*-test; *p* > 0.05) in the transcript levels of NMDA receptors- *Grin1* and *Grin2* and immediate early gene *Fos* (*t*-test; *p* > 0.05; **Figure 4B** and **Supplementary Figure S8C**).

Having ascertained that the molecular changes observed were global in nature, we wanted to study if there were any histological changes associated with this phenotype in socially isolated *Wdr13*^-/0^ mice. We chose to analyze the hippocampal CA1 neurons as they have been reported to be susceptible to chronic stress which is manifested in the form of altered dendritic branching and complexity, and have been related to depression (Krugers et al., 2010; Wang and Michaelis, 2010). We have earlier shown that there was no significant difference in dendritic branching of CA1 neurons between the wild-type and *Wdr13*^-/0^ mice and there was an increase in spine density in the mutant mice (Mitra et al., 2016). However, in the present study, there was a marked decrease (Two-way ANOVA, *F*(1,3900) = 8806; *p* < 0.005) in dendritic arborations and spine density (individual *t*-test; *p* < 0.05) of hippocampal CA1 neurons in the socially isolated *Wdr13*^-/0^ mice (**Figures 4C(i,ii),D**). These data were in sync with our findings of increased anxiety and depression-like sysmptoms, and decreased synaptic proteins in the socially isolated *Wdr13*^-/0^ mice.

### Upregulation of GATA1, a Common Negative Transcription Factor in the Brain of *Wdr13*^-/0^ Mice Following Social Isolation

**FIGURE 5:**
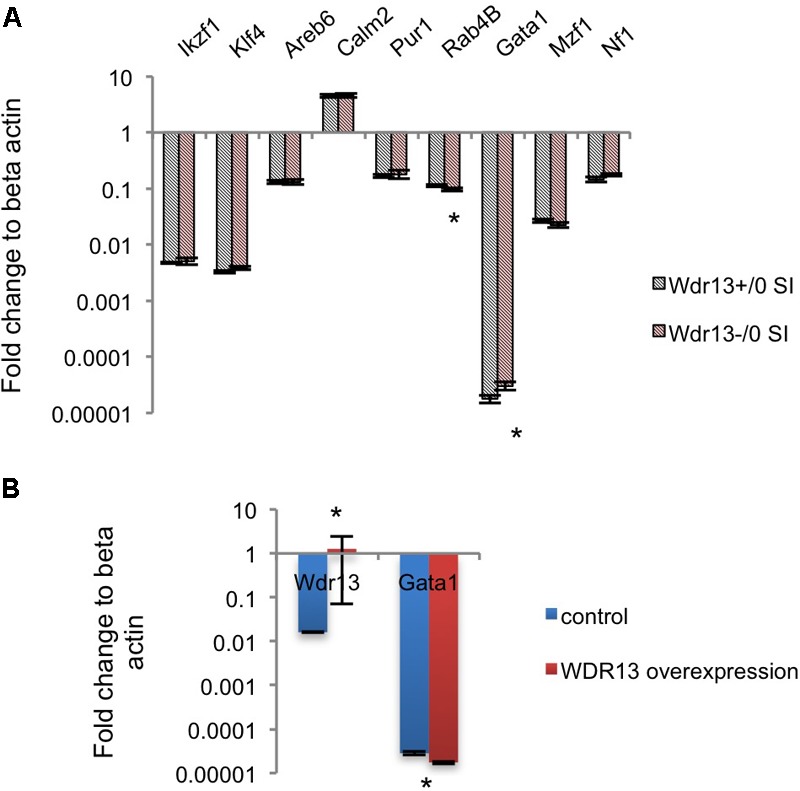
Effect of WDR13 on expression of transcription factors regulating synaptic genes. **(A)** Expression analysis of common transcription factors of downregulated synaptic genes in social isolated *Wdr13*^-/0^ and wild-type mice hippocampus. *Gata1* transcript level increased significantly (*t*-test; *p* < 0.05) in *Wdr13*^-/0^ mice (*n* = 5). **(B)** Effect of overexpression of WDR13 on *Gata1* transcript levels in IMR32 cells. *Wdr13^+/0^*, wild-type; *Wdr13*^-/0^, *Wdr13* knockout mice; SI, Social Isolation. ^∗^ denotes *p* < 0.05 and ^∗∗^ denotes *p* < 0.005.

We were intrigued by the contrasting changes in the expression of synaptic genes in the socially isolated *Wdr13*^-/0^ mice vis-à-vis the corresponding changes in wild-types and in non-stressed conditions (**Figures 2A,B** and **Supplementary Figure S5B**). We predicted that there might be a deregulated master regulator/transcription factor that eventually contributed to the downregulation of several synaptic genes in *Wdr13*^-/0^ mice in response to social isolation stress. It was interesting to note that our proteomics data were similar to expression data in the post-mortem samples from patients suffering from Major Depression (Kang et al., 2012). We analyzed expression of the major transcription factors known to regulate synaptic genes (Kang et al., 2012). In our efforts to find a de-regulated transcription factor acting as a master regulator of synaptic genes in *Wdr13*^-/0^ mice, we noted that *Gata1* transcripts in the PFC (**Supplementary Figure S8A**) and hippocampus (**Figure 5A**), as well as corresponding protein levels (**Supplementary Figure S8D**) in the socially isolated *Wdr13*^-/0^ mice were upregulated (*p* < 0.05). This was similar to the expression profile of GATA1 as was shown by Kang et al. (2012) for Major Depression in humans. These authors had also analyzed multiple common transcription factors of synaptic genes in a bid to discover a master regulator that might be effecting their expression and they provided evidence that *Gata1* was the most likely candiate. Our results predicted a possible role of WDR13 in regulation of GATA1 and it appeared likely that the absence of *Wdr13* predisposed mutant mice to Major Depression-like phenotype when subjected to social isolation stress through this master regulator.

### Regulation of GATA1 by WDR13?

Upon social isolation, the transcript levels of *Wdr13* were increased inthe hippocampus of wild-type mice (Mann–Whitney; *p* < 0.05; **Figure 1A**). Also the transcript levels of *Gata1* were lower in socially isolated wild-type mice, though, the difference was not statistically significant (Mann–Whitney; *p* > 0.05; **Supplementary Figure S8F**). Further, overexpression of WDR13 in IMR-32 human neuroblastoma cell line decreased the transcript levels of *Gata1* by 1.5-fold (Mann–Whitney; *p* < 0.05; **Figure 5B**).

However, it may be noted that the loss of WDR13 in non-stressed *Wdr13*^-/0^ mice did not result in any significant difference in the transcript levels of *Gata1* in their hippocampus as compared that in wild-type mice (Mann–Whitney; *p* > 0.05; **Supplementary Figure S8E**). On the contrary, as shown, in socially isolated *Wdr13*^-/0^ mice, *Gata1* transcript levels increased significantly.

### Effect of Antidepressant

Acute imipramine treatment (**Supplementary Figure S9A**) of the socially isolated mutant mice was able to alleviate the depression like phenotype as assessed through behavioral despair model of FST [**Supplementary Figure S9B**; 2 way ANOVA, *F*(2,36) = 6.353; *p* < 0.005]. This result demonstrated that the absence of *Wdr13* did not result in any irreversible changes at the molecular/transcriptional level but rather had pre-disposed the mutant mice to depression-like-phenotype. Imipramine treatment also resulted in downregulation (independent *t*-test; *p* < 0.05; **Supplementary Figure S9C**) of *Gata1* transcript in socially isolated *Wdr13*^-/0^ mice bringing it closer to physiological levels as observed in wild-type mice.

## Discussion

We have shown that *Wdr13*^-/0^ mice when subjected to 3 weeks of social isolation stress resulted in a phenotype exhibiting anhedonia, heightened anxiety and heightened behavioral despair. There was down-regulation of multiple synaptic genes like *Syn1, Camk2a, Rab3a, Nrxn2*, etc, at transcript and protein levels, after social isolation in the mutant mice along with decreased arboration of hippocampal CA1 neurons. These observations are akin to classical definition of etio-pathology of depression (Hasler et al., 2004). We also found that the absence of *Wdr13* led to up-regulation of GATA1 when mice were subjected to mild social isolation stress. GATA1 is known to repress transcription of synaptic genes like *Syn1, Camk2a*, and *Rab3a*, and associated with Major Depressive Disorder in human patients (Kang et al., 2012). Thus, taken together behavioral, anatomical and molecular changes obeserved in the socially isolated *Wdr13^-^*^/0^ mice were reminiscent of Major Depression (MD) in human beings (Hales et al., 2014). Taking into account that the experiments were performed in 2–3 months old *Wdr13*^-/0^ mice where no notable changes in brain (Mitra et al., 2016) or systemic (Singh et al., 2012) metabolism were observed, and also that this phenotype wasn’t age or strain dependant, the results of the present study were indicative of direct effects of WDR13 deficiency in brain rather than secondary effects.

The current work finds that after the social isolation stress, *Wdr13* transcript levels increased in the wild-type mice and the loss of the gene resulted in MD like phenotype. Interestingly, epigenetic analysis in mice models of depression have showed that the repressive chromatin marks on *Wdr13* decreased upon stress (Wilkinson et al., 2009) indicating the possible up-regulation of this gene in response to stress. *Wdr13* transcript levels have also been shown to increase upon hippocampal lesion (Price et al., 2003). Collectively, these data suggest that the induction of *Wdr13* transcription occurs upon exposure to stress and WDR13 might have a neuro-protective role. This leads to the question that how WDR13 is responsive to stress? What are the transcription factor(s) that might be responsible for this? Analysis of elements binding upstream of *Wdr13* gene would shed light on the regulation of this gene and also provide more information on stress response.

To investigate the changes associated with the depression like phenotype exhibited by the socially isolated *Wdr13*^-/0^ mice, we performed iTRAQ based quantitative proteomics of the PFC and used real time PCR and western analysis to validate the changes in other regions of brain, i.e., hippocampus and nucleus accumbens. We found several proteins to be dysregulated in *Wdr13*^-/0^ mice upon social isolation as compared to the wild-type counterparts. A significant proportion of the quantified proteins were down-regulated in the PFC of socially isolated *Wdr13*^-/0^ mice, of which the synaptic proteins formed a higher proportion of the proteins. Down-regulation of synaptic genes has been reported earlier from rodent and human patient samples linked to Major Depression (Kang et al., 2012; Fuchsova et al., 2015). Downregulation of synaptic genes like *Syn1, Rab3a, Camk2a, Nrxn2* have been strongly associated with major depression (Duman and Aghajanian, 2012; Kang et al., 2012; Duman et al., 2016). Deficiency in CAMK2A protein has been linked to psychiatric disorders (Yamasaki et al., 2008) indicating its importance in the observed phenotype. Decrease in *Rab4B* (**Figure 5A**) might be responsible for decreased dendritic arboration of CA1 hippocampal neurons (Brown et al., 2007) in the socially isolated *Wdr13*^-/0^ mice. Apart from synaptic proteins, we have also found other interesting molecules like proteins belonging to the proteasome pathway (PSMA2, PSMC1, PSMB6, etc.) to be down-regulated. Ubiquitin-proteasome pathway has been shown to be important for synaptic plasticity (Hegde, 2010). Down-regulation of multiple proteins belonging to the proteasome pathway indicated possible compromised proteasome degradation, and hence altered synaptic plasticity. As stated earlier, synaptic plasticity is severely affected in cases of Major Depression (Duman and Aghajanian, 2012; Kang et al., 2012; Duman et al., 2016). We also found down-regulation of a number of proteins belonging to the mitochondrial electron-transport chain pathway like ND4, ETFDH, NDUFB3, NDUFA5, etc., in the socially isolated *Wdr13*^-/0^ mice as compared to their wild-type counterparts (**Supplementary Figure S4C**). Down-regulation of proteins belonging to the latter pathway has been reported in rat model of depression (Henningsen et al., 2012). Interestingly, up-regulation of neurofilaments like NEFM, NEFL have been observed in cases of the neuronal damage and exposure to a traumatic episode (Siedler et al., 2014). Increased levels of these proteins in the socially isolated mutants indicated that the social isolation was indeed stressful for these mice and might have been a key factor behind the observed dendritic atrophy. We also found increase in the astroglial marker GFAP in the socially isolated mutant as compared to its wild-type counterpart. GFAP has been shown to be upregulated as a response to stress (Jauregui-Huerta et al., 2010). This also validates our hypothesis that mild social isolation stress had a greater effect on the mutant mice as compared to the wild-type. Taken together, the differential expression of the above mentioned pathways corroborate our stance that the socially isolated *Wdr13*^-/0^ mice may act as a model for Major Depression (MD) in humans.

In contrast to the observed down-regulation of synaptic genes, we had earlier found multiple synaptic genes to be upregulated in the mutant mice (Mitra et al., 2016). Our investigations into the molecular mechanisms behind this contrasting gene expression led us to find that a common transcription factor -GATA1 was up-regulated in socially isolated *Wdr13*^-/0^ mice. GATA1 binds on promoters of synaptic genes like *Syn1, Rab3a, Rab4b*, etc., and negatively regulate them. Increased levels of GATA1 have been reported in post-mortem samples of clinically depressed individuals and the overexpression of GATA1 led to a phenotype of depression-like-symptoms in rat (Kang et al., 2012), which was similar as in the socially isolated *Wdr13*^-/0^ mice. Also it may noted that upon imipramine treatment there was a significant decrease in levels of *Gata1* as compared to socially isolated *Wdr13*^-/0^ mice and comparable to that of wild-type levels. Hence, *Gata1* expression might be directly correlated with depression like phenotype of socially isolated *Wdr13*^-/0^ mice. Thus, taken together up-regulation of GATA1 in the socially isolated mice lacking *Wdr13* gene, might be one of the key factors responsible for decreased expression of the synaptic genes and the observed phenotype. However, further experiments by knocking down *Gata1* in socially isolated *Wdr13*^-/0^ mice would provide direct evidence on role of *Gata1*.

We have also shown that increased levels of WDR13 in turn repressed GATA1 transcription. We believe from the circumstantial evidences as described in this work that WDR13 may have a transcriptional check on *Gata1* expression, particularly in response to stress. WDR13 acts as a co-repressor with nuclear receptors like Estrogen Receptors- ERα/β (Singh and Shalu, personal communication) and represses transcription from Estrogen Receptor Element containing promoter (Mitra et al., 2016). Is it that the regulation of GATA1 is mediated through WDR13’s interacting partners – Estrogen receptors – which are known to negatively affect GATA1 activation and function directly? This needs to be analyzed further through sequential chromatin immunoprecipitation (ChIP) experiments.

In our study we have subjected mice to social isolation stress for a period of 3 weeks which did not elicit symptoms of major-depression like phenotype in wild-type mice. Earlier studies in which wild-type mice have been subjected to similar time period of social isolation have shown variable results depending upon the strain, behavioral paradigms tested; but none gave any drastic major-depression like phenotype (Rodgers and Cole, 1993; Coudereau et al., 1997; Liu et al., 2012; Slattery and Cryan, 2014; Soga et al., 2015). Correspondingly, GATA1 levels were not up-regulated in wild-type mice. However, in Major Depression and in rodent model of depression, it is known that GATA1 gets upregulated (Kang et al., 2012). This implied that the presence of WDR13 alone is not sufficient to check up-regulation of GATA1 in wild-type when subjected to chronic stress and eventually GATA1 does get upregulated. Hence it’s critical to know the factors that regulate *Gata1* transcription under stress and whether accumulation of these factors upon chronic stress is critical for GATA1 upregulation. It is also worthwhile to note that *Wdr13* promoter region has multiple putative GATA1 binding sites (Suresh et al., 2005). This raises the possibility of existence of a feedback loop and the same needs to be investigated.

Finally, our observations from this work – WDR13 is responsive to stress and the absence of it predisposes mice to symptoms of depression, resonates with the findings from our other experiments. The various phenotypes seen in the *Wdr13* deficient mice were further accentuated by insults challenging the homeostasis; where insults can be induced by high fat diet (Singh et al., 2012, 2015) or administration of toxins (Singh et al., 2017; Mishra et al., personal communication) or behavioral insults as in this study. We believe that WDR13- an adaptor molecule that modulates transcription of multiple genes through its interacting partners, is involved in homeostasis and stress response.

## Conclusion

The present study highlights the importance of this gene in enduring stress and delaying the onset of depression. Depression is a major cause of morbidity worldwide in humans (WHO). Despite multiple genome-wide association studies we do not yet know the genetic factors that might predispose an individual to depression (Lohoff, 2010; Wray et al., 2012; Major Depressive Disorder Working Group of the Psychiatric GWAS Consortium Ripke et al., 2013). Exhibiting endophenotypes of depression that are responsive to anti-depressants, *Wdr13*^-/0^ mice represent a very intriguing model system to study Major Depression- like phenotype and may be useful in pharmacological studies. Considering that this gene is highly conserved, *Wdr13* emerges as a candidate to be considered for genetic screening in patients with Major Depressive Disorder. This present study in conjunction to our previous findings (Mitra et al., 2016), would add to our limited knowledge about the function of this gene and hopefully aid in prognosis of patients bearing mutations in this gene.

## Author Contributions

SM designed and executed the experiments and drafted the manuscript. GSK and SM executed the proteomic experiments and analyzed the data. BJL carried out animal breeding, crossing, maintenance, and genotyping. ST helped in providing scientific and technical inputs regarding designing and execution of proteomic experiments. SK gave suggestions, helped in interpreting the data, and drafting the manuscript.

## Conflict of Interest Statement

The present work has been carried out in entirety in CSIR-CCMB. GSK was involved in the project during his tenure at CSIR-CCMB but is currently an employee of Vimta Labs. Vimta Labs has no contribution in any capacity towards the project. The remaining authors declare no competing financial conflict of interest.

## Supplementary Material

FIGURE S1 Schematic of the experiment.

FIGURE S2 Sucrose preference test in non-stressed wild-type and *Wdr13*^-/0^ mice (Cohort#4) did not reveal any significant differences [*n* = 10; Two way ANOVA; effect of genotype *F*(1,76) = 0.025; *p* > 0.05 | effect of days *F*(3,76) = 6.421; *p* < 0.05 | interaction *F*(3,76) = 0.098; *p* > 0.05]. ^∗^ denotes *p* < 0.05 and ^∗∗^ denotes *p* < 0.005.

FIGURE S3 Replicates of social isolation experiment followed by behavioral analysis. **(A)** There was no significant difference between wild-type and mutant mice in total distance covered in the arena of Open Field Test (OFT) (*n* = 6; *t*-test; *p* > 0.05). **(B)** Socially isolated *Wdr13*^-/0^ mice (Cohort#2,3) showed decreased exploration in the central area of OFT as compared to its wild-type as well as non-stressed counterparts (*n* = 6; independent *t*-test; *p* < 0.05). **(C)** Socially isolated *Wdr13*^-/0^ mice showed increased immobility in Forced Swim Test (FST) as compared to its wild-type as well as non-stressed counterparts (Cohort#2,3; *n* = 6; independent *t*-test; *p* < 0.05). **(D)** There was a significant increase in time immobile in FST for socially isolated mutant mice in an independent cohort of mice (Cohort#5; *n* = 6; *t*-test; *p* < 0.05). **(E)** Time spent in closed arm and open arms of Elevated Plus Maze test by non-stressed mice (Cohort#4; *n* = 10). **(F)** There was a significant increase in total time spent in closed arms by the mutant mice as compared to its wild-type counterparts (Cohort#5; *n* = 6; *t*-test; *p* < 0.05). *Wdr13^+/0^*, wild-type; *Wdr13*^-/0^, *Wdr13* knockout mice; SI, Social Isolation. ^∗^ denotes *p* < 0.05 and ^∗∗^ denotes *p* < 0.005.

FIGURE S4 Effect of age and genetic background on FST after 3 weeks social isolation in *Wdr13*^-/0^ and wild-type mice **(A)** 4 months (Cohort#6; *n* = 8) and **(B)** 7 months (Cohort#7; *n* = 6) old *Wdr13*^-/0^ mice spent significantly (*t*-test; *p* < 0.05) more time immobile in FST than the wild-type after social isolation. **(C)** 2 months old *Wdr13*^-/0^ mice in C57Bl/6J background (*n* = 6–8) also showed similar phenotype- they remained immobile for significantly (*t*-test; *p* < 0.05) greater time as compared to wild-type mice after social isolation stress. *Wdr13^+/0^*, wild-type; *Wdr13*^-/0^, *Wdr13* knockout mice; SI, Social Isolation. ^∗^ denotes *p* < 0.05 and ^∗∗^ denotes *p* < 0.005.

FIGURE S5 Comparative analysis of proteins from pre-frontal cortex (PFC) labeled and quantified using 8 plex iTRAQ reagent. **(A)** Heat map comparing different groups (1% FDR and at-least one unique peptide) I. Socially isolated wild-type (*Wdr13*^+/0^) to non-stressed wild-type II. Socially isolated knockout (*Wdr13*^-/0^) to socially isolated wild-type III. Non-stressed knockout to non-stressed wild-type IV. Socially isolated knockout to non-stressed knockout. **(B)** Comparison of few proteins belonging to regulation of synaptic plasticity amongst different groups. **(C)** Comparison of few proteins belonging to oxidation-reduction pathway amongst different groups *Wdr13^+/0^*: wild-type; *Wdr13*^-/0^: *Wdr13* knockout mice; SI: Social Isolation.

FIGURE S6 **(A)** MS/MS spectra and **(B)** iTRAQ label spectra for CAMK2A, one of the differentially expressed proteins in *Wdr13*^-/0^ mice as compared to wild-type before and after social isolation.

FIGURE S7 **(A)** MS/MS spectra and **(B)** iTRAQ label spectra for SYN1A, one of the differentially expressed proteins in *Wdr13*^-/0^ mice as compared to wild-type before and after social isolation.

FIGURE S8 **(A)** Transcript analysis from pre-frontal cortex (PFC) after social isolation in *Wdr13*^-/0^ mice as compared to the wild-type mice (*n* = 5). Synaptic genes *Rab3a* and *Nrxn2* showed downregulation whereas transcription factor *Gata1* was upregulated. **(B)** Analysis using western blotting for CAMK2A from hippocampus of different groups reveal upregulation in non-stressed mutant (*Wdr13*^-/0^) and downregulation (individual Mann–Whitney test; *p* < 0.05) in socially isolated mutant mice as compared to other counterparts. Data represented as ± SD. **(C)** Transcript analysis of genes from nucleus accumbens (NA) of *Wdr13*^-/0^ and wild-type mice after social isolation (*n* = 5). **(D)** Analysis of GATA1 and NRXN2 in the *Wdr13*^-/0^ mice as compared to wild-type after social isolation. Data represented as ± SD. **(E)** Expression analysis of *Gata1* transcript from hippocampus of *Wdr13*^-/0^ and wild-type mice (*n* = 4). Data represented as ± SD. **(F)** Effect of 3 weeks social isolation on *Gata1* expression in wild-type mice showed no significant difference (Mann–Whitney; *p* > 0.05; *n* = 4). Data represented as ± SD. *Wdr13^+/0^*, wild-type; *Wdr13*^-/0^, *Wdr13* knockout mice; SI, Social Isolation. ^∗^ denotes *p* < 0.05 and ^∗∗^ denotes *p* < 0.005.

FIGURE S9 Effect of anti-depressant imipramine treatment on socially isolated *Wdr13*^-/0^ and wild-type mice measured by FST. **(A)** Schematic of the imipramine treatment (Cohort#8,9,10). **(B)** Upon imipramine treatment there was a significant reduction in immobility in *Wdr13*^-/0^ mice (*n* = 7 each; effect of drug on genotype Two way ANOVA, *F*(2,36) = 6.353; *p* < 0.005). **(C)** There was a decrease in transcript level of *Gata1* (independent *t*-test; *p* < 0.05) upon imipramine treatment as compared to socially isolated *Wdr13*^-/0^ mice. *Wdr13^+/0^*, wild-type; *Wdr13*^-/0^, *Wdr13* knockout mice; SI, Social Isolation. ^∗^ denotes *p* < 0.05 and ^∗∗^ denotes *p* < 0.005.

TABLE S1 Proteomics (8 plex iTRAQ) of pre-frontal cortex (PFC) from *Wdr13*^+/0^ and *Wdr13*^-/0^ mice before and after 3 weeks social isolation (at-least one unique peptide).

TABLE S2 Proteomics (8 plex iTRAQ) of PFC from *Wdr13*^+/0^ and *Wdr13*^-/0^ mice before and after 3 weeks social isolation (at-least two unique peptides).

TABLE S3 Delineation of downregulated proteins (at-least two unique peptides) from PFC of *Wdr13*^-/0^ mice after social isolation into Biological Processes using String analysis.

TABLE S4 Delineation of downregulated proteins from PFC (at-least two unique peptides) of *Wdr13*^-/0^ mice after social isolation into KEGG Pathways using String analysis.

TABLE S5 Proteomics (4 plex iTRAQ) of hippocampus from socially isolated *Wdr13*^+/0^ and *Wdr13*^-/0^ mice.
