# Supplementary figures and images for: Absence of Wdr13 Gene Predisposes Mice to Mild Social Isolation – Chronic Stress, Leading to Depression-Like Phenotype Associated With Differential Expression of Synaptic Proteins
Source: Front Mol Neurosci. 2018 Apr 25;11:133. doi: 10.3389/fnmol.2018.00133 (PMC5930177; doi:10.3389/fnmol.2018.00133)

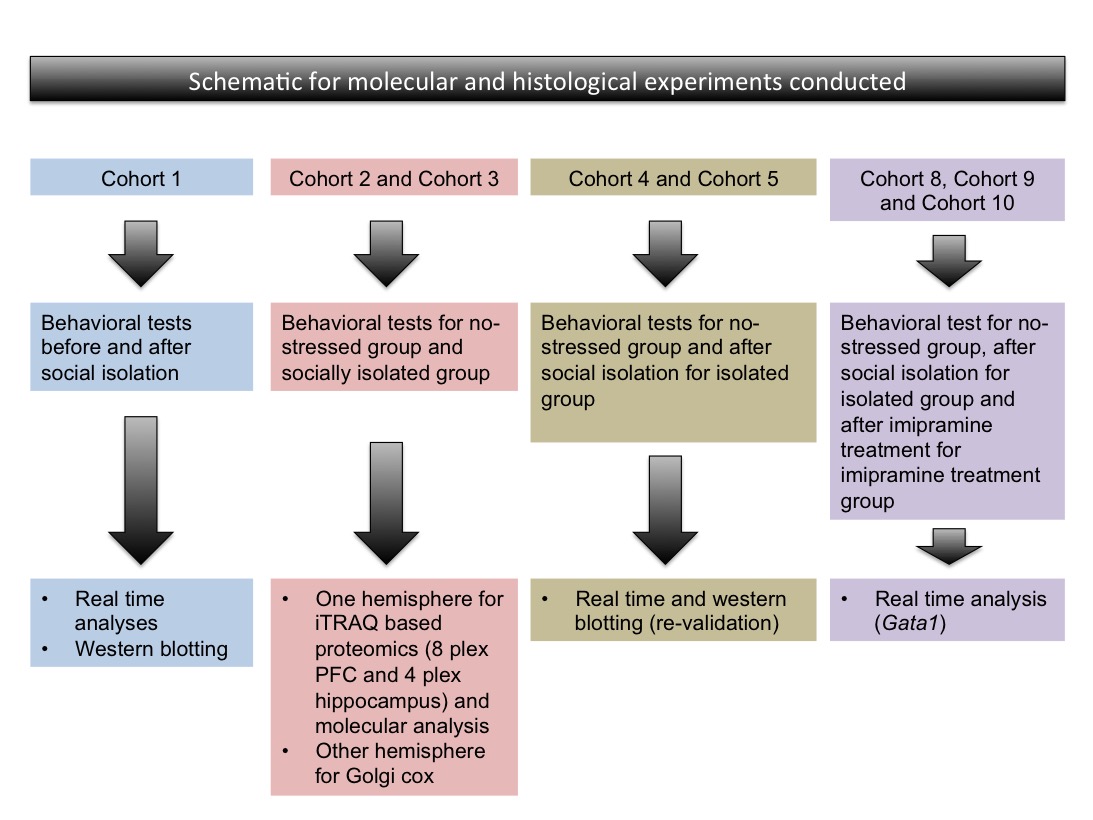

Supplement: FIGURE S1 — Schematic of the experiment. [file Image_1.JPEG]

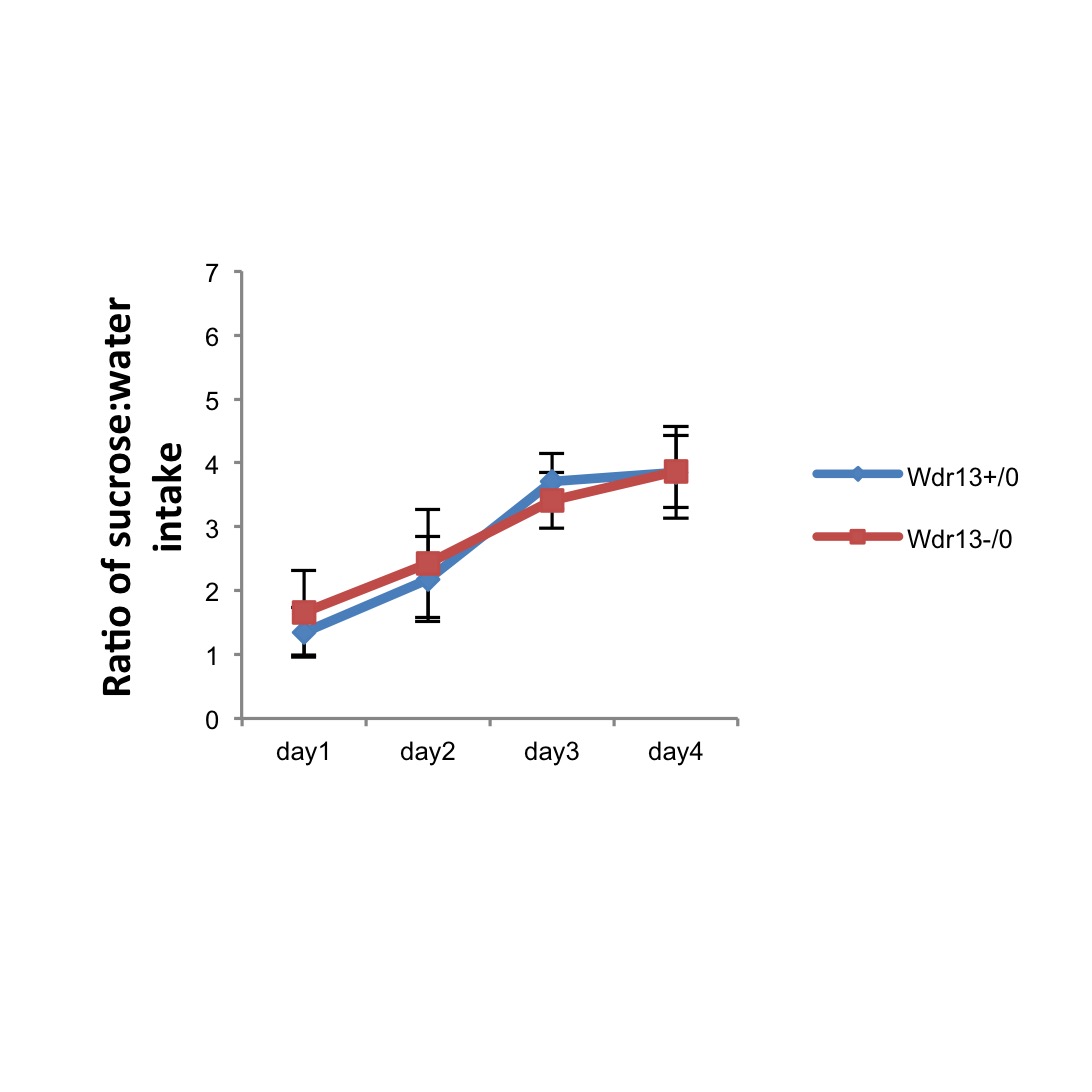

Supplement: FIGURE S2 — Sucrose preference test in non-stressed wild-type and Wdr13-/0 mice (Cohort#4) did not reveal any significant differences [n = 10; Two way ANOVA; effect of genotype F(1,76) = 0.025; p > 0.05 | effect of days F(3,76) = 6.421; p < 0.05 | interaction F(3,76) = 0.098; p > 0.05]. ∗ denotes p < 0.05 and ∗∗ denotes p < 0.005. [file Image_2.JPEG]

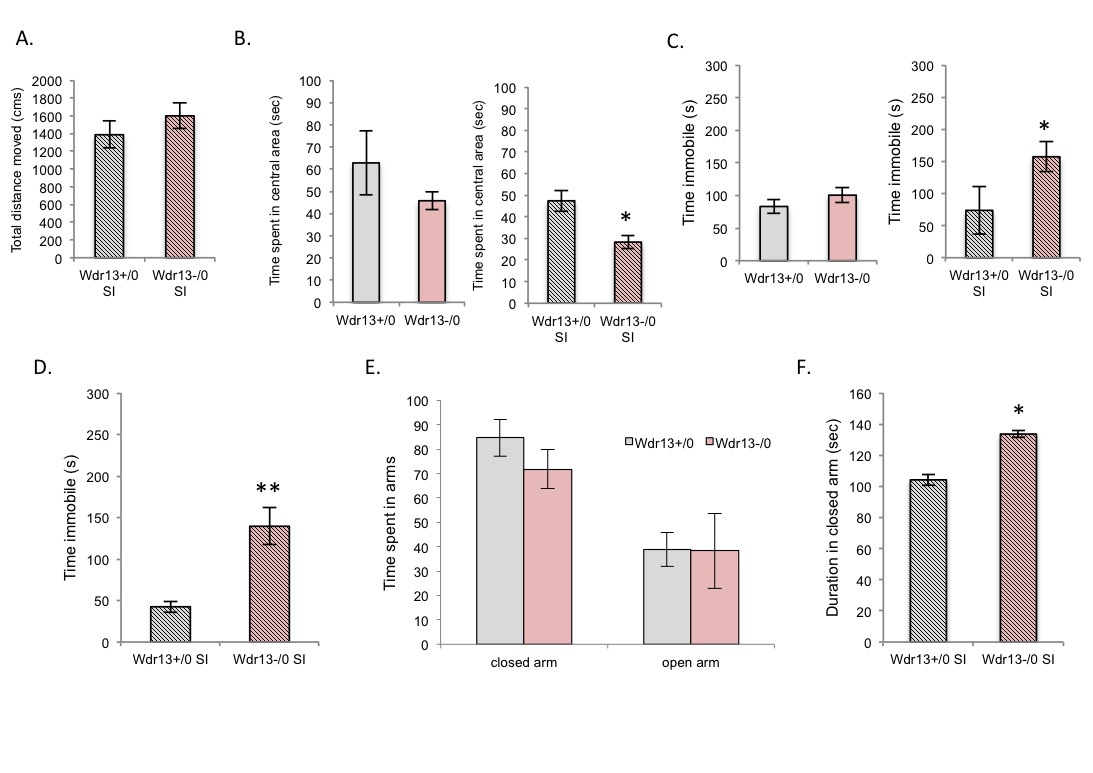

Supplement: FIGURE S3 — Replicates of social isolation experiment followed by behavioral analysis. (A) There was no significant difference between wild-type and mutant mice in total distance covered in the arena of Open Field Test (OFT) (n = 6; t-test; p > 0.05). (B) Socially isolated Wdr13-/0 mice (Cohort#2,3) showed decreased exploration in the central area of OFT as compared to its wild-type as well as non-stressed counterparts (n = 6; independent t-test; p < 0.05). (C) Socially isolated Wdr13-/0 mice showed increased immobility in Forced Swim Test (FST) as compared to its wild-type as well as non-stressed counterparts (Cohort#2,3; n = 6; independent t-test; p < 0.05). (D) There was a significant increase in time immobile in FST for socially isolated mutant mice in an independent cohort of mice (Cohort#5; n = 6; t-test; p < 0.05). (E) Time spent in closed arm and open arms of Elevated Plus Maze test by non-stressed mice (Cohort#4; n = 10). (F) There was a significant increase in total time spent in closed arms by the mutant mice as compared to its wild-type counterparts (Cohort#5; n = 6; t-test; p < 0.05). Wdr13+/0, wild-type; Wdr13-/0, Wdr13 knockout mice; SI, Social Isolation. ∗ denotes p < 0.05 and ∗∗ denotes p < 0.005. [file Image_3.JPEG]

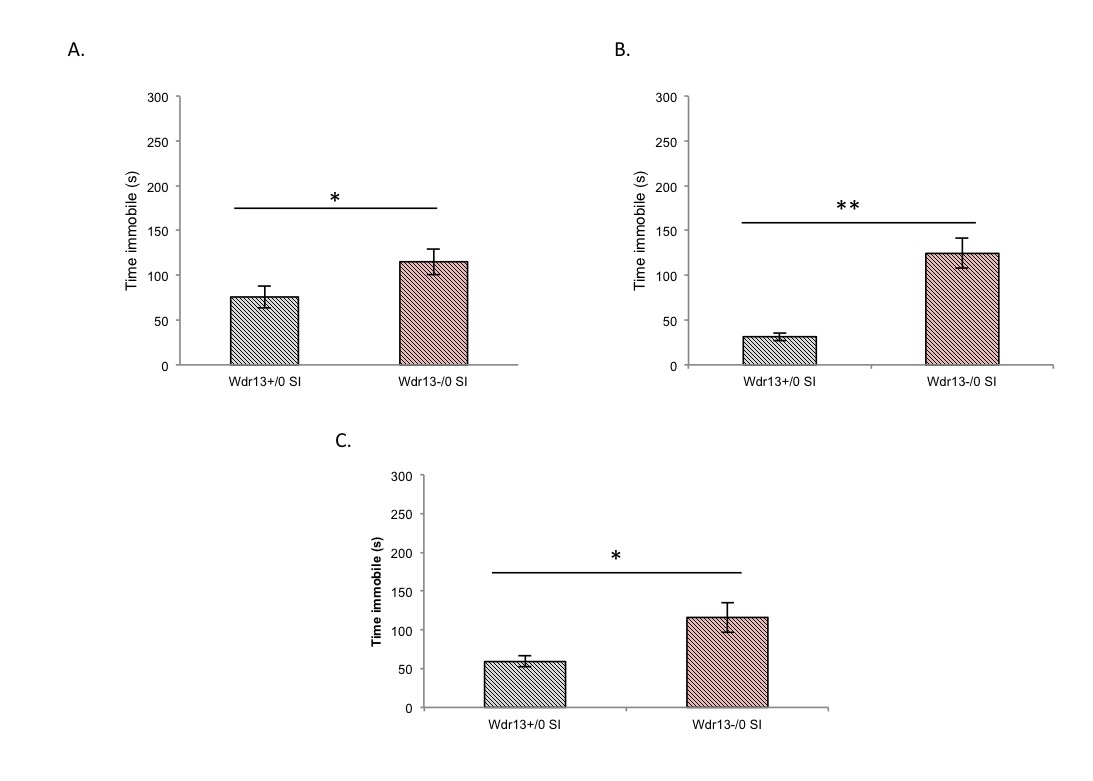

Supplement: FIGURE S4 — Effect of age and genetic background on FST after 3 weeks social isolation in Wdr13-/0 and wild-type mice (A) 4 months (Cohort#6; n = 8) and (B) 7 months (Cohort#7; n = 6) old Wdr13-/0 mice spent significantly (t-test; p < 0.05) more time immobile in FST than the wild-type after social isolation. (C) 2 months old Wdr13-/0 mice in C57Bl/6J background (n = 6–8) also showed similar phenotype- they remained immobile for significantly (t-test; p < 0.05) greater time as compared to wild-type mice after social isolation stress. Wdr13+/0, wild-type; Wdr13-/0, Wdr13 knockout mice; SI, Social Isolation. ∗ denotes p < 0.05 and ∗∗ denotes p < 0.005. [file Image_4.JPEG]

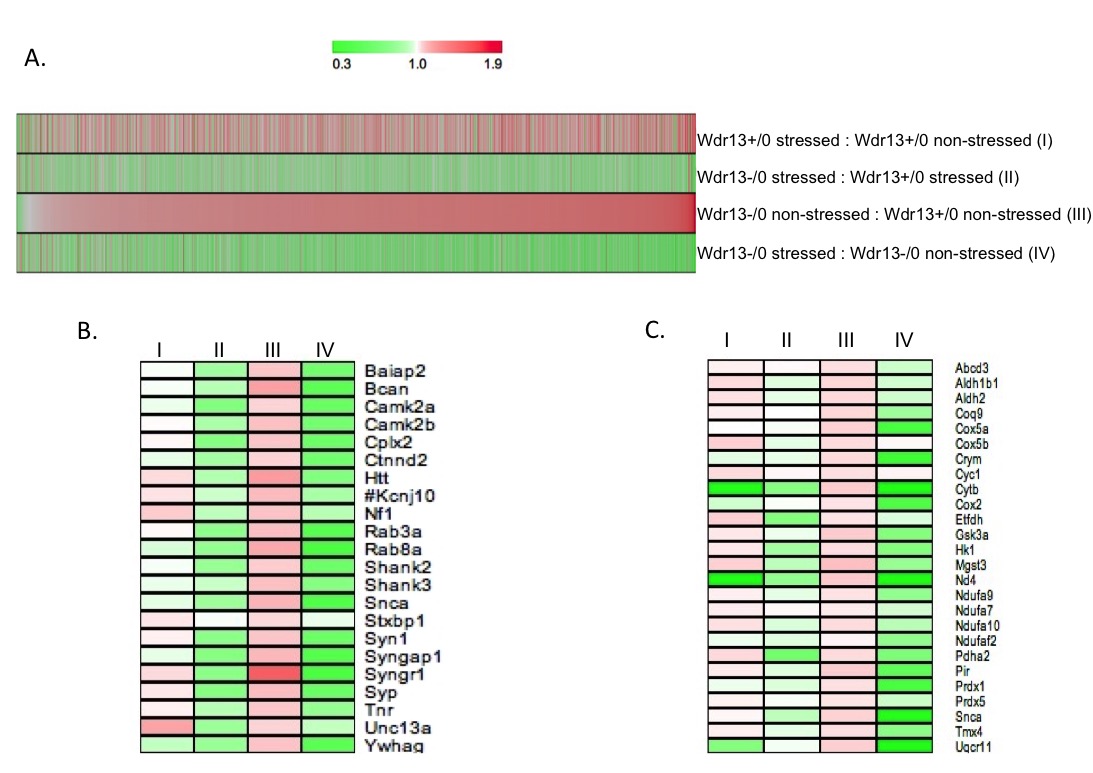

Supplement: FIGURE S5 — Comparative analysis of proteins from pre-frontal cortex (PFC) labeled and quantified using 8 plex iTRAQ reagent. (A) Heat map comparing different groups (1% FDR and at-least one unique peptide) I. Socially isolated wild-type (Wdr13+/0) to non-stressed wild-type II. Socially isolated knockout (Wdr13-/0) to socially isolated wild-type III. Non-stressed knockout to non-stressed wild-type IV. Socially isolated knockout to non-stressed knockout. (B) Comparison of few proteins belonging to regulation of synaptic plasticity amongst different groups. (C) Comparison of few proteins belonging to oxidation-reduction pathway amongst different groups Wdr13+/0: wild-type; Wdr13-/0: Wdr13 knockout mice; SI: Social Isolation. [file Image_5.JPEG]

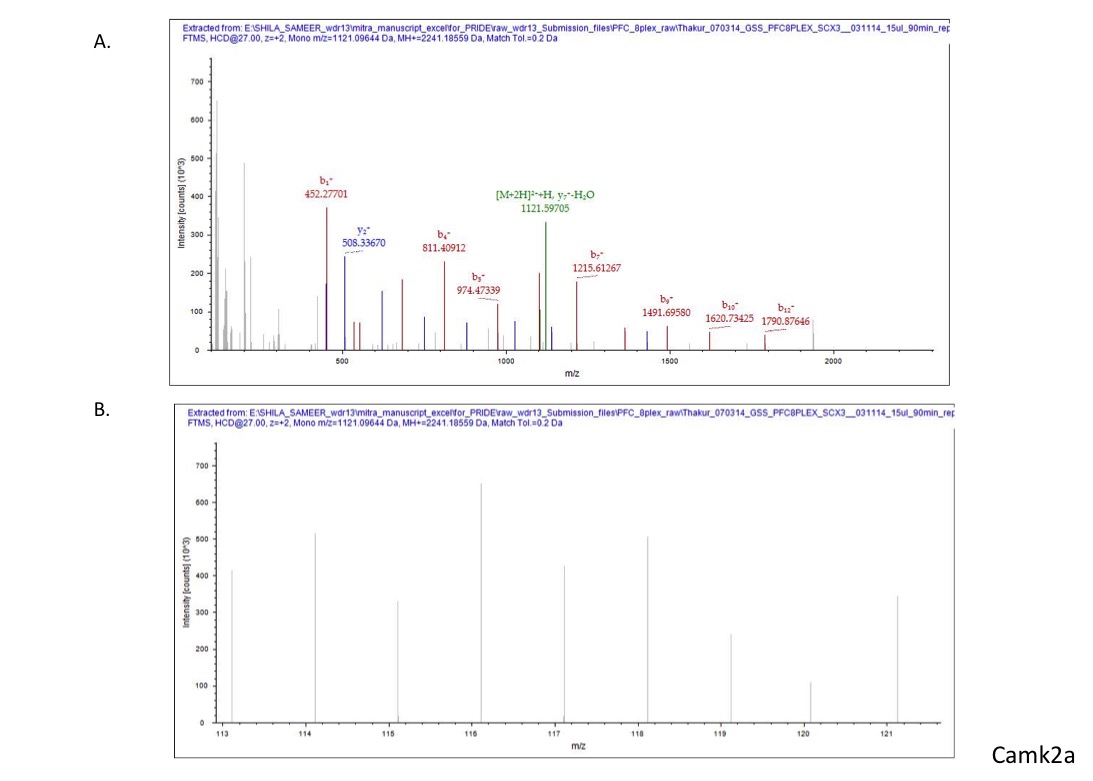

Supplement: FIGURE S6 — (A) MS/MS spectra and (B) iTRAQ label spectra for CAMK2A, one of the differentially expressed proteins in Wdr13-/0 mice as compared to wild-type before and after social isolation. [file Image_6.JPEG]

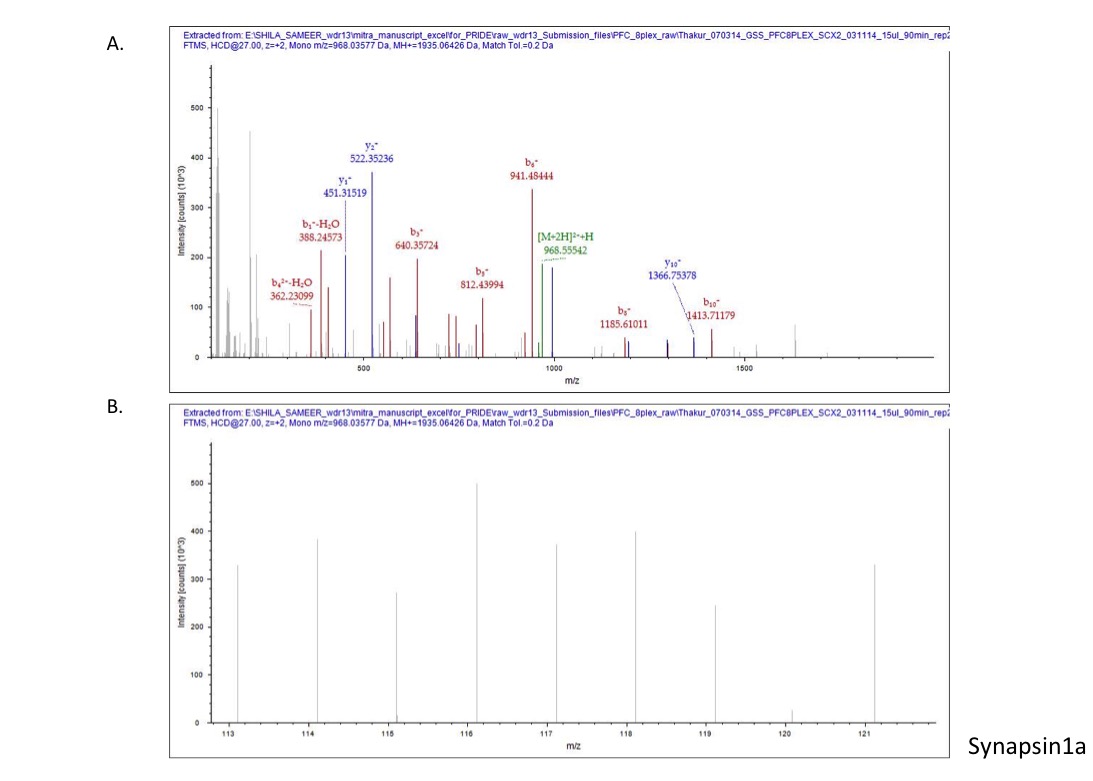

Supplement: FIGURE S7 — (A) MS/MS spectra and (B) iTRAQ label spectra for SYN1A, one of the differentially expressed proteins in Wdr13-/0 mice as compared to wild-type before and after social isolation. [file Image_7.JPEG]

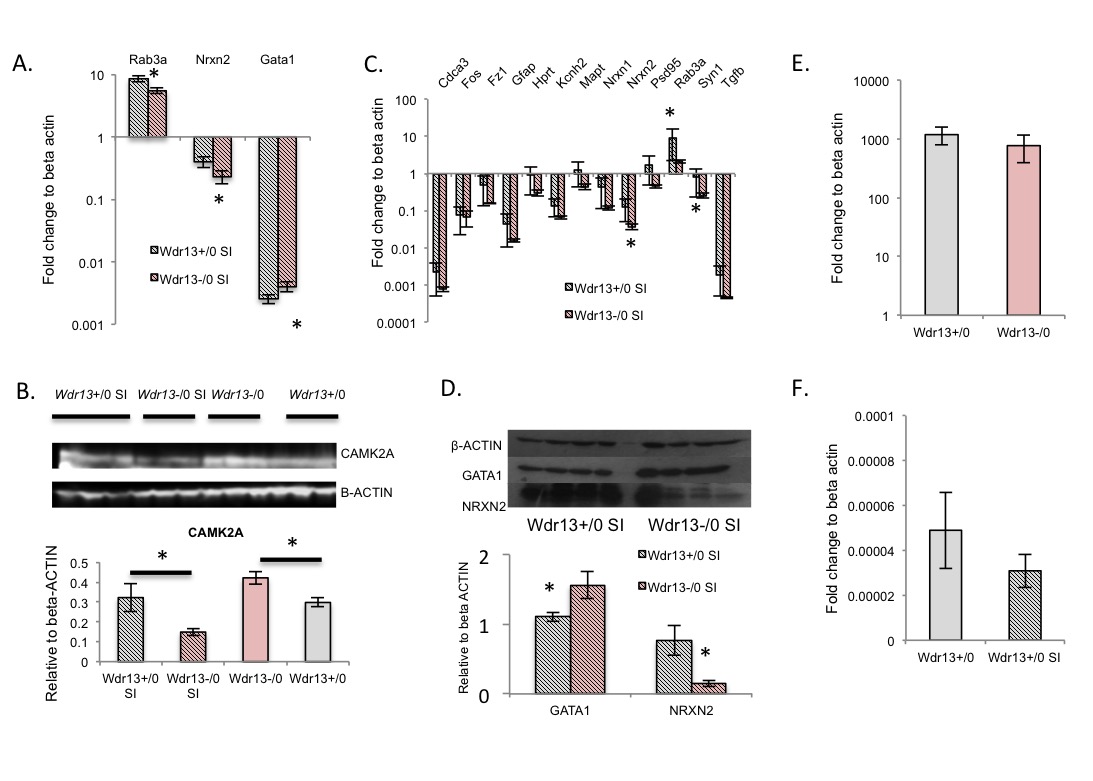

Supplement: FIGURE S8 — (A) Transcript analysis from pre-frontal cortex (PFC) after social isolation in Wdr13-/0 mice as compared to the wild-type mice (n = 5). Synaptic genes Rab3a and Nrxn2 showed downregulation whereas transcription factor Gata1 was upregulated. (B) Analysis using western blotting for CAMK2A from hippocampus of different groups reveal upregulation in non-stressed mutant (Wdr13-/0) and downregulation (individual Mann–Whitney test; p < 0.05) in socially isolated mutant mice as compared to other counterparts. Data represented as ± SD. (C) Transcript analysis of genes from nucleus accumbens (NA) of Wdr13-/0 and wild-type mice after social isolation (n = 5). (D) Analysis of GATA1 and NRXN2 in the Wdr13-/0 mice as compared to wild-type after social isolation. Data represented as ± SD. (E) Expression analysis of Gata1 transcript from hippocampus of Wdr13-/0 and wild-type mice (n = 4). Data represented as ± SD. (F) Effect of 3 weeks social isolation on Gata1 expression in wild-type mice showed no significant difference (Mann–Whitney; p > 0.05; n = 4). Data represented as ± SD. Wdr13+/0, wild-type; Wdr13-/0, Wdr13 knockout mice; SI, Social Isolation. ∗ denotes p < 0.05 and ∗∗ denotes p < 0.005. [file Image_8.JPEG]

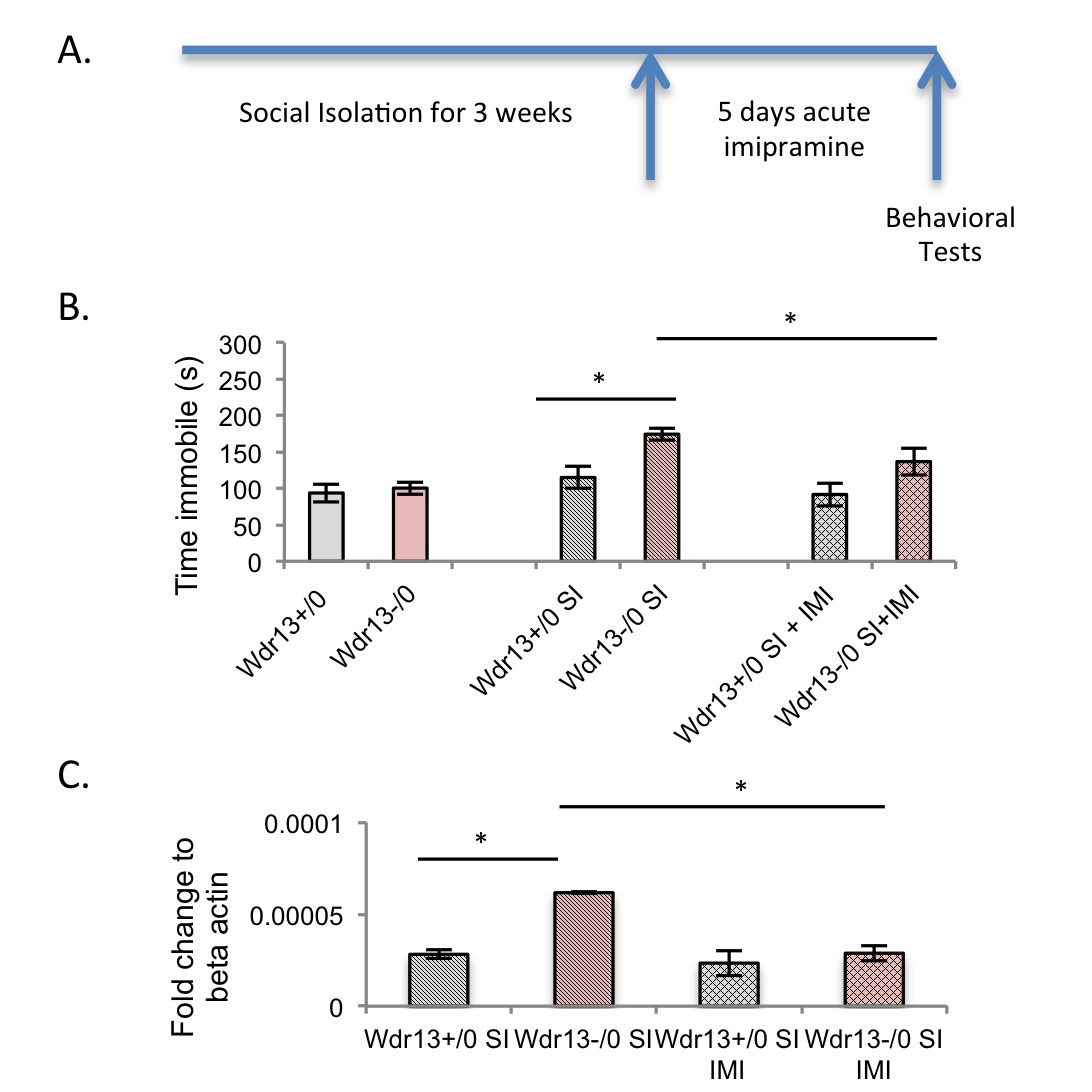

Supplement: FIGURE S9 — Effect of anti-depressant imipramine treatment on socially isolated Wdr13-/0 and wild-type mice measured by FST. (A) Schematic of the imipramine treatment (Cohort#8,9,10). (B) Upon imipramine treatment there was a significant reduction in immobility in Wdr13-/0 mice (n = 7 each; effect of drug on genotype Two way ANOVA, F(2,36) = 6.353; p < 0.005). (C) There was a decrease in transcript level of Gata1 (independent t-test; p < 0.05) upon imipramine treatment as compared to socially isolated Wdr13-/0 mice. Wdr13+/0, wild-type; Wdr13-/0, Wdr13 knockout mice; SI, Social Isolation. ∗ denotes p < 0.05 and ∗∗ denotes p < 0.005. [file Image_9.JPEG]
