# Supplementary material for: Absence of Wdr13 Gene Predisposes Mice to Mild Social Isolation – Chronic Stress, Leading to Depression-Like Phenotype Associated With Differential Expression of Synaptic Proteins
Source: Front Mol Neurosci. 2018 Apr 25;11:133. doi: 10.3389/fnmol.2018.00133 (PMC5930177; doi:10.3389/fnmol.2018.00133)
Supplement: TABLE S1 — Proteomics (8 plex iTRAQ) of pre-frontal cortex (PFC) from Wdr13+/0 and Wdr13-/0 mice before and after 3 weeks social isolation (at-least one unique peptide). [file Table_1.PDF]

SUPPLEMENTARY TABLE 1

Accession	Description	wt stressed/ wt non-stressed	ko stressed/ko non-stressed	113/116	114/116	wt non-stressed/ko non-stressed	ko non-stressed/wt non-stressed	117/116	118/116	119/116	121/116	ko stressed/ko non-stressed
208879441	NP_001129160.1#Ly6h#23934# lymphocyte antigen 6H isoform b precursor [Mus musculus]	**2.065**	0.516	0.365	0.515	0.440	2.272	0.834	0.984	0.421	0.517	0.469
569010783	XP_006528413.1#Gm35586#102639227# PREDICTED: cancer/testis antigen 55-like [Mus musculus]	1.534	0.476	0.295	0.648	0.472	2.120	1.063	0.384	0.273	0.416	0.344
755509391	XP_011248511.1#Nadk#192185# PREDICTED: NAD kinase isoform X2 [Mus musculus]	1.533	0.399	0.261	0.748	0.504	1.983	1.100	0.447	0.310	0.306	0.308
326319955	NP_001191843.1#Reep2#225362# receptor expression-enhancing protein 2 isoform 2 [Mus musculus]	1.147	1.022	0.487	0.633	0.560	1.786	0.517	0.767	0.612	0.701	0.657
498752597	NP_001265090.1#Hbb-b1#15129# hemoglobin subunit beta-1 [Mus musculus]	1.356	0.808	0.512	0.649	0.580	1.723	0.734	0.848	0.548	0.722	0.635
555943687	NP_001273182.1#Nadk2#68646# NAD kinase 2, mitochondrial isoform 3 [Mus musculus]	1.220	0.707	0.526	0.668	0.597	1.675	0.702	0.755	0.459	0.571	0.515
8392847	NP_058556.1#Actr1a#54130# alpha-centractin [Mus musculus]	1.250	1.104	0.493	0.718	0.606	1.651	0.631	0.883	0.775	0.896	0.836
67846099	NP_001020025.1#Timm9#30056# mitochondrial import inner membrane translocase subunit Tim9 [Mus musculus]	**2.206**	0.458	0.557	0.676	0.616	1.622	1.273	1.447	0.578	0.667	0.623
68533251	NP_084384.1#Tmem33#67878# transmembrane protein 33 isoform 2 [Mus musculus]	1.129	0.522	0.374	0.875	0.625	1.601	0.995	0.407	0.312	0.424	0.368
6678193	NP_033329.1#Syngr1#20972# synaptogyrin-1 isoform 1b [Mus musculus]	1.204	0.807	0.522	0.733	0.627	1.594	0.638	0.873	0.487	0.732	0.609
269914154	NP_808385.2#4732456N10Rik#239673# uncharacterized protein LOC239673 [Mus musculus]	0.813	1.483	0.572	0.691	0.632	1.583	0.579	0.448	0.662	0.862	0.762
124286805	NP_031598.2#C1qa#12259# complement C1q subcomponent subunit A precursor [Mus musculus]	1.060	0.820	0.588	0.700	0.644	1.553	0.624	0.741	0.456	0.663	0.560
225543409	NP_079958.3#Pspc1#66645# paraspeckle component 1 [Mus musculus]	1.832	0.602	0.582	0.709	0.645	1.550	1.102	1.262	0.637	0.786	0.712
19526794	NP_598416.1#Cd81#12520# CD81 antigen [Mus musculus]	1.395	0.727	0.490	0.808	0.649	1.541	0.947	0.973	0.578	0.792	0.685
17647499	NP_058652.1#Hbb-b2#15130# hemoglobin subunit beta-2 [Mus musculus]	1.207	0.868	0.573	0.726	0.649	1.541	0.715	0.845	0.566	0.725	0.646
568973615	XP_006533226.1#Uqcrq#22272# PREDICTED: cytochrome b-c1 complex subunit 8 isoform X1 [Mus musculus]	1.576	1.056	0.537	0.763	0.650	1.539	1.005	1.043	0.853	1.311	1.082
85701680	NP_001028349.1#Krt76#77055# keratin, type II cytoskeletal 2 oral [Mus musculus]	0.703	1.535	0.593	0.723	0.658	1.519	0.537	0.388	0.605	0.815	0.710
163310765	NP_033784.2#Alb#11657# serum albumin precursor [Mus musculus]	1.219	0.869	0.579	0.742	0.660	1.515	0.770	0.910	0.595	0.786	0.691
108	K1C10_HUMAN	0.894	1.515	0.599	0.729	0.664	1.506	0.539	0.552	0.709	0.924	0.816
568942466	XP_006506472.1#Nagk#56174# PREDICTED: N-acetyl-D-glucosamine kinase isoform X3 [Mus musculus]	1.284	0.898	0.656	0.685	0.670	1.492	0.791	0.931	0.646	0.900	0.773
197333685	NP_766543.1#Vcan#13003# versican core protein isoform 5 precursor [Mus musculus]	1.655	1.028	0.654	0.697	0.676	1.480	0.975	1.261	0.985	1.314	1.150
568953565	XP_006508978.1#Kbtbd11#74901# PREDICTED: kelch repeat and BTB domain-containing protein 11 isoform X1 [Mus musculus]	1.259	0.819	0.590	0.762	0.676	1.479	0.727	0.975	0.591	0.804	0.697
226423873	NP_001139781.1#Dbnl#13169# drebrin-like protein isoform 3 [Mus musculus]	1.176	0.886	0.535	0.819	0.677	1.477	0.741	0.851	0.564	0.847	0.705
34328075	NP_081084.1#Fitm1#68680# fat storage-inducing transmembrane protein 1 [Mus musculus]	1.097	0.506	0.449	0.908	0.678	1.474	0.937	0.551	0.357	0.396	0.377
13385010	NP_079845.1#Mgst3#66447# microsomal glutathione S-transferase 3 [Mus musculus]	1.335	0.858	0.707	0.663	0.685	1.460	0.884	1.031	0.829	0.796	0.812
568890126	XP_006543864.1#Cgn#70737# PREDICTED: cingulin isoform X1 [Mus musculus]	1.711	0.684	0.634	0.738	0.686	1.457	1.104	1.244	0.658	0.948	0.803
62990174	NP_898964.2#Elfn2#207393# protein phosphatase 1 regulatory subunit 29 precursor [Mus musculus]	1.260	0.800	0.655	0.718	0.686	1.457	0.780	0.949	0.642	0.743	0.692
6679359	NP_032888.1#Pkia#18767# cAMP-dependent protein kinase inhibitor alpha [Mus musculus]	1.252	0.954	0.633	0.747	0.690	1.449	0.792	0.936	0.684	0.965	0.824
6679227	NP_032817.1#Pcp4#18546# Purkinje cell protein 4 [Mus musculus]	1.049	0.833	0.564	0.817	0.691	1.448	0.709	0.761	0.588	0.750	0.669
85677491	NP_001034262.1#Dok6#623279# docking protein 6 [Mus musculus]	1.117	0.871	0.580	0.806	0.693	1.444	0.839	0.709	0.552	0.796	0.674
31980922	NP_061266.2#Eef1b2#55949# elongation factor 1-beta [Mus musculus]	1.099	0.949	0.574	0.811	0.693	1.443	0.689	0.834	0.640	0.805	0.722
755491057	XP_011236578.1#LOC102642577#102642577# PREDICTED: serine protease inhibitor A3K-like [Mus musculus]	1.078	0.991	0.645	0.741	0.693	1.442	0.702	0.846	0.667	0.855	0.761
755569271	XP_011249834.1#Mat2a#232087# PREDICTED: S-adenosylmethionine synthase isoform type-2 isoform X1 [Mus musculus]	1.181	0.661	0.501	0.886	0.693	1.442	0.938	0.668	0.504	0.596	0.550
568982529	XP_006517001.1#Tmem14c#66154# PREDICTED: transmembrane protein 14C isoform X2 [Mus musculus]	1.235	0.926	0.649	0.739	0.694	1.441	0.744	0.971	0.648	0.939	0.793
564112202	NP_001032889.3#Cd209b#69165# CD209 antigen-like protein B isoform b [Mus musculus]	1.270	0.999	0.630	0.760	0.695	1.439	0.792	0.973	0.744	1.018	0.881
6753144	NP_033859.1#Atp6v0c#11984# V-type proton ATPase 16 kDa proteolipid subunit [Mus musculus]	0.766	0.835	0.649	0.741	0.695	1.439	0.532	0.548	0.402	0.511	0.457
23943838	NP_694790.1#Slc25a1#13358# tricarboxylate transport protein, mitochondrial [Mus musculus]	1.066	0.876	0.591	0.806	0.698	1.432	0.674	0.815	0.566	0.738	0.652
568974605	XP_006533694.1#Rap1gap2#380711# PREDICTED: rap1 GTPase-activating protein 2 isoform X10 [Mus musculus]	1.097	0.769	0.678	0.734	0.706	1.417	0.656	0.893	0.538	0.652	0.595
12963653	NP_075860.1#Pin1#23988# peptidyl-prolyl cis-trans isomerase NIMA-interacting 1 [Mus musculus]	1.435	0.788	0.603	0.812	0.708	1.413	0.882	1.149	0.663	0.938	0.800
62000629	NP_084137.1#Cnrip1#380686# CB1 cannabinoid receptor-interacting protein 1 [Mus musculus]	1.087	0.874	0.624	0.797	0.711	1.407	0.727	0.818	0.568	0.782	0.675
15011884	NP_076288.1#Mgea5#76055# protein O-GlcNAcase [Mus musculus]	1.172	0.909	0.683	0.742	0.712	1.404	0.743	0.928	0.747	0.772	0.759
568960050	XP_006510660.1#4931406C07Rik#70984# PREDICTED: ester hydrolase C11orf54 homolog isoform X2 [Mus musculus]	1.532	0.679	0.603	0.825	0.714	1.401	1.095	1.092	0.716	0.770	0.743
568933786	XP_006503807.1#Htt#15194# PREDICTED: huntingtin isoform X1 [Mus musculus]	1.197	0.910	0.654	0.779	0.716	1.396	0.836	0.879	0.628	0.932	0.780
10946986	NP_067523.1#Arpp19#59046# cAMP-regulated phosphoprotein 19 isoform 1 [Mus musculus]	1.356	0.869	0.610	0.824	0.717	1.394	0.954	0.990	0.782	0.906	0.844
8567402	NP_038691.1#Srsf3#20383# serine/arginine-rich splicing factor 3 [Mus musculus]	1.164	0.925	0.702	0.734	0.718	1.392	0.767	0.905	0.674	0.873	0.774
226823223	NP_001152823.1#Ube2v2#70620# ubiquitin-conjugating enzyme E2 variant 2 isoform 2 [Mus musculus]	1.101	0.923	0.627	0.811	0.719	1.391	0.751	0.832	0.609	0.852	0.731
9790221	NP_062741.1#Arpc1a#56443# actin-related protein 2/3 complex subunit 1A [Mus musculus]	1.283	0.780	0.671	0.768	0.719	1.390	0.769	1.077	0.658	0.782	0.720
568905274	XP_006495535.1#Lypla1#18777# PREDICTED: acyl-protein thioesterase 1 isoform X2 [Mus musculus]	0.858	0.864	0.650	0.788	0.719	1.390	0.540	0.693	0.468	0.597	0.533
701462854	NP_001289564.1#Phactr1#218194# phosphatase and actin regulator 1 isoform 4 [Mus musculus]	1.013	0.861	0.629	0.811	0.720	1.389	0.668	0.791	0.530	0.726	0.628
22129129	NP_667085.1#Olfr900#258874# olfactory receptor 900 [Mus musculus]	1.346	0.869	0.650	0.791	0.720	1.388	0.867	1.072	0.737	0.948	0.843
167716846	YP_001686707.1#ND4#5912276# NADH dehydrogenase subunit 4 [Mus musculus musculus]	0.568	0.806	0.651	0.791	0.721	1.387	0.380	0.439	0.307	0.353	0.330
185	PPIA_HUMAN	0.878	0.931	0.654	0.793	0.723	1.383	0.614	0.716	0.541	0.725	0.633
167716849	YP_001686710.1#CYTB#5912277# cytochrome b [Mus musculus musculus]	0.621	0.793	0.598	0.850	0.724	1.381	0.391	0.508	0.312	0.401	0.357
157951643	NP_150371.4#Actn2#11472# alpha-actinin-2 [Mus musculus]	1.022	0.948	0.603	0.848	0.725	1.379	0.667	0.816	0.595	0.824	0.709
34328322	NP_076294.2#Slc38a3#76257# sodium-coupled neutral amino acid transporter 3 [Mus musculus]	1.207	1.145	0.645	0.807	0.726	1.378	0.686	1.065	0.856	1.150	1.003
568949306	XP_006507253.1#Me3#109264# PREDICTED: NADP-dependent malic enzyme, mitochondrial isoform X1 [Mus musculus]	1.189	0.898	0.639	0.815	0.727	1.376	0.742	0.916	0.576	0.808	0.692
568921663	XP_006500999.1#Bcan#12032# PREDICTED: brevican core protein isoform X1 [Mus musculus]	1.077	0.920	0.632	0.823	0.728	1.374	0.703	0.831	0.590	0.741	0.666
568900009	XP_006543175.1#Gm10257#544973# PREDICTED: histone H3.3-like [Mus musculus]	0.888	0.916	0.704	0.756	0.730	1.370	0.582	0.714	0.501	0.683	0.592
407263560	XP_003945495.1#Gm21540#100862175# PREDICTED: transmembrane emp24 domain-containing protein 2 [Mus musculus]	1.198	0.800	0.582	0.880	0.731	1.369	0.807	0.944	0.581	0.819	0.700
116089322	NP_083058.2#Lonp1#74142# lon protease homolog, mitochondrial precursor [Mus musculus]	0.974	0.884	0.687	0.775	0.731	1.368	0.676	0.747	0.555	0.704	0.629
123	K2C1_HUMAN	0.809	1.238	0.630	0.832	0.731	1.368	0.602	0.598	0.633	0.808	0.720
20330802	NP_598738.1#Trf#22041# serotransferrin precursor [Mus musculus]	1.237	0.845	0.652	0.812	0.732	1.367	0.838	0.971	0.641	0.837	0.739
226437665	NP_081520.1#Ndufa11#69875# NADH dehydrogenase [ubiquinone] 1 alpha subcomplex subunit 11 [Mus musculus]	1.145	0.897	0.669	0.798	0.733	1.363	0.719	0.915	0.641	0.839	0.740
568960893	XP_006510944.1#Scg3#20255# PREDICTED: secretogranin-3 isoform X1 [Mus musculus]	1.069	0.928	0.650	0.818	0.734	1.363	0.652	0.917	0.587	0.869	0.728
13385090	NP_079904.1#Cox6b1#110323# cytochrome c oxidase subunit 6B1 [Mus musculus]	1.147	0.886	0.656	0.812	0.734	1.363	0.759	0.937	0.647	0.865	0.756
568982554	XP_006517013.1#Diras2#68203# PREDICTED: GTP-binding protein Di-Ras2 isoform X1 [Mus musculus]	1.137	0.842	0.648	0.820	0.734	1.363	0.809	0.860	0.586	0.819	0.702
568946346	XP_006540322.1#Gsk3a#606496# PREDICTED: glycogen synthase kinase-3 alpha isoform X2 [Mus musculus]	1.159	0.929	0.640	0.828	0.734	1.362	0.765	0.937	0.665	0.915	0.790
569012254	XP_006529041.1#Pir#69656# PREDICTED: pirin isoform X2 [Mus musculus]	1.120	0.907	0.682	0.787	0.734	1.362	0.762	0.883	0.638	0.854	0.746
83816893	NP_031866.2#Ddx5#13207# probable ATP-dependent RNA helicase DDX5 [Mus musculus]	1.075	0.907	0.682	0.788	0.735	1.360	0.738	0.843	0.621	0.812	0.716
31981100	NP_065625.2#Rps14#20044# 40S ribosomal protein S14 [Mus musculus]	1.009	0.713	0.611	0.860	0.735	1.360	0.885	0.599	0.446	0.612	0.529
17647119	NP_080102.1#Acadsb#66885# short/branched chain specific acyl-CoA dehydrogenase, mitochondrial [Mus musculus]	1.251	0.867	0.743	0.729	0.736	1.358	0.805	1.037	0.725	0.871	0.798
38372905	NP_075615.2#Rab8a#17274# ras-related protein Rab-8A [Mus musculus]	1.002	0.827	0.682	0.792	0.737	1.357	0.644	0.832	0.523	0.698	0.610
755494753	XP_011246411.1#Clasp1#76707# PREDICTED: CLIP-associating protein 1 isoform X30 [Mus musculus]	1.405	0.903	0.671	0.804	0.737	1.356	0.969	1.102	0.807	1.064	0.935
58219050	NP_081226.1#Ociad2#433904# OCIA domain-containing protein 2 [Mus musculus]	1.039	0.915	0.646	0.831	0.739	1.354	0.791	0.924	0.756	0.853	0.804
13385112	NP_079926.1#Uqcr11#66594# cytochrome b-c1 complex subunit 10 [Mus musculus]	0.790	0.941	0.630	0.849	0.740	1.352	0.568	0.623	0.488	0.635	0.562
162417988	NP_602320.2#Gpr37l1#171469# prosaposin receptor GPR37L1 precursor [Mus musculus]	1.033	0.754	0.717	0.762	0.740	1.352	0.666	0.862	0.424	0.729	0.576
755565607	XP_011245948.1#Snx12#55988# PREDICTED: sorting nexin-12 isoform X1 [Mus musculus]	1.148	0.917	0.712	0.767	0.740	1.352	0.804	0.894	0.662	0.895	0.779
506326231	NP_001265185.1#Prnp#19122# major prion protein precursor [Mus musculus]	1.051	0.849	0.654	0.829	0.741	1.349	0.753	0.806	0.577	0.747	0.662
253314534	NP_001156504.1#Synpr#72003# synaptoporin isoform 2 precursor [Mus musculus]	1.290	0.828	0.682	0.803	0.743	1.347	0.873	1.043	0.672	0.914	0.793
31560653	NP_032852.2#Pfkl#18641# ATP-dependent 6-phosphofructokinase, liver type [Mus musculus]	1.151	0.868	0.650	0.836	0.743	1.346	0.791	0.960	0.650	0.824	0.737
21361250	NP_081715.1#Atp6ap2#70495# renin receptor precursor [Mus musculus]	1.188	0.842	0.640	0.846	0.743	1.346	0.820	0.946	0.666	0.821	0.744

13591860	NP_076253.1#Tollip#54473# toll-interacting protein [Mus musculus]	1.127	0.856	0.643	0.845	0.744	1.344	0.778	0.960	0.628	0.877	0.753
568984656	XP_006517804.1#Rab3c#67295# PREDICTED: ras-related protein Rab-3C isoform X2 [Mus musculus]	1.084	0.869	0.639	0.851	0.745	1.342	0.789	0.911	0.577	0.841	0.709
165972305	NP_033321.2#Stxbp1#20910# syntaxin-binding protein 1 isoform b [Mus musculus]	1.137	0.843	0.680	0.811	0.746	1.341	0.844	0.851	0.696	0.734	0.715
257096046	NP_001157875.1#Ptpro#19277# receptor-type tyrosine-protein phosphatase O isoform 4 [Mus musculus]	0.867	0.960	0.785	0.708	0.746	1.340	0.651	0.643	0.558	0.685	0.621
568959944	XP_006510610.1#Chordc1#66917# PREDICTED: cysteine and histidine-rich domain-containing protein 1 isoform X1 [Mus musculus]	1.350	0.803	0.630	0.863	0.746	1.340	0.883	1.135	0.686	0.936	0.811
31980772	NP_038664.2#Ppp1cc#19047# serine/threonine-protein phosphatase PP1-gamma catalytic subunit [Mus musculus]	1.482	0.778	0.665	0.828	0.747	1.339	1.016	1.196	0.782	0.939	0.860
227908861	NP_032470.3#Kif3b#16569# kinesin-like protein KIF3B [Mus musculus]	1.051	0.937	0.695	0.799	0.747	1.339	0.812	0.757	0.640	0.830	0.735
167716839	YP_001686700.1#COX1#5912286# cytochrome c oxidase subunit I [Mus musculus musculus]	0.752	1.022	0.776	0.719	0.747	1.338	0.527	0.607	0.553	0.605	0.579
530677960	NP_001268969.1#Pmm1#29858# phosphomannomutase 1 isoform 2 [Mus musculus]	1.289	0.813	0.656	0.840	0.748	1.337	0.939	0.990	0.637	0.931	0.784
160707958	NP_001104264.1#Anxa7#11750# annexin A7 [Mus musculus]	0.895	0.912	0.686	0.811	0.748	1.337	0.601	0.736	0.500	0.719	0.610
225543378	NP_001139449.1#Cadm2#239857# cell adhesion molecule 2 isoform 2 precursor [Mus musculus]	1.200	0.840	0.671	0.827	0.749	1.335	0.823	0.972	0.664	0.845	0.754
52317148	NP_067467.2#Ap3b2#11775# AP-3 complex subunit beta-2 [Mus musculus]	1.115	0.792	0.662	0.836	0.749	1.335	0.734	0.943	0.572	0.702	0.637
568934257	XP_006504035.1#Ppp2r2c#269643# PREDICTED: serine/threonine-protein phosphatase 2A 55 kDa regulatory subunit B gamma isoform isoform X2 [I	1.123	0.899	0.588	0.912	0.750	1.334	0.796	0.888	0.674	0.841	0.757
27369732	NP_766111.1#Slc18a2#214084# synaptic vesicular amine transporter [Mus musculus]	0.969	1.008	0.564	0.936	0.750	1.333	0.625	0.828	0.725	0.740	0.733
42794773	NP_079580.2#Lcmt1#30949# leucine carboxyl methyltransferase 1 [Mus musculus]	1.016	0.916	0.722	0.779	0.750	1.333	0.669	0.855	0.616	0.781	0.698
13937391	NP_034442.1#Gnb2#14693# guanine nucleotide-binding protein G(I)/G(S)/G(T) subunit beta-2 [Mus musculus]	1.114	0.745	0.667	0.834	0.750	1.333	0.766	0.925	0.611	0.754	0.683
84794552	NP_061346.2#Pebp1#23980# phosphatidylethanolamine-binding protein 1 [Mus musculus]	0.874	0.941	0.676	0.827	0.752	1.331	0.667	0.690	0.603	0.791	0.697
267844806	NP_001161208.1#Chchd6#66098# MICOS complex subunit Mic25 isoform 2 [Mus musculus]	1.396	0.813	0.683	0.820	0.752	1.330	0.960	1.138	0.729	0.976	0.853
11612657	NP_071287.1#Fxyd6#59095# FXYD domain-containing ion transport regulator 6 precursor [Mus musculus]	1.293	0.925	0.644	0.861	0.752	1.329	0.838	1.107	0.725	1.075	0.900
6755448	NP_035472.1#Sec22b#20333# vesicle-trafficking protein SEC22b precursor [Mus musculus]	1.333	0.802	0.731	0.774	0.753	1.329	0.905	1.101	0.761	0.848	0.805
568968337	XP_006514080.1#Atp2b1#67972# PREDICTED: plasma membrane calcium-transporting ATPase 1 isoform X3 [Mus musculus]	1.210	0.851	0.680	0.826	0.753	1.328	0.935	1.110	0.742	1.014	0.878
568978770	XP_006515515.1#Cfl2#12632# PREDICTED: cofilin-2 isoform X1 [Mus musculus]	1.088	0.950	0.703	0.805	0.754	1.326	0.666	0.791	0.580	0.708	0.644
6755080	NP_035232.1#Prkcg#18752# protein kinase C gamma type isoform 1 [Mus musculus]	1.154	0.793	0.655	0.854	0.755	1.325	0.817	1.078	0.615	0.812	0.713
123701966	NP_808383.3#Kctd12#239217# BTB/POZ domain-containing protein KCTD12 [Mus musculus]	0.964	0.818	0.739	0.772	0.756	1.324	0.665	0.791	0.597	0.593	0.595
6755100	NP_035249.1#Pa2g4#18813# proliferation-associated protein 2G4 [Mus musculus]	1.117	0.937	0.681	0.830	0.756	1.324	0.806	0.881	0.686	0.895	0.790
6755997	NP_035846.1#Wfs1#22393# wolframin [Mus musculus]	1.072	0.962	0.741	0.771	0.756	1.323	0.675	0.915	0.723	0.831	0.777
7305443	NP_038749.1#Rpl7a#27176# 60S ribosomal protein L7a [Mus musculus]	1.152	1.034	0.703	0.810	0.756	1.322	0.800	0.878	0.678	1.062	0.870
9845253	NP_063921.1#Hnrnph2#56258# heterogeneous nuclear ribonucleoprotein H2 [Mus musculus]	1.093	0.916	0.705	0.808	0.757	1.322	0.713	0.941	0.608	0.908	0.758
356582311	NP_001239148.1#Rpl31#114641# 60S ribosomal protein L31 [Mus musculus]	1.070	0.900	0.680	0.834	0.757	1.321	0.727	0.930	0.685	0.760	0.722
46592826	NP_997569.1#Elavl2#15569# ELAV-like protein 2 isoform 3 [Mus musculus]	1.259	0.851	0.685	0.833	0.759	1.318	0.900	1.012	0.689	0.937	0.813
162951840	NP_001106171.1#Cyth2#19158# cytohesin-2 isoform 2 [Mus musculus]	1.181	0.821	0.602	0.916	0.759	1.317	0.794	1.000	0.634	0.838	0.736
6680836	NP_031617.1#Calr#12317# calreticulin precursor [Mus musculus]	1.001	0.892	0.681	0.838	0.759	1.317	0.687	0.812	0.597	0.744	0.670
530677964	NP_001268971.1#Sh3glb1#54673# endophilin-B1 isoform 3 [Mus musculus]	1.146	0.827	0.663	0.856	0.760	1.317	0.795	0.947	0.611	0.829	0.720
568987808	XP_006519137.1#Prmt5#27374# PREDICTED: protein arginine N-methyltransferase 5 isoform X2 [Mus musculus]	0.818	0.851	0.589	0.930	0.760	1.316	0.594	0.649	0.460	0.597	0.529
188219614	NP_035800.2#Uchl1#22223# ubiquitin carboxyl-terminal hydrolase isozyme L1 [Mus musculus]	1.180	0.966	0.650	0.870	0.760	1.316	0.854	1.019	0.778	1.098	0.938
158508568	NP_001103462.1#Ptpn11#19247# tyrosine-protein phosphatase non-receptor type 11 isoform b [Mus musculus]	1.401	0.775	0.717	0.803	0.760	1.316	0.959	1.169	0.744	0.906	0.825
755564500	XP_011245759.1#Usp9x#22284# PREDICTED: probable ubiquitin carboxyl-terminal hydrolase FAF-X isoform X1 [Mus musculus]	1.057	0.909	0.665	0.855	0.760	1.315	0.679	0.928	0.583	0.879	0.731
569009379	XP_006541599.1#Mcts1#68995# PREDICTED: malignant T-cell-amplified sequence 1 isoform X2 [Mus musculus]	1.079	0.847	0.658	0.863	0.760	1.315	0.750	0.891	0.553	0.834	0.694
568967188	XP_006513531.1#Mcu#215999# PREDICTED: calcium uniporter protein, mitochondrial isoform X1 [Mus musculus]	1.158	0.831	0.664	0.857	0.761	1.315	0.816	0.894	0.485	0.795	0.640
6681079	NP_031824.1#Ctsb#13030# cathepsin B preproprotein [Mus musculus]	1.273	0.854	0.637	0.884	0.761	1.314	0.893	1.043	0.722	0.933	0.827
549806752	NP_001272360.1#Eef1d#66656# elongation factor 1-delta isoform c [Mus musculus]	0.946	1.014	0.739	0.783	0.761	1.314	0.674	0.765	0.629	0.830	0.729
157277948	NP_033067.2#Rdx#19684# radixin isoform a [Mus musculus]	1.122	0.888	0.648	0.874	0.761	1.314	0.789	0.918	0.653	0.864	0.758
6678047	NP_033247.1#Snca#20617# alpha-synuclein [Mus musculus]	1.030	0.856	0.660	0.863	0.761	1.313	0.643	0.865	0.520	0.745	0.632
112181182	NP_031773.2#Cox5a#12858# cytochrome c oxidase subunit 5A, mitochondrial precursor [Mus musculus]	0.966	0.945	0.673	0.850	0.762	1.313	0.668	0.799	0.594	0.836	0.715
568940864	XP_006505700.1#Itpr1#16438# PREDICTED: inositol 1,4,5-trisphosphate receptor type 1 isoform X15 [Mus musculus]	1.679	0.684	0.678	0.846	0.762	1.312	1.151	1.407	0.773	0.977	0.875
6755911	NP_035790.1#Txn1#22166# thioredoxin [Mus musculus]	1.097	0.897	0.649	0.876	0.762	1.312	0.795	0.898	0.669	0.873	0.771
569003077	XP_006525616.1#Cdh2#12558# PREDICTED: cadherin-2 isoform X1 [Mus musculus]	1.034	0.812	0.647	0.878	0.763	1.311	0.687	0.989	0.495	0.656	0.575
755545692	XP_011242884.1#Faf2#76577# PREDICTED: FAS-associated factor 2 isoform X2 [Mus musculus]	1.384	0.919	0.721	0.805	0.763	1.311	0.977	1.135	0.870	1.070	0.970
568970106	XP_006514692.1#Lgalsl#216551# PREDICTED: galectin-related protein isoform X2 [Mus musculus]	1.231	0.863	0.654	0.872	0.763	1.311	0.795	1.054	0.726	0.910	0.818
13385932	NP_080711.1#Ufm1#67890# ubiquitin-fold modifier 1 precursor [Mus musculus]	0.921	0.970	0.661	0.866	0.764	1.309	0.676	0.731	0.573	0.790	0.681
22507361	NP_683749.1#Ly6g5c#114652# lymphocyte antigen 6 complex locus protein G5c precursor [Mus musculus]	1.176	0.819	0.684	0.844	0.764	1.309	0.879	0.918	0.665	0.806	0.736
9903609	NP_064297.1#Txn2#56551# thioredoxin, mitochondrial precursor [Mus musculus]	1.256	0.886	0.744	0.785	0.765	1.307	0.858	1.063	0.736	0.967	0.851
569007752	XP_006527376.1#Atad1#67979# PREDICTED: ATPase family AAA domain-containing protein 1 isoform X1 [Mus musculus]	1.021	0.955	0.685	0.846	0.765	1.307	0.670	0.892	0.661	0.830	0.746
9506983	NP_062284.1#Ppp2ca#19052# serine/threonine-protein phosphatase 2A catalytic subunit alpha isoform [Mus musculus]	1.143	0.855	0.685	0.845	0.765	1.307	0.741	0.971	0.692	0.886	0.789
569000227	XP_006524307.1#Syngap1#240057# PREDICTED: ras/Rap GTPase-activating protein SynGAP isoform X10 [Mus musculus]	1.037	0.792	0.698	0.834	0.766	1.306	0.732	0.867	0.566	0.717	0.642
19705424	NP_033465.1#Psmd3#22123# 26S proteasome non-ATPase regulatory subunit 3 [Mus musculus]	0.723	0.835	0.701	0.830	0.766	1.306	0.532	0.586	0.428	0.526	0.477
84781779	NP_001028477.1#Ndufb6#230075# NADH dehydrogenase [ubiquinone] 1 beta subcomplex subunit 6 [Mus musculus]	1.259	0.886	0.655	0.877	0.766	1.306	0.880	1.170	0.785	0.985	0.885
755533985	XP_011241621.1#Ap3d1#11776# PREDICTED: AP-3 complex subunit delta-1 isoform X3 [Mus musculus]	1.253	0.818	0.676	0.857	0.766	1.305	0.860	1.056	0.702	0.876	0.789
755502503	XP_011246447.1#Tpd52#21985# PREDICTED: tumor protein D52 isoform X8 [Mus musculus]	1.041	0.882	0.687	0.846	0.766	1.305	0.758	0.886	0.615	0.777	0.696
568983719	XP_006517481.1#Cltb#74325# PREDICTED: clathrin light chain B isoform X1 [Mus musculus]	1.178	0.872	0.702	0.831	0.767	1.304	0.862	0.928	0.670	0.928	0.799
6679911	NP_032097.1#Gabrb3#14402# gamma-aminobutyric acid receptor subunit beta-3 isoform a precursor [Mus musculus]	0.942	0.920	0.720	0.815	0.767	1.303	0.643	0.802	0.572	0.757	0.665
15808998	NP_291045.1#Cecr6#94047# cat eye syndrome critical region protein 6 homolog [Mus musculus]	0.875	0.749	0.717	0.818	0.768	1.303	0.632	0.711	0.422	0.584	0.503
312596932	NP_001186115.1#Slc44a2#68682# choline transporter-like protein 2 isoform 1 [Mus musculus]	1.338	0.869	0.670	0.865	0.768	1.302	0.913	1.134	0.825	1.046	0.935
755500872	XP_011238157.1#Vps16#80743# PREDICTED: vacuolar protein sorting-associated protein 16 homolog isoform X2 [Mus musculus]	1.186	0.731	0.612	0.925	0.768	1.302	0.833	0.989	0.631	0.701	0.666
755534606	XP_011241762.1#Syn3#27204# PREDICTED: synapsin-3 isoform X4 [Mus musculus]	1.663	1.115	0.630	0.908	0.769	1.300	1.117	1.442	1.228	1.624	1.426
568995493	XP_006522270.1#Abcf3#27406# PREDICTED: ATP-binding cassette sub-family F member 3 isoform X1 [Mus musculus]	1.108	0.802	0.687	0.851	0.769	1.300	0.792	0.913	0.607	0.760	0.684
329664963	NP_001192314.1#Actr3#74117# actin-related protein 3 [Mus musculus]	1.035	0.816	0.699	0.840	0.770	1.299	0.918	1.038	0.605	0.811	0.708
568986489	XP_006518504.1#Adk#11534# PREDICTED: adenosine kinase isoform X2 [Mus musculus]	1.343	0.858	0.668	0.872	0.770	1.299	0.885	1.183	0.785	0.990	0.888
238231384	NP_032972.3#Psmb6#19175# proteasome subunit beta type-6 precursor [Mus musculus]	1.174	0.757	0.673	0.867	0.770	1.299	0.778	1.031	0.617	0.750	0.683
568917774	XP_006499945.1#Nckap1#50884# PREDICTED: nck-associated protein 1 isoform X2 [Mus musculus]	1.181	0.817	0.654	0.887	0.770	1.298	0.925	1.009	0.686	0.871	0.778
568995358	XP_006522204.1#Usp7#252870# PREDICTED: ubiquitin carboxyl-terminal hydrolase 7 isoform X3 [Mus musculus]	1.122	0.953	0.678	0.864	0.771	1.297	0.719	0.988	0.529	0.844	0.686
83715998	NP_031533.2#Atp5k#11958# ATP synthase subunit e, mitochondrial [Mus musculus]	1.146	0.943	0.656	0.887	0.771	1.297	0.878	0.897	0.645	0.884	0.764
357588422	NP_001239496.1#Sez6l2#233878# seizure 6-like protein 2 isoform 3 precursor [Mus musculus]	0.971	0.771	0.682	0.860	0.771	1.297	0.668	0.830	0.506	0.649	0.578
568945112	XP_006539726.1#Ppp5c#19060# PREDICTED: serine/threonine-protein phosphatase 5 isoform X1 [Mus musculus]	1.152	0.823	0.637	0.906	0.771	1.296	0.790	1.114	0.681	0.906	0.794
161016826	NP_031900.3#Reep5#13476# receptor expression-enhancing protein 5 [Mus musculus]	1.260	0.847	0.704	0.839	0.772	1.296	0.841	1.103	0.693	0.951	0.822
23956148	NP_080011.1#Map1lc3a#66734# microtubule-associated proteins 1A/1B light chain 3A [Mus musculus]	1.126	0.821	0.682	0.862	0.772	1.296	0.759	0.979	0.574	0.853	0.714
568948233	XP_006541123.1#Sv2b#64176# PREDICTED: synaptic vesicle glycoprotein 2B isoform X1 [Mus musculus]	1.130	0.746	0.682	0.862	0.772	1.295	0.789	0.907	0.537	0.720	0.628
33859690	NP_080728.1#Coq9#67914# ubiquinone biosynthesis protein COQ9, mitochondrial precursor [Mus musculus]	1.103	0.966	0.649	0.896	0.772	1.295	0.806	0.897	0.704	0.941	0.822
6754920	NP_035084.1#Ncam2#17968# neural cell adhesion molecule 2 isoform b precursor [Mus musculus]	0.978	0.851	0.663	0.881	0.772	1.295	0.706	0.865	0.534	0.729	0.631
755495480	XP_006496740.2#Kcnj10#16513# PREDICTED: ATP-sensitive inward rectifier potassium channel 10 isoform X1 [Mus musculus]	1.183	0.970	0.639	0.906	0.772	1.295	0.826	1.001	0.711	1.062	0.886
6678551	NP_033523.1#Vamp2#22318# vesicle-associated membrane protein 2 [Mus musculus]	1.119	0.851	0.694	0.852	0.773	1.294	0.790	0.943	0.619	0.869	0.744
13385558	NP_080337.1#Ndufb8#67264# NADH dehydrogenase [ubiquinone] 1 beta subcomplex subunit 8, mitochondrial precursor [Mus musculus]	1.260	0.975	0.658	0.887	0.773	1.294	0.829	1.119	0.765	1.135	0.950
227430399	NP_001153084.1#Lrrc57#66606# leucine-rich repeat-containing protein 57 isoform c [Mus musculus]	1.176	0.788	0.693	0.853	0.773	1.294	0.863	0.955	0.594	0.836	0.715

254587958	NP_079793.2#Rtca#66368# RNA 3'-terminal phosphate cyclase [Mus musculus]	1.069	0.867	0.698	0.849	0.773	1.293	0.759	0.886	0.641	0.806	0.723
166235165	NP_033331.2#Syp#20977# synaptophysin [Mus musculus]	1.166	0.792	0.684	0.863	0.774	1.293	0.816	0.884	0.633	0.788	0.711
261862282	NP_035531.3#Slc2a3#20527# solute carrier family 2, facilitated glucose transporter member 3 [Mus musculus]	1.177	0.942	0.700	0.848	0.774	1.292	0.822	1.020	0.704	0.947	0.826
755551182	XP_006520651.2#Pde1b#18574# PREDICTED: calcium/calmodulin-dependent 3',5'-cyclic nucleotide phosphodiesterase 1B isoform X1 [Mus musculus	1.031	1.204	0.687	0.862	0.774	1.291	0.749	0.850	0.856	1.070	0.963
13385652	NP_080423.1#Rps20#67427# 40S ribosomal protein S20 [Mus musculus]	0.917	0.956	0.746	0.803	0.774	1.291	0.628	0.791	0.574	0.783	0.678
30725780	NP_849251.1#Mal2#105853# protein MAL2 [Mus musculus]	1.074	0.767	0.667	0.883	0.775	1.291	0.752	0.912	0.568	0.710	0.639
568982992	XP_006517138.1#Hapln1#12950# PREDICTED: hyaluronan and proteoglycan link protein 1 isoform X1 [Mus musculus]	1.052	0.882	0.708	0.842	0.775	1.291	0.817	0.997	0.562	0.825	0.693
51092272	NP_808574.2#Opcml#330908# opioid-binding protein/cell adhesion molecule precursor [Mus musculus]	1.056	0.732	0.685	0.865	0.775	1.290	0.795	0.920	0.501	0.750	0.625
755502283	XP_011238238.1#Arfgap1#228998# PREDICTED: ADP-ribosylation factor GTPase-activating protein 1 isoform X3 [Mus musculus]	1.035	0.874	0.729	0.821	0.775	1.290	0.676	0.928	0.586	0.816	0.701
568951392	XP_006508263.1#Ndufab1#70316# PREDICTED: acyl carrier protein, mitochondrial isoform X1 [Mus musculus]	1.302	0.857	0.659	0.891	0.775	1.290	0.908	1.148	0.822	0.998	0.910
568977589	XP_006515077.1#Prkar2b#19088# PREDICTED: cAMP-dependent protein kinase type II-beta regulatory subunit isoform X1 [Mus musculus]	1.031	0.920	0.665	0.885	0.775	1.290	0.765	0.848	0.666	0.806	0.736
21312260	NP_082546.1#Aldh1b1#72535# aldehyde dehydrogenase X, mitochondrial precursor [Mus musculus]	1.257	0.907	0.713	0.838	0.775	1.290	0.911	1.045	0.807	0.966	0.886
6679501	NP_032973.1#Psmc1#19179# 26S protease regulatory subunit 4 [Mus musculus]	1.094	0.780	0.609	0.942	0.775	1.290	0.919	0.929	0.614	0.780	0.697
755509256	XP_006538588.2#Eno1#13806# PREDICTED: alpha-enolase isoform X1 [Mus musculus]	1.054	0.905	0.673	0.878	0.775	1.290	0.741	0.866	0.618	0.844	0.731
21624617	NP_081018.1#Ndufaf4#68493# NADH dehydrogenase [ubiquinone] 1 alpha subcomplex assembly factor 4 [Mus musculus]	0.915	0.978	0.670	0.881	0.775	1.290	0.669	0.752	0.642	0.746	0.694
31981086	NP_080270.2#Efhd2#27984# EF-hand domain-containing protein D2 [Mus musculus]	1.108	0.854	0.669	0.883	0.776	1.289	0.769	0.975	0.640	0.852	0.746
256000796	NP_001157728.1#Tpm1#22003# tropomyosin alpha-1 chain isoform Tpm1.12br [Mus musculus]	1.061	0.883	0.665	0.886	0.776	1.289	0.755	0.937	0.631	0.840	0.736
569005647	XP_006531743.1#Pitpnm1#18739# PREDICTED: membrane-associated phosphatidylinositol transfer protein 1 isoform X1 [Mus musculus]	1.187	0.918	0.683	0.869	0.776	1.288	0.926	0.917	0.701	0.992	0.846
145699091	NP_001005510.2#Syne2#319565# nesprin-2 [Mus musculus]	1.239	0.982	0.738	0.815	0.777	1.288	0.924	1.002	0.855	1.036	0.945
568955318	XP_006509666.1#Rab3a#19339# PREDICTED: ras-related protein Rab-3A isoform X1 [Mus musculus]	1.117	0.816	0.684	0.869	0.777	1.287	0.798	0.897	0.541	0.757	0.649
755539425	XP_011247497.1#Fam114a2#67726# PREDICTED: protein FAM114A2 isoform X1 [Mus musculus]	1.108	0.921	0.645	0.910	0.777	1.286	0.786	0.936	0.647	0.940	0.793
568993153	XP_006521374.1#Eif3h#68135# PREDICTED: eukaryotic translation initiation factor 3 subunit H isoform X1 [Mus musculus]	1.050	0.820	0.685	0.870	0.777	1.286	0.720	0.913	0.577	0.762	0.669
29825827	NP_821170.1#Vps26b#69091# vacuolar protein sorting-associated protein 26B [Mus musculus]	1.255	0.821	0.698	0.857	0.778	1.286	0.897	1.054	0.680	0.922	0.801
568905198	XP_006495497.1#Atp6v1h#108664# PREDICTED: V-type proton ATPase subunit H isoform X2 [Mus musculus]	1.072	0.868	0.706	0.850	0.778	1.286	0.748	0.904	0.655	0.833	0.744
428673529	NP_001258802.1#Ano10#102566# anoctamin-10 isoform 2 [Mus musculus]	0.976	0.990	0.726	0.831	0.778	1.285	0.727	0.792	0.613	0.891	0.752
157277969	NP_444493.1#Hnrnpa3#229279# heterogeneous nuclear ribonucleoprotein A3 isoform c [Mus musculus]	1.181	0.886	0.700	0.857	0.778	1.285	0.894	1.037	0.741	0.981	0.861
13195674	NP_077249.1#Rab6a#19346# ras-related protein Rab-6A isoform 2 [Mus musculus]	0.920	0.940	0.687	0.870	0.779	1.284	0.714	0.850	0.594	0.811	0.702
40018610	NP_796188.2#Snrnp200#320632# U5 small nuclear ribonucleoprotein 200 kDa helicase [Mus musculus]	0.905	0.913	0.678	0.880	0.779	1.284	0.624	0.785	0.546	0.740	0.643
38371755	NP_081269.1#Ddah1#69219# N(G),N(G)-dimethylarginine dimethylaminohydrolase 1 [Mus musculus]	1.073	0.987	0.730	0.827	0.779	1.284	0.834	0.924	0.667	0.994	0.830
568929824	XP_006503492.1#Sgip1#73094# PREDICTED: SH3-containing GRB2-like protein 3-interacting protein 1 isoform X3 [Mus musculus]	1.141	0.848	0.721	0.836	0.779	1.284	0.946	1.042	0.745	0.984	0.865
20070408	NP_613061.1#Gldc#104174# glycine dehydrogenase (decarboxylating), mitochondrial precursor [Mus musculus]	1.337	0.988	0.647	0.911	0.779	1.284	0.950	1.132	0.892	1.166	1.029
21450055	NP_659096.1#Nrbp2#223649# nuclear receptor-binding protein 2 [Mus musculus]	1.025	0.925	0.718	0.840	0.779	1.283	0.686	0.911	0.666	0.811	0.738
257467657	NP_918950.2#D630045J12Rik#330286# UPF0606 protein KIAA1549 precursor [Mus musculus]	0.912	0.763	0.656	0.902	0.779	1.283	0.670	0.751	0.468	0.617	0.542
254675178	NP_032949.3#Prkar1b#19085# cAMP-dependent protein kinase type I-beta regulatory subunit [Mus musculus]	1.118	0.864	0.680	0.878	0.779	1.283	0.795	0.948	0.636	0.878	0.757
33186863	NP_058018.2#Rpl13#270106# 60S ribosomal protein L13 [Mus musculus]	1.015	0.926	0.664	0.896	0.780	1.282	0.695	0.888	0.630	0.836	0.733
568915917	XP_006499045.1#Sirpa#19261# PREDICTED: tyrosine-protein phosphatase non-receptor type substrate 1 isoform X2 [Mus musculus]	1.151	0.808	0.700	0.861	0.780	1.282	0.778	0.968	0.616	0.796	0.706
12963723	NP_076026.1#Rab3b#69908# ras-related protein Rab-3B [Mus musculus]	1.004	1.153	0.672	0.889	0.780	1.282	0.748	0.820	0.730	1.077	0.904
21312204	NP_077219.1#Hdhd3#72748# haloacid dehalogenase-like hydrolase domain-containing protein 3 [Mus musculus]	0.892	0.905	0.675	0.885	0.780	1.281	0.629	0.763	0.561	0.699	0.630
755497128	XP_006497718.2#Dnm1#13429# PREDICTED: dynamin-1 isoform X7 [Mus musculus]	1.303	0.997	0.714	0.847	0.780	1.281	0.955	1.080	0.876	1.153	1.015
145587092	NP_034936.1#Mllt4#17356# afadin [Mus musculus]	0.996	0.902	0.732	0.829	0.781	1.281	0.742	0.813	0.565	0.837	0.701
6681071	NP_031819.1#Cstb#13014# cystatin-B [Mus musculus]	0.916	0.979	0.667	0.894	0.781	1.281	0.619	0.812	0.615	0.786	0.701
226693349	NP_031621.3#Camk2b#12323# calcium/calmodulin-dependent protein kinase type II subunit beta isoform 2 [Mus musculus]	1.109	0.893	0.687	0.875	0.781	1.281	0.755	0.950	0.653	0.838	0.746
229608940	NP_001153489.1#Gnb1#14688# guanine nucleotide-binding protein G(I)/G(S)/G(T) subunit beta-1 [Mus musculus]	1.213	0.765	0.674	0.889	0.781	1.280	0.856	1.031	0.683	0.840	0.761
568987040	XP_006518774.1#Ppp3cb#19056# PREDICTED: serine/threonine-protein phosphatase 2B catalytic subunit beta isoform isoform X5 [Mus musculus]	1.216	0.846	0.726	0.836	0.781	1.280	0.883	1.016	0.731	0.875	0.803
31981600	NP_035015.2#Ndufa2#17991# NADH dehydrogenase [ubiquinone] 1 alpha subcomplex subunit 2 [Mus musculus]	1.382	0.862	0.683	0.879	0.781	1.280	1.056	1.103	0.835	1.026	0.930
19527026	NP_598568.1#Lrrc59#98238# leucine-rich repeat-containing protein 59 [Mus musculus]	1.265	0.807	0.679	0.884	0.781	1.280	1.014	0.962	0.687	0.908	0.797
6753960	NP_034396.1#Gda#14544# guanine deaminase [Mus musculus]	1.021	0.884	0.673	0.890	0.781	1.280	0.720	0.877	0.581	0.811	0.696
359279908	NP_001240669.1#Tpm3#59069# tropomyosin alpha-3 chain isoform Tpm3.2cy [Mus musculus]	1.064	0.944	0.710	0.853	0.781	1.280	0.764	0.976	0.659	0.913	0.786
10946854	NP_067438.1#Prps1#19139# ribose-phosphate pyrophosphokinase 1 [Mus musculus]	1.019	0.856	0.663	0.900	0.781	1.280	0.821	0.951	0.583	0.791	0.687
6677813	NP_033124.1#Rps8#20116# 40S ribosomal protein S8 [Mus musculus]	0.955	0.892	0.653	0.910	0.782	1.279	0.606	0.811	0.551	0.718	0.635
6755965	NP_035825.1#Vdac2#22334# voltage-dependent anion-selective channel protein 2 [Mus musculus]	1.107	0.897	0.687	0.876	0.782	1.279	0.799	0.941	0.660	0.876	0.768
755551956	XP_006521287.2#Shank3#58234# PREDICTED: SH3 and multiple ankyrin repeat domains protein 3 isoform X5 [Mus musculus]	1.046	0.968	0.666	0.898	0.782	1.279	0.767	0.869	0.715	0.869	0.792
116014342	NP_001070652.1#Bsg#12215# basigin isoform 2 precursor [Mus musculus]	1.211	0.861	0.712	0.851	0.782	1.279	0.887	1.082	0.708	0.883	0.796
13384778	NP_079672.1#Pgls#66171# 6-phosphogluconolactonase isoform 1 [Mus musculus]	0.954	0.929	0.698	0.867	0.782	1.278	0.668	0.730	0.587	0.745	0.666
162417957	NP_001104784.1#Ngef#53972# ephexin-1 isoform 1 [Mus musculus]	1.173	0.885	0.646	0.919	0.783	1.278	0.863	0.949	0.647	0.921	0.784
18158420	NP_076302.1#Camk2d#108058# calcium/calmodulin-dependent protein kinase type II subunit delta isoform 3 [Mus musculus]	0.872	0.966	0.716	0.850	0.783	1.277	0.578	0.787	0.572	0.744	0.658
568950486	XP_006507823.1#Fus#233908# PREDICTED: RNA-binding protein FUS isoform X2 [Mus musculus]	1.085	0.878	0.711	0.855	0.783	1.277	0.767	0.955	0.751	0.919	0.835
568971403	XP_006532165.1#Cbx1#12412# PREDICTED: chromobox protein homolog 1 isoform X5 [Mus musculus]	1.219	0.890	0.696	0.871	0.783	1.276	0.843	1.067	0.747	0.953	0.850
13386100	NP_080890.1#Ndufa5#68202# NADH dehydrogenase [ubiquinone] 1 alpha subcomplex subunit 5 [Mus musculus]	1.117	0.818	0.716	0.851	0.783	1.276	0.811	0.831	0.597	0.732	0.664
409191595	NP_001258513.1#Dnajc5#13002# dnaJ homolog subfamily C member 5 [Mus musculus]	1.238	0.937	0.694	0.873	0.783	1.276	0.909	1.028	0.802	1.017	0.910
12963633	NP_075801.1#Ndufa13#67184# NADH dehydrogenase [ubiquinone] 1 alpha subcomplex subunit 13 [Mus musculus]	0.962	0.894	0.718	0.850	0.784	1.276	0.773	0.905	0.615	0.804	0.710
31543976	NP_061359.2#Ywhag#22628# 14-3-3 protein gamma [Mus musculus]	0.947	0.840	0.705	0.862	0.784	1.276	0.676	0.816	0.598	0.728	0.663
262050551	NP_001159925.1#Pls3#102866# plastin-3 [Mus musculus]	1.819	0.915	0.647	0.921	0.784	1.276	1.366	1.485	1.099	1.510	1.304
13385044	NP_079868.1#Rpl35#66489# 60S ribosomal protein L35 [Mus musculus]	0.849	0.713	0.611	0.956	0.784	1.276	0.787	0.575	0.420	0.551	0.485
46309573	NP_035474.1#Sel1l#20338# protein sel-1 homolog 1 isoform b precursor [Mus musculus]	0.997	0.933	0.699	0.868	0.784	1.276	0.714	0.850	0.579	0.879	0.729
28201978	NP_780303.1#Pdhx#27402# pyruvate dehydrogenase protein X component, mitochondrial [Mus musculus]	0.920	0.901	0.761	0.807	0.784	1.276	0.701	0.742	0.551	0.750	0.650
568988256	XP_006519357.1#Rabggta#56187# PREDICTED: geranylgeranyl transferase type-2 subunit alpha isoform X1 [Mus musculus]	1.226	0.808	0.669	0.899	0.784	1.275	0.881	1.041	0.623	0.929	0.776
21312012	NP_080979.1#Ndufa8#68375# NADH dehydrogenase [ubiquinone] 1 alpha subcomplex subunit 8 [Mus musculus]	1.089	0.988	0.691	0.878	0.784	1.275	0.763	0.955	0.690	0.958	0.824
7710012	NP_057878.1#Crym#12971# ketimine reductase mu-crystallin [Mus musculus]	0.916	0.923	0.702	0.866	0.784	1.275	0.620	0.726	0.595	0.792	0.693
568982871	XP_006517079.1#Sncb#104069# PREDICTED: beta-synuclein isoform X1 [Mus musculus]	1.080	0.924	0.668	0.900	0.784	1.275	0.778	0.883	0.685	0.928	0.807
209862992	NP_001129558.1#Dpysl3#22240# dihydropyrimidinase-related protein 3 isoform 1 [Mus musculus]	0.959	0.971	0.665	0.904	0.785	1.275	0.712	0.901	0.618	0.922	0.770
755495853	XP_006497018.2#Sdhc#66052# PREDICTED: succinate dehydrogenase cytochrome b560 subunit, mitochondrial isoform X1 [Mus musculus]	1.021	0.950	0.720	0.850	0.785	1.274	0.706	0.890	0.663	0.932	0.797
19705578	NP_031535.2#Atp6v1b2#11966# V-type proton ATPase subunit B, brain isoform [Mus musculus]	1.099	0.841	0.699	0.870	0.785	1.274	0.775	0.961	0.624	0.841	0.732
9910452	NP_064304.1#Lamtor3#56692# ragulator complex protein LAMTOR3 [Mus musculus]	0.980	0.926	0.707	0.863	0.785	1.274	0.720	0.817	0.622	0.802	0.712
755510673	XP_006503649.2#Pclo#26875# PREDICTED: protein piccolo isoform X4 [Mus musculus]	1.056	0.831	0.687	0.883	0.785	1.274	0.729	0.927	0.584	0.796	0.690
568941915	XP_006506209.1#Srgap3#259302# PREDICTED: SLIT-ROBO Rho GTPase-activating protein 3 isoform X3 [Mus musculus]	0.969	0.879	0.716	0.855	0.785	1.274	0.727	0.795	0.600	0.737	0.668
569000052	XP_006524222.1#Safb2#224902# PREDICTED: scaffold attachment factor B2 isoform X3 [Mus musculus]	1.241	0.813	0.709	0.862	0.785	1.273	0.858	1.091	0.701	0.883	0.792
19526814	NP_598427.1#Ndufv1#17995# NADH dehydrogenase [ubiquinone] flavoprotein 1, mitochondrial precursor [Mus musculus]	1.186	0.883	0.688	0.882	0.785	1.273	0.850	1.054	0.671	0.937	0.804
16716597	NP_444492.1#Hsd17b11#114664# estradiol 17-beta-dehydrogenase 11 precursor [Mus musculus]	1.069	0.858	0.655	0.916	0.785	1.273	0.755	0.924	0.629	0.811	0.720
817379474	NP_001295379.1#Aldh2#11669# aldehyde dehydrogenase, mitochondrial isoform 2 precursor [Mus musculus]	1.200	0.921	0.707	0.863	0.785	1.273	0.866	1.007	0.772	0.991	0.882
568965313	XP_006512755.1#D10Bwg1379e#215821# PREDICTED: brefeldin A-inhibited guanine nucleotide-exchange protein 3 isoform X1 [Mus musculus]	1.232	0.892	0.703	0.868	0.785	1.273	0.842	0.976	0.681	0.864	0.773
357394970	NP_001239453.1#Picalm#233489# phosphatidylinositol-binding clathrin assembly protein isoform 6 [Mus musculus]	1.246	0.954	0.724	0.847	0.785	1.273	0.844	1.117	0.823	1.046	0.935
27754056	NP_080749.2#Tubb6#67951# tubulin beta-6 chain [Mus musculus]	1.604	0.838	0.672	0.899	0.786	1.273	1.280	1.240	0.877	1.234	1.055

568984258	XP_006517611.1#Map1b#17755# PREDICTED: microtubule-associated protein 1B isoform X1 [Mus musculus]	1.124	0.950	0.716	0.856	0.786	1.273	0.813	0.952	0.735	0.945	0.840
40254160	NP_083795.2#Rap2a#76108# ras-related protein Rap-2a precursor [Mus musculus]	1.153	0.868	0.666	0.906	0.786	1.273	0.770	1.021	0.650	0.803	0.727
568972104	XP_006532505.1#Nf1#18015# PREDICTED: neurofibromin isoform X7 [Mus musculus]	1.249	0.939	0.699	0.874	0.787	1.271	0.838	1.126	0.794	1.052	0.923
54291704	NP_694769.2#Pacs1#107975# phosphofurin acidic cluster sorting protein 1 [Mus musculus]	1.236	0.774	0.780	0.794	0.787	1.271	0.858	1.087	0.660	0.844	0.752
755519087	XP_006539595.2#Epn1#13854# PREDICTED: epsin-1 isoform X2 [Mus musculus]	1.174	0.868	0.680	0.894	0.787	1.270	0.854	1.017	0.677	0.930	0.803
568972574	XP_006532728.1#Eftud2#20624# PREDICTED: 116 kDa U5 small nuclear ribonucleoprotein component isoform X1 [Mus musculus]	1.115	0.847	0.633	0.942	0.787	1.270	0.822	0.932	0.614	0.868	0.741
51172612	NP_032122.1#Gc#14473# vitamin D-binding protein precursor [Mus musculus]	1.009	0.863	0.658	0.917	0.787	1.270	0.658	0.931	0.623	0.749	0.686
568933623	XP_006503727.1#Slc30a9#109108# PREDICTED: zinc transporter 9 isoform X1 [Mus musculus]	1.137	0.822	0.649	0.926	0.787	1.270	0.820	0.971	0.662	0.810	0.736
568918357	XP_006500218.1#Gorasp2#70231# PREDICTED: Golgi reassembly-stacking protein 2 isoform X1 [Mus musculus]	1.125	0.763	0.728	0.847	0.787	1.270	0.850	0.922	0.581	0.770	0.676
568986698	XP_006518606.1#Esd#13885# PREDICTED: S-formylglutathione hydrolase isoform X1 [Mus musculus]	1.124	0.893	0.720	0.855	0.788	1.270	0.781	0.990	0.660	0.921	0.790
568914105	XP_006498300.1#Pmpca#66865# PREDICTED: mitochondrial-processing peptidase subunit alpha isoform X1 [Mus musculus]	1.113	0.877	0.748	0.828	0.788	1.269	0.751	1.002	0.649	0.892	0.771
568995735	XP_006522387.1#Kng2#385643# PREDICTED: kininogen 2 isoform X1 [Mus musculus]	1.065	0.920	0.700	0.877	0.788	1.269	0.753	0.926	0.689	0.855	0.772
33563294	NP_473392.1#Pip4k2b#108083# phosphatidylinositol 5-phosphate 4-kinase type-2 beta [Mus musculus]	0.993	0.908	0.674	0.902	0.788	1.269	0.768	0.798	0.629	0.793	0.711
160707901	NP_038708.3#Syn1#20964# synapsin-1 isoform a [Mus musculus]	1.145	0.811	0.695	0.882	0.788	1.269	0.801	0.969	0.611	0.824	0.718
85861218	NP_001028472.2#Gmps#229363# GMP synthase [glutamine-hydrolyzing] [Mus musculus]	1.127	0.918	0.685	0.892	0.788	1.268	0.820	1.012	0.709	0.912	0.810
6754012	NP_034438.1#Gnao1#14681# guanine nucleotide-binding protein G(o) subunit alpha isoform A [Mus musculus]	1.153	0.845	0.699	0.879	0.789	1.268	0.879	1.060	0.732	0.929	0.830
568960933	XP_006510964.1#Snap91#20616# PREDICTED: clathrin coat assembly protein AP180 isoform X8 [Mus musculus]	1.175	0.856	0.688	0.890	0.789	1.268	0.888	1.032	0.681	0.924	0.802
689972387	NP_001289008.1#Fkbp1a#14225# peptidyl-prolyl cis-trans isomerase FKBP1A isoform 3 [Mus musculus]	0.897	0.914	0.672	0.905	0.789	1.268	0.664	0.757	0.550	0.790	0.670
568907654	XP_006496541.1#Tuba4a#22145# PREDICTED: tubulin alpha-4A chain isoform X1 [Mus musculus]	1.205	0.815	0.691	0.887	0.789	1.267	0.842	1.027	0.676	0.899	0.788
46559412	NP_848142.1#Pacsin1#23969# protein kinase C and casein kinase substrate in neurons protein 1 [Mus musculus]	1.183	0.819	0.709	0.869	0.789	1.267	0.846	1.024	0.702	0.865	0.784
157909797	NP_058567.2#Tomm40#53333# mitochondrial import receptor subunit TOM40 homolog [Mus musculus]	1.264	0.891	0.666	0.913	0.789	1.267	0.867	1.129	0.714	1.064	0.889
21313679	NP_082138.1#Atp5h#71679# ATP synthase subunit d, mitochondrial [Mus musculus]	1.231	1.003	0.708	0.871	0.789	1.267	0.866	1.024	0.758	0.966	0.862
83649713	NP_001032843.1#Baiap2#108100# brain-specific angiogenesis inhibitor 1-associated protein 2 isoform c [Mus musculus]	1.082	0.861	0.692	0.888	0.790	1.266	0.748	0.952	0.632	0.844	0.738
6680934	NP_031720.1#Chgb#12653# secretogranin-1 precursor [Mus musculus]	1.040	0.886	0.733	0.847	0.790	1.266	0.740	0.902	0.672	0.784	0.728
6753508	NP_034076.1#Cplx2#12890# complexin-2 [Mus musculus]	1.126	0.792	0.688	0.892	0.790	1.266	0.861	1.013	0.614	0.828	0.721
6677801	NP_033118.1#Rps17#20068# 40S ribosomal protein S17 [Mus musculus]	1.206	0.898	0.713	0.867	0.790	1.266	0.818	0.975	0.643	0.893	0.768
269784615	NP_001161421.1#Atp2b4#381290# plasma membrane calcium-transporting ATPase 4 isoform a [Mus musculus]	1.086	0.810	0.694	0.886	0.790	1.265	0.769	0.937	0.567	0.800	0.683
568955190	XP_006509606.1#Ncan#13004# PREDICTED: neurocan core protein isoform X1 [Mus musculus]	1.026	0.915	0.685	0.896	0.790	1.265	0.699	0.870	0.648	0.792	0.720
47059143	NP_081240.1#Ccdc124#234388# coiled-coil domain-containing protein 124 [Mus musculus]	1.076	0.857	0.690	0.891	0.790	1.265	0.780	0.922	0.649	0.810	0.729
226246593	NP_062759.2#Actr10#56444# actin-related protein 10 [Mus musculus]	1.043	0.893	0.716	0.866	0.791	1.265	0.697	0.953	0.631	0.842	0.736
683523935	NP_001288639.1#Mpdu1#24070# mannose-P-dolichol utilization defect 1 protein isoform 2 [Mus musculus]	1.148	0.876	0.735	0.846	0.791	1.265	0.891	0.926	0.660	0.929	0.795
568948700	XP_006541348.1#Cpt1c#78070# PREDICTED: carnitine O-palmitoyltransferase 1, brain isoform isoform X5 [Mus musculus]	1.202	0.823	0.700	0.882	0.791	1.264	0.865	1.037	0.676	0.890	0.783
27754144	NP_079592.2#Ndufb5#66046# NADH dehydrogenase [ubiquinone] 1 beta subcomplex subunit 5, mitochondrial precursor [Mus musculus]	0.931	0.930	0.727	0.855	0.791	1.264	0.697	0.840	0.595	0.784	0.689
172072657	NP_064382.3#Alg2#56737# alpha-1,3/1,6-mannosyltransferase ALG2 [Mus musculus]	1.094	0.874	0.701	0.881	0.791	1.264	0.740	1.012	0.641	0.837	0.739
6753618	NP_034157.1#Ddt#13202# D-dopachrome decarboxylase [Mus musculus]	0.548	0.845	0.702	0.880	0.791	1.264	0.395	0.491	0.322	0.424	0.373
569019884	XP_006537479.1#Atp6v1g2#66237# PREDICTED: V-type proton ATPase subunit G 2 isoform X1 [Mus musculus]	1.158	0.822	0.790	0.793	0.792	1.263	0.843	0.990	0.705	0.802	0.754
13195624	NP_077159.1#Ndufa10#67273# NADH dehydrogenase [ubiquinone] 1 alpha subcomplex subunit 10, mitochondrial precursor [Mus musculus]	1.210	0.898	0.719	0.864	0.792	1.263	0.923	1.024	0.738	0.969	0.853
9790219	NP_062745.1#Dstn#56431# destrin [Mus musculus]	1.004	0.899	0.699	0.885	0.792	1.262	0.790	0.909	0.678	0.811	0.745
568952682	XP_006508569.1#Nap1l4#17955# PREDICTED: nucleosome assembly protein 1-like 4 isoform X4 [Mus musculus]	1.032	0.918	0.655	0.929	0.792	1.262	0.726	0.880	0.633	0.911	0.772
229577358	NP_001153360.1#Ociad1#68095# OCIA domain-containing protein 1 isoform 3 [Mus musculus]	1.093	0.926	0.687	0.898	0.793	1.262	0.787	0.946	0.620	0.984	0.802
576067874	NP_001276603.1#Fbxo41#330369# F-box only protein 41 [Mus musculus]	1.215	0.711	0.692	0.893	0.793	1.262	0.886	0.991	0.595	0.759	0.677
568925931	XP_006537609.1#Wwp1#107568# PREDICTED: NEDD4-like E3 ubiquitin-protein ligase WWP1 isoform X2 [Mus musculus]	0.992	0.869	0.690	0.896	0.793	1.262	0.691	0.882	0.580	0.788	0.684
39652626	NP_035924.2#Bpnt1#23827# 3'(2'),5'-bisphosphate nucleotidase 1 [Mus musculus]	1.162	0.831	0.736	0.849	0.793	1.261	0.821	0.983	0.691	0.908	0.800
568920250	XP_006500693.1#Psma7#26444# PREDICTED: proteasome subunit alpha type-7 isoform X1 [Mus musculus]	1.010	0.756	0.741	0.845	0.793	1.261	0.734	0.869	0.517	0.695	0.606
568910653	XP_006496809.1#Tnr#21960# PREDICTED: tenascin-R isoform X3 [Mus musculus]	1.140	0.919	0.687	0.899	0.793	1.261	0.816	1.020	0.724	0.941	0.833
568983700	XP_006517472.1#Tppp#72948# PREDICTED: tubulin polymerization-promoting protein isoform X1 [Mus musculus]	1.141	0.868	0.712	0.874	0.793	1.261	0.809	0.985	0.628	0.876	0.752
227499234	NP_001153096.1#Rnpep#215615# aminopeptidase B isoform 2 [Mus musculus]	1.150	0.883	0.739	0.848	0.793	1.260	0.813	1.031	0.716	0.945	0.830
568917807	XP_006499960.1#Stk39#53416# PREDICTED: STE20/SPS1-related proline-alanine-rich protein kinase isoform X2 [Mus musculus]	1.049	0.866	0.728	0.859	0.794	1.260	0.857	0.946	0.662	0.898	0.780
568972247	XP_006532574.1#Prkar1a#19084# PREDICTED: cAMP-dependent protein kinase type I-alpha regulatory subunit isoform X1 [Mus musculus]	1.070	0.951	0.681	0.906	0.794	1.260	0.766	0.932	0.693	0.923	0.808
112807195	NP_034072.2#Cox5b#12859# cytochrome c oxidase subunit 5B, mitochondrial [Mus musculus]	1.335	0.916	0.728	0.860	0.794	1.260	0.915	1.177	0.876	1.149	1.013
6754240	NP_034601.1#Hpca#15444# neuron-specific calcium-binding protein hippocalcin [Mus musculus]	1.453	0.817	0.699	0.889	0.794	1.260	0.985	1.219	0.767	1.034	0.901
568971897	XP_006532405.1#Lasp1#16796# PREDICTED: LIM and SH3 domain protein 1 isoform X2 [Mus musculus]	1.071	0.860	0.691	0.897	0.794	1.259	0.777	0.924	0.601	0.862	0.731
568952442	XP_006536292.1#Rplp2#67186# PREDICTED: 60S acidic ribosomal protein P2 isoform X1 [Mus musculus]	1.118	0.914	0.698	0.892	0.795	1.258	0.810	0.993	0.675	0.904	0.789
31981830	NP_034075.2#Cox7a2#12866# cytochrome c oxidase subunit 7A2, mitochondrial precursor [Mus musculus]	1.055	0.920	0.670	0.920	0.795	1.258	0.773	1.060	0.701	1.059	0.880
31560517	NP_036105.2#Rpl27a#26451# 60S ribosomal protein L27a [Mus musculus]	1.022	0.775	0.666	0.924	0.795	1.258	0.907	0.704	0.577	0.685	0.631
34610235	NP_918943.1#Rtn4#68585# reticulon-4 isoform A [Mus musculus]	1.021	0.905	0.710	0.880	0.795	1.258	0.784	0.854	0.720	0.976	0.848
9790259	NP_062720.1#Pdcd5#56330# programmed cell death protein 5 [Mus musculus]	1.065	0.850	0.700	0.891	0.795	1.257	0.770	0.923	0.608	0.833	0.720
568935665	XP_006535018.1#Cops4#26891# PREDICTED: COP9 signalosome complex subunit 4 isoform X3 [Mus musculus]	1.060	0.957	0.716	0.875	0.796	1.257	0.785	0.870	0.667	0.922	0.795
225543319	NP_033529.3#Vcp#269523# transitional endoplasmic reticulum ATPase [Mus musculus]	1.207	0.880	0.716	0.875	0.796	1.257	0.884	1.056	0.740	0.946	0.843
158303337	NP_001103445.1#Synpo#104027# synaptopodin isoform B [Mus musculus]	0.992	0.832	0.708	0.884	0.796	1.257	0.675	0.894	0.573	0.784	0.679
156231075	NP_001095900.1#Arfgef1#211673# brefeldin A-inhibited guanine nucleotide-exchange protein 1 [Mus musculus]	1.087	0.764	0.695	0.896	0.796	1.257	0.825	0.904	0.548	0.773	0.660
569009085	XP_006541454.1#Sept6#56526# PREDICTED: septin-6 isoform X5 [Mus musculus]	1.198	0.793	0.694	0.897	0.796	1.257	0.880	1.029	0.618	0.875	0.746
568958751	XP_006510025.1#Hyou1#12282# PREDICTED: hypoxia up-regulated protein 1 isoform X2 [Mus musculus]	1.034	0.892	0.695	0.897	0.796	1.256	0.783	0.862	0.616	0.873	0.745
6671539	NP_031464.1#Aldoa#11674# fructose-bisphosphate aldolase A isoform 2 [Mus musculus]	1.052	0.893	0.692	0.900	0.796	1.256	0.787	0.945	0.650	0.917	0.783
166851828	NP_079589.2#Atp5d#66043# ATP synthase subunit delta, mitochondrial precursor [Mus musculus]	1.097	0.933	0.671	0.922	0.796	1.256	0.770	0.953	0.706	0.855	0.780
30520029	NP_848777.1#Atcay#16467# caytaxin [Mus musculus]	1.180	0.894	0.681	0.912	0.796	1.256	0.847	1.033	0.707	0.974	0.840
13385054	NP_079873.1#Ndufb3#66495# NADH dehydrogenase [ubiquinone] 1 beta subcomplex subunit 3 [Mus musculus]	1.000	0.815	0.724	0.869	0.796	1.256	0.725	0.779	0.524	0.665	0.595
22094075	NP_031477.1#Slc25a5#11740# ADP/ATP translocase 2 [Mus musculus]	1.036	0.926	0.690	0.903	0.796	1.256	0.789	0.938	0.667	0.944	0.806
569005698	XP_006531767.1#Sptbn2#20743# PREDICTED: spectrin beta chain, non-erythrocytic 2 isoform X1 [Mus musculus]	1.219	0.805	0.707	0.886	0.796	1.256	0.869	1.040	0.685	0.922	0.804
84579915	NP_001033744.1#Gng7#14708# guanine nucleotide-binding protein G(I)/G(S)/G(O) subunit gamma-7 [Mus musculus]	0.963	1.417	0.766	0.827	0.796	1.256	0.725	0.807	0.976	1.199	1.088
31543349	NP_032766.2#Nsf#18195# vesicle-fusing ATPase [Mus musculus]	1.143	0.898	0.708	0.885	0.797	1.255	0.850	1.007	0.699	0.942	0.820
568974214	XP_006533504.1#Mprip#26936# PREDICTED: myosin phosphatase Rho-interacting protein isoform X14 [Mus musculus]	1.266	0.947	0.648	0.945	0.797	1.255	0.883	1.134	0.778	1.132	0.955
8567410	NP_038709.1#Syn2#20965# synapsin-2 isoform IIb [Mus musculus]	1.047	0.889	0.719	0.874	0.797	1.255	0.767	0.903	0.629	0.848	0.739
6679263	NP_032837.1#Pdha2#18598# pyruvate dehydrogenase E1 component subunit alpha, testis-specific form, mitochondrial precursor [Mus musculus]	1.272	0.771	0.701	0.893	0.797	1.255	0.900	1.127	0.677	0.885	0.781
76881807	NP_033270.3#Serpina1b#20701# alpha-1-antitrypsin 1-2 precursor [Mus musculus]	1.025	0.994	0.709	0.884	0.797	1.255	0.776	0.867	0.758	0.875	0.816
7242197	NP_035315.1#Psmb1#19170# proteasome subunit beta type-1 precursor [Mus musculus]	1.024	0.769	0.696	0.898	0.797	1.255	0.687	0.825	0.595	0.771	0.683
755542314	XP_011242321.1#Ptprn2#19276# PREDICTED: receptor-type tyrosine-protein phosphatase N2 isoform X1 [Mus musculus]	1.011	0.961	0.731	0.863	0.797	1.255	0.702	0.909	0.667	0.881	0.774
568941435	XP_006505979.1#Usp5#22225# PREDICTED: ubiquitin carboxyl-terminal hydrolase 5 isoform X2 [Mus musculus]	1.188	0.898	0.690	0.903	0.797	1.255	0.850	1.011	0.703	0.934	0.818
755503893	XP_011238429.1#Nbea#26422# PREDICTED: neurobeachin isoform X6 [Mus musculus]	0.974	0.843	0.711	0.883	0.797	1.255	0.728	0.869	0.572	0.773	0.673
568937008	XP_006530299.1#Camkk2#207565# PREDICTED: calcium/calmodulin-dependent protein kinase kinase 2 isoform X6 [Mus musculus]	1.108	0.836	0.709	0.886	0.797	1.254	0.896	0.871	0.647	0.831	0.739
21703842	NP_663397.1#Rtcb#28088# tRNA-splicing ligase RtcB homolog [Mus musculus]	1.021	0.960	0.715	0.880	0.797	1.254	0.727	0.901	0.664	0.899	0.782
31980937	NP_080717.2#Pef1#67898# peflin [Mus musculus]	1.094	0.961	0.659	0.936	0.797	1.254	0.840	0.905	0.707	0.970	0.838
31982332	NP_032157.2#Glul#14645# glutamine synthetase [Mus musculus]	1.229	0.887	0.686	0.908	0.797	1.254	0.886	1.069	0.769	1.010	0.889

755520215	XP_011249044.1#Mboat7#77582# PREDICTED: lysophospholipid acyltransferase 7 isoform X2 [Mus musculus]	0.815	0.784	0.635	0.960	0.798	1.254	0.658	0.642	0.445	0.575	0.510
14149647	NP_035422.1#Rpl9#20005# 60S ribosomal protein L9 [Mus musculus]	0.971	0.839	0.675	0.921	0.798	1.253	0.755	0.794	0.593	0.706	0.650
254553321	NP_058655.3#Rps3a1#20091# 40S ribosomal protein S3a [Mus musculus]	1.121	0.840	0.682	0.914	0.798	1.253	0.787	0.982	0.623	0.866	0.744
160333553	NP_033102.2#Rpl12#269261# 60S ribosomal protein L12 [Mus musculus]	1.188	0.860	0.704	0.892	0.798	1.253	0.869	1.027	0.727	0.903	0.815
6755196	NP_036096.1#Psma4#26441# proteasome subunit alpha type-4 [Mus musculus]	1.014	0.707	0.682	0.915	0.798	1.253	0.734	0.886	0.495	0.650	0.573
29789104	NP_062606.1#Napb#17957# beta-soluble NSF attachment protein [Mus musculus]	1.064	0.872	0.701	0.895	0.798	1.253	0.783	0.938	0.638	0.839	0.739
110626050	NP_083707.1#Them4#75778# acyl-coenzyme A thioesterase THEM4 [Mus musculus]	1.188	0.829	0.694	0.903	0.798	1.253	0.895	1.005	0.669	0.905	0.787
158966670	NP_001103681.1#Anxa6#11749# annexin A6 isoform b [Mus musculus]	0.875	1.039	0.726	0.871	0.799	1.252	0.657	0.843	0.620	0.891	0.755
170295840	NP_035718.2#Trim28#21849# transcription intermediary factor 1-beta [Mus musculus]	1.131	0.734	0.626	0.971	0.799	1.252	0.902	0.905	0.526	0.799	0.663
45592934	NP_033033.1#Rac1#19353# ras-related C3 botulinum toxin substrate 1 precursor [Mus musculus]	1.079	0.884	0.655	0.942	0.799	1.252	0.802	0.918	0.631	0.888	0.760
568942584	XP_006506531.1#Chchd3#66075# PREDICTED: MICOS complex subunit Mic19 isoform X2 [Mus musculus]	1.118	0.953	0.662	0.936	0.799	1.252	0.822	0.967	0.711	0.994	0.852
550821975	NP_001272410.1#Hp1bp3#15441# heterochromatin protein 1-binding protein 3 isoform 5 [Mus musculus]	1.176	0.946	0.680	0.918	0.799	1.252	0.823	1.007	0.700	0.930	0.815
12963591	NP_075720.1#Stoml2#66592# stomatin-like protein 2, mitochondrial [Mus musculus]	1.074	0.871	0.650	0.948	0.799	1.252	0.727	0.988	0.652	0.843	0.747
6678347	NP_033408.1#Thy1#21838# thy-1 membrane glycoprotein preproprotein [Mus musculus]	1.008	0.885	0.712	0.885	0.799	1.252	0.751	0.865	0.593	0.822	0.707
12963615	NP_075768.1#Tubb3#22152# tubulin beta-3 chain [Mus musculus]	1.109	0.980	0.709	0.889	0.799	1.251	0.792	0.989	0.776	0.996	0.886
597517957	NP_001277968.1#Cds2#110911# phosphatidate cytidylyltransferase 2 isoform b [Mus musculus]	0.798	0.872	0.720	0.878	0.799	1.251	0.576	0.709	0.483	0.619	0.551
568896389	XP_006544502.1#LOC102641332#102641332# PREDICTED: GTP-binding nuclear protein Ran [Mus musculus]	1.049	0.877	0.704	0.895	0.800	1.251	0.802	0.972	0.614	0.791	0.702
25742730	NP_742083.1#Rpl32#19951# 60S ribosomal protein L32 [Mus musculus]	1.003	0.874	0.707	0.892	0.800	1.251	0.682	0.922	0.604	0.797	0.701
568967029	XP_006513453.1#Rps15#20054# PREDICTED: 40S ribosomal protein S15 isoform X1 [Mus musculus]	1.072	0.806	0.692	0.907	0.800	1.251	0.730	0.984	0.611	0.771	0.691
85861220	NP_001034284.1#Gria2#14800# glutamate receptor 2 isoform 3 precursor [Mus musculus]	1.233	0.731	0.713	0.886	0.800	1.250	0.897	1.111	0.664	0.796	0.730
126517505	NP_062689.2#Kcmf1#74287# E3 ubiquitin-protein ligase KCMF1 [Mus musculus]	1.238	0.835	0.641	0.959	0.800	1.250	0.880	1.099	0.637	1.016	0.827
568938111	XP_006504490.1#Clip2#269713# PREDICTED: CAP-Gly domain-containing linker protein 2 isoform X1 [Mus musculus]	1.122	0.890	0.710	0.890	0.800	1.250	0.837	1.004	0.698	0.939	0.819
568997213	XP_006522939.1#Atp5j#11957# PREDICTED: ATP synthase-coupling factor 6, mitochondrial isoform X1 [Mus musculus]	1.183	0.928	0.696	0.903	0.800	1.250	0.889	1.041	0.760	1.030	0.895
188219524	NP_036123.3#Dpysl4#26757# dihydropyrimidinase-related protein 4 [Mus musculus]	1.079	0.937	0.733	0.867	0.800	1.250	0.764	1.021	0.757	0.913	0.835
84370353	NP_001033702.1#Olfm1#56177# noelin isoform c precursor [Mus musculus]	1.179	0.792	0.652	0.949	0.800	1.250	0.849	1.028	0.563	0.884	0.723
10181184	NP_065607.1#Atp5j2#57423# ATP synthase subunit f, mitochondrial [Mus musculus]	1.196	0.944	0.689	0.911	0.800	1.250	0.850	1.065	0.783	1.022	0.903
312032384	NP_001185815.1#Dpp6#13483# dipeptidyl aminopeptidase-like protein 6 isoform 4 [Mus musculus]	1.011	0.896	0.701	0.899	0.800	1.250	0.686	0.895	0.597	0.823	0.710
163792196	NP_058582.3#Gria3#53623# glutamate receptor 3 isoform a precursor [Mus musculus]	1.075	0.805	0.690	0.911	0.800	1.250	0.820	0.973	0.631	0.735	0.683
30424918	NP_780475.1#Epm2aip1#77781# EPM2A-interacting protein 1 [Mus musculus]	1.076	0.906	0.760	0.840	0.800	1.249	0.799	0.923	0.711	0.849	0.780
731184185	NP_001289899.1#Ap2m1#11773# AP-2 complex subunit mu isoform 2 [Mus musculus]	1.102	0.840	0.719	0.882	0.800	1.249	0.814	0.971	0.616	0.838	0.727
9790017	NP_062740.1#Ptges3#56351# prostaglandin E synthase 3 [Mus musculus]	1.062	0.911	0.700	0.901	0.800	1.249	0.806	0.994	0.685	0.892	0.788
356582426	NP_001239190.1#Npm1#18148# nucleophosmin isoform 3 [Mus musculus]	1.157	0.800	0.714	0.887	0.800	1.249	0.863	1.014	0.645	0.837	0.741
569007565	XP_006527286.1#Arl3#56350# PREDICTED: ADP-ribosylation factor-like protein 3 isoform X1 [Mus musculus]	1.061	0.892	0.740	0.862	0.801	1.249	0.749	0.959	0.623	0.902	0.762
6679935	NP_032109.1#Gap43#14432# neuromodulin [Mus musculus]	0.944	1.063	0.722	0.880	0.801	1.248	0.698	0.775	0.705	0.859	0.782
568952742	XP_006508599.1#Shank2#210274# PREDICTED: SH3 and multiple ankyrin repeat domains protein 2 isoform X15 [Mus musculus]	1.076	0.834	0.776	0.827	0.801	1.248	0.821	0.903	0.664	0.774	0.719
754170335	NP_001291503.1#Arf2#11841# ADP-ribosylation factor 2 [Mus musculus]	0.909	0.853	0.711	0.891	0.801	1.248	0.668	0.721	0.492	0.710	0.601
7242142	NP_036133.1#Cops7a#26894# COP9 signalosome complex subunit 7a isoform 1 [Mus musculus]	1.042	0.888	0.697	0.906	0.801	1.248	0.846	0.823	0.673	0.809	0.741
568997209	XP_006522937.1#App#11820# PREDICTED: amyloid beta A4 protein isoform X2 [Mus musculus]	1.090	0.801	0.742	0.861	0.801	1.248	0.866	0.991	0.706	0.800	0.753
31982030	NP_598557.3#Arhgdia#192662# rho GDP-dissociation inhibitor 1 [Mus musculus]	0.963	0.910	0.725	0.878	0.801	1.248	0.695	0.846	0.637	0.780	0.709
755504615	XP_011238569.1#Rprd2#75137# PREDICTED: regulation of nuclear pre-mRNA domain-containing protein 2 isoform X3 [Mus musculus]	1.429	0.897	0.724	0.879	0.802	1.247	1.119	1.173	0.957	1.098	1.027
33468999	NP_081241.1#Comtd1#69156# catechol O-methyltransferase domain-containing protein 1 [Mus musculus]	1.101	0.960	0.672	0.932	0.802	1.247	0.909	1.018	0.782	1.102	0.942
31559995	NP_598417.2#Crk#12928# adapter molecule crk isoform 2 [Mus musculus]	1.021	0.834	0.676	0.929	0.802	1.247	0.773	0.873	0.603	0.769	0.686
52353955	NP_058662.2#Phgdh#236539# D-3-phosphoglycerate dehydrogenase [Mus musculus]	1.173	0.904	0.755	0.850	0.802	1.247	0.884	1.039	0.779	0.885	0.832
34328236	NP_061268.2#Ubqln2#54609# ubiquilin-2 [Mus musculus]	1.129	0.940	0.698	0.907	0.803	1.246	0.714	0.947	0.694	0.801	0.747
568980730	XP_006516455.1#Dlst#78920# PREDICTED: dihydrolipoyllysine-residue succinyltransferase component of 2-oxoglutarate dehydrogenase complex, mit	0.940	0.906	0.700	0.905	0.803	1.246	0.672	0.796	0.577	0.763	0.670
355390341	NP_001239035.1#Cadps2#320405# calcium-dependent secretion activator 2 isoform 3 [Mus musculus]	1.173	0.920	0.735	0.870	0.803	1.246	0.804	1.079	0.782	0.951	0.866
568918896	XP_006500478.1#Eif3j1#78655# PREDICTED: eukaryotic translation initiation factor 3 subunit J isoform X1 [Mus musculus]	1.092	0.993	0.784	0.822	0.803	1.246	0.808	0.945	0.797	0.944	0.870
6680924	NP_031713.1#Cfl1#12631# cofilin-1 [Mus musculus]	1.104	0.856	0.718	0.888	0.803	1.246	0.827	0.951	0.643	0.872	0.757
754170607	NP_001291648.1#Atp5f1#11950# ATP synthase F(0) complex subunit B1, mitochondrial isoform 2 [Mus musculus]	1.011	0.905	0.723	0.883	0.803	1.246	0.737	0.870	0.638	0.793	0.715
31982856	NP_031887.2#Dld#13382# dihydrolipoyl dehydrogenase, mitochondrial precursor [Mus musculus]	1.057	0.934	0.728	0.878	0.803	1.245	0.792	0.871	0.666	0.852	0.759
568975478	XP_006534116.1#Vps53#68299# PREDICTED: vacuolar protein sorting-associated protein 53 homolog isoform X2 [Mus musculus]	1.130	0.918	0.695	0.911	0.803	1.245	0.818	0.997	0.718	0.948	0.833
568933539	XP_006503687.1#Mapre3#100732# PREDICTED: microtubule-associated protein RP/EB family member 3 isoform X2 [Mus musculus]	1.119	0.863	0.721	0.886	0.804	1.245	0.872	0.977	0.674	0.891	0.783
113866024	NP_077776.2#Rab5c#19345# ras-related protein Rab-5C isoform 1 [Mus musculus]	1.175	0.892	0.712	0.895	0.804	1.244	0.930	1.053	0.732	0.964	0.848
568971596	XP_006532259.1#Gabra1#14394# PREDICTED: gamma-aminobutyric acid receptor subunit alpha-1 isoform X1 [Mus musculus]	1.022	0.846	0.673	0.934	0.804	1.244	0.721	0.922	0.578	0.811	0.695
568942612	XP_006506544.1#Chmp3#66700# PREDICTED: charged multivesicular body protein 3 isoform X3 [Mus musculus]	1.163	0.906	0.776	0.831	0.804	1.244	0.821	1.049	0.731	0.963	0.847
226874869	NP_062282.2#Omg#18377# oligodendrocyte-myelin glycoprotein precursor [Mus musculus]	1.352	0.967	0.698	0.910	0.804	1.244	0.940	1.208	0.899	1.229	1.064
112817607	NP_083424.1#Tmx4#52837# thioredoxin-related transmembrane protein 4 precursor [Mus musculus]	1.087	0.915	0.673	0.934	0.804	1.244	0.731	1.017	0.643	0.956	0.800
13384870	NP_079736.1#Tmem126a#66271# transmembrane protein 126A [Mus musculus]	1.172	0.865	0.708	0.900	0.804	1.244	0.787	1.098	0.679	0.952	0.815
110347469	NP_031402.3#Pzp#11287# alpha-2-macroglobulin precursor [Mus musculus]	1.171	0.938	0.728	0.880	0.804	1.244	0.821	1.029	0.745	1.002	0.873
6753364	NP_033991.1#Cdc42#12540# cell division control protein 42 homolog isoform 1 precursor [Mus musculus]	1.187	0.933	0.704	0.904	0.804	1.244	0.991	1.076	0.833	1.090	0.962
228480253	NP_001153190.1#Sept2#18000# septin-2 isoform b [Mus musculus]	1.044	0.898	0.713	0.895	0.804	1.244	0.735	0.927	0.646	0.869	0.757
6754724	NP_034947.1#Psmd7#17463# 26S proteasome non-ATPase regulatory subunit 7 [Mus musculus]	1.008	0.933	0.760	0.848	0.804	1.244	0.878	0.737	0.690	0.774	0.732
550544190	NP_001272385.1#Mapt#17762# microtubule-associated protein tau isoform e [Mus musculus]	1.178	0.887	0.713	0.895	0.804	1.243	0.885	1.052	0.755	0.941	0.848
6754022	NP_034446.1#Gng3#14704# guanine nucleotide-binding protein G(I)/G(S)/G(O) subunit gamma-3 precursor [Mus musculus]	1.059	0.925	0.688	0.921	0.804	1.243	0.854	0.850	0.645	0.932	0.788
294610683	NP_001009949.3#Slc25a51#230125# solute carrier family 25 member 51 [Mus musculus]	1.129	0.861	0.710	0.899	0.804	1.243	0.704	1.114	0.699	0.865	0.782
27369748	NP_766120.1#Aldh5a1#214579# succinate-semialdehyde dehydrogenase, mitochondrial precursor [Mus musculus]	1.147	1.003	0.686	0.923	0.804	1.243	0.854	1.076	0.786	1.108	0.947
568930415	XP_006538530.1#Atad3a#108888# PREDICTED: ATPase family AAA domain-containing protein 3 isoform X1 [Mus musculus]	1.107	0.854	0.730	0.879	0.805	1.243	0.816	0.940	0.669	0.782	0.725
27229277	NP_149065.2#Tars#110960# threonine--tRNA ligase, cytoplasmic [Mus musculus]	1.089	0.858	0.761	0.850	0.805	1.242	0.882	0.871	0.734	0.771	0.752
134031976	NP_082509.2#Lrpprc#72416# leucine-rich PPR motif-containing protein, mitochondrial precursor [Mus musculus]	1.159	0.926	0.737	0.874	0.805	1.242	1.024	1.162	0.881	1.188	1.034
755530077	XP_006511157.2#Coro2b#235431# PREDICTED: coronin-2B isoform X1 [Mus musculus]	1.201	0.836	0.725	0.886	0.805	1.242	0.883	1.024	0.615	0.924	0.770
124494256	NP_032538.2#Lrp1#16971# prolow-density lipoprotein receptor-related protein 1 precursor [Mus musculus]	1.223	0.883	0.712	0.899	0.805	1.241	0.900	1.133	0.712	0.928	0.820
161086984	NP_941015.2#Ap2s1#232910# AP-2 complex subunit sigma [Mus musculus]	1.046	0.826	0.692	0.919	0.806	1.241	0.826	0.972	0.597	0.830	0.713
255759890	NP_001157572.1#Add3#27360# gamma-adducin isoform b [Mus musculus]	1.000	0.866	0.687	0.924	0.806	1.241	0.734	0.875	0.597	0.803	0.700
114326546	NP_075907.2#Pgam1#18648# phosphoglycerate mutase 1 [Mus musculus]	0.979	0.935	0.718	0.893	0.806	1.241	0.738	0.889	0.673	0.885	0.779
568981954	XP_006516799.1#Bphl#68021# PREDICTED: valacyclovir hydrolase isoform X1 [Mus musculus]	1.204	0.899	0.817	0.794	0.806	1.241	0.890	1.050	0.769	0.976	0.872
22219434	NP_671734.1#Nrbp1#192292# nuclear receptor-binding protein [Mus musculus]	1.106	0.868	0.747	0.864	0.806	1.241	0.829	0.959	0.646	0.949	0.797
96975138	NP_038584.2#Hprt#15452# hypoxanthine-guanine phosphoribosyltransferase [Mus musculus]	0.989	0.951	0.713	0.899	0.806	1.241	0.728	0.855	0.645	0.874	0.760
569007769	XP_006527383.1#Hnrnpul2#68693# PREDICTED: heterogeneous nuclear ribonucleoprotein U-like protein 2 isoform X1 [Mus musculus]	1.305	0.787	0.700	0.911	0.806	1.241	0.993	1.207	0.729	0.977	0.853
51491845	NP_001003908.1#Cltc#67300# clathrin heavy chain 1 [Mus musculus]	1.291	0.856	0.713	0.899	0.806	1.241	0.936	1.139	0.761	1.008	0.884
254553458	NP_032181.2#Gpi1#14751# glucose-6-phosphate isomerase [Mus musculus]	0.950	0.925	0.720	0.892	0.806	1.241	0.685	0.818	0.621	0.816	0.718
755519373	XP_011248807.1#Shisa7#232813# PREDICTED: protein shisa-7 isoform X3 [Mus musculus]	1.031	0.794	0.688	0.924	0.806	1.240	0.723	0.939	0.562	0.758	0.660
568922992	XP_006501641.1#Negr1#320840# PREDICTED: neuronal growth regulator 1 isoform X3 [Mus musculus]	0.965	0.821	0.706	0.907	0.806	1.240	0.727	0.841	0.586	0.754	0.670
163644277	NP_031485.3#Ap2a2#11772# AP-2 complex subunit alpha-2 [Mus musculus]	1.172	0.839	0.733	0.880	0.806	1.240	0.868	1.032	0.719	0.904	0.811

568978916	XP_006515585.1#Mark3#17169# PREDICTED: MAP/microtubule affinity-regulating kinase 3 isoform X12 [Mus musculus]	0.927	0.799	0.653	0.960	0.807	1.240	0.653	0.822	0.483	0.729	0.606
27883844	NP_033253.1#Snrpe#20643# small nuclear ribonucleoprotein E [Mus musculus]	0.946	0.876	0.736	0.877	0.807	1.240	0.727	0.798	0.595	0.741	0.668
17298686	NP_473438.1#Pip4k2c#117150# phosphatidylinositol 5-phosphate 4-kinase type-2 gamma [Mus musculus]	1.098	0.814	0.706	0.907	0.807	1.240	0.800	0.971	0.613	0.829	0.721
125346154	NP_001074927.1#Ppp2r5c#26931# serine/threonine-protein phosphatase 2A 56 kDa regulatory subunit gamma isoform isoform c [Mus musculus]	0.782	0.894	0.700	0.913	0.807	1.240	0.572	0.689	0.489	0.639	0.564
26986569	NP_758475.1#Slc6a17#229706# sodium-dependent neutral amino acid transporter SLC6A17 isoform2 [Mus musculus]	1.121	0.837	0.670	0.943	0.807	1.239	0.798	1.013	0.623	0.889	0.756
569009783	XP_006527922.1#L1cam#16728# PREDICTED: neural cell adhesion molecule L1 isoform X1 [Mus musculus]	1.112	0.910	0.735	0.879	0.807	1.239	0.775	0.914	0.707	0.890	0.799
568914422	XP_006498454.1#Arpc5l#74192# PREDICTED: actin-related protein 2/3 complex subunit 5-like protein isoform X2 [Mus musculus]	1.104	0.829	0.755	0.860	0.807	1.239	0.855	0.978	0.621	0.881	0.751
227908823	NP_080471.2#Atic#108147# bifunctional purine biosynthesis protein PURH [Mus musculus]	1.052	0.923	0.709	0.906	0.807	1.239	0.779	0.919	0.637	0.933	0.785
189339262	NP_079770.2#Atp6v1c1#66335# V-type proton ATPase subunit C 1 [Mus musculus]	1.578	0.837	0.711	0.904	0.807	1.238	1.147	1.422	0.877	1.151	1.014
568926120	XP_006537702.1#Alad#17025# PREDICTED: delta-aminolevulinic acid dehydratase isoform X1 [Mus musculus]	1.204	0.879	0.710	0.905	0.807	1.238	0.885	1.060	0.757	0.952	0.855
56711244	NP_035018.1#Ndufs6#407785# NADH dehydrogenase [ubiquinone] iron-sulfur protein 6, mitochondrial precursor [Mus musculus]	1.368	0.914	0.729	0.886	0.807	1.238	0.857	1.088	0.745	1.100	0.923
568999125	XP_006523772.1#Hdgfrp2#15193# PREDICTED: hepatoma-derived growth factor-related protein 2 isoform X5 [Mus musculus]	0.910	1.114	0.716	0.899	0.808	1.238	0.724	0.745	0.795	0.842	0.819
21311873	NP_077181.1#Hsbp1#68196# heat shock factor-binding protein 1 [Mus musculus]	1.349	0.782	0.724	0.892	0.808	1.238	0.803	1.125	0.711	1.085	0.898
357527382	NP_001239484.1#Gstz1#14874# maleylacetoacetate isomerase isoform 2 [Mus musculus]	1.068	0.897	0.708	0.907	0.808	1.238	0.745	0.981	0.680	0.909	0.795
568909823	XP_006530014.1#Shisa4#77552# PREDICTED: protein shisa-4 isoform X1 [Mus musculus]	1.051	0.854	0.749	0.867	0.808	1.238	0.779	0.920	0.604	0.846	0.725
755553282	XP_011244152.1#Stxbp5l#207227# PREDICTED: syntaxin-binding protein 5-like isoform X4 [Mus musculus]	1.642	0.815	0.769	0.847	0.808	1.238	1.168	1.485	0.881	1.282	1.081
755563901	XP_011245712.1#Sfxn4#94281# PREDICTED: sideroflexin-4 isoform X4 [Mus musculus]	1.071	0.845	0.699	0.917	0.808	1.238	0.738	0.990	0.640	0.822	0.731
6679082	NP_032733.1#Nmt1#18107# glycylpeptide N-tetradecanoyltransferase 1 [Mus musculus]	1.172	0.911	0.732	0.884	0.808	1.237	0.915	0.979	0.743	0.983	0.863
755530267	XP_011241071.1#Tmod2#50876# PREDICTED: tropomodulin-2 isoform X1 [Mus musculus]	1.067	0.901	0.729	0.887	0.808	1.237	0.728	0.862	0.622	0.843	0.732
13386026	NP_080804.1#2700060E02Rik#68045# UPF0568 protein C14orf166 homolog [Mus musculus]	1.154	0.921	0.729	0.888	0.808	1.237	0.785	1.023	0.732	1.021	0.876
120537241	NP_598898.2#Lars#107045# leucine--tRNA ligase, cytoplasmic [Mus musculus]	1.057	0.839	0.707	0.910	0.808	1.237	0.790	0.919	0.633	0.801	0.717
568936138	XP_006535247.1#Paics#67054# PREDICTED: multifunctional protein ADE2 isoform X2 [Mus musculus]	0.980	0.917	0.690	0.927	0.808	1.237	0.751	0.833	0.620	0.833	0.727
755550334	XP_011243654.1#Ywhaz#22631# PREDICTED: 14-3-3 protein zeta/delta isoform X1 [Mus musculus]	1.074	0.843	0.715	0.902	0.808	1.237	0.799	0.925	0.589	0.841	0.715
33598964	NP_780469.1#Myh10#77579# myosin-10 [Mus musculus]	1.096	0.904	0.733	0.884	0.808	1.237	0.814	0.972	0.726	0.965	0.845
568931876	XP_006539230.1#Acot7#70025# PREDICTED: cytosolic acyl coenzyme A thioester hydrolase isoform X2 [Mus musculus]	0.967	0.963	0.732	0.885	0.809	1.237	0.717	0.830	0.643	0.882	0.762
226958349	NP_033441.2#Tpi1#21991# triosephosphate isomerase [Mus musculus]	1.078	0.946	0.737	0.880	0.809	1.237	0.797	0.945	0.708	0.909	0.809
21312062	NP_081051.1#Tmed10#68581# transmembrane emp24 domain-containing protein 10 precursor [Mus musculus]	1.042	0.914	0.711	0.907	0.809	1.236	0.861	0.975	0.667	0.950	0.809
568905907	XP_006495820.1#Map2#17756# PREDICTED: microtubule-associated protein 2 isoform X5 [Mus musculus]	1.100	0.855	0.709	0.909	0.809	1.236	0.825	0.969	0.648	0.882	0.765
226443015	NP_034303.3#Faah#14073# fatty-acid amide hydrolase 1 [Mus musculus]	1.222	0.807	0.681	0.938	0.809	1.236	0.823	1.153	0.704	0.892	0.798
569009695	XP_006527878.1#Fmr1#14265# PREDICTED: fragile X mental retardation protein 1 homolog isoform X5 [Mus musculus]	1.087	0.888	0.679	0.940	0.809	1.236	0.781	0.978	0.669	0.893	0.781
569016865	XP_006535429.1#Crip2#68337# PREDICTED: cysteine-rich protein 2 isoform X1 [Mus musculus]	0.964	0.897	0.720	0.899	0.810	1.235	0.812	0.748	0.562	0.837	0.700
268370057	NP_077222.4#Ccdc132#73288# coiled-coil domain-containing protein 132 isoform 1 [Mus musculus]	1.218	0.927	0.744	0.875	0.810	1.235	0.902	1.070	0.826	1.001	0.914
755516379	XP_011239564.1#Exoc4#20336# PREDICTED: exocyst complex component 4 isoform X1 [Mus musculus]	1.264	0.902	0.749	0.870	0.810	1.235	0.901	1.146	0.724	1.122	0.923
568902472	XP_006543439.1#LOC100862455#100862455# PREDICTED: 60S ribosomal protein L23 isoform X1 [Mus musculus]	1.134	0.887	0.704	0.916	0.810	1.235	0.884	1.016	0.700	0.914	0.807
568974918	XP_006533846.1#Srcin1#56013# PREDICTED: SRC kinase signaling inhibitor 1 isoform X22 [Mus musculus]	1.041	0.859	0.716	0.904	0.810	1.235	0.770	0.927	0.655	0.835	0.745
7106387	NP_036097.1#Psma5#26442# proteasome subunit alpha type-5 [Mus musculus]	0.916	0.728	0.733	0.887	0.810	1.234	0.697	0.780	0.486	0.625	0.555
257467604	NP_780589.3#Gpd1l#333433# glycerol-3-phosphate dehydrogenase 1-like protein [Mus musculus]	1.240	0.849	0.721	0.899	0.810	1.234	0.960	1.097	0.675	1.046	0.861
755553155	XP_011244125.1#Dlg1#13383# PREDICTED: disks large homolog 1 isoform X14 [Mus musculus]	1.226	0.805	0.735	0.886	0.810	1.234	0.917	1.121	0.663	0.947	0.805
226874906	NP_033562.3#Ywhae#22627# 14-3-3 protein epsilon [Mus musculus]	0.957	0.883	0.707	0.914	0.810	1.234	0.716	0.837	0.593	0.779	0.686
6680720	NP_031505.1#Arf4#11843# ADP-ribosylation factor 4 [Mus musculus]	1.123	0.920	0.759	0.861	0.810	1.234	0.775	1.048	0.662	0.994	0.828
569001363	XP_006524859.1#Ubxn6#66530# PREDICTED: UBX domain-containing protein 6 isoform X1 [Mus musculus]	1.395	1.022	0.707	0.913	0.810	1.234	1.030	1.231	0.957	1.354	1.156
13385726	NP_080495.1#Uqcrb#67530# cytochrome b-c1 complex subunit 7 [Mus musculus]	1.095	0.954	0.714	0.907	0.810	1.234	0.828	1.032	0.754	1.003	0.878
73695877	NP_808430.2#Aak1#269774# AP2 associated kinase 1 isoform 2 [Mus musculus]	1.264	0.793	0.730	0.892	0.811	1.233	0.938	1.060	0.667	0.925	0.796
568969997	XP_006514639.1#Nipsnap1#18082# PREDICTED: protein NipSnap homolog 1 isoform X2 [Mus musculus]	1.088	0.921	0.703	0.919	0.811	1.233	0.811	0.969	0.692	0.999	0.846
568907852	XP_006496636.1#Mff#75734# PREDICTED: mitochondrial fission factor isoform X17 [Mus musculus]	0.890	0.959	0.665	0.957	0.811	1.233	0.658	0.785	0.598	0.786	0.692
27228985	NP_079827.2#Ndufa12#66414# NADH dehydrogenase [ubiquinone] 1 alpha subcomplex subunit 12 [Mus musculus]	1.023	0.906	0.721	0.900	0.811	1.233	0.794	0.865	0.639	0.961	0.800
568966729	XP_006513310.1#Hk1#15275# PREDICTED: hexokinase-1 isoform X4 [Mus musculus]	1.147	0.826	0.715	0.906	0.811	1.233	0.870	1.038	0.696	0.884	0.790
568911768	XP_006497257.1#Marc2#67247# PREDICTED: mitochondrial amidoxime reducing component 2 isoform X1 [Mus musculus]	1.482	0.788	0.754	0.868	0.811	1.233	1.075	1.329	0.783	1.113	0.948
597517929	NP_001278006.1#Snph#241727# syntaphilin isoform c [Mus musculus]	1.026	0.866	0.746	0.876	0.811	1.233	0.736	0.928	0.585	0.856	0.720
27597069	NP_775482.1#Prune#229589# protein prune homolog [Mus musculus]	1.244	0.873	0.750	0.873	0.811	1.233	0.885	1.134	0.756	1.007	0.882
218156282	NP_892038.2#Slc17a7#72961# vesicular glutamate transporter 1 [Mus musculus]	1.086	0.830	0.695	0.928	0.811	1.232	0.754	0.910	0.678	0.844	0.761
568921574	XP_006500959.1#Ank2#109676# PREDICTED: ankyrin-2 isoform X25 [Mus musculus]	1.215	0.898	0.712	0.912	0.812	1.232	0.859	1.060	0.764	1.010	0.887
568942798	XP_006506635.1#Scrn1#69938# PREDICTED: secernin-1 isoform X1 [Mus musculus]	1.342	0.812	0.727	0.896	0.812	1.232	0.999	1.183	0.738	1.031	0.884
755561255	XP_011245300.1#Afg3l2#69597# PREDICTED: AFG3-like protein 2 isoform X1 [Mus musculus]	1.176	0.854	0.688	0.936	0.812	1.232	0.844	1.034	0.684	0.826	0.755
242332525	NP_082171.1#Ulk3#71742# serine/threonine-protein kinase ULK3 [Mus musculus]	1.129	0.840	0.693	0.931	0.812	1.232	0.873	0.960	0.619	0.920	0.770
30519997	NP_848760.1#Blmh#104184# bleomycin hydrolase [Mus musculus]	1.124	0.874	0.712	0.912	0.812	1.232	0.827	0.999	0.669	0.926	0.798
21313640	NP_082191.1#Ap2b1#71770# AP-2 complex subunit beta isoform b [Mus musculus]	1.177	0.852	0.694	0.930	0.812	1.232	0.865	1.035	0.688	0.937	0.812
28076897	NP_080914.1#Cdipt#52858# CDP-diacylglycerol--inositol 3-phosphatidyltransferase [Mus musculus]	1.002	0.887	0.692	0.932	0.812	1.231	0.759	0.873	0.674	0.836	0.755
37718983	NP_937806.1#Rab35#77407# ras-related protein Rab-35 [Mus musculus]	1.236	0.855	0.742	0.882	0.812	1.231	0.759	1.081	0.755	0.874	0.814
15011853	NP_058081.2#Stx1a#20907# syntaxin-1A [Mus musculus]	1.174	0.809	0.684	0.940	0.812	1.231	0.845	0.993	0.652	0.831	0.741
568922111	XP_006501215.1#Abcd3#19299# PREDICTED: ATP-binding cassette sub-family D member 3 isoform X1 [Mus musculus]	1.072	1.008	0.720	0.904	0.812	1.231	0.811	0.931	0.748	1.007	0.878
40254577	NP_035425.2#Rps12#20042# 40S ribosomal protein S12 [Mus musculus]	1.319	0.865	0.711	0.913	0.812	1.231	0.978	1.160	0.834	1.020	0.927
34787412	NP_038914.2#Hdgfrp3#29877# hepatoma-derived growth factor-related protein 3 [Mus musculus]	1.152	0.923	0.690	0.935	0.812	1.231	0.841	1.031	0.702	1.024	0.863
12963495	NP_075611.1#Gpm6b#14758# neuronal membrane glycoprotein M6-b isoform 4 [Mus musculus]	1.189	0.825	0.712	0.913	0.812	1.231	0.881	1.071	0.623	0.832	0.728
31560792	NP_062408.2#Sh3gl2#20404# endophilin-A1 [Mus musculus]	1.084	0.814	0.705	0.920	0.813	1.231	0.807	0.932	0.607	0.813	0.710
568995482	XP_006522265.1#Lsamp#268890# PREDICTED: limbic system-associated membrane protein isoform X2 [Mus musculus]	1.027	0.852	0.734	0.891	0.813	1.231	0.748	0.879	0.597	0.844	0.721
6677809	NP_033122.1#Rps6#20104# 40S ribosomal protein S6 [Mus musculus]	1.045	0.924	0.685	0.940	0.813	1.231	0.774	0.925	0.687	0.883	0.785
568978634	XP_006515450.1#Actn1#109711# PREDICTED: alpha-actinin-1 isoform X4 [Mus musculus]	1.156	0.798	0.720	0.905	0.813	1.230	0.905	1.071	0.652	0.860	0.756
6680309	NP_032329.1#Hspe1#15528# 10 kDa heat shock protein, mitochondrial [Mus musculus]	1.065	0.970	0.710	0.915	0.813	1.230	0.751	0.952	0.672	0.921	0.797
19526960	NP_598513.1#Opa1#74143# dynamin-like 120 kDa protein, mitochondrial isoform 2 precursor [Mus musculus]	1.238	0.888	0.710	0.916	0.813	1.230	0.933	1.134	0.775	1.063	0.919
254675232	NP_081611.1#2210016F16Rik#70153# UPF0553 protein C9orf64 homolog [Mus musculus]	1.343	0.886	0.755	0.871	0.813	1.230	0.985	1.200	0.870	1.065	0.967
568945146	XP_006539743.1#Rps19#20085# PREDICTED: 40S ribosomal protein S19 isoform X1 [Mus musculus]	0.957	0.934	0.725	0.902	0.813	1.230	0.748	0.881	0.643	0.831	0.737
568908602	XP_006529424.1#Cntn2#21367# PREDICTED: contactin-2 isoform X1 [Mus musculus]	1.153	0.853	0.773	0.853	0.813	1.230	0.846	1.030	0.721	0.878	0.800
31542143	NP_058082.2#Rhoa#11848# transforming protein RhoA precursor [Mus musculus]	1.434	0.820	0.749	0.878	0.813	1.229	1.124	1.163	0.839	0.975	0.907
6754976	NP_035164.1#Prdx1#18477# peroxiredoxin-1 [Mus musculus]	0.929	0.902	0.679	0.948	0.813	1.229	0.657	0.823	0.608	0.819	0.714
6753964	NP_034397.1#Gdap1#14545# ganglioside-induced differentiation-associated protein 1 [Mus musculus]	1.140	0.967	0.769	0.858	0.813	1.229	0.820	1.034	0.820	0.973	0.897
24762230	NP_733769.1#Rps15a#267019# 40S ribosomal protein S15a [Mus musculus]	1.253	0.891	0.732	0.895	0.814	1.229	0.943	1.059	0.792	0.991	0.891
569016358	XP_006497365.1#Farsb#23874# PREDICTED: phenylalanine--tRNA ligase beta subunit isoform X1 [Mus musculus]	1.218	0.867	0.704	0.923	0.814	1.229	0.869	1.113	0.712	1.007	0.860
283806722	NP_001164339.1#Ncald#52589# neurocalcin-delta [Mus musculus]	**2.197**	0.812	0.716	0.911	0.814	1.229	1.718	2.193	1.342	1.763	1.552
755498625	XP_011237574.1#Csnk2a1#12995# PREDICTED: casein kinase II subunit alpha isoform X1 [Mus musculus]	1.079	0.910	0.705	0.922	0.814	1.229	0.813	0.984	0.714	0.926	0.820
755529755	XP_011240968.1#Me1#17436# PREDICTED: NADP-dependent malic enzyme isoform X1 [Mus musculus]	1.142	0.866	0.710	0.918	0.814	1.229	0.825	0.985	0.696	0.914	0.805
649572215	NP_001280488.1#Cox4i1#12857# cytochrome c oxidase subunit 4 isoform 1, mitochondrial isoform 2 [Mus musculus]	1.156	0.973	0.693	0.935	0.814	1.229	0.847	1.035	0.844	1.086	0.965
6680690	NP_031478.1#Prdx3#11757# thioredoxin-dependent peroxide reductase, mitochondrial precursor [Mus musculus]	1.008	0.968	0.716	0.911	0.814	1.229	0.754	0.898	0.672	0.928	0.800

21314824	NP_079657.1#Atp6v1f#66144# V-type proton ATPase subunit F [Mus musculus]	1.063	0.875	0.733	0.894	0.814	1.229	0.743	0.949	0.650	0.854	0.752
255958286	NP_063932.2#Suclg1#56451# succinyl-CoA ligase [ADP/GDP-forming] subunit alpha, mitochondrial precursor [Mus musculus]	1.222	0.968	0.736	0.892	0.814	1.228	0.927	1.054	0.793	1.074	0.934
594190942	NP_001277398.1#Atp1a3#232975# sodium/potassium-transporting ATPase subunit alpha-3 [Mus musculus]	1.236	0.987	0.713	0.915	0.814	1.228	0.941	1.146	0.892	1.148	1.020
38490690	NP_941055.1#Iars2#381314# isoleucine--tRNA ligase, mitochondrial precursor [Mus musculus]	1.284	0.863	0.723	0.905	0.814	1.228	0.916	1.174	0.776	1.029	0.903
594542446	NP_613071.3#Ppp1r3f#54646# protein phosphatase 1 regulatory subunit 3F isoform 2 [Mus musculus]	0.945	0.867	0.699	0.930	0.814	1.228	0.755	0.785	0.532	0.802	0.667
33563266	NP_035016.1#Ndufa4#17992# cytochrome c oxidase subunit NDUFA4 [Mus musculus]	1.024	0.933	0.742	0.887	0.815	1.228	0.712	0.894	0.669	0.831	0.750
6754036	NP_034455.1#Got2#14719# aspartate aminotransferase, mitochondrial [Mus musculus]	1.167	0.925	0.725	0.904	0.815	1.228	0.892	1.015	0.774	0.982	0.878
31559891	NP_067511.4#Rhot1#59040# mitochondrial Rho GTPase 1 isoform 1 [Mus musculus]	1.045	0.897	0.743	0.886	0.815	1.228	0.771	0.835	0.666	0.841	0.753
569008135	XP_006527556.1#Sfxn3#94280# PREDICTED: sideroflexin-3 isoform X1 [Mus musculus]	1.070	0.835	0.699	0.931	0.815	1.228	0.827	1.040	0.704	0.884	0.794
568990382	XP_006520096.1#Ctnnd2#18163# PREDICTED: catenin delta-2 isoform X2 [Mus musculus]	1.038	0.870	0.706	0.923	0.815	1.227	0.784	0.910	0.646	0.826	0.736
6753492	NP_034028.1#Coro1a#12721# coronin-1A isoform 1 [Mus musculus]	1.166	0.829	0.715	0.914	0.815	1.227	0.845	1.027	0.692	0.894	0.793
554506543	NP_001273009.1#Nin#18080# ninein isoform 3 [Mus musculus]	1.012	0.878	0.733	0.896	0.815	1.227	0.745	0.904	0.609	0.840	0.724
124486895	NP_001074743.1#Pgd#110208# 6-phosphogluconate dehydrogenase, decarboxylating [Mus musculus]	1.130	0.980	0.719	0.910	0.815	1.227	0.897	1.053	0.791	1.033	0.912
229892320	NP_001153511.1#Ndufs1#227197# NADH-ubiquinone oxidoreductase 75 kDa subunit, mitochondrial precursor [Mus musculus]	1.318	0.933	0.729	0.901	0.815	1.227	0.964	1.176	0.891	1.113	1.002
257153390	NP_001158057.1#Dnajc6#72685# putative tyrosine-protein phosphatase auxilin isoform c [Mus musculus]	1.179	0.881	0.768	0.862	0.815	1.227	0.911	1.066	0.748	0.995	0.872
755530455	XP_011241115.1#Tln2#70549# PREDICTED: talin-2 isoform X4 [Mus musculus]	1.128	0.825	0.713	0.917	0.815	1.227	0.901	1.045	0.708	0.931	0.819
51511741	NP_001003933.1#Rtn3#20168# reticulon-3 isoform 2 [Mus musculus]	1.237	0.857	0.726	0.905	0.815	1.227	0.902	1.004	0.691	0.881	0.786
755561214	XP_011245294.1#Ccny#67974# PREDICTED: cyclin-Y isoform X2 [Mus musculus]	0.971	0.924	0.776	0.854	0.815	1.227	0.723	0.859	0.644	0.817	0.731
568974067	XP_006533435.1#Map2k4#26398# PREDICTED: dual specificity mitogen-activated protein kinase kinase 4 isoform X3 [Mus musculus]	1.180	0.883	0.715	0.916	0.816	1.226	0.768	0.985	0.687	0.926	0.806
256773218	NP_001038980.1#Synj1#104015# synaptojanin-1 isoform b [Mus musculus]	1.104	0.873	0.716	0.916	0.816	1.226	0.826	1.011	0.690	0.902	0.796
547235396	NP_001271257.1#Syncrip#56403# heterogeneous nuclear ribonucleoprotein Q isoform 3 [Mus musculus]	0.916	0.852	0.689	0.943	0.816	1.226	0.683	0.891	0.557	0.796	0.676
568967742	XP_006513797.1#Vps26a#30930# PREDICTED: vacuolar protein sorting-associated protein 26A isoform X1 [Mus musculus]	0.897	0.846	0.691	0.941	0.816	1.226	0.671	0.791	0.517	0.719	0.618
6680954	NP_031753.1#Cntn1#12805# contactin-1 precursor [Mus musculus]	1.039	0.886	0.733	0.899	0.816	1.226	0.778	0.962	0.654	0.882	0.768
6679583	NP_033023.1#Rab11b#19326# ras-related protein Rab-11B [Mus musculus]	0.830	0.882	0.717	0.915	0.816	1.225	0.642	0.699	0.521	0.681	0.601
254540082	NP_032971.2#Psmb4#19172# proteasome subunit beta type-4 precursor [Mus musculus]	0.830	0.718	0.720	0.912	0.816	1.225	0.565	0.791	0.445	0.527	0.486
568960833	XP_006510918.1#Pkm#18746# PREDICTED: pyruvate kinase PKM isoform X1 [Mus musculus]	1.403	0.889	0.762	0.870	0.816	1.225	1.043	1.240	0.846	1.217	1.032
327180707	NP_001192117.1#Ppp2r2a#71978# serine/threonine-protein phosphatase 2A 55 kDa regulatory subunit B alpha isoform isoform 2 [Mus musculus]	0.979	0.894	0.701	0.931	0.816	1.225	0.775	0.954	0.639	0.861	0.750
74271899	NP_034435.1#Gnai1#14677# guanine nucleotide-binding protein G(i) subunit alpha-1 [Mus musculus]	0.986	0.834	0.718	0.915	0.816	1.225	0.772	0.872	0.628	0.780	0.704
469832253	NP_001264160.1#Crkl#12929# crk-like protein isoform 2 [Mus musculus]	0.881	0.935	0.745	0.888	0.816	1.225	0.697	0.741	0.542	0.803	0.672
755551082	XP_011243780.1#Oxr1#170719# PREDICTED: oxidation resistance protein 1 isoform X2 [Mus musculus]	1.109	0.916	0.683	0.950	0.816	1.225	0.786	1.025	0.709	0.951	0.830
283837783	NP_001025044.2#Unc13a#382018# protein unc-13 homolog A [Mus musculus]	1.370	0.852	0.787	0.846	0.816	1.225	1.030	1.208	0.792	1.117	0.954
181	NEDD8_HUMAN	0.993	0.877	0.735	0.898	0.816	1.225	0.766	0.855	0.622	0.800	0.711
313569763	NP_001186268.1#Apoo#68316# apolipoprotein O isoform 3 [Mus musculus]	0.849	1.025	0.703	0.930	0.817	1.225	0.606	0.781	0.692	0.728	0.710
6996911	NP_031520.1#Ass1#11898# argininosuccinate synthase [Mus musculus]	1.186	0.879	0.718	0.915	0.817	1.224	0.839	1.093	0.749	0.954	0.851
37693505	NP_937760.1#Abi2#329165# abl interactor 2 isoform 3 [Mus musculus]	1.047	0.864	0.728	0.905	0.817	1.224	0.744	0.924	0.629	0.789	0.709
227500281	NP_663590.3#Etfa#110842# electron transfer flavoprotein subunit alpha, mitochondrial [Mus musculus]	1.082	0.943	0.701	0.933	0.817	1.224	0.784	0.944	0.733	0.981	0.857
13385664	NP_080436.1#Map1lc3b#67443# microtubule-associated proteins 1A/1B light chain 3B [Mus musculus]	1.053	0.853	0.705	0.929	0.817	1.224	0.826	0.894	0.660	0.807	0.733
755492095	XP_011236737.1#Lancl1#14768# PREDICTED: lanC-like protein 1 isoform X2 [Mus musculus]	1.148	0.884	0.645	0.989	0.817	1.224	0.898	0.978	0.717	0.940	0.829
126723461	NP_033970.3#Cct8#12469# T-complex protein 1 subunit theta [Mus musculus]	1.105	0.910	0.687	0.947	0.817	1.224	0.810	0.989	0.711	0.953	0.832
19882201	NP_598862.1#Psmd2#21762# 26S proteasome non-ATPase regulatory subunit 2 [Mus musculus]	1.065	0.884	0.738	0.896	0.817	1.224	0.824	1.010	0.679	0.957	0.818
576583456	NP_001276671.1#Lrrc4c#241568# leucine-rich repeat-containing protein 4C precursor [Mus musculus]	1.068	0.823	0.684	0.950	0.817	1.224	0.841	0.904	0.620	0.817	0.718
208431800	NP_694774.3#Irgq#210146# immunity-related GTPase family Q protein [Mus musculus]	1.183	0.905	0.773	0.861	0.817	1.224	0.864	1.069	0.766	0.983	0.875
33563284	NP_036132.1#Cops6#26893# COP9 signalosome complex subunit 6 [Mus musculus]	1.151	0.882	0.705	0.930	0.817	1.224	0.873	1.012	0.699	0.964	0.831
569019357	XP_006537268.1#Slc25a11#67863# PREDICTED: mitochondrial 2-oxoglutarate/malate carrier protein isoform X1 [Mus musculus]	0.902	0.911	0.719	0.916	0.817	1.224	0.642	0.770	0.574	0.759	0.667
329299065	NP_001192296.1#Sept7#235072# septin-7 isoform 2 [Mus musculus]	1.026	0.821	0.725	0.909	0.817	1.224	0.830	1.025	0.640	0.845	0.742
226437629	NP_034460.3#Emb#13723# embigin precursor [Mus musculus]	1.118	0.943	0.738	0.897	0.817	1.223	0.848	0.981	0.768	0.955	0.862
6755224	NP_036006.1#Twf2#23999# twinfilin-2 [Mus musculus]	1.120	0.850	0.718	0.917	0.818	1.223	0.811	0.978	0.627	0.864	0.745
134053913	NP_542121.2#Psmd5#66998# 26S proteasome non-ATPase regulatory subunit 5 [Mus musculus]	0.982	0.983	0.725	0.910	0.818	1.223	0.743	0.859	0.684	0.894	0.789
568935106	XP_006534747.1#Rpl5#100503670# PREDICTED: 60S ribosomal protein L5 isoform X1 [Mus musculus]	1.181	0.913	0.768	0.867	0.818	1.223	0.874	1.057	0.709	1.055	0.882
568952448	XP_006536295.1#Slc25a22#68267# PREDICTED: mitochondrial glutamate carrier 1 isoform X1 [Mus musculus]	0.959	0.924	0.718	0.918	0.818	1.223	0.813	0.978	0.688	0.984	0.836
145301549	NP_001077424.1#Hba-a2#110257# hemoglobin alpha, adult chain 2 [Mus musculus]	1.129	0.924	0.720	0.916	0.818	1.222	0.824	1.000	0.777	0.981	0.879
18152793	NP_077183.1#Pdhb#68263# pyruvate dehydrogenase E1 component subunit beta, mitochondrial precursor [Mus musculus]	1.183	0.917	0.746	0.890	0.818	1.222	0.836	1.032	0.726	0.955	0.840
224809382	NP_080645.2#Arpc5#67771# actin-related protein 2/3 complex subunit 5 [Mus musculus]	0.990	0.851	0.723	0.913	0.818	1.222	0.753	0.853	0.619	0.818	0.718
568986366	XP_006518443.1#Phyhip#105653# PREDICTED: phytanoyl-CoA hydroxylase-interacting protein isoform X1 [Mus musculus]	1.489	0.715	0.746	0.890	0.818	1.222	1.026	1.317	0.719	0.958	0.839
31982273	NP_032318.2#Hsd17b4#15488# peroxisomal multifunctional enzyme type 2 [Mus musculus]	1.100	0.850	0.711	0.926	0.818	1.222	0.859	0.932	0.701	0.841	0.771
100818462	NP_663519.2#Rap1gds1#229877# rap1 GTPase-GDP dissociation stimulator 1 isoform b [Mus musculus]	1.144	0.859	0.697	0.940	0.818	1.222	0.842	0.997	0.694	0.956	0.825
6677713	NP_033073.1#Rem1#19700# GTP-binding protein REM 1 [Mus musculus]	1.776	0.843	0.730	0.906	0.818	1.222	1.381	1.526	0.989	1.463	1.226
6755963	NP_035824.1#Vdac1#22333# voltage-dependent anion-selective channel protein 1 [Mus musculus]	1.041	0.871	0.730	0.906	0.818	1.222	0.799	0.912	0.659	0.865	0.762
31980744	NP_038823.2#Atp5l#27425# ATP synthase subunit g, mitochondrial [Mus musculus]	0.963	0.958	0.762	0.875	0.818	1.222	0.734	0.818	0.688	0.843	0.765
148271067	NP_001087234.1#Myadm#50918# myeloid-associated differentiation marker [Mus musculus]	0.844	0.808	0.721	0.916	0.818	1.222	0.615	0.765	0.402	0.714	0.558
6755967	NP_035826.1#Vdac3#22335# voltage-dependent anion-selective channel protein 3 isoform 2 [Mus musculus]	0.929	0.915	0.709	0.928	0.818	1.222	0.734	0.866	0.607	0.772	0.690
254939522	NP_001157095.1#Prepl#213760# prolyl endopeptidase-like isoform b [Mus musculus]	1.200	0.856	0.770	0.867	0.819	1.222	0.856	1.112	0.706	0.978	0.842
51093840	NP_660121.2#Eif3l#223691# eukaryotic translation initiation factor 3 subunit L [Mus musculus]	0.850	0.857	0.719	0.918	0.819	1.221	0.638	0.779	0.520	0.664	0.592
568963563	XP_006512047.1#Plcd1#18799# PREDICTED: 1-phosphatidylinositol 4,5-bisphosphate phosphodiesterase delta-1 isoform X1 [Mus musculus]	0.871	0.994	0.754	0.883	0.819	1.221	0.652	0.774	0.596	0.821	0.709
281182473	NP_001161943.1#Dynll2#68097# dynein light chain 2, cytoplasmic [Mus musculus]	0.770	0.882	0.754	0.884	0.819	1.221	0.579	0.677	0.491	0.585	0.538
568967149	XP_006513512.1#Syt1#20979# PREDICTED: synaptotagmin-1 isoform X2 [Mus musculus]	1.050	0.797	0.727	0.911	0.819	1.221	0.791	0.937	0.590	0.760	0.675
84875526	NP_001034182.1#Tom1l2#216810# TOM1-like protein 2 isoform c [Mus musculus]	1.197	0.908	0.730	0.908	0.819	1.221	0.819	1.079	0.715	1.017	0.866
131412198	NP_032476.2#Klc1#16593# kinesin light chain 1 isoform 1A [Mus musculus]	1.135	0.877	0.691	0.947	0.819	1.221	0.894	0.967	0.711	0.920	0.815
12963799	NP_076210.1#Atp6v1d#73834# V-type proton ATPase subunit D [Mus musculus]	0.886	0.867	0.719	0.919	0.819	1.221	0.678	0.754	0.619	0.701	0.660
568987437	XP_006518960.1#Ccar2#219158# PREDICTED: cell cycle and apoptosis regulator protein 2 isoform X2 [Mus musculus]	1.284	0.927	0.762	0.877	0.819	1.220	0.981	1.123	0.831	1.119	0.975
28916677	NP_803126.1#Camk2a#12322# calcium/calmodulin-dependent protein kinase type II subunit alpha isoform 2 [Mus musculus]	1.048	0.780	0.722	0.917	0.820	1.220	0.790	0.973	0.613	0.793	0.703
568936990	XP_006530290.1#Rpl6#19988# PREDICTED: 60S ribosomal protein L6 isoform X1 [Mus musculus]	1.039	0.912	0.754	0.886	0.820	1.220	0.750	0.945	0.670	0.859	0.764
27370240	NP_766415.1#Lnpep#240028# leucyl-cystinyl aminopeptidase [Mus musculus]	1.008	0.914	0.654	0.985	0.820	1.220	0.745	0.908	0.643	0.868	0.756
21704122	NP_663605.1#Pdk3#236900# pyruvate dehydrogenase kinase, isozyme 3 [Mus musculus]	1.133	0.898	0.765	0.874	0.820	1.220	0.899	0.959	0.695	0.974	0.834
58037109	NP_080960.1#Ndufb10#68342# NADH dehydrogenase [ubiquinone] 1 beta subcomplex subunit 10 [Mus musculus]	1.192	0.944	0.707	0.932	0.820	1.220	0.799	1.050	0.789	1.002	0.896
568966266	XP_006513094.1#Lin7a#108030# PREDICTED: protein lin-7 homolog A isoform X1 [Mus musculus]	1.458	0.873	0.723	0.917	0.820	1.220	1.109	1.285	0.891	1.199	1.045
9256519	NP_061341.1#Rplp1#56040# 60S acidic ribosomal protein P1 [Mus musculus]	1.237	0.887	0.728	0.912	0.820	1.220	0.950	1.102	0.694	1.025	0.859
163644296	NP_081902.1#Psd3#234353# PH and SEC7 domain-containing protein 3 isoform 3 [Mus musculus]	0.968	0.831	0.776	0.864	0.820	1.219	0.708	0.880	0.563	0.756	0.660
755495449	XP_011237059.1#Igsf8#140559# PREDICTED: immunoglobulin superfamily member 8 isoform X1 [Mus musculus]	1.015	0.944	0.695	0.945	0.820	1.219	0.767	0.898	0.686	0.968	0.827
215983062	NP_065603.2#Ehd3#57440# EH domain-containing protein 3 [Mus musculus]	1.058	0.887	0.715	0.926	0.820	1.219	0.770	0.923	0.628	0.882	0.755
569016861	XP_006507197.1#Bcl2l13#94044# PREDICTED: bcl-2-like protein 13 isoform X2 [Mus musculus]	0.921	0.842	0.732	0.909	0.820	1.219	0.793	0.714	0.526	0.746	0.636
160420342	NP_659140.2#Gdap1l1#228858# ganglioside-induced differentiation-associated protein 1-like 1 [Mus musculus]	1.098	0.978	0.725	0.916	0.821	1.219	0.820	0.986	0.772	0.992	0.882
568917832	XP_006499972.1#Ywhab#54401# PREDICTED: 14-3-3 protein beta/alpha isoform X1 [Mus musculus]	1.011	0.841	0.701	0.940	0.821	1.219	0.752	0.920	0.596	0.790	0.693

568968038	XP_006513939.1#Sgta#52551# PREDICTED: small glutamine-rich tetratricopeptide repeat-containing protein alpha isoform X1 [Mus musculus]	1.045	0.958	0.774	0.868	0.821	1.218	0.752	0.963	0.850	0.999	0.925
13386338	NP_082988.1#Rap2b#74012# ras-related protein Rap-2b precursor [Mus musculus]	1.027	0.895	0.745	0.897	0.821	1.218	0.804	0.942	0.678	0.862	0.770
568983158	XP_006517219.1#Pdcd6#18570# PREDICTED: programmed cell death protein 6 isoform X1 [Mus musculus]	1.141	0.997	0.719	0.923	0.821	1.218	0.824	1.050	0.835	1.032	0.934
568952416	XP_006536280.1#Cend1#57754# PREDICTED: cell cycle exit and neuronal differentiation protein 1 isoform X1 [Mus musculus]	1.270	0.959	0.733	0.909	0.821	1.218	0.894	1.096	0.769	1.002	0.886
6679587	NP_033022.1#Rab1#19324# ras-related protein Rab-1A [Mus musculus]	1.158	0.863	0.723	0.919	0.821	1.218	0.848	1.029	0.702	0.963	0.832
13385006	NP_079843.1#Cyc1#66445# cytochrome c1, heme protein, mitochondrial [Mus musculus]	1.263	1.027	0.702	0.941	0.821	1.218	0.925	1.113	0.905	1.191	1.048
30519995	NP_848754.1#Sfxn5#94282# sideroflexin-5 [Mus musculus]	1.134	0.924	0.732	0.910	0.821	1.218	0.864	0.973	0.733	0.903	0.818
544346120	NP_001269666.1#Magoh#17149# protein mago nashi homolog [Mus musculus]	0.861	1.015	0.758	0.884	0.821	1.218	0.687	0.728	0.548	0.887	0.718
755539138	XP_006533808.2#Sept9#53860# PREDICTED: septin-9 isoform X1 [Mus musculus]	1.067	0.818	0.727	0.916	0.821	1.218	0.819	0.933	0.610	0.824	0.717
63999380	NP_038615.2#Lrpap1#16976# alpha-2-macroglobulin receptor-associated protein precursor [Mus musculus]	1.028	0.986	0.732	0.911	0.821	1.218	0.792	0.898	0.716	0.952	0.834
70778976	NP_032854.2#Pgk1#18655# phosphoglycerate kinase 1 [Mus musculus]	0.942	0.924	0.733	0.910	0.821	1.218	0.743	0.848	0.629	0.828	0.729
27369922	NP_766234.1#Dnm3#103967# dynamin-3 isoform 2 [Mus musculus]	1.189	0.843	0.742	0.901	0.821	1.218	0.865	1.036	0.717	0.944	0.830
31980762	NP_038699.2#Sod2#20656# superoxide dismutase [Mn], mitochondrial precursor [Mus musculus]	1.076	0.978	0.734	0.908	0.821	1.217	0.790	0.944	0.696	1.038	0.867
40556608	NP_032328.2#Hsp90ab1#15516# heat shock protein HSP 90-beta [Mus musculus]	1.007	0.894	0.725	0.918	0.821	1.217	0.722	0.886	0.626	0.782	0.704
568975126	XP_006533946.1#Hnrnph1#59013# PREDICTED: heterogeneous nuclear ribonucleoprotein H isoform X6 [Mus musculus]	1.037	0.971	0.656	0.987	0.821	1.217	0.639	1.063	0.691	0.963	0.827
6753138	NP_033851.1#Atp1b1#11931# sodium/potassium-transporting ATPase subunit beta-1 [Mus musculus]	1.055	0.930	0.735	0.908	0.822	1.217	0.824	0.981	0.709	0.943	0.826
568966884	XP_006513387.1#Myl6#17904# PREDICTED: myosin light polypeptide 6 isoform X5 [Mus musculus]	0.980	0.886	0.719	0.924	0.822	1.217	0.725	0.884	0.629	0.783	0.706
257796245	NP_663589.3#Dlat#235339# dihydrolipoyllysine-residue acetyltransferase component of pyruvate dehydrogenase complex, mitochondrial [Mus muscu	1.181	0.877	0.735	0.908	0.822	1.217	0.907	1.039	0.744	0.967	0.855
568956646	XP_006530987.1#Aars#234734# PREDICTED: alanine--tRNA ligase, cytoplasmic isoform X1 [Mus musculus]	1.190	0.883	0.742	0.902	0.822	1.217	0.946	1.016	0.769	0.950	0.859
7305631	NP_038869.1#Vps45#22365# vacuolar protein sorting-associated protein 45 [Mus musculus]	1.066	0.898	0.795	0.849	0.822	1.217	0.790	0.932	0.677	0.869	0.773
667751602	NP_001288140.1#Ap1g1#11765# AP-1 complex subunit gamma-1 isoform 2 [Mus musculus]	1.100	0.830	0.730	0.915	0.822	1.216	0.906	0.955	0.696	0.869	0.782
818213430	NP_001295403.1#Porcn#53627# protein-serine O-palmitoleoyltransferase porcupine isoform E [Mus musculus]	0.977	0.911	0.711	0.934	0.822	1.216	0.746	0.861	0.630	0.835	0.732
568991032	XP_006520346.1#Cyb5r3#109754# PREDICTED: NADH-cytochrome b5 reductase 3 isoform X2 [Mus musculus]	1.045	0.928	0.719	0.926	0.822	1.216	0.796	1.035	0.703	1.019	0.861
6754524	NP_034829.1#Ldha#16828# L-lactate dehydrogenase A chain isoform 1 [Mus musculus]	0.980	0.878	0.706	0.939	0.822	1.216	0.727	0.860	0.593	0.798	0.696
597517964	NP_001278003.1#Nsfl1c#386649# NSFL1 cofactor p47 isoform b [Mus musculus]	1.098	0.858	0.719	0.926	0.822	1.216	0.799	1.006	0.650	0.898	0.774
113680348	NP_032010.2#Fscn1#14086# fascin [Mus musculus]	1.063	0.873	0.705	0.941	0.823	1.216	0.814	0.945	0.683	0.889	0.786
755476121	XP_011250267.1#Gm6745#627317# PREDICTED: 60S ribosomal protein L11-like [Mus musculus]	1.051	0.853	0.721	0.924	0.823	1.216	0.809	0.920	0.635	0.840	0.738
31980806	NP_058063.2#Pgrmc1#53328# membrane-associated progesterone receptor component 1 [Mus musculus]	0.929	0.891	0.744	0.902	0.823	1.215	0.718	0.810	0.571	0.791	0.681
255069795	NP_035820.3#Vars#22321# valine--tRNA ligase [Mus musculus]	1.241	0.815	0.729	0.917	0.823	1.215	0.950	0.987	0.713	0.908	0.811
10092608	NP_038569.1#Gstp1#14870# glutathione S-transferase P 1 [Mus musculus]	0.863	1.137	0.671	0.975	0.823	1.215	0.672	0.808	0.717	0.958	0.837
755504821	XP_011238614.1#Wdr47#99512# PREDICTED: WD repeat-containing protein 47 isoform X7 [Mus musculus]	1.259	0.925	0.727	0.919	0.823	1.215	0.908	1.167	0.776	1.142	0.959
6756037	NP_035868.1#Ywhah#22629# 14-3-3 protein eta [Mus musculus]	1.059	0.819	0.722	0.924	0.823	1.215	0.750	0.788	0.545	0.676	0.610
13385492	NP_080263.1#Ndufa6#67130# NADH dehydrogenase [ubiquinone] 1 alpha subcomplex subunit 6 [Mus musculus]	1.030	0.915	0.747	0.899	0.823	1.215	0.764	0.892	0.637	0.861	0.749
18266680	NP_077150.1#Oxct1#67041# succinyl-CoA:3-ketoacid coenzyme A transferase 1, mitochondrial precursor [Mus musculus]	1.093	0.872	0.723	0.923	0.823	1.215	0.830	0.977	0.677	0.852	0.764
117606375	NP_036019.2#Sept3#24050# neuronal-specific septin-3 [Mus musculus]	1.162	0.835	0.691	0.956	0.823	1.215	0.840	0.990	0.631	0.850	0.740
31981822	NP_034106.2#Cst3#13010# cystatin-C precursor [Mus musculus]	1.036	0.832	0.641	1.006	0.823	1.215	0.799	0.906	0.624	0.795	0.710
9789937	NP_062768.1#Dnaja2#56445# dnaJ homolog subfamily A member 2 [Mus musculus]	1.073	0.867	0.690	0.956	0.823	1.214	0.787	0.981	0.676	0.857	0.767
755522217	XP_011239997.1#Prkcb#18751# PREDICTED: protein kinase C beta type isoform X2 [Mus musculus]	1.199	0.796	0.757	0.890	0.824	1.214	0.918	1.057	0.745	0.806	0.776
312596940	NP_001186117.1#Snx5#69178# sorting nexin-5 [Mus musculus]	0.993	0.898	0.814	0.834	0.824	1.214	0.789	0.846	0.615	0.853	0.734
569017187	XP_006536087.1#Bcat1#12035# PREDICTED: branched-chain-amino-acid aminotransferase, cytosolic isoform X1 [Mus musculus]	1.067	1.023	0.751	0.896	0.824	1.214	0.770	0.993	0.760	1.043	0.901
568962761	XP_006511700.1#Gnai2#14678# PREDICTED: guanine nucleotide-binding protein G(i) subunit alpha-2 isoform X1 [Mus musculus]	1.112	0.963	0.741	0.906	0.824	1.214	0.911	1.156	0.861	1.189	1.025
568972134	XP_006532520.1#Pdk2#18604# PREDICTED: pyruvate dehydrogenase kinase, isoenzyme 2 isoform X1 [Mus musculus]	1.180	0.886	0.766	0.882	0.824	1.214	0.871	1.076	0.737	0.918	0.827
71040107	NP_001020771.1#Cadm1#54725# cell adhesion molecule 1 isoform d precursor [Mus musculus]	1.025	1.040	0.757	0.891	0.824	1.214	0.753	0.936	0.802	0.955	0.878
158508501	NP_998779.2#Sept5#18951# septin-5 [Mus musculus]	1.129	0.807	0.724	0.923	0.824	1.214	0.857	1.038	0.622	0.865	0.744
110625954	NP_082664.1#Ndufv2#72900# NADH dehydrogenase [ubiquinone] flavoprotein 2, mitochondrial isoform 1 precursor [Mus musculus]	1.162	0.961	0.717	0.930	0.824	1.214	0.877	1.012	0.787	1.034	0.911
13385598	NP_080371.1#Snrpd3#67332# small nuclear ribonucleoprotein Sm D3 [Mus musculus]	0.833	0.805	0.718	0.930	0.824	1.214	0.679	0.694	0.498	0.607	0.552
71774133	NP_035279.2#Ppib#19035# peptidyl-prolyl cis-trans isomerase B precursor [Mus musculus]	1.008	0.986	0.786	0.861	0.824	1.214	0.741	0.920	0.707	0.931	0.819
568930664	XP_006538651.1#Kcnab2#16498# PREDICTED: voltage-gated potassium channel subunit beta-2 isoform X7 [Mus musculus]	1.158	0.866	0.730	0.918	0.824	1.214	0.897	1.116	0.742	1.020	0.881
339895916	NP_001229973.1#Ap1b1#11764# AP-1 complex subunit beta-1 isoform 3 [Mus musculus]	1.060	0.852	0.727	0.921	0.824	1.214	0.787	0.953	0.693	0.871	0.782
58037117	NP_080964.1#Ndufs3#68349# NADH dehydrogenase [ubiquinone] iron-sulfur protein 3, mitochondrial precursor [Mus musculus]	1.158	0.909	0.707	0.941	0.824	1.213	0.876	1.042	0.747	0.976	0.861
6671509	NP_031419.1#Actb#11461# actin, cytoplasmic 1 [Mus musculus]	1.150	0.874	0.732	0.916	0.824	1.213	0.862	1.051	0.726	0.957	0.842
568971592	XP_006532257.1#Flot2#14252# PREDICTED: flotillin-2 isoform X3 [Mus musculus]	1.055	0.855	0.739	0.910	0.824	1.213	0.841	0.948	0.638	0.869	0.754
755521369	XP_011249216.1#Ppfia3#76787# PREDICTED: liprin-alpha-3 isoform X2 [Mus musculus]	1.275	0.879	0.708	0.941	0.825	1.213	0.936	1.166	0.813	1.036	0.924
755560613	XP_011245162.1#Pura#19290# PREDICTED: transcriptional activator protein Pur-alpha isoform X1 [Mus musculus]	0.986	0.885	0.743	0.906	0.825	1.213	0.723	0.820	0.560	0.763	0.662
239787090	NP_001155139.1#Acox2#93732# peroxisomal acyl-coenzyme A oxidase 2 [Mus musculus]	1.230	0.861	0.706	0.943	0.825	1.213	0.932	1.096	0.801	0.947	0.874
239985643	NP_001155219.1#Palm#18483# paralemmin-1 isoform 2 [Mus musculus]	1.105	0.881	0.713	0.936	0.825	1.213	0.857	0.969	0.725	0.937	0.831
31560737	NP_031448.2#Adss#11566# adenylosuccinate synthetase isozyme 2 [Mus musculus]	1.198	0.933	0.757	0.892	0.825	1.212	0.921	1.010	0.778	1.056	0.917
568915813	XP_006498993.1#Plcb1#18795# PREDICTED: 1-phosphatidylinositol 4,5-bisphosphate phosphodiesterase beta-1 isoform X1 [Mus musculus]	1.279	0.790	0.725	0.924	0.825	1.212	0.935	1.132	0.713	0.945	0.829
6671672	NP_031630.1#Capza2#12343# F-actin-capping protein subunit alpha-2 [Mus musculus]	0.963	0.861	0.729	0.921	0.825	1.212	0.682	0.994	0.642	0.793	0.718
31981269	NP_075629.2#Glrx3#30926# glutaredoxin-3 [Mus musculus]	1.079	0.897	0.743	0.907	0.825	1.212	0.848	0.945	0.657	0.948	0.802
568889869	XP_006543820.1#LOC102641678#102641678# PREDICTED: ATP synthase subunit O, mitochondrial [Mus musculus]	1.038	0.922	0.720	0.930	0.825	1.212	0.756	0.915	0.669	0.853	0.761
12963569	NP_075689.1#Ppp1r7#66385# protein phosphatase 1 regulatory subunit 7 [Mus musculus]	0.869	0.884	0.755	0.895	0.825	1.212	0.653	0.796	0.567	0.761	0.664
27229051	NP_080543.2#Necap1#67602# adaptin ear-binding coat-associated protein 1 [Mus musculus]	1.065	0.897	0.713	0.937	0.825	1.212	0.764	0.966	0.678	0.835	0.756
568984240	XP_006517602.1#Kif2a#16563# PREDICTED: kinesin-like protein KIF2A isoform X6 [Mus musculus]	1.029	0.926	0.736	0.914	0.825	1.212	0.806	0.894	0.673	0.900	0.786
113195678	NP_034814.2#Lamp1#16783# lysosome-associated membrane glycoprotein 1 precursor [Mus musculus]	0.965	0.856	0.747	0.904	0.825	1.212	0.689	0.903	0.624	0.739	0.682
568967045	XP_006513461.1#Sar1a#20224# PREDICTED: GTP-binding protein SAR1a isoform X1 [Mus musculus]	0.964	0.999	0.711	0.940	0.825	1.211	0.688	0.902	0.686	0.899	0.793
755498690	XP_011237589.1#Elmo2#140579# PREDICTED: engulfment and cell motility protein 2 isoform X3 [Mus musculus]	1.140	0.886	0.749	0.902	0.826	1.211	0.877	0.975	0.729	0.937	0.833
568975386	XP_006534072.1#Rpl38#67671# PREDICTED: 60S ribosomal protein L38 isoform X1 [Mus musculus]	0.909	0.809	0.698	0.954	0.826	1.211	0.722	0.780	0.549	0.666	0.608
6680946	NP_031731.1#Cirbp#12696# cold-inducible RNA-binding protein [Mus musculus]	1.045	1.002	0.722	0.930	0.826	1.211	0.772	0.955	0.732	0.998	0.865
60687506	NP_033787.2#Aldoc#11676# fructose-bisphosphate aldolase C [Mus musculus]	1.044	0.920	0.721	0.931	0.826	1.211	0.820	0.927	0.686	0.908	0.797
29789148	NP_075661.1#Ndufb9#66218# NADH dehydrogenase [ubiquinone] 1 beta subcomplex subunit 9 [Mus musculus]	1.048	0.968	0.786	0.865	0.826	1.211	0.813	0.918	0.731	0.946	0.838
31560697	NP_032223.2#H1f0#14958# histone H1.0 [Mus musculus]	1.094	0.920	0.717	0.935	0.826	1.211	0.843	1.015	0.711	0.994	0.852
568959251	XP_006510273.1#Ntm#235106# PREDICTED: neurotrimin isoform X6 [Mus musculus]	1.008	0.780	0.734	0.918	0.826	1.211	0.839	0.967	0.587	0.771	0.679
13385472	NP_080250.1#Rpl14#67115# 60S ribosomal protein L14 [Mus musculus]	0.990	0.879	0.686	0.966	0.826	1.211	0.732	0.884	0.617	0.856	0.737
568935110	XP_006534749.1#Lrrc8c#100604# PREDICTED: volume-regulated anion channel subunit LRRC8C isoform X2 [Mus musculus]	1.031	0.864	0.717	0.935	0.826	1.211	0.776	0.927	0.627	0.844	0.735
169790856	NP_766101.3#Fam126b#213056# protein FAM126B [Mus musculus]	1.182	0.848	0.760	0.892	0.826	1.211	0.887	1.066	0.706	0.950	0.828
19526818	NP_598429.1#Slc25a3#18674# phosphate carrier protein, mitochondrial precursor [Mus musculus]	1.250	0.903	0.716	0.936	0.826	1.210	0.945	1.165	0.787	1.073	0.930
13385312	NP_080112.1#Plin3#66905# perilipin-3 [Mus musculus]	1.385	0.970	0.724	0.928	0.826	1.210	1.078	1.211	1.008	1.212	1.110
124248577	NP_036004.2#Psmc4#23996# 26S protease regulatory subunit 6B [Mus musculus]	1.001	0.945	0.756	0.896	0.826	1.210	0.781	0.874	0.669	0.898	0.783
568917472	XP_006499797.1#Tcp11l1#320554# PREDICTED: T-complex protein 11-like protein 1 isoform X1 [Mus musculus]	1.067	0.889	0.737	0.915	0.826	1.210	0.771	0.993	0.596	0.971	0.784
31981690	NP_112442.2#Hspa8#15481# heat shock cognate 71 kDa protein [Mus musculus]	1.079	0.906	0.737	0.916	0.826	1.210	0.809	0.940	0.699	0.911	0.805
23943898	NP_084202.2#Irak4#266632# interleukin-1 receptor-associated kinase 4 [Mus musculus]	1.159	0.856	0.757	0.896	0.827	1.210	0.918	0.998	0.718	0.922	0.820
568934349	XP_006504080.1#Preb#50907# PREDICTED: prolactin regulatory element-binding protein isoform X2 [Mus musculus]	1.061	0.969	0.732	0.921	0.827	1.210	0.831	0.923	0.764	0.937	0.850

19525729	NP_084280.2#Cryl1#68631# lambda-crystallin homolog [Mus musculus]	1.029	0.907	0.699	0.954	0.827	1.210	0.752	0.950	0.654	0.889	0.772
755509924	XP_011248613.1#Park7#57320# PREDICTED: protein DJ-1 isoform X1 [Mus musculus]	0.912	0.993	0.761	0.892	0.827	1.210	0.694	0.814	0.634	0.859	0.746
225543482	NP_444319.3#Naa15#74838# N-alpha-acetyltransferase 15, NatA auxiliary subunit [Mus musculus]	1.068	0.985	0.730	0.923	0.827	1.210	0.803	0.964	0.735	1.005	0.870
8393866	NP_058674.1#Oat#18242# ornithine aminotransferase, mitochondrial precursor [Mus musculus]	1.091	0.975	0.722	0.932	0.827	1.209	0.840	0.965	0.733	1.026	0.880
568907993	XP_006529127.1#Cops8#108679# PREDICTED: COP9 signalosome complex subunit 8 isoform X1 [Mus musculus]	1.003	0.940	0.772	0.882	0.827	1.209	0.779	0.881	0.706	0.851	0.778
38348492	NP_941027.1#Mtss1l#244654# MTSS1-like protein [Mus musculus]	1.194	0.901	0.744	0.910	0.827	1.209	0.842	1.133	0.796	0.985	0.890
442535508	NP_001259026.1#Fabp5#16592# fatty acid-binding protein, epidermal isoform 2 [Mus musculus]	1.116	1.001	0.769	0.885	0.827	1.209	0.868	0.980	0.805	1.045	0.925
6756051	NP_035881.1#Zfp207#22680# BUB3-interacting and GLEBS motif-containing protein ZNF207 isoform 4 [Mus musculus]	1.019	0.829	0.684	0.970	0.827	1.209	0.693	0.992	0.602	0.794	0.698
13277394	NP_077798.1#Grpel1#17713# grpE protein homolog 1, mitochondrial precursor [Mus musculus]	1.145	0.824	0.752	0.902	0.827	1.209	0.878	1.004	0.687	0.960	0.823
6680748	NP_031531.1#Atp5a1#11946# ATP synthase subunit alpha, mitochondrial precursor [Mus musculus]	1.095	0.917	0.730	0.925	0.827	1.209	0.832	0.980	0.727	0.949	0.838
7106257	NP_033857.1#Atp8a1#11980# phospholipid-transporting ATPase IA isoform b [Mus musculus]	1.093	0.830	0.718	0.937	0.827	1.209	0.818	0.991	0.677	0.825	0.751
755498668	XP_011237583.1#Epb4.1l1#13821# PREDICTED: band 4.1-like protein 1 isoform X8 [Mus musculus]	1.081	0.910	0.749	0.906	0.828	1.208	0.871	0.969	0.711	0.924	0.818
46849708	NP_035636.1#Sucla2#20916# succinyl-CoA ligase [ADP-forming] subunit beta, mitochondrial precursor [Mus musculus]	1.083	0.891	0.758	0.897	0.828	1.208	0.807	0.976	0.684	0.893	0.788
6755386	NP_035439.1#S100a1#20193# protein S100-A1 [Mus musculus]	0.956	0.942	0.738	0.917	0.828	1.208	0.718	0.865	0.676	0.815	0.745
568930711	XP_006538673.1#Mfn2#170731# PREDICTED: mitofusin-2 isoform X1 [Mus musculus]	1.186	0.906	0.746	0.909	0.828	1.208	0.883	1.081	0.734	1.044	0.889
568999382	XP_006523896.1#Prkce#18754# PREDICTED: protein kinase C epsilon type isoform X2 [Mus musculus]	1.104	0.829	0.703	0.953	0.828	1.208	0.808	0.987	0.661	0.872	0.767
27923929	NP_778163.1#Hpcal4#170638# hippocalcin-like protein 4 [Mus musculus]	1.183	0.766	0.719	0.937	0.828	1.208	0.890	1.053	0.628	0.796	0.712
568961798	XP_006511383.1#Scamp5#56807# PREDICTED: secretory carrier-associated membrane protein 5 isoform X1 [Mus musculus]	0.968	0.881	0.748	0.908	0.828	1.208	0.731	0.838	0.611	0.771	0.691
268370088	NP_082538.2#Setd3#52690# histone-lysine N-methyltransferase setd3 [Mus musculus]	1.547	0.821	0.729	0.927	0.828	1.208	1.055	1.214	0.888	1.110	0.999
340007377	NP_001229979.1#Atp6v0a1#11975# V-type proton ATPase 116 kDa subunit a isoform 1 isoform 3 [Mus musculus]	1.084	0.848	0.725	0.931	0.828	1.208	0.868	1.058	0.684	0.913	0.799
6753556	NP_034113.1#Ctsd#13033# cathepsin D precursor [Mus musculus]	1.132	0.878	0.725	0.931	0.828	1.207	0.801	1.048	0.703	0.989	0.846
13385384	NP_080170.1#Psmd12#66997# 26S proteasome non-ATPase regulatory subunit 12 [Mus musculus]	0.984	0.941	0.756	0.900	0.828	1.207	0.779	0.880	0.662	0.873	0.767
27229101	NP_081740.2#Fam213a#70564# redox-regulatory protein FAM213A [Mus musculus]	1.163	0.990	0.714	0.943	0.828	1.207	0.794	1.094	0.733	1.054	0.894
549806742	NP_001272357.1#Cacnb4#12298# voltage-dependent L-type calcium channel subunit beta-4 isoform e [Mus musculus]	0.962	0.803	0.741	0.916	0.828	1.207	0.746	0.848	0.574	0.706	0.640
37718972	NP_937803.1#Smarcc2#68094# SWI/SNF complex subunit SMARCC2 isoform 3 [Mus musculus]	1.025	0.891	0.727	0.930	0.828	1.207	0.771	0.927	0.665	0.849	0.757
165932389	NP_573490.3#Nup155#170762# nuclear pore complex protein Nup155 [Mus musculus]	1.215	0.841	0.773	0.884	0.829	1.207	0.915	1.098	0.746	0.946	0.846
568908350	XP_006529301.1#Ncl#17975# PREDICTED: nucleolin isoform X1 [Mus musculus]	1.127	0.863	0.724	0.933	0.829	1.207	0.886	0.982	0.706	0.905	0.806
165972307	NP_001107041.1#Stxbp1#20910# syntaxin-binding protein 1 isoform a [Mus musculus]	1.173	1.080	0.749	0.909	0.829	1.206	0.914	1.028	0.995	1.099	1.047
19526912	NP_598487.1#St13#70356# hsc70-interacting protein [Mus musculus]	0.949	0.964	0.713	0.945	0.829	1.206	0.753	0.880	0.639	0.950	0.794
31980939	NP_080719.2#Mtfp1#67900# mitochondrial fission process protein 1 [Mus musculus]	0.947	0.869	0.682	0.977	0.829	1.206	0.691	0.900	0.533	0.773	0.653
28076935	NP_081427.1#Dctn2#69654# dynactin subunit 2 isoform 3 [Mus musculus]	1.096	0.891	0.774	0.884	0.829	1.206	0.778	0.954	0.675	0.887	0.781
214010170	NP_038520.2#Clu#12759# clusterin precursor [Mus musculus]	1.203	0.820	0.704	0.954	0.829	1.206	0.828	1.168	0.697	0.940	0.818
149273202	XP_001476757.1#Gm20899#100042025# PREDICTED: glyceraldehyde-3-phosphate dehydrogenase [Mus musculus]	0.979	0.928	0.716	0.943	0.829	1.206	0.769	0.896	0.647	0.871	0.759
6679299	NP_032857.1#Phb#18673# prohibitin [Mus musculus]	1.038	0.923	0.739	0.921	0.830	1.205	0.827	0.914	0.696	0.912	0.804
755497651	XP_011237415.1#Scai#320271# PREDICTED: protein SCAI isoform X1 [Mus musculus]	1.125	0.881	0.712	0.948	0.830	1.205	0.834	1.033	0.693	0.952	0.822
344925843	NP_851383.2#Atpaf1#230649# ATP synthase mitochondrial F1 complex assembly factor 1 [Mus musculus]	0.846	0.896	0.785	0.874	0.830	1.205	0.626	0.778	0.504	0.754	0.629
294997259	NP_001171128.1#Grin1#14810# glutamate receptor ionotropic, NMDA 1 isoform 3 precursor [Mus musculus]	0.930	0.796	0.765	0.895	0.830	1.205	0.721	0.823	0.551	0.678	0.615
755513560	XP_011246490.1#Rph3a#19894# PREDICTED: rabphilin-3A isoform X2 [Mus musculus]	1.240	0.876	0.739	0.921	0.830	1.205	0.922	1.094	0.751	0.988	0.869
18017605	NP_542127.1#Ube2n#93765# ubiquitin-conjugating enzyme E2 N [Mus musculus]	1.057	0.926	0.744	0.916	0.830	1.205	0.752	0.943	0.675	0.884	0.780
568994656	XP_006521871.1#Cd47#16423# PREDICTED: leukocyte surface antigen CD47 isoform X3 [Mus musculus]	1.138	0.864	0.721	0.939	0.830	1.205	0.848	1.060	0.720	0.936	0.828
755516671	XP_011239634.1#Mgll#23945# PREDICTED: monoglyceride lipase isoform X1 [Mus musculus]	1.155	0.819	0.743	0.918	0.830	1.205	0.797	1.023	0.695	0.927	0.811
755543957	XP_011242554.1#Wdr37#207615# PREDICTED: WD repeat-containing protein 37 isoform X3 [Mus musculus]	1.276	0.837	0.733	0.927	0.830	1.205	0.956	1.149	0.741	0.893	0.817
22122625	NP_666220.1#Hibch#227095# 3-hydroxyisobutyryl-CoA hydrolase, mitochondrial precursor [Mus musculus]	1.056	0.881	0.766	0.894	0.830	1.205	0.820	0.936	0.704	0.844	0.774
238624114	NP_598949.3#Acot2#171210# acyl-coenzyme A thioesterase 2, mitochondrial precursor [Mus musculus]	1.164	0.901	0.695	0.966	0.830	1.205	0.912	1.020	0.740	1.001	0.871
755522634	XP_011240088.1#Dlg2#23859# PREDICTED: disks large homolog 2 isoform X22 [Mus musculus]	0.940	0.904	0.739	0.922	0.830	1.205	0.723	0.840	0.625	0.788	0.706
6678131	NP_033298.1#Srm#20810# spermidine synthase [Mus musculus]	1.202	0.870	0.730	0.930	0.830	1.205	0.844	1.151	0.719	1.016	0.867
146219837	NP_034253.3#Eif3a#13669# eukaryotic translation initiation factor 3 subunit A [Mus musculus]	1.047	0.827	0.789	0.872	0.830	1.204	0.821	1.012	0.648	0.897	0.773
167716837	YP_001686698.1#ND1#5912287# NADH dehydrogenase subunit 1 [Mus musculus musculus]	1.114	0.900	0.752	0.909	0.830	1.204	0.824	1.026	0.710	0.955	0.832
31980942	NP_061352.2#Impa1#55980# inositol monophosphatase 1 [Mus musculus]	1.031	0.846	0.715	0.946	0.830	1.204	0.777	0.864	0.589	0.794	0.692
31560686	NP_032327.2#Hspa2#15512# heat shock-related 70 kDa protein 2 [Mus musculus]	1.046	0.931	0.771	0.890	0.830	1.204	0.757	0.982	0.712	0.906	0.809
755522937	XP_011240168.1#Stx1b#56216# PREDICTED: syntaxin-1B isoform X2 [Mus musculus]	1.270	0.928	0.749	0.912	0.831	1.204	0.926	1.175	0.841	1.111	0.976
84490421	NP_001033726.1#Gng2#14702# guanine nucleotide-binding protein G(I)/G(S)/G(O) subunit gamma-2 precursor [Mus musculus]	1.440	0.798	0.754	0.908	0.831	1.204	1.030	1.339	0.814	1.115	0.964
157951727	NP_663785.2#Ctnna2#12386# catenin alpha-2 isoform 1 [Mus musculus]	1.151	0.857	0.729	0.932	0.831	1.204	0.908	1.070	0.719	0.935	0.827
568957227	XP_006531268.1#Scoc#56367# PREDICTED: short coiled-coil protein isoform X2 [Mus musculus]	0.920	0.977	0.773	0.888	0.831	1.204	0.664	0.865	0.657	0.836	0.746
568944750	XP_006539551.1#Capns1#12336# PREDICTED: calpain small subunit 1 isoform X1 [Mus musculus]	1.129	0.868	0.792	0.869	0.831	1.204	0.889	0.987	0.667	0.958	0.812
225637531	NP_079620.2#Eif3f#66085# eukaryotic translation initiation factor 3 subunit F [Mus musculus]	1.018	0.832	0.746	0.916	0.831	1.204	0.793	0.894	0.625	0.818	0.722
13386106	NP_080899.1#Nudt21#68219# cleavage and polyadenylation specificity factor subunit 5 [Mus musculus]	0.898	0.913	0.725	0.937	0.831	1.203	0.713	0.779	0.594	0.769	0.681
33859560	NP_034403.1#Gdi1#14567# rab GDP dissociation inhibitor alpha [Mus musculus]	1.012	0.926	0.730	0.933	0.831	1.203	0.780	0.966	0.699	0.870	0.784
9506971	NP_062283.1#Pfn2#18645# profilin-2 [Mus musculus]	0.907	0.873	0.729	0.934	0.831	1.203	0.750	0.908	0.656	0.825	0.740
12746424	NP_075534.1#Dpysl5#65254# dihydropyrimidinase-related protein 5 [Mus musculus]	0.992	1.028	0.735	0.928	0.831	1.203	0.774	0.894	0.731	1.027	0.879
568939259	XP_006504978.1#Hsph1#15505# PREDICTED: heat shock protein 105 kDa isoform X2 [Mus musculus]	1.109	0.891	0.736	0.927	0.831	1.203	0.847	1.002	0.777	0.950	0.864
224922803	NP_034404.3#Gpd2#14571# glycerol-3-phosphate dehydrogenase, mitochondrial precursor [Mus musculus]	1.187	0.828	0.747	0.915	0.831	1.203	0.891	1.087	0.694	0.982	0.838
110835726	NP_001036085.1#Dhx15#13204# pre-mRNA-splicing factor ATP-dependent RNA helicase DHX15 isoform 1 [Mus musculus]	0.963	0.958	0.762	0.901	0.831	1.203	0.762	0.840	0.710	0.824	0.767
113199771	NP_034944.2#Mog#17441# myelin-oligodendrocyte glycoprotein precursor [Mus musculus]	1.010	1.207	0.705	0.958	0.831	1.203	0.784	0.936	0.935	1.309	1.122
19527148	NP_598691.1#Creld1#171508# cysteine-rich with EGF-like domain protein 1 precursor [Mus musculus]	1.349	0.855	0.739	0.924	0.832	1.202	1.014	1.229	0.857	1.062	0.959
34328251	NP_062787.2#Dbn1#56320# drebrin isoform 3 [Mus musculus]	1.153	0.853	0.749	0.915	0.832	1.202	0.851	1.089	0.739	0.923	0.831
9938002	NP_064674.1#Lgi1#56839# leucine-rich glioma-inactivated protein 1 precursor [Mus musculus]	1.003	0.826	0.742	0.922	0.832	1.202	0.762	0.908	0.572	0.804	0.688
7110703	NP_032976.1#Psmc5#19184# 26S protease regulatory subunit 8 [Mus musculus]	0.946	0.842	0.798	0.866	0.832	1.202	0.832	1.029	0.673	0.860	0.766
568931690	XP_006539140.1#Mtor#56717# PREDICTED: serine/threonine-protein kinase mTOR isoform X1 [Mus musculus]	1.242	0.821	0.738	0.926	0.832	1.202	0.853	1.019	0.678	0.834	0.756
568965113	XP_006512658.1#Pcmt1#18537# PREDICTED: protein-L-isoaspartate(D-aspartate) O-methyltransferase isoform X6 [Mus musculus]	0.941	0.894	0.727	0.937	0.832	1.202	0.729	0.797	0.603	0.828	0.715
568961539	XP_006511259.1#Map2k1#26395# PREDICTED: dual specificity mitogen-activated protein kinase kinase 1 isoform X1 [Mus musculus]	1.046	0.866	0.740	0.925	0.832	1.201	0.825	0.928	0.662	0.833	0.748
255003735	NP_035417.2#Rpl10a#19896# 60S ribosomal protein L10a [Mus musculus]	1.012	0.873	0.723	0.942	0.832	1.201	0.844	0.985	0.720	0.900	0.810
755508327	XP_011238859.1#Acot11#329910# PREDICTED: acyl-coenzyme A thioesterase 11 isoform X2 [Mus musculus]	1.286	0.890	0.734	0.930	0.832	1.201	0.948	1.193	0.806	1.100	0.953
13385168	NP_079986.1#Uqcrfs1#66694# cytochrome b-c1 complex subunit Rieske, mitochondrial [Mus musculus]	0.994	0.935	0.739	0.926	0.833	1.201	0.836	0.786	0.714	0.951	0.832
568974806	XP_006533792.1#Cntnap1#53321# PREDICTED: contactin-associated protein 1 isoform X1 [Mus musculus]	1.125	0.869	0.731	0.934	0.833	1.201	0.880	1.058	0.729	0.962	0.846
110626031	NP_035447.3#Khdrbs1#20218# KH domain-containing, RNA-binding, signal transduction-associated protein 1 [Mus musculus]	1.185	0.914	0.834	0.831	0.833	1.201	1.003	0.970	0.708	1.096	0.902
157823795	NP_001102512.1#Kif21a#16564# kinesin-like protein KIF21A isoform 4 [Mus musculus]	0.976	0.963	0.771	0.894	0.833	1.201	0.752	0.874	0.683	0.883	0.783
6679261	NP_032836.1#Pdha1#18597# pyruvate dehydrogenase E1 component subunit alpha, somatic form, mitochondrial precursor [Mus musculus]	1.113	0.860	0.732	0.934	0.833	1.201	0.879	1.010	0.659	0.938	0.798
113204613	NP_034967.2#Map6#17760# microtubule-associated protein 6 isoform 1 [Mus musculus]	1.108	0.900	0.736	0.930	0.833	1.201	0.875	1.004	0.752	0.994	0.873
294774562	NP_001171030.1#Gng12#14701# guanine nucleotide-binding protein G(I)/G(S)/G(O) subunit gamma-12 [Mus musculus]	1.357	1.020	0.704	0.962	0.833	1.200	1.101	1.198	0.985	1.361	1.173
568934035	XP_006503926.1#Wdr1#22388# PREDICTED: WD repeat-containing protein 1 isoform X1 [Mus musculus]	1.007	0.865	0.842	0.825	0.833	1.200	0.790	0.925	0.712	0.789	0.751
312032462	NP_001185842.1#Arhgef2#16800# rho guanine nucleotide exchange factor 2 isoform 4 [Mus musculus]	1.210	0.870	0.735	0.931	0.833	1.200	0.872	1.144	0.795	0.959	0.877
755497126	XP_006497717.2#Dnm1#13429# PREDICTED: dynamin-1 isoform X6 [Mus musculus]	1.193	0.796	0.763	0.903	0.833	1.200	0.938	1.056	0.751	0.849	0.800

19527228	NP_598768.1#Cisd1#52637# CDGSH iron-sulfur domain-containing protein 1 [Mus musculus]	1.160	0.925	0.747	0.920	0.833	1.200	0.871	1.028	0.796	1.104	0.950
568962941	XP_006511785.1#Camkv#235604# PREDICTED: caM kinase-like vesicle-associated protein isoform X1 [Mus musculus]	0.882	0.804	0.741	0.926	0.833	1.200	0.673	0.780	0.528	0.642	0.585
568986628	XP_006518571.1#Dpysl2#12934# PREDICTED: dihydropyrimidinase-related protein 2 isoform X1 [Mus musculus]	1.083	0.898	0.739	0.928	0.833	1.200	0.811	0.994	0.679	0.920	0.799
6755040	NP_035202.1#Pfn1#18643# profilin-1 [Mus musculus]	0.967	0.914	0.721	0.947	0.834	1.200	0.761	0.891	0.635	0.822	0.728
116256510	NP_001070732.1#Ap2a1#11771# AP-2 complex subunit alpha-1 isoform b [Mus musculus]	1.243	0.829	0.750	0.917	0.834	1.200	0.971	1.090	0.777	1.008	0.893
54292132	NP_803155.1#Psat1#107272# phosphoserine aminotransferase isoform 1 [Mus musculus]	0.999	0.986	0.737	0.930	0.834	1.200	0.770	0.953	0.713	0.939	0.826
31088914	NP_852063.1#Anks1b#77531# ankyrin repeat and sterile alpha motif domain-containing protein 1B isoform 2 [Mus musculus]	1.029	0.790	0.698	0.969	0.834	1.199	0.736	0.860	0.582	0.732	0.657
568921739	XP_006501036.1#Cryz#12972# PREDICTED: quinone oxidoreductase isoform X1 [Mus musculus]	1.040	0.933	0.785	0.883	0.834	1.199	0.786	0.914	0.720	0.901	0.810
568977910	XP_006515226.1#Efr3b#668212# PREDICTED: protein EFR3 homolog B isoform X2 [Mus musculus]	1.433	0.855	0.759	0.909	0.834	1.199	1.053	1.336	0.887	1.156	1.021
755544221	XP_011242606.1#Amph#218038# PREDICTED: amphiphysin isoform X1 [Mus musculus]	1.097	0.848	0.734	0.934	0.834	1.199	0.816	1.012	0.649	0.905	0.777
568973007	XP_006532937.1#Arhgap44#216831# PREDICTED: rho GTPase-activating protein 44 isoform X4 [Mus musculus]	1.037	0.856	0.800	0.868	0.834	1.199	0.788	0.941	0.673	0.807	0.740
599043813	NP_001278070.1#Rabep1#54189# rab GTPase-binding effector protein 1 isoform 2 [Mus musculus]	1.140	0.871	0.715	0.953	0.834	1.199	0.852	1.049	0.738	0.918	0.828
254692859	NP_079634.2#Ndufa9#66108# NADH dehydrogenase [ubiquinone] 1 alpha subcomplex subunit 9, mitochondrial precursor [Mus musculus]	1.078	0.917	0.762	0.906	0.834	1.199	0.796	1.013	0.737	0.878	0.807
22122825	NP_666355.1#Actr2#66713# actin-related protein 2 [Mus musculus]	1.093	0.883	0.748	0.920	0.834	1.199	0.825	1.015	0.723	0.949	0.836
6678674	NP_032518.1#Ldhb#16832# L-lactate dehydrogenase B chain isoform 1 [Mus musculus]	1.005	0.976	0.744	0.924	0.834	1.199	0.743	0.898	0.636	0.861	0.748
568940591	XP_006505567.1#Eno2#13807# PREDICTED: gamma-enolase isoform X1 [Mus musculus]	0.978	0.946	0.732	0.936	0.834	1.199	0.757	0.919	0.665	0.885	0.775
755530066	XP_006511148.2#Dmxl2#235380# PREDICTED: dmX-like protein 2 isoform X3 [Mus musculus]	1.244	0.815	0.752	0.916	0.834	1.199	0.952	1.119	0.734	0.949	0.842
47059486	NP_064667.2#Pdxp#57028# pyridoxal phosphate phosphatase [Mus musculus]	0.955	0.913	0.722	0.947	0.834	1.199	0.673	0.829	0.610	0.810	0.710
6755372	NP_036182.1#Rps3#27050# 40S ribosomal protein S3 [Mus musculus]	1.063	0.833	0.687	0.982	0.835	1.198	0.817	0.935	0.687	0.865	0.776
27370092	NP_766333.1#Tufm#233870# elongation factor Tu, mitochondrial isoform 1 [Mus musculus]	1.145	0.921	0.719	0.951	0.835	1.198	0.823	1.034	0.769	1.002	0.886
315507139	NP_001186831.1#Pfdn4#109054# prefoldin subunit 4 isoform 3 [Mus musculus]	1.187	0.884	0.763	0.907	0.835	1.198	0.967	1.015	0.707	1.046	0.876
21312314	NP_082483.1#Dusp3#72349# dual specificity protein phosphatase 3 [Mus musculus]	0.928	0.940	0.732	0.938	0.835	1.198	0.744	0.877	0.693	0.858	0.776
21312036	NP_081806.1#Rufy3#52822# protein RUFY3 isoform 4 [Mus musculus]	0.984	0.954	0.708	0.962	0.835	1.197	0.783	0.918	0.716	0.914	0.815
114326446	NP_071855.2#Myh9#17886# myosin-9 [Mus musculus]	1.080	0.924	0.740	0.930	0.835	1.197	0.801	1.003	0.718	0.949	0.833
262118191	NP_766520.2#Nlgn3#245537# neuroligin-3 precursor [Mus musculus]	1.141	0.852	0.749	0.922	0.835	1.197	0.874	1.033	0.676	0.949	0.813
568987240	XP_006518868.1#Tsc22d1#21807# PREDICTED: TSC22 domain family protein 1 isoform X6 [Mus musculus]	1.107	0.876	0.694	0.977	0.835	1.197	0.878	0.971	0.698	0.923	0.810
568912243	XP_006497460.1#Pip4k2a#18718# PREDICTED: phosphatidylinositol 5-phosphate 4-kinase type-2 alpha isoform X4 [Mus musculus]	1.250	0.930	0.738	0.933	0.835	1.197	0.944	1.143	0.884	1.057	0.971
19482160	NP_082347.1#Cotl1#72042# coactosin-like protein [Mus musculus]	0.976	0.869	0.759	0.912	0.835	1.197	0.799	0.980	0.735	0.882	0.808
568903782	XP_006543536.1#LOC102642689#102642689# PREDICTED: 40S ribosomal protein SA [Mus musculus]	0.815	0.896	0.795	0.876	0.836	1.197	0.629	0.817	0.585	0.759	0.672
157951668	NP_065065.3#Ank#11732# progressive ankylosis protein [Mus musculus]	1.029	1.007	0.684	0.988	0.836	1.197	0.800	0.947	0.745	0.939	0.842
755532899	XP_011241444.1#Prep#19072# PREDICTED: prolyl endopeptidase isoform X1 [Mus musculus]	1.041	0.907	0.802	0.869	0.836	1.197	0.788	0.951	0.634	0.942	0.788
568921751	XP_006501042.1#Dclk1#13175# PREDICTED: serine/threonine-protein kinase DCLK1 isoform X3 [Mus musculus]	1.102	0.856	0.751	0.920	0.836	1.197	0.763	0.990	0.608	0.822	0.715
66792896	NP_001019731.1#Snx32#225861# sorting nexin-32 [Mus musculus]	1.222	0.784	0.820	0.851	0.836	1.197	0.947	1.095	0.695	0.906	0.800
6680027	NP_032159.1#Glud1#14661# glutamate dehydrogenase 1, mitochondrial precursor [Mus musculus]	1.101	0.955	0.748	0.924	0.836	1.197	0.855	1.002	0.757	0.996	0.877
27552760	NP_613065.2#Tomm70a#28185# mitochondrial import receptor subunit TOM70 [Mus musculus]	1.108	0.939	0.755	0.917	0.836	1.196	0.851	1.026	0.770	0.983	0.876
568916013	XP_006499092.1#Scn1a#20265# PREDICTED: sodium channel protein type 1 subunit alpha isoform X3 [Mus musculus]	0.755	0.953	0.723	0.950	0.836	1.196	0.578	0.685	0.513	0.691	0.602
46195798	NP_031864.2#Ddost#13200# dolichyl-diphosphooligosaccharide--protein glycosyltransferase 48 kDa subunit precursor [Mus musculus]	1.016	0.867	0.750	0.922	0.836	1.196	0.758	0.941	0.640	0.833	0.736
50053703	NP_758465.2#Ppp1r9b#217124# neurabin-2 [Mus musculus]	1.108	0.820	0.733	0.939	0.836	1.196	0.880	1.049	0.666	0.943	0.804
163838648	NP_001106209.1#Atp5c1#11949# ATP synthase subunit gamma, mitochondrial isoform b [Mus musculus]	0.985	0.932	0.708	0.965	0.836	1.196	0.721	0.841	0.596	0.871	0.733
94721328	NP_038961.2#Vapa#30960# vesicle-associated membrane protein-associated protein A [Mus musculus]	1.142	0.902	0.749	0.924	0.836	1.195	0.871	1.070	0.800	1.019	0.909
84370256	NP_598530.2#Cyfip2#76884# cytoplasmic FMR1-interacting protein 2 [Mus musculus]	1.224	0.844	0.773	0.900	0.836	1.195	0.941	1.140	0.729	0.898	0.814
568980361	XP_006516277.1#2010107E04Rik#70257# PREDICTED: 6.8 kDa mitochondrial proteolipid isoform X1 [Mus musculus]	1.444	0.965	0.835	0.839	0.837	1.195	1.223	1.193	1.046	1.285	1.165
568930614	XP_006538626.1#Hmgcl#15356# PREDICTED: hydroxymethylglutaryl-CoA lyase, mitochondrial isoform X1 [Mus musculus]	1.108	0.907	0.723	0.951	0.837	1.195	0.897	0.961	0.701	0.940	0.821
31982186	NP_032643.2#Mdh2#17448# malate dehydrogenase, mitochondrial precursor [Mus musculus]	1.083	0.915	0.741	0.933	0.837	1.195	0.814	0.988	0.703	0.935	0.819
13385976	NP_080766.1#Mrpl19#56284# 39S ribosomal protein L19, mitochondrial [Mus musculus]	1.054	0.886	0.685	0.988	0.837	1.195	0.799	1.026	0.732	0.913	0.823
13386040	NP_080812.1#Atp5s#68055# ATP synthase subunit s, mitochondrial precursor [Mus musculus]	1.079	0.967	0.736	0.937	0.837	1.195	0.866	0.940	0.700	1.045	0.872
568991693	XP_006520665.1#Pfkm#18642# PREDICTED: ATP-dependent 6-phosphofructokinase, muscle type isoform X1 [Mus musculus]	1.070	0.900	0.734	0.940	0.837	1.195	0.821	0.931	0.684	0.882	0.783
18390323	NP_080973.1#Rab14#68365# ras-related protein Rab-14 [Mus musculus]	0.999	0.827	0.740	0.933	0.837	1.195	0.819	0.833	0.596	0.780	0.688
568967017	XP_006513447.1#Rab5b#19344# PREDICTED: ras-related protein Rab-5B isoform X1 [Mus musculus]	1.036	0.926	0.701	0.972	0.837	1.195	0.778	0.959	0.749	0.882	0.816
283945579	NP_001152987.1#H2afy#26914# core histone macro-H2A.1 isoform 4 [Mus musculus]	0.959	0.990	0.721	0.953	0.837	1.195	0.735	0.829	0.638	0.869	0.754
31981304	NP_038505.2#Atp6v0d1#11972# V-type proton ATPase subunit d 1 [Mus musculus]	1.109	0.815	0.751	0.923	0.837	1.195	0.826	0.965	0.638	0.779	0.708
58037267	NP_082235.1#Pdia6#71853# protein disulfide-isomerase A6 precursor [Mus musculus]	1.022	0.858	0.757	0.917	0.837	1.195	0.786	0.985	0.639	0.855	0.747
13385854	NP_080628.1#Ppid#67738# peptidyl-prolyl cis-trans isomerase D [Mus musculus]	1.074	0.924	0.750	0.924	0.837	1.195	0.829	1.001	0.709	0.936	0.822
568916080	XP_006499124.1#Ggt7#207182# PREDICTED: gamma-glutamyltransferase 7 isoform X4 [Mus musculus]	1.511	0.793	0.798	0.876	0.837	1.195	1.169	1.360	0.805	1.201	1.003
568960677	XP_006510842.1#Anp32a#11737# PREDICTED: acidic leucine-rich nuclear phosphoprotein 32 family member A isoform X1 [Mus musculus]	1.173	0.865	0.727	0.947	0.837	1.194	0.831	1.046	0.707	1.064	0.885
568944716	XP_006539535.1#Slc8a2#110891# PREDICTED: sodium/calcium exchanger 2 isoform X3 [Mus musculus]	1.092	0.849	0.749	0.925	0.837	1.194	0.810	0.990	0.730	0.921	0.826
568939485	XP_006505081.1#Ptprz1#19283# PREDICTED: receptor-type tyrosine-protein phosphatase zeta isoform X7 [Mus musculus]	0.923	0.992	0.722	0.952	0.837	1.194	0.713	0.855	0.714	0.868	0.791
33859640	NP_035658.1#Taldo1#21351# transaldolase [Mus musculus]	1.201	0.956	0.814	0.860	0.837	1.194	0.869	1.015	0.705	0.995	0.850
295054266	NP_001171138.1#Sptan1#20740# spectrin alpha chain, non-erythrocytic 1 isoform 2 [Mus musculus]	1.220	0.909	0.746	0.929	0.837	1.194	0.925	1.113	0.795	1.064	0.929
159110562	NP_032345.2#Icam5#15898# intercellular adhesion molecule 5 precursor [Mus musculus]	1.102	0.735	0.742	0.934	0.838	1.194	0.868	1.035	0.627	0.761	0.694
568965829	XP_006512996.1#Wasf1#83767# PREDICTED: wiskott-Aldrich syndrome protein family member 1 isoform X2 [Mus musculus]	1.100	0.864	0.708	0.967	0.838	1.194	0.809	1.006	0.665	0.916	0.790
56682931	NP_001003899.1#Tardbp#230908# TAR DNA-binding protein 43 isoform 3 [Mus musculus]	1.145	0.831	0.729	0.947	0.838	1.193	0.870	1.026	0.661	0.945	0.803
226442759	NP_001140159.1#Pip5k1c#18717# phosphatidylinositol 4-phosphate 5-kinase type-1 gamma isoform 2 [Mus musculus]	1.093	0.859	0.616	1.059	0.838	1.193	0.874	0.968	0.655	0.929	0.792
568917818	XP_006499965.1#Caprin1#53872# PREDICTED: caprin-1 isoform X1 [Mus musculus]	1.207	0.885	0.699	0.977	0.838	1.193	0.934	1.088	0.768	1.021	0.894
13928670	NP_075373.1#Vps35#65114# vacuolar protein sorting-associated protein 35 [Mus musculus]	1.175	0.831	0.754	0.922	0.838	1.193	0.879	1.048	0.698	0.902	0.800
27369581	NP_766024.1#Slc25a12#78830# calcium-binding mitochondrial carrier protein Aralar1 [Mus musculus]	1.168	0.909	0.755	0.921	0.838	1.193	0.887	1.066	0.792	0.972	0.882
237820660	NP_001153791.1#Ubr4#69116# E3 ubiquitin-protein ligase UBR4 [Mus musculus]	1.140	0.851	0.755	0.921	0.838	1.193	0.861	1.049	0.779	0.847	0.813
239051082	NP_001155090.1#Cul5#75717# cullin-5 isoform 2 [Mus musculus]	1.074	0.877	0.743	0.933	0.838	1.193	0.870	0.926	0.677	0.874	0.776
568991661	XP_006520649.1#Pcbp2#18521# PREDICTED: poly(rC)-binding protein 2 isoform X16 [Mus musculus]	1.276	0.813	0.732	0.945	0.838	1.193	1.034	1.154	0.788	1.019	0.903
46575788	NP_034155.2#Dcx#13193# neuronal migration protein doublecortin isoform c [Mus musculus]	1.076	0.927	0.751	0.926	0.838	1.193	0.804	1.001	0.675	0.999	0.837
124377998	NP_081204.2#Fuom#69064# fucose mutarotase isoform 1 [Mus musculus]	0.873	1.161	0.736	0.940	0.838	1.193	0.657	0.805	0.743	0.956	0.849
13507622	NP_109617.1#Sacm1l#83493# phosphatidylinositide phosphatase SAC1 [Mus musculus]	1.207	0.892	0.782	0.895	0.838	1.193	0.881	1.012	0.801	0.997	0.899
568963189	XP_006511905.1#Cpne4#74020# PREDICTED: copine-4 isoform X1 [Mus musculus]	1.152	0.899	0.718	0.959	0.838	1.193	0.857	1.074	0.754	0.982	0.868
253735731	NP_001156715.1#Fis1#66437# mitochondrial fission 1 protein isoform 2 [Mus musculus]	1.046	0.964	0.698	0.979	0.838	1.193	0.760	1.003	0.665	1.012	0.838
359807367	NP_001240812.1#Pkm#18746# pyruvate kinase PKM isoform M1 [Mus musculus]	1.100	0.841	0.756	0.921	0.839	1.193	0.850	0.966	0.727	0.937	0.832
568979363	XP_006515792.1#Ahsa1#217737# PREDICTED: activator of 90 kDa heat shock protein ATPase homolog 1 isoform X1 [Mus musculus]	1.083	0.877	0.720	0.957	0.839	1.193	0.864	0.953	0.693	0.903	0.798
7106439	NP_035785.1#Tubb5#22154# tubulin beta-5 chain [Mus musculus]	1.176	0.887	0.751	0.926	0.839	1.193	0.888	1.016	0.768	1.027	0.897
545746274	NP_001271118.1#Homer1#26556# homer protein homolog 1 isoform b [Mus musculus]	1.004	0.836	0.760	0.917	0.839	1.192	0.738	0.865	0.547	0.783	0.665
568987482	XP_006518982.1#Timm8a2#223262# PREDICTED: putative mitochondrial import inner membrane translocase subunit Tim8 A-B isoform X1 [Mus mus	1.208	0.845	0.746	0.931	0.839	1.192	0.930	1.096	0.697	1.015	0.856
226874952	NP_032270.3#Hgs#15239# hepatocyte growth factor-regulated tyrosine kinase substrate isoform 2 [Mus musculus]	1.038	0.939	0.722	0.956	0.839	1.192	0.784	0.956	0.683	0.952	0.818
126521835	NP_031662.2#Cct2#12461# T-complex protein 1 subunit beta [Mus musculus]	1.082	0.935	0.737	0.941	0.839	1.192	0.848	0.999	0.728	0.974	0.851
9789991	NP_062631.1#Hsd17b12#56348# very-long-chain 3-oxoacyl-CoA reductase [Mus musculus]	1.013	0.882	0.714	0.964	0.839	1.192	0.800	0.900	0.663	0.847	0.755
9789997	NP_062668.1#Letm1#56384# LETM1 and EF-hand domain-containing protein 1, mitochondrial precursor [Mus musculus]	1.179	0.917	0.704	0.975	0.839	1.192	0.854	1.133	0.786	1.041	0.914

568904377	XP_006544804.1#LOC102642137#102642137# PREDICTED: 40S ribosomal protein S13 [Mus musculus]	1.029	0.880	0.731	0.948	0.839	1.192	0.787	0.938	0.636	0.857	0.746
755548661	XP_006519417.2#Nisch#64652# PREDICTED: nischarin isoform X2 [Mus musculus]	1.150	0.771	0.717	0.962	0.839	1.191	0.818	1.112	0.697	0.791	0.744
45504359	NP_031536.2#Atp6v1e1#11973# V-type proton ATPase subunit E 1 [Mus musculus]	1.069	0.887	0.743	0.936	0.840	1.191	0.904	1.017	0.707	0.936	0.821
13385322	NP_080119.1#Ndufb7#66916# NADH dehydrogenase [ubiquinone] 1 beta subcomplex subunit 7 [Mus musculus]	1.199	0.914	0.729	0.951	0.840	1.191	0.877	1.070	0.758	1.027	0.892
755495690	XP_011237111.1#Rgs7#24012# PREDICTED: regulator of G-protein signaling 7 isoform X7 [Mus musculus]	1.026	0.772	0.751	0.928	0.840	1.191	0.795	0.929	0.594	0.736	0.665
568999008	XP_006523719.1#Flot1#14251# PREDICTED: flotillin-1 isoform X1 [Mus musculus]	1.064	0.846	0.736	0.943	0.840	1.191	0.774	0.904	0.628	0.815	0.721
33859482	NP_031933.1#Eef2#13629# elongation factor 2 [Mus musculus]	1.180	0.912	0.740	0.940	0.840	1.191	0.930	1.086	0.755	1.003	0.879
13386054	NP_080828.1#Arpc4#68089# actin-related protein 2/3 complex subunit 4 isoform 1 [Mus musculus]	1.057	0.796	0.748	0.932	0.840	1.191	0.695	0.899	0.518	0.722	0.620
22550094	NP_032950.1#Prkar2a#19087# cAMP-dependent protein kinase type II-alpha regulatory subunit [Mus musculus]	1.079	0.868	0.746	0.934	0.840	1.191	0.812	0.966	0.693	0.894	0.793
13385084	NP_079899.1#Nipsnap3b#66536# protein NipSnap homolog 3B [Mus musculus]	1.266	0.839	0.777	0.903	0.840	1.191	0.925	1.202	0.746	1.039	0.892
110625624	NP_038714.2#Tcp1#21454# T-complex protein 1 subunit alpha isoform 1 [Mus musculus]	1.074	0.886	0.744	0.936	0.840	1.190	0.818	0.971	0.698	0.883	0.790
93102409	NP_032014.3#Fasn#14104# fatty acid synthase [Mus musculus]	1.215	0.944	0.740	0.940	0.840	1.190	0.915	1.056	0.808	0.997	0.902
722828890	NP_001289728.1#Rab27b#80718# ras-related protein Rab-27B isoform 2 [Mus musculus]	1.360	0.891	0.727	0.953	0.840	1.190	1.022	1.264	0.909	1.127	1.018
21704140	NP_663542.1#Hibadh#58875# 3-hydroxyisobutyrate dehydrogenase, mitochondrial precursor [Mus musculus]	0.926	0.893	0.689	0.991	0.840	1.190	0.700	0.855	0.626	0.763	0.695
260166642	NP_001159374.1#Ctnnb1#12387# catenin beta-1 [Mus musculus]	1.121	0.834	0.744	0.936	0.840	1.190	0.844	1.033	0.692	0.918	0.805
568958962	XP_006510130.1#Ncam1#17967# PREDICTED: neural cell adhesion molecule 1 isoform X13 [Mus musculus]	1.010	0.986	0.747	0.934	0.840	1.190	0.775	0.923	0.741	0.932	0.837
167716840	YP_001686701.1#COX2#5912281# cytochrome c oxidase subunit II [Mus musculus musculus]	0.883	0.950	0.713	0.968	0.841	1.190	0.670	0.800	0.601	0.845	0.723
225579033	NP_766599.2#Idh2#269951# isocitrate dehydrogenase [NADP], mitochondrial precursor [Mus musculus]	1.122	0.883	0.721	0.961	0.841	1.190	0.849	1.073	0.695	0.964	0.829
568986360	XP_006518440.1#Mbnl2#105559# PREDICTED: muscleblind-like protein 2 isoform X7 [Mus musculus]	1.111	0.916	0.775	0.906	0.841	1.190	0.839	1.029	0.725	0.986	0.855
33859662	NP_036167.1#Vat1#26949# synaptic vesicle membrane protein VAT-1 homolog [Mus musculus]	1.065	0.939	0.746	0.935	0.841	1.190	0.820	0.964	0.755	0.936	0.845
6753322	NP_033967.1#Cct4#12464# T-complex protein 1 subunit delta [Mus musculus]	1.110	0.907	0.750	0.931	0.841	1.190	0.840	1.006	0.689	0.966	0.827
46430508	NP_997406.1#Rpl23a#268449# 60S ribosomal protein L23a [Mus musculus]	1.030	0.927	0.702	0.979	0.841	1.189	0.769	0.963	0.708	0.897	0.803
569003865	XP_006525999.1#Pcyox1l#240334# PREDICTED: prenylcysteine oxidase-like isoform X1 [Mus musculus]	1.014	0.849	0.731	0.951	0.841	1.189	0.800	0.966	0.649	0.839	0.744
114158683	NP_001041473.1#Agtpbp1#67269# cytosolic carboxypeptidase 1 isoform 2 [Mus musculus]	1.035	0.869	0.759	0.923	0.841	1.189	0.782	0.959	0.639	0.873	0.756
459683847	NP_001264043.1#Rpl22#19934# 60S ribosomal protein L22 isoform c [Mus musculus]	0.912	0.849	0.740	0.942	0.841	1.189	0.731	0.802	0.572	0.730	0.651
21489933	NP_036082.1#Mapk3#26417# mitogen-activated protein kinase 3 [Mus musculus]	1.110	0.889	0.740	0.943	0.841	1.189	0.830	1.037	0.694	0.965	0.830
160298209	NP_034454.2#Got1#14718# aspartate aminotransferase, cytoplasmic [Mus musculus]	1.018	0.940	0.768	0.915	0.841	1.189	0.782	0.967	0.701	0.959	0.830
254588014	NP_080070.2#Etfdh#66841# electron transfer flavoprotein-ubiquinone oxidoreductase, mitochondrial precursor [Mus musculus]	1.358	0.790	0.697	0.985	0.841	1.189	1.002	1.283	0.732	1.073	0.902
31980648	NP_058054.2#Atp5b#11947# ATP synthase subunit beta, mitochondrial precursor [Mus musculus]	1.017	0.932	0.751	0.932	0.841	1.189	0.789	0.941	0.703	0.908	0.806
568994456	XP_006521777.1#Alcam#11658# PREDICTED: CD166 antigen isoform X1 [Mus musculus]	1.074	0.994	0.737	0.946	0.841	1.188	0.793	0.927	0.740	0.951	0.845
126723336	NP_031557.2#Phb2#12034# prohibitin-2 [Mus musculus]	1.102	0.895	0.746	0.937	0.841	1.188	0.853	0.976	0.691	0.923	0.807
568984189	XP_006517578.1#Scamp1#107767# PREDICTED: secretory carrier-associated membrane protein 1 isoform X1 [Mus musculus]	1.220	0.805	0.791	0.892	0.842	1.188	0.929	1.374	0.791	1.008	0.899
568952615	XP_006508536.1#Cttn#13043# PREDICTED: src substrate cortactin isoform X1 [Mus musculus]	0.991	0.815	0.754	0.930	0.842	1.188	0.772	0.898	0.594	0.767	0.681
568953607	XP_006508997.1#Cul4a#99375# PREDICTED: cullin-4A isoform X2 [Mus musculus]	0.821	0.803	0.820	0.864	0.842	1.187	0.600	0.783	0.463	0.647	0.555
568904643	XP_006544594.1#LOC102642619#102642619# PREDICTED: peroxiredoxin-6 [Mus musculus]	1.076	0.950	0.761	0.923	0.842	1.187	0.820	0.990	0.730	0.989	0.859
266458391	NP_067259.4#Kras#16653# GTPase KRas [Mus musculus]	0.961	0.906	0.737	0.948	0.843	1.187	0.715	0.895	0.589	0.798	0.693
14192922	NP_033738.1#Actc1#11464# actin, alpha cardiac muscle 1 [Mus musculus]	1.229	0.862	0.734	0.951	0.843	1.187	0.980	1.167	0.732	1.063	0.897
9790055	NP_062732.1#Mtch2#56428# mitochondrial carrier homolog 2 [Mus musculus]	1.019	0.912	0.761	0.925	0.843	1.187	0.733	0.983	0.698	0.867	0.783
261824055	NP_001159840.1#Fkbp2#14227# peptidyl-prolyl cis-trans isomerase FKBP2 precursor [Mus musculus]	1.004	0.959	0.743	0.942	0.843	1.187	0.754	0.939	0.667	0.956	0.811
568906168	XP_006495948.1#Tpp2#22019# PREDICTED: tripeptidyl-peptidase 2 isoform X1 [Mus musculus]	1.239	0.882	0.764	0.921	0.843	1.187	0.907	1.213	0.835	1.042	0.938
6754004	NP_034431.1#Gna11#14672# guanine nucleotide-binding protein subunit alpha-11 [Mus musculus]	1.211	0.873	0.756	0.930	0.843	1.186	0.886	1.102	0.772	0.955	0.864
755554060	XP_011244319.1#Bdh1#71911# PREDICTED: D-beta-hydroxybutyrate dehydrogenase, mitochondrial isoform X1 [Mus musculus]	1.113	0.879	0.724	0.962	0.843	1.186	0.886	0.980	0.679	0.885	0.782
18250284	NP_083849.1#Idh3a#67834# isocitrate dehydrogenase [NAD] subunit alpha, mitochondrial precursor [Mus musculus]	1.047	0.905	0.744	0.942	0.843	1.186	0.827	0.999	0.718	0.951	0.834
568968072	XP_006513956.1#Nap1l1#53605# PREDICTED: nucleosome assembly protein 1-like 1 isoform X4 [Mus musculus]	1.084	0.968	0.744	0.942	0.843	1.186	0.867	0.962	0.756	1.014	0.885
56090141	NP_001007597.1#Rtn1#104001# reticulon-1 isoform C [Mus musculus]	1.038	0.826	0.751	0.935	0.843	1.186	0.779	0.946	0.622	0.740	0.681
568987554	XP_006519014.1#Ogdhl#239017# PREDICTED: 2-oxoglutarate dehydrogenase-like, mitochondrial isoform X1 [Mus musculus]	1.168	0.923	0.744	0.942	0.843	1.186	0.876	1.140	0.800	1.086	0.943
568912868	XP_006497711.1#Crat#12908# PREDICTED: carnitine O-acetyltransferase isoform X3 [Mus musculus]	1.148	0.904	0.735	0.952	0.843	1.186	0.868	1.069	0.715	1.035	0.875
22267442	NP_080175.1#Uqcrc2#67003# cytochrome b-c1 complex subunit 2, mitochondrial precursor [Mus musculus]	1.089	0.917	0.739	0.948	0.843	1.186	0.827	1.014	0.763	0.952	0.858
359279952	NP_001240685.1#Gpm6a#234267# neuronal membrane glycoprotein M6-a isoform 3 [Mus musculus]	0.848	0.887	0.761	0.926	0.843	1.186	0.627	0.808	0.588	0.703	0.645
31543942	NP_033528.3#Vcl#22330# vinculin [Mus musculus]	1.063	0.959	0.782	0.904	0.843	1.186	0.846	0.947	0.723	0.996	0.860
21312594	NP_081706.1#Mpc2#70456# mitochondrial pyruvate carrier 2 [Mus musculus]	0.944	0.946	0.773	0.914	0.844	1.185	0.774	0.844	0.644	0.853	0.749
121674797	NP_032943.2#Ppt1#19063# palmitoyl-protein thioesterase 1 precursor [Mus musculus]	1.089	0.951	0.731	0.956	0.844	1.185	0.837	1.000	0.735	1.011	0.873
597517936	NP_001277985.1#Snap25#20614# synaptosomal-associated protein 25 isoform b [Mus musculus]	1.165	0.866	0.730	0.957	0.844	1.185	0.924	1.099	0.764	1.000	0.882
14389431	NP_058017.1#Stip1#20867# stress-induced-phosphoprotein 1 [Mus musculus]	1.107	0.952	0.724	0.963	0.844	1.185	0.819	1.006	0.709	0.978	0.843
6754254	NP_034610.1#Hsp90aa1#15519# heat shock protein HSP 90-alpha [Mus musculus]	1.059	0.907	0.760	0.928	0.844	1.185	0.829	0.999	0.745	0.967	0.856
84452155	NP_001033678.1#Usp14#59025# ubiquitin carboxyl-terminal hydrolase 14 isoform 2 [Mus musculus]	1.018	0.917	0.739	0.950	0.844	1.185	0.713	1.006	0.703	0.872	0.788
256818746	NP_001157960.1#Wars#22375# tryptophan--tRNA ligase, cytoplasmic isoform 2 [Mus musculus]	0.737	0.895	0.735	0.953	0.844	1.185	0.588	0.657	0.506	0.608	0.557
37574048	NP_932096.1#Uqcr10#66152# cytochrome b-c1 complex subunit 9 [Mus musculus]	1.056	0.926	0.714	0.974	0.844	1.185	0.797	0.987	0.735	0.917	0.826
35215309	NP_874385.1#Nfasc#269116# neurofascin isoform 1 precursor [Mus musculus]	1.295	0.878	0.681	1.008	0.844	1.184	1.040	1.147	0.835	1.086	0.961
755528368	XP_011240701.1#Elavl3#15571# PREDICTED: ELAV-like protein 3 isoform X3 [Mus musculus]	1.210	0.869	0.700	0.989	0.844	1.184	0.956	1.088	0.767	1.009	0.888
31982393	NP_031966.2#Ephx2#13850# bifunctional epoxide hydrolase 2 isoform a [Mus musculus]	1.120	0.960	0.792	0.897	0.844	1.184	0.862	1.031	0.781	0.973	0.877
10946574	NP_067248.1#Ckb#12709# creatine kinase B-type [Mus musculus]	1.079	0.934	0.763	0.926	0.845	1.184	0.824	0.996	0.742	0.940	0.841
254540027	NP_032644.3#Mdh1#17449# malate dehydrogenase, cytoplasmic [Mus musculus]	0.970	0.926	0.761	0.928	0.845	1.184	0.748	0.913	0.638	0.927	0.782
194473613	NP_001123957.1#Rpl35a#57808# 60S ribosomal protein L35a [Mus musculus]	0.716	1.013	0.714	0.975	0.845	1.184	0.534	0.675	0.507	0.717	0.612
808688278	NP_001294969.1#Ttyh3#78339# protein tweety homolog 3 isoform 3 [Mus musculus]	1.308	0.782	0.789	0.900	0.845	1.184	1.099	1.110	0.685	1.044	0.864
31542413	NP_035909.2#Coro1c#23790# coronin-1C [Mus musculus]	1.040	0.877	0.752	0.937	0.845	1.184	0.727	0.942	0.634	0.844	0.739
110625948	NP_694791.2#Acat3#224530# acetyl-Coenzyme A acetyltransferase 3 [Mus musculus]	1.128	0.931	0.780	0.910	0.845	1.184	0.837	1.120	0.779	1.019	0.899
569009753	XP_006527907.1#Idh3g#15929# PREDICTED: isocitrate dehydrogenase [NAD] subunit gamma 1, mitochondrial isoform X2 [Mus musculus]	1.069	0.957	0.763	0.927	0.845	1.184	0.833	1.060	0.743	0.986	0.865
6680598	NP_032493.1#Kpna4#16649# importin subunit alpha-3 [Mus musculus]	1.518	0.832	0.748	0.942	0.845	1.184	1.149	1.416	0.949	1.187	1.068
755523881	XP_006536234.2#Pddc1#213350# PREDICTED: Parkinson disease 7 domain-containing protein 1 isoform X1 [Mus musculus]	1.099	0.878	0.745	0.945	0.845	1.183	0.804	1.053	0.685	0.945	0.815
19527258	NP_598803.1#Aldh6a1#104776# methylmalonate-semialdehyde dehydrogenase [acylating], mitochondrial precursor [Mus musculus]	1.119	0.924	0.737	0.953	0.845	1.183	0.868	1.062	0.710	0.950	0.830
34098931	NP_031612.1#Calb2#12308# calretinin [Mus musculus]	0.798	1.202	0.751	0.939	0.845	1.183	0.659	0.760	0.678	0.951	0.814
111038118	NP_032238.2#Hadh#15107# hydroxyacyl-coenzyme A dehydrogenase, mitochondrial precursor [Mus musculus]	1.287	0.948	0.791	0.899	0.845	1.183	0.987	1.175	0.832	1.112	0.972
254281331	NP_061349.3#Slc1a4#55963# neutral amino acid transporter A [Mus musculus]	0.990	0.948	0.763	0.927	0.845	1.183	0.751	0.872	0.718	0.854	0.786
357394934	NP_001239447.1#Nono#53610# non-POU domain-containing octamer-binding protein [Mus musculus]	1.081	0.933	0.705	0.986	0.845	1.183	0.702	1.093	0.801	0.897	0.849
568945132	XP_006539736.1#Rab4b#19342# PREDICTED: ras-related protein Rab-4B isoform X2 [Mus musculus]	1.094	0.766	0.719	0.972	0.846	1.183	0.979	0.871	0.648	0.769	0.708
755519194	XP_011248758.1#Pld3#18807# PREDICTED: phospholipase D3 isoform X3 [Mus musculus]	1.062	0.797	0.728	0.963	0.846	1.183	0.840	0.979	0.612	0.852	0.732
110625902	NP_082293.1#Napg#108123# gamma-soluble NSF attachment protein [Mus musculus]	1.170	0.916	0.738	0.954	0.846	1.182	0.926	1.068	0.789	1.049	0.919
6753324	NP_033968.1#Cct6a#12466# T-complex protein 1 subunit zeta [Mus musculus]	1.054	0.927	0.769	0.922	0.846	1.182	0.797	0.989	0.722	0.880	0.801
18079339	NP_542364.1#Aco2#11429# aconitate hydratase, mitochondrial precursor [Mus musculus]	1.200	0.941	0.764	0.927	0.846	1.182	0.913	1.114	0.839	1.096	0.967
568994470	XP_006521784.1#Atp6v1a#11964# PREDICTED: V-type proton ATPase catalytic subunit A isoform X1 [Mus musculus]	1.077	0.836	0.739	0.952	0.846	1.182	0.827	0.975	0.686	0.910	0.798
87116677	NP_001034572.1#Tmco1#68944# transmembrane and coiled-coil domain-containing protein 1 precursor [Mus musculus]	1.018	0.925	0.772	0.920	0.846	1.182	0.776	0.947	0.723	0.872	0.797
568946338	XP_006540318.1#Actn4#60595# PREDICTED: alpha-actinin-4 isoform X2 [Mus musculus]	1.072	0.850	0.783	0.909	0.846	1.182	0.873	0.948	0.697	0.854	0.775

568911813	XP_006497279.1#Rab3gap2#98732# PREDICTED: rab3 GTPase-activating protein non-catalytic subunit isoform X1 [Mus musculus]	1.101	0.852	0.719	0.973	0.846	1.182	0.876	0.993	0.690	0.902	0.796
60097931	NP_084248.2#Ermn#77767# ermin [Mus musculus]	1.211	1.390	0.719	0.974	0.846	1.182	0.989	1.060	1.226	1.623	1.424
325910859	NP_001191804.1#Set#56086# protein SET isoform 2 [Mus musculus]	0.991	0.903	0.731	0.961	0.846	1.182	0.769	0.897	0.651	0.873	0.762
161086902	NP_001104316.1#Cacna2d1#12293# voltage-dependent calcium channel subunit alpha-2/delta-1 isoform d preproprotein [Mus musculus]	1.049	0.930	0.772	0.921	0.846	1.182	0.864	0.963	0.668	0.935	0.802
152	TRYP_PIG	1.001	0.947	0.750	0.942	0.846	1.182	0.742	0.888	0.679	0.886	0.783
803378413	NP_001292773.1#Apoe#11816# apolipoprotein E precursor [Mus musculus]	1.113	0.888	0.794	0.899	0.846	1.181	0.869	0.993	0.770	0.893	0.831
755497436	XP_011237380.1#Sh3glb2#227700# PREDICTED: endophilin-B2 isoform X6 [Mus musculus]	1.098	0.856	0.734	0.959	0.847	1.181	0.832	1.029	0.678	0.940	0.809
7305581	NP_038923.1#Timm13#30055# mitochondrial import inner membrane translocase subunit Tim13 [Mus musculus]	1.032	0.913	0.717	0.976	0.847	1.181	0.855	1.067	0.802	1.017	0.909
42415475	NP_035162.1#P4hb#18453# protein disulfide-isomerase precursor [Mus musculus]	1.072	1.004	0.757	0.936	0.847	1.181	0.821	0.941	0.743	0.983	0.863
61888838	NP_058043.3#Hsd17b10#15108# 3-hydroxyacyl-CoA dehydrogenase type-2 [Mus musculus]	1.132	0.879	0.768	0.925	0.847	1.181	1.021	1.041	0.782	1.062	0.922
30794138	NP_082568.1#Ppme1#72590# protein phosphatase methylesterase 1 [Mus musculus]	1.094	0.825	0.729	0.964	0.847	1.181	0.877	1.016	0.655	0.836	0.745
7305521	NP_038789.1#Srr#27364# serine racemase [Mus musculus]	1.058	0.816	0.841	0.852	0.847	1.181	0.778	0.968	0.613	0.774	0.693
23956214	NP_076092.1#Sfpq#71514# splicing factor, proline- and glutamine-rich [Mus musculus]	1.015	0.837	0.730	0.964	0.847	1.181	0.799	1.000	0.680	0.840	0.760
31542273	NP_666269.2#Emc1#230866# ER membrane protein complex subunit 1 isoform 1 precursor [Mus musculus]	1.149	0.910	0.778	0.917	0.847	1.180	0.905	1.042	0.750	1.022	0.886
11528518	NP_071313.1#Sv2a#64051# synaptic vesicle glycoprotein 2A [Mus musculus]	1.053	0.902	0.742	0.952	0.847	1.180	0.803	0.959	0.716	0.888	0.802
568960899	XP_006510947.1#Nptn#20320# PREDICTED: neuroplastin isoform X2 [Mus musculus]	1.078	0.846	0.749	0.946	0.847	1.180	0.875	1.007	0.685	0.889	0.787
6755114	NP_036151.1#Prdx5#54683# peroxiredoxin-5, mitochondrial precursor [Mus musculus]	1.055	0.944	0.740	0.955	0.847	1.180	0.819	0.974	0.732	1.018	0.875
568922710	XP_006501503.1#Ak5#229949# PREDICTED: adenylate kinase isoenzyme 5 isoform X2 [Mus musculus]	0.958	0.901	0.787	0.907	0.847	1.180	0.743	0.881	0.639	0.823	0.731
31560656	NP_032800.2#Pabpc1#18458# polyadenylate-binding protein 1 [Mus musculus]	1.000	0.907	0.710	0.985	0.847	1.180	0.762	0.807	0.632	0.763	0.697
755565294	XP_011245855.1#Rps4x#20102# PREDICTED: 40S ribosomal protein S4, X isoform isoform X1 [Mus musculus]	1.068	0.873	0.787	0.908	0.847	1.180	0.815	0.964	0.698	0.904	0.801
311771690	NP_001185720.1#Ak1#11636# adenylate kinase isoenzyme 1 isoform 2 [Mus musculus]	1.088	0.891	0.739	0.956	0.847	1.180	0.810	1.054	0.701	0.968	0.834
568951405	XP_006508268.1#Pgm2l1#70974# PREDICTED: glucose 1,6-bisphosphate synthase isoform X1 [Mus musculus]	1.121	0.902	0.744	0.951	0.847	1.180	0.856	1.020	0.710	1.018	0.864
568981400	XP_006516550.1#Pfkp#56421# PREDICTED: ATP-dependent 6-phosphofructokinase, platelet type isoform X1 [Mus musculus]	1.110	0.880	0.755	0.940	0.847	1.180	0.859	1.037	0.711	0.938	0.824
568921291	XP_006500830.1#Hspa4l#18415# PREDICTED: heat shock 70 kDa protein 4L isoform X1 [Mus musculus]	1.154	0.905	0.730	0.965	0.848	1.180	0.863	1.087	0.786	1.028	0.907
6755304	NP_035386.1#Pitpnm2#19679# membrane-associated phosphatidylinositol transfer protein 2 isoform 1 [Mus musculus]	1.116	0.889	0.767	0.928	0.848	1.180	0.834	1.058	0.747	0.935	0.841
312433957	NP_001186066.1#Macf1#11426# microtubule-actin cross-linking factor 1 isoform 2 [Mus musculus]	0.972	0.899	0.775	0.921	0.848	1.180	0.770	0.878	0.628	0.854	0.741
9790019	NP_062708.1#Asah1#11886# acid ceramidase precursor [Mus musculus]	1.001	0.901	0.751	0.945	0.848	1.179	0.789	0.904	0.638	0.893	0.766
6755566	NP_036160.1#Slc9a3r1#26941# Na(+)/H(+) exchange regulatory cofactor NHE-RF1 [Mus musculus]	1.112	0.928	0.749	0.947	0.848	1.179	0.867	1.041	0.732	1.017	0.874
755522062	XP_011239970.1#Tpp1#12751# PREDICTED: tripeptidyl-peptidase 1 isoform X2 [Mus musculus]	1.286	0.840	0.699	0.997	0.848	1.179	0.965	1.215	0.776	1.056	0.916
568910562	XP_006496765.1#Pea15a#18611# PREDICTED: astrocytic phosphoprotein PEA-15 isoform X1 [Mus musculus]	0.948	0.924	0.755	0.941	0.848	1.179	0.712	0.905	0.651	0.856	0.754
7106301	NP_031922.1#Mapre1#13589# microtubule-associated protein RP/EB family member 1 [Mus musculus]	1.064	0.947	0.712	0.984	0.848	1.179	0.843	0.962	0.700	1.009	0.855
116174793	NP_115999.2#Sptbn4#80297# spectrin beta chain, non-erythrocytic 4 isoform sigma1 [Mus musculus]	1.059	0.940	0.769	0.927	0.848	1.179	0.826	0.969	0.723	0.967	0.845
148747424	NP_031476.3#Slc25a4#11739# ADP/ATP translocase 1 [Mus musculus]	1.129	0.910	0.742	0.955	0.848	1.179	0.883	1.031	0.744	1.010	0.877
162461907	NP_034611.2#Hspa9#15526# stress-70 protein, mitochondrial [Mus musculus]	1.168	0.925	0.760	0.937	0.848	1.179	0.885	1.110	0.791	1.060	0.926
148747558	NP_035693.3#Prdx2#21672# peroxiredoxin-2 [Mus musculus]	0.831	0.938	0.728	0.969	0.848	1.179	0.643	0.766	0.567	0.702	0.634
170650601	NP_598908.2#Macrod1#107227# O-acetyl-ADP-ribose deacetylase MACROD1 [Mus musculus]	1.323	0.959	0.800	0.897	0.848	1.179	1.043	1.203	0.989	1.164	1.077
158635979	NP_001103610.1#Atp2a2#11938# sarcoplasmic/endoplasmic reticulum calcium ATPase 2 isoform b [Mus musculus]	1.176	0.830	0.772	0.925	0.848	1.179	0.954	1.086	0.678	0.918	0.798
568908107	XP_006529184.1#Ecel1#13599# PREDICTED: endothelin-converting enzyme-like 1 isoform X1 [Mus musculus]	0.920	0.747	0.737	0.960	0.849	1.179	0.673	0.888	0.505	0.659	0.582
755553212	XP_011244136.1#Kpna1#16646# PREDICTED: importin subunit alpha-5 isoform X2 [Mus musculus]	1.137	0.909	0.807	0.890	0.849	1.178	0.932	1.001	0.839	0.916	0.878
6680908	NP_031694.1#Cdk5#12568# cyclin-dependent kinase 5 [Mus musculus]	1.311	0.895	0.768	0.929	0.849	1.178	0.951	1.226	0.797	1.022	0.910
29893569	NP_775539.1#Eif5#217869# eukaryotic translation initiation factor 5 [Mus musculus]	1.113	0.919	0.761	0.937	0.849	1.178	0.896	1.040	0.788	1.019	0.904
42415473	NP_032939.1#Ppp3ca#19055# serine/threonine-protein phosphatase 2B catalytic subunit alpha isoform isoform 1 [Mus musculus]	1.159	0.844	0.742	0.956	0.849	1.178	0.881	1.086	0.678	0.933	0.806
568915235	XP_006498719.1#Ckmt1#12716# PREDICTED: creatine kinase U-type, mitochondrial isoform X1 [Mus musculus]	1.201	0.922	0.733	0.965	0.849	1.178	0.928	1.112	0.818	1.057	0.938
33859751	NP_077774.1#Rab21#216344# ras-related protein Rab-21 [Mus musculus]	1.046	0.932	0.722	0.976	0.849	1.178	0.800	0.938	0.722	0.895	0.808
93102415	NP_034386.2#Gart#14450# trifunctional purine biosynthetic protein adenosine-3 [Mus musculus]	1.169	0.936	0.785	0.913	0.849	1.177	0.961	1.048	0.874	1.137	1.006
23956084	NP_059062.1#Acadvl#11370# very long-chain specific acyl-CoA dehydrogenase, mitochondrial precursor [Mus musculus]	1.070	0.956	0.770	0.929	0.849	1.177	0.773	0.932	0.614	0.827	0.720
84794597	NP_077779.2#Ppp3r1#19058# calcineurin subunit B type 1 [Mus musculus]	1.243	0.842	0.760	0.939	0.849	1.177	0.920	0.995	0.670	1.000	0.835
568968863	XP_006514333.1#Ndufs7#75406# PREDICTED: NADH dehydrogenase [ubiquinone] iron-sulfur protein 7, mitochondrial isoform X1 [Mus musculus]	1.148	0.998	0.728	0.971	0.850	1.177	0.859	0.992	0.782	1.078	0.930
6678437	NP_033455.1#Tpt1#22070# translationally-controlled tumor protein [Mus musculus]	1.269	0.914	0.751	0.948	0.850	1.177	1.008	1.155	0.819	1.097	0.958
6679158	NP_032775.1#Nucb1#18220# nucleobindin-1 isoform 2 precursor [Mus musculus]	1.086	0.953	0.718	0.981	0.850	1.177	0.824	0.956	0.693	0.996	0.844
254540168	NP_001156906.1#Hspa5#14828# 78 kDa glucose-regulated protein precursor [Mus musculus]	1.049	0.934	0.756	0.943	0.850	1.177	0.823	0.979	0.700	0.967	0.833
568935980	XP_006535170.1#Sept11#52398# PREDICTED: septin-11 isoform X9 [Mus musculus]	1.054	0.921	0.743	0.956	0.850	1.177	0.729	1.062	0.744	0.905	0.825
443287660	NP_001263208.1#Cyld#74256# ubiquitin carboxyl-terminal hydrolase CYLD isoform c [Mus musculus]	1.152	0.831	0.707	0.992	0.850	1.177	0.891	1.066	0.680	0.947	0.813
568935633	XP_006535002.1#2900026A02Rik#243219# PREDICTED: uncharacterized protein KIAA1671 isoform X2 [Mus musculus]	1.034	0.825	0.754	0.945	0.850	1.177	0.797	0.961	0.628	0.821	0.725
569011801	XP_006528817.1#Pak3#18481# PREDICTED: serine/threonine-protein kinase PAK 3 isoform X3 [Mus musculus]	1.123	0.998	0.751	0.948	0.850	1.177	0.865	1.042	0.851	1.052	0.952
19882207	NP_608219.1#Fdps#110196# farnesyl pyrophosphate synthase isoform 2 [Mus musculus]	0.941	0.816	0.730	0.970	0.850	1.177	0.705	0.892	0.553	0.751	0.652
148747526	NP_033031.2#Rab7#19349# ras-related protein Rab-7a [Mus musculus]	0.990	0.889	0.746	0.954	0.850	1.177	0.753	0.935	0.620	0.820	0.720
557636704	NP_001273537.1#Banf1#23825# barrier-to-autointegration factor [Mus musculus]	0.865	0.924	0.770	0.930	0.850	1.177	0.680	0.791	0.577	0.783	0.680
444299620	NP_001263245.1#Uba1#22201# ubiquitin-like modifier-activating enzyme 1 isoform 2 [Mus musculus]	1.141	0.901	0.771	0.929	0.850	1.176	0.899	1.046	0.758	0.961	0.860
755560616	XP_011245163.1#Rab18#19330# PREDICTED: ras-related protein Rab-18 isoform X1 [Mus musculus]	0.977	0.759	0.691	1.010	0.850	1.176	0.801	0.862	0.529	0.732	0.631
148747410	NP_666097.3#Arcn1#213827# coatomer subunit delta [Mus musculus]	1.056	0.912	0.736	0.964	0.850	1.176	0.839	1.042	0.681	0.955	0.818
568985464	XP_006518099.1#Cadps#27062# PREDICTED: calcium-dependent secretion activator 1 isoform X13 [Mus musculus]	1.260	0.909	0.775	0.926	0.850	1.176	1.025	1.209	0.830	1.141	0.986
755547555	XP_011243186.1#Exoc5#105504# PREDICTED: exocyst complex component 5 isoform X2 [Mus musculus]	1.084	0.959	0.754	0.947	0.850	1.176	0.823	1.020	0.800	0.968	0.884
183396771	NP_034607.3#Hspd1#15510# 60 kDa heat shock protein, mitochondrial [Mus musculus]	1.072	0.919	0.754	0.947	0.850	1.176	0.810	0.963	0.703	0.939	0.821
568942796	XP_006506634.1#Agk#69923# PREDICTED: acylglycerol kinase, mitochondrial isoform X2 [Mus musculus]	1.136	0.943	0.757	0.944	0.850	1.176	0.768	1.094	0.717	1.016	0.866
160333216	NP_001103970.1#Canx#12330# calnexin precursor [Mus musculus]	1.078	0.941	0.730	0.971	0.850	1.176	0.853	0.995	0.709	1.000	0.855
344217717	NP_001230686.1#Pde2a#207728# cGMP-dependent 3',5'-cyclic phosphodiesterase isoform PDE2A1 [Mus musculus]	1.199	0.882	0.746	0.955	0.850	1.176	0.927	1.108	0.755	1.009	0.882
755544665	XP_011242675.1#Sfxn1#14057# PREDICTED: sideroflexin-1 isoform X2 [Mus musculus]	1.000	0.934	0.740	0.961	0.850	1.176	0.747	0.888	0.695	0.837	0.766
291045426	NP_001166977.1#Map1a#17754# microtubule-associated protein 1A isoform 2 [Mus musculus]	1.159	0.884	0.751	0.950	0.850	1.176	0.893	1.092	0.734	0.992	0.863
40068507	NP_031791.3#Crmp1#12933# dihydropyrimidinase-related protein 1 isoform 2 [Mus musculus]	1.027	0.875	0.751	0.950	0.851	1.176	0.763	0.990	0.625	0.829	0.727
68226731	NP_032968.2#Npepps#19155# puromycin-sensitive aminopeptidase [Mus musculus]	1.059	0.908	0.777	0.924	0.851	1.176	0.758	0.979	0.664	0.991	0.827
568960787	XP_006510895.1#Myo5a#17918# PREDICTED: unconventional myosin-Va isoform X6 [Mus musculus]	1.176	0.900	0.751	0.950	0.851	1.176	0.904	1.107	0.814	1.033	0.923
568912261	XP_006497469.1#Stam#20844# PREDICTED: signal transducing adapter molecule 1 isoform X1 [Mus musculus]	1.211	0.801	0.682	1.019	0.851	1.175	0.969	1.092	0.737	0.915	0.826
255522823	NP_775278.3#Tbc1d24#224617# TBC1 domain family member 24 isoform b [Mus musculus]	1.239	0.826	0.753	0.949	0.851	1.175	0.959	1.150	0.726	1.014	0.870
755539074	XP_011247425.1#Nt5c#50773# PREDICTED: 5'(3')-deoxyribonucleotidase, cytosolic type isoform X1 [Mus musculus]	0.872	1.007	0.717	0.985	0.851	1.175	0.685	0.902	0.672	0.898	0.785
568913241	XP_006497891.1#Surf4#20932# PREDICTED: surfeit locus protein 4 isoform X1 [Mus musculus]	0.968	0.933	0.798	0.904	0.851	1.175	0.734	0.874	0.644	0.860	0.752
226453485	YP_002791052.1#ND5#7668600# NADH dehydrogenase subunit 5 [Mus musculus castaneus]	1.016	0.908	0.745	0.957	0.851	1.175	0.683	0.884	0.614	0.758	0.686
13385942	NP_080720.1#Cs#12974# citrate synthase, mitochondrial precursor [Mus musculus]	0.994	0.930	0.749	0.953	0.851	1.175	0.807	0.922	0.676	0.921	0.799
11140825	NP_068561.1#Trappc4#60409# trafficking protein particle complex subunit 4 [Mus musculus]	0.844	0.917	0.742	0.961	0.851	1.174	0.671	0.766	0.581	0.736	0.659
568909147	XP_006529690.1#Dpp10#269109# PREDICTED: inactive dipeptidyl peptidase 10 isoform X2 [Mus musculus]	0.996	0.877	0.760	0.943	0.851	1.174	0.727	0.896	0.648	0.784	0.716
124486684	NP_001074524.1#Trappc10#216131# trafficking protein particle complex subunit 10 [Mus musculus]	1.073	0.828	0.700	1.004	0.852	1.174	0.886	0.942	0.644	0.869	0.756
568939692	XP_006505184.1#Tnpo3#320938# PREDICTED: transportin-3 isoform X2 [Mus musculus]	1.083	0.943	0.780	0.924	0.852	1.174	0.843	1.002	0.815	0.925	0.870
569019198	XP_006537191.1#Dlg4#13385# PREDICTED: disks large homolog 4 isoform X8 [Mus musculus]	1.009	0.812	0.754	0.950	0.852	1.174	0.755	0.950	0.601	0.795	0.698

18859587	NP_080076.1#Ppp1r2#66849# protein phosphatase inhibitor 2 [Mus musculus]	1.087	0.960	0.779	0.925	0.852	1.174	0.854	0.998	0.765	1.012	0.889
240120054	NP_079613.3#Akr7a5#110198# aflatoxin B1 aldehyde reductase member 2 [Mus musculus]	1.132	0.858	0.722	0.982	0.852	1.173	0.860	1.068	0.695	0.960	0.828
256773295	NP_001157715.1#Prpsap2#212627# phosphoribosyl pyrophosphate synthase-associated protein 2 isoform b [Mus musculus]	1.070	0.952	0.748	0.957	0.852	1.173	0.916	0.970	0.800	1.079	0.940
219282712	NP_001136437.1#Frrs1l#230235# DOMON domain-containing protein FRRS1L precursor [Mus musculus]	0.925	0.977	0.741	0.963	0.852	1.173	0.707	0.870	0.712	0.828	0.770
568992197	XP_006520909.1#Fmnl3#22379# PREDICTED: formin-like protein 3 isoform X4 [Mus musculus]	1.351	0.937	0.750	0.955	0.852	1.173	1.001	1.302	0.893	1.265	1.079
13385554	NP_080332.1#Cap2#67252# adenylyl cyclase-associated protein 2 [Mus musculus]	1.076	0.848	0.757	0.948	0.853	1.173	0.842	0.970	0.678	0.854	0.766
6678359	NP_033414.1#Tkt#21881# transketolase [Mus musculus]	1.114	0.948	0.765	0.940	0.853	1.173	0.834	1.022	0.776	1.033	0.905
112363072	NP_083987.1#Arpc2#76709# actin-related protein 2/3 complex subunit 2 [Mus musculus]	0.977	0.888	0.736	0.969	0.853	1.173	0.824	0.952	0.804	0.908	0.856
157951598	NP_033924.2#Capn2#12334# calpain-2 catalytic subunit [Mus musculus]	1.563	0.929	0.690	1.016	0.853	1.172	1.168	1.498	1.090	1.385	1.238
46049022	NP_079826.2#Psmd6#66413# 26S proteasome non-ATPase regulatory subunit 6 [Mus musculus]	0.963	0.811	0.784	0.922	0.853	1.172	0.752	0.893	0.610	0.725	0.668
254826716	NP_001156977.1#Atl3#109168# atlastin-3 isoform 1 [Mus musculus]	0.980	0.924	0.794	0.913	0.853	1.172	0.718	0.955	0.694	0.851	0.773
112181194	NP_035165.2#Pak1#18479# serine/threonine-protein kinase PAK 1 [Mus musculus]	0.992	0.999	0.760	0.947	0.853	1.172	0.783	0.909	0.752	0.937	0.844
13385536	NP_080310.1#Armc10#67211# armadillo repeat-containing protein 10 [Mus musculus]	1.309	0.811	0.777	0.929	0.853	1.172	0.984	1.250	0.769	1.042	0.905
755492134	XP_011236744.1#Inpp1#16329# PREDICTED: inositol polyphosphate 1-phosphatase isoform X1 [Mus musculus]	1.038	0.906	0.767	0.940	0.853	1.172	0.798	0.974	0.718	0.888	0.803
31543971	NP_062635.2#Ykt6#56418# synaptobrevin homolog YKT6 [Mus musculus]	1.090	0.929	0.746	0.961	0.854	1.172	0.838	1.023	0.736	0.991	0.864
568932065	XP_006539321.1#Mtfr1l#76824# PREDICTED: mitochondrial fission regulator 1-like isoform X1 [Mus musculus]	1.052	0.942	0.721	0.986	0.854	1.171	0.815	0.981	0.724	0.968	0.846
6681273	NP_031932.1#Eef1a2#13628# elongation factor 1-alpha 2 [Mus musculus]	1.061	0.923	0.726	0.982	0.854	1.171	0.820	0.971	0.706	0.876	0.791
755509987	XP_006539192.2#Tprgl#67808# PREDICTED: tumor protein p63-regulated gene 1-like protein isoform X1 [Mus musculus]	1.137	0.843	0.725	0.983	0.854	1.171	0.851	1.092	0.695	0.943	0.819
568963429	XP_006511987.1#Acaa1a#113868# PREDICTED: 3-ketoacyl-CoA thiolase A, peroxisomal isoform X2 [Mus musculus]	1.213	0.859	0.732	0.976	0.854	1.171	0.908	1.161	0.749	1.031	0.890
45433560	NP_032475.2#Kif5c#16574# kinesin heavy chain isoform 5C [Mus musculus]	1.092	0.919	0.761	0.947	0.854	1.171	0.848	1.056	0.758	0.966	0.862
6753320	NP_033966.1#Cct3#12462# T-complex protein 1 subunit gamma [Mus musculus]	1.068	0.897	0.737	0.971	0.854	1.171	0.840	0.978	0.751	0.918	0.835
568971697	XP_006532308.1#Guk1#14923# PREDICTED: guanylate kinase isoform X1 [Mus musculus]	0.956	0.946	0.753	0.955	0.854	1.171	0.767	0.865	0.627	0.916	0.771
568999766	XP_006524083.1#Fbxl16#214931# PREDICTED: F-box/LRR-repeat protein 16 isoform X1 [Mus musculus]	1.220	0.937	0.774	0.934	0.854	1.171	0.893	1.071	0.729	0.918	0.824
238637279	NP_032603.3#Slc3a2#17254# 4F2 cell-surface antigen heavy chain isoform b [Mus musculus]	1.088	0.922	0.760	0.948	0.854	1.171	0.887	1.040	0.761	1.031	0.896
568940123	XP_006505342.1#Aldh1l1#107747# PREDICTED: cytosolic 10-formyltetrahydrofolate dehydrogenase isoform X1 [Mus musculus]	1.181	0.845	0.760	0.950	0.855	1.170	0.943	1.144	0.752	0.980	0.866
116089273	NP_032138.3#Gdi2#14569# rab GDP dissociation inhibitor beta [Mus musculus]	0.999	0.919	0.730	0.979	0.855	1.170	0.712	0.890	0.634	0.909	0.771
8394027	NP_058587.1#Ppp2r1a#51792# serine/threonine-protein phosphatase 2A 65 kDa regulatory subunit A alpha isoform [Mus musculus]	1.230	0.888	0.753	0.956	0.855	1.170	0.928	1.124	0.829	1.046	0.937
167716841	YP_001686702.1#ATP8#5912284# ATP synthase F0 subunit 8 [Mus musculus musculus]	0.953	1.017	0.716	0.993	0.855	1.170	0.695	0.804	0.703	0.963	0.833
110626163	NP_598518.1#Pgs1#74451# CDP-diacylglycerol--glycerol-3-phosphate 3-phosphatidyltransferase, mitochondrial [Mus musculus]	0.935	1.034	0.776	0.934	0.855	1.170	0.720	0.878	0.685	0.968	0.826
18700024	NP_570954.1#Idh3b#170718# isocitrate dehydrogenase [NAD] subunit beta, mitochondrial [Mus musculus]	1.072	0.872	0.764	0.946	0.855	1.170	0.875	0.979	0.704	0.921	0.813
160707894	NP_033788.3#Akr1b3#11677# aldose reductase [Mus musculus]	1.040	0.943	0.761	0.949	0.855	1.170	0.777	0.986	0.694	0.958	0.826
45598372	NP_081671.1#Basp1#70350# brain acid soluble protein 1 [Mus musculus]	1.121	0.871	0.759	0.951	0.855	1.169	0.854	1.049	0.707	0.942	0.824
568964960	XP_006512583.1#Epb4.1l2#13822# PREDICTED: band 4.1-like protein 2 isoform X12 [Mus musculus]	0.870	1.052	0.776	0.934	0.855	1.169	0.662	0.856	0.708	0.894	0.801
755544316	XP_011242633.1#Acot13#66834# PREDICTED: acyl-coenzyme A thioesterase 13 isoform X1 [Mus musculus]	0.966	0.920	0.768	0.942	0.855	1.169	0.810	0.868	0.628	0.897	0.763
569005645	XP_006531742.1#Pcx#18563# PREDICTED: pyruvate carboxylase, mitochondrial isoform X1 [Mus musculus]	1.265	0.893	0.763	0.947	0.855	1.169	0.978	1.169	0.868	1.110	0.989
28077013	NP_082810.1#Smap1#98366# stromal membrane-associated protein 1 isoform 1 [Mus musculus]	1.059	0.919	0.735	0.976	0.855	1.169	0.820	1.017	0.766	0.940	0.853
344313177	NP_001230698.1#Cdc42#12540# cell division control protein 42 homolog isoform 2 [Mus musculus]	1.075	0.842	0.784	0.927	0.855	1.169	0.846	0.989	0.641	0.905	0.773
30519943	NP_848729.1#Samm50#68653# sorting and assembly machinery component 50 homolog [Mus musculus]	1.094	0.947	0.713	0.998	0.855	1.169	0.838	0.934	0.773	0.969	0.871
568966705	XP_006513298.1#Gnaz#14687# PREDICTED: guanine nucleotide-binding protein G(z) subunit alpha isoform X1 [Mus musculus]	1.165	0.890	0.746	0.965	0.856	1.169	0.946	1.173	0.877	1.009	0.943
6671569	NP_031501.1#Rplp0#11837# 60S acidic ribosomal protein P0 [Mus musculus]	1.038	0.843	0.739	0.972	0.856	1.169	0.812	0.953	0.628	0.895	0.762
46195430	NP_659119.2#Ndufs8#225887# NADH dehydrogenase [ubiquinone] iron-sulfur protein 8, mitochondrial [Mus musculus]	1.371	0.964	0.713	0.999	0.856	1.169	1.047	1.278	0.921	1.315	1.118
6753510	NP_034077.1#Cpne6#12891# copine-6 isoform a [Mus musculus]	1.057	0.859	0.761	0.950	0.856	1.169	0.793	0.988	0.662	0.876	0.769
755565394	XP_011245887.1#Arhgef9#236915# PREDICTED: rho guanine nucleotide exchange factor 9 isoform X5 [Mus musculus]	1.152	0.929	0.782	0.929	0.856	1.169	0.933	1.039	0.797	1.037	0.917
71061466	NP_001021383.1#Ensa#56205# alpha-endosulfine isoform b [Mus musculus]	1.078	0.912	0.768	0.944	0.856	1.168	0.829	1.016	0.705	0.979	0.842
569001421	XP_006524888.1#Ergic1#67458# PREDICTED: endoplasmic reticulum-Golgi intermediate compartment protein 1 isoform X2 [Mus musculus]	1.120	0.911	0.764	0.947	0.856	1.168	0.875	1.041	0.710	1.035	0.873
6753590	NP_034140.1#Cyp46a1#13116# cholesterol 24-hydroxylase precursor [Mus musculus]	1.230	1.015	0.789	0.923	0.856	1.168	0.939	1.166	0.830	1.306	1.068
46593021	NP_079683.2#Uqcrc1#22273# cytochrome b-c1 complex subunit 1, mitochondrial precursor [Mus musculus]	1.187	0.907	0.753	0.959	0.856	1.168	0.993	1.096	0.816	1.057	0.936
755506225	XP_011248279.1#Cdk5rap2#214444# PREDICTED: CDK5 regulatory subunit-associated protein 2 isoform X10 [Mus musculus]	1.073	0.895	0.733	0.979	0.856	1.168	0.835	0.996	0.773	0.954	0.864
568929156	XP_006503181.1#Ncdn#26562# PREDICTED: neurochondrin isoform X2 [Mus musculus]	1.026	0.884	0.778	0.934	0.856	1.168	0.792	0.925	0.663	0.870	0.766
568917895	XP_006500001.1#Rapgef4#56508# PREDICTED: rap guanine nucleotide exchange factor 4 isoform X2 [Mus musculus]	0.859	0.884	0.795	0.917	0.856	1.168	0.663	0.807	0.572	0.727	0.650
755496301	XP_011237210.1#Eprs#107508# PREDICTED: bifunctional glutamate/proline--tRNA ligase isoform X3 [Mus musculus]	1.161	0.874	0.780	0.933	0.856	1.168	0.842	1.038	0.795	0.935	0.865
27754103	NP_080235.2#Psmc6#67089# 26S protease regulatory subunit 10B [Mus musculus]	1.080	0.934	0.798	0.916	0.857	1.168	0.899	1.027	0.783	0.962	0.872
9506843	NP_062263.1#Lmna#16905# prelamin-A/C isoform C2 [Mus musculus]	1.184	0.881	0.772	0.941	0.857	1.167	0.983	1.045	0.810	0.975	0.893
568915799	XP_006498986.1#Pde1a#18573# PREDICTED: calcium/calmodulin-dependent 3',5'-cyclic nucleotide phosphodiesterase 1A isoform X3 [Mus musculus]	1.137	0.861	0.778	0.936	0.857	1.167	0.860	1.019	0.703	0.945	0.824
568974718	XP_006533748.1#Mpp2#50997# PREDICTED: MAGUK p55 subfamily member 2 isoform X1 [Mus musculus]	1.117	0.797	0.776	0.938	0.857	1.167	0.848	1.020	0.700	0.880	0.790
568934351	XP_006504081.1#Ube2k#53323# PREDICTED: ubiquitin-conjugating enzyme E2 K isoform X1 [Mus musculus]	1.049	0.918	0.763	0.951	0.857	1.167	0.911	0.886	0.683	0.966	0.825
755547716	XP_011243240.1#Cacna2d3#12294# PREDICTED: voltage-dependent calcium channel subunit alpha-2/delta-3 isoform X2 [Mus musculus]	1.004	0.913	0.766	0.948	0.857	1.167	0.780	0.941	0.704	0.867	0.786
6677779	NP_033107.1#Rpl28#19943# 60S ribosomal protein L28 [Mus musculus]	1.157	0.827	0.724	0.990	0.857	1.167	0.893	1.090	0.757	0.883	0.820
6680139	NP_032220.1#Gk#14933# glycerol kinase isoform 1 [Mus musculus]	1.149	0.899	0.748	0.965	0.857	1.167	0.940	1.030	0.804	1.044	0.924
134288917	NP_084514.2#Dync1h1#13424# cytoplasmic dynein 1 heavy chain 1 [Mus musculus]	1.173	0.903	0.752	0.962	0.857	1.167	0.908	1.104	0.769	1.037	0.903
568935657	XP_006535014.1#Mapk10#26414# PREDICTED: mitogen-activated protein kinase 10 isoform X6 [Mus musculus]	1.079	0.816	0.773	0.940	0.857	1.167	0.808	0.969	0.645	0.805	0.725
31542438	NP_079834.2#Cyb5b#66427# cytochrome b5 type B precursor [Mus musculus]	1.079	0.895	0.747	0.967	0.857	1.167	0.737	0.899	0.668	0.879	0.774
6753912	NP_034369.1#Fth1#14319# ferritin heavy chain [Mus musculus]	1.091	0.890	0.749	0.966	0.857	1.167	0.881	0.990	0.699	0.966	0.833
568986774	XP_006518644.1#Hnrnpc#15381# PREDICTED: heterogeneous nuclear ribonucleoproteins C1/C2 isoform X3 [Mus musculus]	1.260	0.757	0.774	0.940	0.857	1.167	0.967	1.159	0.758	0.901	0.829
13385878	NP_080662.1#Snx2#67804# sorting nexin-2 [Mus musculus]	0.970	0.917	0.788	0.926	0.857	1.166	0.787	0.876	0.659	0.867	0.763
260166715	NP_001159459.1#Ubap2l#74383# ubiquitin-associated protein 2-like isoform 6 [Mus musculus]	1.012	0.944	0.769	0.946	0.857	1.166	0.840	1.018	0.722	1.001	0.861
9789995	NP_062615.1#Stmn1#16765# stathmin [Mus musculus]	1.069	0.878	0.734	0.981	0.858	1.166	0.808	0.971	0.719	0.888	0.803
111494223	NP_780540.2#Nt5dc3#103466# 5'-nucleotidase domain-containing protein 3 [Mus musculus]	0.817	1.110	0.797	0.918	0.858	1.166	0.634	0.771	0.646	0.915	0.780
6754910	NP_035078.1#Nudc#18221# nuclear migration protein nudC [Mus musculus]	1.053	0.900	0.793	0.922	0.858	1.166	0.870	1.003	0.702	0.832	0.767
22165384	NP_666228.1#Tubb4b#227613# tubulin beta-4B chain [Mus musculus]	1.028	0.933	0.742	0.973	0.858	1.166	0.811	1.002	0.768	1.072	0.920
31543940	NP_062780.2#Vapb#56491# vesicle-associated membrane protein-associated protein B [Mus musculus]	1.019	0.993	0.812	0.904	0.858	1.166	0.845	0.972	0.771	1.032	0.902
7305031	NP_038841.1#Epb4.1l3#13823# band 4.1-like protein 3 [Mus musculus]	1.117	0.972	0.760	0.956	0.858	1.166	0.868	1.008	0.787	1.056	0.922
569008633	XP_006527629.1#Ddx3x#13205# PREDICTED: ATP-dependent RNA helicase DDX3X isoform X1 [Mus musculus]	1.080	0.856	0.724	0.992	0.858	1.166	0.863	1.067	0.700	0.894	0.797
34996495	NP_062616.2#Rpn2#20014# dolichyl-diphosphooligosaccharide--protein glycosyltransferase subunit 2 precursor [Mus musculus]	0.974	0.933	0.777	0.939	0.858	1.166	0.715	0.816	0.654	0.808	0.731
115385966	NP_001041732.1#Tnpo1#238799# transportin-1 isoform 2 [Mus musculus]	1.270	0.941	0.743	0.973	0.858	1.165	0.981	1.198	0.880	1.170	1.025
568988956	XP_006519696.1#Pck2#74551# PREDICTED: phosphoenolpyruvate carboxykinase [GTP], mitochondrial isoform X3 [Mus musculus]	1.113	0.969	0.750	0.966	0.858	1.165	0.851	1.059	0.805	1.044	0.925
755493915	XP_011246242.1#Cdk18#18557# PREDICTED: cyclin-dependent kinase 18 isoform X1 [Mus musculus]	1.056	0.870	0.739	0.977	0.858	1.165	0.821	0.992	0.671	0.905	0.788
31543900	NP_067299.2#Ttyh1#57776# protein tweety homolog 1 isoform 2 [Mus musculus]	1.036	0.838	0.749	0.967	0.858	1.165	0.805	0.972	0.681	0.809	0.745
568931796	XP_006539191.1#Sdhb#67680# PREDICTED: succinate dehydrogenase [ubiquinone] iron-sulfur subunit, mitochondrial isoform X1 [Mus musculus]	1.141	0.944	0.780	0.937	0.858	1.165	0.909	1.096	0.838	1.058	0.948
7710086	NP_057885.1#Rab10#19325# ras-related protein Rab-10 [Mus musculus]	0.999	0.866	0.746	0.971	0.859	1.165	0.760	0.938	0.630	0.946	0.788
27370360	NP_766478.1#Slc6a11#243616# sodium- and chloride-dependent GABA transporter 3 [Mus musculus]	0.959	1.033	0.719	0.999	0.859	1.165	0.852	0.833	0.735	0.959	0.847
569003981	XP_006526055.1#Bin1#30948# PREDICTED: myc box-dependent-interacting protein 1 isoform X15 [Mus musculus]	1.095	0.933	0.751	0.966	0.859	1.165	0.846	1.018	0.754	1.026	0.890
31542873	NP_080969.2#Gabarapl2#93739# gamma-aminobutyric acid receptor-associated protein-like 2 [Mus musculus]	0.978	0.953	0.779	0.938	0.859	1.164	0.790	0.890	0.692	0.909	0.801

6679753	NP_032016.1#Fau#14109# 40S ribosomal protein S30 precursor [Mus musculus]	0.953	1.005	0.761	0.957	0.859	1.164	0.791	0.846	0.690	0.956	0.823
164698474	NP_035500.2#Cyfip1#20430# cytoplasmic FMR1-interacting protein 1 isoform a [Mus musculus]	1.208	0.930	0.781	0.937	0.859	1.164	0.895	1.180	0.876	1.053	0.965
568956344	XP_006530838.1#Rbmxl1#19656# PREDICTED: RNA binding motif protein, X-linked-like-1 isoform X1 [Mus musculus]	0.790	0.862	0.741	0.977	0.859	1.164	0.636	0.803	0.517	0.700	0.609
74315975	NP_081633.1#Psmd1#70247# 26S proteasome non-ATPase regulatory subunit 1 [Mus musculus]	1.108	0.869	0.709	1.009	0.859	1.164	0.905	1.047	0.695	0.976	0.836
85362729	NP_001034228.1#Camk2g#12325# calcium/calmodulin-dependent protein kinase type II subunit gamma isoform 3 [Mus musculus]	1.156	0.757	0.744	0.974	0.859	1.164	0.930	1.211	0.689	0.874	0.782
112293264	NP_031978.2#Pdia3#14827# protein disulfide-isomerase A3 precursor [Mus musculus]	1.152	0.924	0.749	0.970	0.859	1.164	0.884	1.030	0.775	1.043	0.909
552953687	NP_001272802.1#Trim3#55992# tripartite motif-containing protein 3 isoform b [Mus musculus]	1.006	0.868	0.742	0.977	0.859	1.164	0.845	1.108	0.716	0.924	0.820
569001103	XP_006524732.1#Mtch1#56462# PREDICTED: mitochondrial carrier homolog 1 isoform X2 [Mus musculus]	1.091	0.988	0.743	0.976	0.860	1.163	0.823	1.055	0.815	1.039	0.927
7305395	NP_038660.1#Pnp#18950# purine nucleoside phosphorylase [Mus musculus]	1.048	0.916	0.786	0.934	0.860	1.163	0.764	0.920	0.680	0.848	0.764
31981577	NP_035200.2#Pfdn2#18637# prefoldin subunit 2 [Mus musculus]	0.970	0.945	0.778	0.942	0.860	1.163	0.762	0.906	0.620	0.956	0.788
54607098	NP_075770.1#Sdha#66945# succinate dehydrogenase [ubiquinone] flavoprotein subunit, mitochondrial precursor [Mus musculus]	1.225	0.936	0.744	0.976	0.860	1.163	0.935	1.153	0.825	1.085	0.955
569002922	XP_006525541.1#Wdr7#104082# PREDICTED: WD repeat-containing protein 7 isoform X2 [Mus musculus]	1.128	0.841	0.750	0.970	0.860	1.163	0.914	1.102	0.717	0.959	0.838
71061455	NP_001021118.1#Dnm1l#74006# dynamin-1-like protein isoform b [Mus musculus]	1.066	0.863	0.764	0.956	0.860	1.163	0.794	0.950	0.676	0.902	0.789
568915130	XP_006498668.1#Atp9a#11981# PREDICTED: probable phospholipid-transporting ATPase IIA isoform X4 [Mus musculus]	1.151	0.764	0.729	0.991	0.860	1.162	0.948	1.032	0.655	0.858	0.757
30519971	NP_848743.1#Atl1#73991# atlastin-1 [Mus musculus]	1.078	0.931	0.767	0.953	0.860	1.162	0.802	0.982	0.723	0.999	0.861
19526936	NP_598498.1#Lancl2#71835# lanC-like protein 2 [Mus musculus]	1.325	0.779	0.811	0.910	0.860	1.162	0.945	1.273	0.733	0.980	0.856
157951596	NP_033931.4#Car2#12349# carbonic anhydrase 2 [Mus musculus]	1.010	1.029	0.775	0.946	0.860	1.162	0.823	0.943	0.807	1.070	0.939
755492825	XP_011236912.1#Rftn2#74013# PREDICTED: raftlin-2 isoform X2 [Mus musculus]	1.151	1.013	0.719	1.002	0.860	1.162	0.851	1.129	0.900	1.107	1.003
21450129	NP_659033.1#Acat1#110446# acetyl-CoA acetyltransferase, mitochondrial precursor [Mus musculus]	1.176	0.916	0.738	0.984	0.861	1.162	0.963	1.144	0.815	1.059	0.937
7305523	NP_038863.1#Trove2#20822# 60 kDa SS-A/Ro ribonucleoprotein [Mus musculus]	1.003	0.917	0.776	0.946	0.861	1.162	0.775	0.952	0.674	0.911	0.792
6680724	NP_031507.1#Arf6#11845# ADP-ribosylation factor 6 [Mus musculus]	0.989	0.850	0.785	0.937	0.861	1.162	0.797	0.966	0.632	0.829	0.730
255759902	NP_776101.3#Maoa#17161# amine oxidase [flavin-containing] A [Mus musculus]	1.000	0.944	0.729	0.994	0.861	1.161	0.817	0.959	0.711	0.958	0.835
84000009	NP_036144.2#Gprin1#26913# G protein-regulated inducer of neurite outgrowth 1 [Mus musculus]	1.149	0.865	0.775	0.948	0.861	1.161	0.892	1.123	0.726	0.993	0.860
568965329	XP_006512763.1#Lace1#215951# PREDICTED: lactation elevated protein 1 isoform X2 [Mus musculus]	1.018	0.868	0.745	0.978	0.862	1.161	0.806	0.948	0.731	0.792	0.761
156713423	NP_080821.3#Psmd8#57296# 26S proteasome non-ATPase regulatory subunit 8 [Mus musculus]	1.065	0.880	0.809	0.914	0.862	1.160	0.889	0.947	0.736	0.880	0.808
110625979	NP_080283.3#Eef1g#67160# elongation factor 1-gamma [Mus musculus]	1.006	0.893	0.759	0.965	0.862	1.160	0.783	0.998	0.715	0.995	0.855
755499882	XP_011237899.1#Ndrg3#29812# PREDICTED: protein NDRG3 isoform X4 [Mus musculus]	1.097	0.878	0.716	1.008	0.862	1.160	0.837	1.054	0.702	0.959	0.831
569010326	XP_006528189.1#Dlg3#53310# PREDICTED: disks large homolog 3 isoform X4 [Mus musculus]	1.022	0.881	0.708	1.016	0.862	1.160	0.712	1.050	0.625	0.928	0.776
21704066	NP_663516.1#Rap1a#109905# ras-related protein Rap-1A precursor [Mus musculus]	1.160	1.052	0.866	0.859	0.863	1.159	0.903	1.098	0.876	1.230	1.053
6755901	NP_035783.1#Tuba1a#22142# tubulin alpha-1A chain [Mus musculus]	1.107	0.918	0.766	0.959	0.863	1.159	0.862	1.021	0.719	0.969	0.844
30089688	NP_835738.1#Arrb1#109689# beta-arrestin-1 isoform B [Mus musculus]	0.865	1.003	0.770	0.955	0.863	1.159	0.686	0.807	0.663	0.833	0.748
165932333	NP_001107032.1#Glo1#109801# lactoylglutathione lyase [Mus musculus]	0.919	1.121	0.770	0.955	0.863	1.159	0.704	0.850	0.768	1.034	0.901
568933716	XP_006503773.1#Ctbp1#13016# PREDICTED: C-terminal-binding protein 1 isoform X1 [Mus musculus]	0.997	0.764	0.765	0.961	0.863	1.159	0.799	0.951	0.558	0.778	0.668
568938650	XP_006504732.1#Adap1#231821# PREDICTED: arf-GAP with dual PH domain-containing protein 1 isoform X1 [Mus musculus]	1.094	0.829	0.815	0.911	0.863	1.159	0.838	1.086	0.696	0.912	0.804
568964425	XP_006512466.1#Lztfl1#93730# PREDICTED: leucine zipper transcription factor-like protein 1 isoform X2 [Mus musculus]	1.076	0.875	0.725	1.001	0.863	1.159	0.854	1.003	0.711	0.914	0.813
755495420	XP_011237050.1#Copa#12847# PREDICTED: coatomer subunit alpha isoform X1 [Mus musculus]	1.523	0.831	0.789	0.936	0.863	1.159	1.190	1.439	0.976	1.209	1.092
6678055	NP_033252.1#Snrpd1#20641# small nuclear ribonucleoprotein Sm D1 [Mus musculus]	0.934	0.844	0.818	0.908	0.863	1.159	0.604	1.007	0.541	0.810	0.675
254675270	NP_033121.2#Rps5#20103# 40S ribosomal protein S5 [Mus musculus]	1.272	0.861	0.791	0.935	0.863	1.159	0.939	1.243	0.818	1.068	0.943
189409138	NP_082270.1#Cand1#71902# cullin-associated NEDD8-dissociated protein 1 [Mus musculus]	1.148	0.887	0.779	0.947	0.863	1.159	0.944	1.087	0.757	1.020	0.888
117320524	NP_001070963.1#Pik3r1#18708# phosphatidylinositol 3-kinase regulatory subunit alpha isoform 2 [Mus musculus]	1.000	0.910	0.760	0.966	0.863	1.159	0.798	0.927	0.678	0.892	0.785
164663791	NP_035231.2#Prkca#18750# protein kinase C alpha type [Mus musculus]	0.967	0.876	0.726	1.001	0.863	1.158	0.781	0.889	0.625	0.838	0.731
568990429	XP_006520117.1#Trio#223435# PREDICTED: triple functional domain protein isoform X6 [Mus musculus]	1.194	0.852	0.790	0.937	0.863	1.158	0.931	1.132	0.733	1.024	0.878
93102417	NP_851009.2#Gars#353172# glycine--tRNA ligase [Mus musculus]	0.944	1.080	0.780	0.947	0.864	1.158	0.770	0.860	0.812	0.948	0.880
33859811	NP_849209.1#Hadha#97212# trifunctional enzyme subunit alpha, mitochondrial precursor [Mus musculus]	1.168	0.944	0.766	0.962	0.864	1.158	0.885	1.067	0.824	1.098	0.961
31980632	NP_058033.2#Lxn#17035# latexin [Mus musculus]	1.071	0.934	0.763	0.964	0.864	1.158	0.804	1.046	0.751	0.976	0.864
56790921	NP_780398.2#Hepacam#72927# hepatocyte cell adhesion molecule precursor [Mus musculus]	1.069	1.012	0.736	0.992	0.864	1.158	0.776	1.057	0.880	1.216	1.048
9790285	NP_062754.1#Vps29#56433# vacuolar protein sorting-associated protein 29 [Mus musculus]	0.863	0.980	0.778	0.950	0.864	1.158	0.753	0.836	0.617	0.930	0.773
6680047	NP_032169.1#Gnb2l1#14694# guanine nucleotide-binding protein subunit beta-2-like 1 [Mus musculus]	1.120	0.862	0.772	0.956	0.864	1.158	0.874	1.072	0.702	0.972	0.837
568928284	XP_006502760.1#Cap1#12331# PREDICTED: adenylyl cyclase-associated protein 1 isoform X1 [Mus musculus]	1.146	0.853	0.767	0.961	0.864	1.157	0.908	0.987	0.751	0.943	0.847
568970049	XP_006514664.1#Sptbn1#20742# PREDICTED: spectrin beta chain, non-erythrocytic 1 isoform X2 [Mus musculus]	1.166	0.936	0.761	0.968	0.864	1.157	0.914	1.097	0.793	1.063	0.928
411147445	NP_001258657.1#Trim2#80890# tripartite motif-containing protein 2 isoform 2 [Mus musculus]	1.099	0.868	0.702	1.027	0.864	1.157	0.866	1.029	0.685	0.990	0.838
755538368	XP_011247262.1#Ube2o#217342# PREDICTED: E2/E3 hybrid ubiquitin-protein ligase UBE2O isoform X2 [Mus musculus]	1.175	0.866	0.783	0.946	0.864	1.157	0.916	1.115	0.797	0.963	0.880
569020715	XP_006544954.1#Aip#11632# PREDICTED: AH receptor-interacting protein isoform X1 [Mus musculus]	1.162	0.879	0.743	0.986	0.864	1.157	0.903	1.107	0.735	1.032	0.884
357394768	NP_001239422.1#Osbpl1a#64291# oxysterol-binding protein-related protein 1 isoform 3 [Mus musculus]	1.074	0.844	0.728	1.001	0.865	1.156	0.912	1.075	0.726	0.951	0.838
568975422	XP_006534088.1#Snap47#67826# PREDICTED: synaptosomal-associated protein 47 isoform X2 [Mus musculus]	1.031	0.874	0.782	0.947	0.865	1.156	0.814	0.942	0.661	0.869	0.765
219275596	NP_081626.2#Nars#70223# asparagine--tRNA ligase, cytoplasmic isoform 2 [Mus musculus]	1.146	0.981	0.827	0.902	0.865	1.156	0.980	1.004	0.916	1.032	0.974
6677839	NP_033141.1#S100b#20203# protein S100-B [Mus musculus]	0.952	0.965	0.776	0.954	0.865	1.156	0.714	0.887	0.687	0.898	0.792
27370032	NP_766300.1#Uba6#231380# ubiquitin-like modifier-activating enzyme 6 [Mus musculus]	0.943	0.940	0.721	1.009	0.865	1.156	0.759	0.871	0.692	0.841	0.767
6753966	NP_034401.1#Gpd1#14555# glycerol-3-phosphate dehydrogenase [NAD(+)], cytoplasmic [Mus musculus]	1.045	0.844	0.782	0.949	0.865	1.156	0.847	1.019	0.681	0.843	0.762
30061351	NP_835582.1#Hist1h4j#319159# histone H4 [Mus musculus]	0.682	0.964	0.758	0.972	0.865	1.156	0.531	0.620	0.480	0.659	0.569
13385374	NP_080163.1#Rab5a#271457# ras-related protein Rab-5A [Mus musculus]	1.177	0.935	0.809	0.922	0.865	1.155	0.892	1.022	0.721	0.817	0.769
651164671	NP_001280550.1#Ganab#14376# neutral alpha-glucosidase AB isoform 2 precursor [Mus musculus]	0.999	0.886	0.793	0.938	0.866	1.155	0.778	0.908	0.712	0.812	0.762
568971868	XP_006532391.1#Kif3a#16568# PREDICTED: kinesin-like protein KIF3A isoform X3 [Mus musculus]	1.139	0.998	0.691	1.040	0.866	1.155	0.897	1.074	0.918	1.049	0.983
755538682	XP_011247334.1#Gga3#260302# PREDICTED: ADP-ribosylation factor-binding protein GGA3 isoform X4 [Mus musculus]	1.169	0.888	0.744	0.988	0.866	1.155	0.901	1.123	0.764	1.032	0.898
10946678	NP_067329.1#Drg2#13495# developmentally-regulated GTP-binding protein 2 [Mus musculus]	0.981	0.954	0.802	0.929	0.866	1.155	0.786	0.914	0.716	0.905	0.811
161086893	NP_598542.3#Cab39#12283# calcium-binding protein 39 [Mus musculus]	1.106	0.771	0.726	1.005	0.866	1.155	0.967	1.076	0.678	0.960	0.819
33859650	NP_035973.1#Esyt1#23943# extended synaptotagmin-1 [Mus musculus]	0.990	0.899	0.771	0.961	0.866	1.155	0.785	0.930	0.634	0.906	0.770
25141330	NP_733467.1#Cpne1#266692# copine-1 [Mus musculus]	1.111	0.883	0.803	0.928	0.866	1.155	0.887	1.037	0.763	0.935	0.849
569006023	XP_006531920.1#Scyl1#78891# PREDICTED: N-terminal kinase-like protein isoform X2 [Mus musculus]	1.120	0.958	0.760	0.972	0.866	1.155	0.802	1.138	0.804	1.054	0.929
568987095	XP_006518798.1#Rps24#20088# PREDICTED: 40S ribosomal protein S24 isoform X5 [Mus musculus]	0.801	0.834	0.752	0.980	0.866	1.155	0.634	0.754	0.551	0.606	0.579
427918099	NP_001258790.1#Add2#11519# beta-adducin isoform 2 [Mus musculus]	1.125	0.853	0.771	0.962	0.866	1.154	0.839	1.031	0.706	0.897	0.802
7304855	NP_038484.1#Actn3#11474# alpha-actinin-3 [Mus musculus]	0.971	0.865	0.745	0.988	0.866	1.154	0.745	0.937	0.595	0.860	0.728
576796187	NP_001276700.1#Exoc1#69940# exocyst complex component 1 isoform 3 [Mus musculus]	0.940	0.846	0.761	0.972	0.866	1.154	0.720	0.908	0.606	0.771	0.689
21728376	NP_075891.1#Myl12b#67938# myosin regulatory light chain 12B [Mus musculus]	1.051	0.929	0.742	0.991	0.866	1.154	0.837	1.025	0.697	0.981	0.839
282398108	NP_598694.3#Rpn1#103963# dolichyl-diphosphooligosaccharide--protein glycosyltransferase subunit 1 precursor [Mus musculus]	1.139	0.836	0.703	1.030	0.866	1.154	0.965	1.008	0.726	0.923	0.825
124517709	NP_032104.2#Gad2#14417# glutamate decarboxylase 2 [Mus musculus]	1.214	1.130	0.754	0.979	0.866	1.154	0.927	1.178	0.978	1.399	1.189
569003989	XP_006526059.1#Rpl17#319195# PREDICTED: 60S ribosomal protein L17 isoform X1 [Mus musculus]	0.988	0.912	0.790	0.944	0.867	1.154	0.777	0.937	0.678	0.894	0.786
83699424	NP_033103.2#Rpl18#19899# 60S ribosomal protein L18 [Mus musculus]	0.891	0.886	0.756	0.977	0.867	1.154	0.672	0.816	0.456	0.702	0.579
33413404	NP_877957.1#Gpt#76282# alanine aminotransferase 1 [Mus musculus]	1.043	0.849	0.707	1.026	0.867	1.154	0.815	0.994	0.647	0.890	0.768
124339829	NP_034609.2#Hspa1a#193740# heat shock 70 kDa protein 1A [Mus musculus]	0.957	0.874	0.740	0.994	0.867	1.154	0.727	0.883	0.651	0.886	0.769
557636720	NP_001273530.1#Clasp2#76499# CLIP-associating protein 2 isoform f [Mus musculus]	1.312	0.967	0.766	0.968	0.867	1.154	1.057	1.219	0.969	1.231	1.100
124487313	NP_001074550.1#Gls#14660# glutaminase kidney isoform, mitochondrial isoform 1 [Mus musculus]	1.165	0.850	0.755	0.979	0.867	1.153	0.950	1.123	0.745	0.964	0.855
6681069	NP_031817.1#Csrp1#13007# cysteine and glycine-rich protein 1 [Mus musculus]	1.269	0.968	0.706	1.028	0.867	1.153	0.980	1.178	0.881	1.208	1.045

27734986	NP_663517.2#Ahcyl1#229709# putative adenosylhomocysteinase 2 [Mus musculus]	1.159	0.900	0.771	0.963	0.867	1.153	0.894	1.090	0.713	0.975	0.844
117606385	NP_938037.2#Tmx3#67988# protein disulfide-isomerase TMX3 precursor [Mus musculus]	0.980	0.899	0.733	1.001	0.867	1.153	0.809	0.890	0.622	0.906	0.764
568948570	XP_006541286.1#Mcee#73724# PREDICTED: methylmalonyl-CoA epimerase, mitochondrial isoform X1 [Mus musculus]	1.077	0.957	0.712	1.022	0.867	1.153	0.866	1.001	0.767	1.022	0.894
47894398	NP_001001491.1#Tpm4#326618# tropomyosin alpha-4 chain [Mus musculus]	1.107	0.858	0.738	0.997	0.867	1.153	0.903	1.018	0.665	0.984	0.824
755555583	XP_011244521.1#Mpc1#55951# PREDICTED: mitochondrial pyruvate carrier 1 isoform X1 [Mus musculus]	0.822	0.926	0.809	0.926	0.867	1.153	0.767	0.704	0.646	0.729	0.688
83649709	NP_058539.2#Atxn10#54138# ataxin-10 [Mus musculus]	1.051	0.848	0.764	0.970	0.867	1.153	0.859	1.041	0.676	0.955	0.815
6754218	NP_034575.1#Hmx1#15371# homeobox protein HMX1 [Mus musculus]	1.062	0.847	0.724	1.010	0.867	1.153	0.867	0.975	0.593	0.967	0.780
23346461	NP_694704.1#Ndufs2#226646# NADH dehydrogenase [ubiquinone] iron-sulfur protein 2, mitochondrial precursor [Mus musculus]	1.113	0.977	0.772	0.963	0.868	1.153	0.854	1.084	0.810	1.032	0.921
755500512	XP_011238071.1#Lnp#69605# PREDICTED: protein lunapark isoform X2 [Mus musculus]	1.027	0.915	0.753	0.983	0.868	1.152	0.871	0.912	0.684	0.948	0.816
568967383	XP_006513626.1#Agap2#216439# PREDICTED: arf-GAP with GTPase, ANK repeat and PH domain-containing protein 2 isoform X2 [Mus musculus]	1.020	0.890	0.758	0.977	0.868	1.152	0.780	0.989	0.689	0.886	0.788
13430890	NP_056602.1#Hist1h1e#50709# histone H1.4 [Mus musculus]	1.066	1.105	0.762	0.974	0.868	1.152	0.810	0.994	0.809	1.142	0.975
568936184	XP_006535270.1#Sec31a#69162# PREDICTED: protein transport protein Sec31A isoform X15 [Mus musculus]	1.105	0.881	0.791	0.945	0.868	1.152	0.958	1.080	0.738	0.964	0.851
164565394	NP_766292.2#Dnajc11#230935# dnaJ homolog subfamily C member 11 [Mus musculus]	1.140	0.910	0.807	0.929	0.868	1.152	0.865	1.112	0.824	0.987	0.905
568916474	XP_006499314.1#Arhgap1#228359# PREDICTED: rho GTPase-activating protein 1 isoform X2 [Mus musculus]	1.115	0.900	0.731	1.006	0.868	1.152	0.834	1.104	0.792	0.953	0.873
568929440	XP_006503316.1#Akr1a1#58810# PREDICTED: alcohol dehydrogenase [NADP(+)] isoform X1 [Mus musculus]	1.044	0.939	0.782	0.955	0.868	1.151	0.811	0.984	0.744	0.925	0.834
10946940	NP_067493.1#Rab2a#59021# ras-related protein Rab-2A [Mus musculus]	0.879	0.890	0.768	0.969	0.869	1.151	0.714	0.879	0.664	0.829	0.747
356991208	NP_001239332.1#Gria1#14799# glutamate receptor 1 isoform 3 [Mus musculus]	1.095	0.881	0.820	0.917	0.869	1.151	0.853	1.049	0.644	1.031	0.838
23956176	NP_080750.1#Sugt1#67955# suppressor of G2 allele of SKP1 homolog [Mus musculus]	0.953	0.920	0.813	0.925	0.869	1.151	0.763	0.891	0.595	0.928	0.762
13195604	NP_077137.1#Rps23#66475# 40S ribosomal protein S23 [Mus musculus]	0.940	0.893	0.764	0.973	0.869	1.151	0.756	0.886	0.653	0.852	0.753
225007593	NP_034573.2#Hmox2#15369# heme oxygenase 2 [Mus musculus]	1.146	0.940	0.727	1.011	0.869	1.151	0.930	1.086	0.834	1.045	0.939
116268115	NP_057918.2#Auh#11992# methylglutaconyl-CoA hydratase, mitochondrial precursor [Mus musculus]	1.184	0.925	0.757	0.981	0.869	1.151	0.959	1.152	0.816	1.091	0.953
21450201	NP_659071.1#Micu1#216001# calcium uptake protein 1, mitochondrial isoform 3 [Mus musculus]	1.068	0.916	0.825	0.912	0.869	1.151	0.819	1.036	0.723	0.976	0.849
755531683	XP_011241239.1#Map4#17758# PREDICTED: microtubule-associated protein 4 isoform X4 [Mus musculus]	1.139	1.003	0.778	0.960	0.869	1.151	0.899	1.080	0.851	1.134	0.992
158937240	NP_038927.2#Timm10#30059# mitochondrial import inner membrane translocase subunit Tim10 [Mus musculus]	1.088	0.854	0.782	0.956	0.869	1.151	0.907	0.984	0.667	0.948	0.808
6680502	NP_032436.1#Itm2b#16432# integral membrane protein 2B [Mus musculus]	1.196	0.959	0.752	0.986	0.869	1.151	0.981	1.156	0.855	1.195	1.025
568978506	XP_006515387.1#Rtn1#104001# PREDICTED: reticulon-1 isoform X1 [Mus musculus]	1.068	0.866	0.762	0.976	0.869	1.151	0.836	0.960	0.674	0.908	0.791
241982742	NP_001155296.1#Mtx1#17827# metaxin-1 isoform 2 [Mus musculus]	1.370	0.856	0.753	0.985	0.869	1.151	1.020	1.361	0.849	1.189	1.019
239985479	NP_001155268.1#Gucy1b3#54195# guanylate cyclase soluble subunit beta-1 isoform 2 [Mus musculus]	1.137	0.908	0.783	0.956	0.869	1.151	0.890	1.032	0.832	1.033	0.932
755553088	XP_011244110.1#Arvcf#11877# PREDICTED: armadillo repeat protein deleted in velo-cardio-facial syndrome homolog isoform X2 [Mus musculus]	1.175	0.893	0.774	0.964	0.869	1.151	0.927	1.113	0.764	1.060	0.912
33859753	NP_077777.1#Rap1b#215449# ras-related protein Rap-1b precursor [Mus musculus]	1.101	0.956	0.755	0.984	0.869	1.150	0.883	1.031	0.782	1.047	0.915
33859488	NP_033476.1#Tubb2a#22151# tubulin beta-2A chain [Mus musculus]	1.003	0.914	0.774	0.964	0.869	1.150	0.745	0.929	0.658	0.867	0.763
52345394	NP_001004365.1#Actr3b#242894# actin-related protein 3B [Mus musculus]	1.055	0.847	0.775	0.964	0.869	1.150	0.868	0.966	0.684	0.870	0.777
41281679	NP_619733.1#Gnb5#14697# guanine nucleotide-binding protein subunit beta-5 isoform 2 [Mus musculus]	1.059	0.836	0.795	0.944	0.869	1.150	0.923	1.051	0.707	0.889	0.798
568950024	XP_006507600.1#Stim1#20866# PREDICTED: stromal interaction molecule 1 isoform X5 [Mus musculus]	1.039	1.001	0.746	0.993	0.870	1.150	0.743	1.063	0.720	1.089	0.904
550822410	NP_001272433.1#Nde1#67203# nuclear distribution protein nudE homolog 1 isoform d [Mus musculus]	1.087	0.905	0.745	0.995	0.870	1.150	0.806	1.021	0.694	0.958	0.826
568889025	XP_006543745.1#LOC102642884#102642884# PREDICTED: cytochrome c oxidase subunit 7C, mitochondrial [Mus musculus]	1.186	1.036	0.795	0.945	0.870	1.150	0.887	1.187	0.970	1.111	1.041
21313458	NP_084428.1#Nol3#78688# nucleolar protein 3 [Mus musculus]	1.718	0.814	0.767	0.973	0.870	1.150	1.340	1.649	1.087	1.346	1.216
595763404	NP_001277728.1#Inpp4a#269180# type I inositol 3,4-bisphosphate 4-phosphatase isoform 5 [Mus musculus]	1.028	0.858	0.726	1.014	0.870	1.150	0.817	0.973	0.670	0.864	0.767
31982520	NP_031407.2#Acadl#11363# long-chain specific acyl-CoA dehydrogenase, mitochondrial precursor [Mus musculus]	1.050	1.079	0.798	0.942	0.870	1.149	0.837	0.990	0.865	1.108	0.986
568965538	XP_006512864.1#Stx7#53331# PREDICTED: syntaxin-7 isoform X1 [Mus musculus]	0.973	0.955	0.692	1.048	0.870	1.149	0.760	0.933	0.716	0.900	0.808
358030322	NP_001239595.1#Dmtn#13829# dematin isoform 4 [Mus musculus]	0.862	0.882	0.804	0.937	0.870	1.149	0.685	0.816	0.575	0.748	0.661
568988404	XP_006519428.1#Sdr39u1#654795# PREDICTED: epimerase family protein SDR39U1 isoform X1 [Mus musculus]	0.967	0.889	0.722	1.018	0.870	1.149	0.656	1.026	0.616	0.881	0.748
122	K22E_HUMAN	0.766	1.149	0.779	0.962	0.870	1.149	0.650	0.645	0.639	0.823	0.731
569004246	XP_006526183.1#Ube2d2a#56550# PREDICTED: ubiquitin-conjugating enzyme E2 D2 isoform X1 [Mus musculus]	1.176	0.899	0.759	0.982	0.870	1.149	0.883	1.164	0.804	1.034	0.919
13386452	NP_084358.1#Hist3h2ba#78303# histone H2B type 3-A [Mus musculus]	1.095	1.039	0.750	0.991	0.871	1.149	0.794	1.112	0.880	1.100	0.990
568904952	XP_006544850.1#LOC102641949#102641949# PREDICTED: ES1 protein homolog, mitochondrial [Mus musculus]	1.155	0.910	0.783	0.958	0.871	1.149	0.921	1.072	0.828	1.079	0.953
24418919	NP_722476.1#Pygb#110078# glycogen phosphorylase, brain form [Mus musculus]	1.124	0.935	0.775	0.967	0.871	1.149	0.891	1.069	0.798	1.038	0.918
569003472	XP_006525808.1#Mapre2#212307# PREDICTED: microtubule-associated protein RP/EB family member 2 isoform X2 [Mus musculus]	1.060	0.872	0.744	0.997	0.871	1.148	0.904	1.020	0.686	0.978	0.832
313151222	NP_001186225.1#Acly#104112# ATP-citrate synthase isoform 1 [Mus musculus]	1.129	0.969	0.758	0.984	0.871	1.148	0.889	1.056	0.804	1.094	0.949
326381098	NP_001191908.1#Sars#20226# serine--tRNA ligase, cytoplasmic isoform 2 [Mus musculus]	1.020	0.909	0.738	1.004	0.871	1.148	0.845	0.962	0.684	0.954	0.819
30794450	NP_077174.1#Rpl4#67891# 60S ribosomal protein L4 [Mus musculus]	1.018	0.907	0.792	0.950	0.871	1.148	0.831	0.920	0.695	0.870	0.782
134031994	NP_032970.2#Psma2#19166# proteasome subunit alpha type-2 [Mus musculus]	0.872	0.661	0.806	0.936	0.871	1.148	0.707	0.797	0.343	0.584	0.463
70778812	NP_780421.2#Tmem65#74868# transmembrane protein 65 [Mus musculus]	1.040	0.950	0.758	0.984	0.871	1.148	0.829	1.012	0.749	1.047	0.898
254675197	NP_038671.2#Ptpn5#19259# tyrosine-protein phosphatase non-receptor type 5 [Mus musculus]	1.122	1.038	0.833	0.909	0.871	1.148	0.947	1.008	0.811	1.219	1.015
11096336	NP_068361.1#Chst7#60322# carbohydrate sulfotransferase 7 precursor [Mus musculus]	1.113	0.900	0.809	0.934	0.871	1.148	0.990	0.948	0.734	1.010	0.872
20070422	NP_613068.1#Praf2#54637# PRA1 family protein 2 [Mus musculus]	1.489	0.963	0.741	1.002	0.872	1.147	1.158	1.438	1.030	1.470	1.250
254540041	NP_080223.2#Dynlrb1#67068# dynein light chain roadblock-type 1 isoform b [Mus musculus]	0.925	0.969	0.716	1.028	0.872	1.147	0.766	0.847	0.628	0.930	0.779
407027854	NP_001258335.1#Capzb#12345# F-actin-capping protein subunit beta isoform d [Mus musculus]	1.014	0.898	0.791	0.953	0.872	1.147	0.818	0.854	0.674	0.839	0.757
32469489	NP_862898.1#Grm3#108069# metabotropic glutamate receptor 3 precursor [Mus musculus]	1.235	0.773	0.802	0.942	0.872	1.147	1.033	1.122	0.727	0.937	0.832
411147387	NP_598912.4#Yars#107271# tyrosine--tRNA ligase, cytoplasmic [Mus musculus]	1.170	0.969	0.792	0.952	0.872	1.147	0.981	1.113	0.874	1.165	1.019
568988186	XP_006519322.1#Dhrs1#52585# PREDICTED: dehydrogenase/reductase SDR family member 1 isoform X1 [Mus musculus]	1.077	0.990	0.782	0.963	0.872	1.147	0.885	0.993	0.733	1.128	0.930
13384616	NP_079553.1#Gng10#14700# guanine nucleotide-binding protein G(I)/G(S)/G(O) subunit gamma-10 precursor [Mus musculus]	1.219	0.896	0.754	0.991	0.872	1.146	0.929	1.198	0.806	1.100	0.953
568960115	XP_006510692.1#Scn2b#72821# PREDICTED: sodium channel subunit beta-2 isoform X2 [Mus musculus]	0.857	0.918	0.745	1.000	0.873	1.146	0.638	0.858	0.629	0.744	0.687
568979853	XP_006516032.1#Nrcam#319504# PREDICTED: neuronal cell adhesion molecule isoform X21 [Mus musculus]	0.988	0.922	0.762	0.983	0.873	1.146	0.830	0.961	0.699	0.965	0.832
226443091	NP_084148.1#Hnrnpa0#77134# heterogeneous nuclear ribonucleoprotein A0 [Mus musculus]	1.037	0.894	0.729	1.016	0.873	1.146	0.826	0.985	0.650	0.969	0.809
6680832	NP_031615.1#Calm2#12314# calmodulin [Mus musculus]	0.535	0.859	0.777	0.969	0.873	1.146	0.419	0.530	0.373	0.477	0.425
6681137	NP_031856.1#Dbi#13167# acyl-CoA-binding protein isoform 2 [Mus musculus]	0.882	1.113	0.765	0.981	0.873	1.145	0.738	0.784	0.740	0.898	0.819
568961317	XP_006511152.1#Lingo1#235402# PREDICTED: leucine-rich repeat and immunoglobulin-like domain-containing nogo receptor-interacting protein 1 is	1.011	0.821	0.760	0.986	0.873	1.145	0.833	1.041	0.652	0.859	0.755
568955031	XP_006509585.1#Micu3#78506# PREDICTED: calcium uptake protein 3, mitochondrial isoform X1 [Mus musculus]	1.207	0.812	0.734	1.012	0.873	1.145	0.956	1.150	0.725	0.986	0.855
568979767	XP_006515990.1#Ppp2r5e#26932# PREDICTED: serine/threonine-protein phosphatase 2A 56 kDa regulatory subunit epsilon isoform isoform X3 [Mus	1.066	0.905	0.771	0.975	0.873	1.145	0.820	1.042	0.748	0.935	0.842
18859597	NP_077182.1#Ndufc2#68197# NADH dehydrogenase [ubiquinone] 1 subunit C2 [Mus musculus]	1.052	0.942	0.776	0.971	0.873	1.145	0.830	0.930	0.702	0.928	0.815
568962755	XP_006511697.1#Bsn#12217# PREDICTED: protein bassoon isoform X1 [Mus musculus]	1.190	0.857	0.765	0.981	0.873	1.145	0.945	1.138	0.773	0.963	0.868
356582492	NP_001239217.1#Ogdh#18293# 2-oxoglutarate dehydrogenase, mitochondrial isoform 4 [Mus musculus]	1.254	0.931	0.774	0.973	0.873	1.145	0.987	1.167	0.882	1.111	0.996
25141233	NP_034901.2#Matr3#17184# matrin-3 [Mus musculus]	1.132	0.885	0.751	0.996	0.873	1.145	0.877	1.070	0.740	1.005	0.873
226823367	NP_034339.2#Fh1#14194# fumarate hydratase, mitochondrial precursor [Mus musculus]	1.096	0.924	0.763	0.984	0.873	1.145	0.814	1.010	0.692	0.963	0.827
24233554	NP_683740.1#Slc1a3#20512# excitatory amino acid transporter 1 [Mus musculus]	1.041	0.916	0.776	0.971	0.873	1.145	0.817	1.000	0.729	0.941	0.835
568990408	XP_006520108.1#March11#211147# PREDICTED: E3 ubiquitin-protein ligase MARCH11 isoform X1 [Mus musculus]	1.061	0.888	0.771	0.976	0.874	1.145	0.857	0.996	0.731	0.915	0.823
9790141	NP_062798.1#Arpc3#56378# actin-related protein 2/3 complex subunit 3 [Mus musculus]	0.812	0.863	0.756	0.991	0.874	1.145	0.632	0.755	0.525	0.672	0.599
568960036	XP_006510653.1#Sik3#70661# PREDICTED: serine/threonine-protein kinase SIK3 isoform X1 [Mus musculus]	0.942	0.936	0.792	0.956	0.874	1.145	0.769	0.877	0.702	0.838	0.770
6755368	NP_035426.1#Rps18#20084# 40S ribosomal protein S18 [Mus musculus]	0.847	0.863	0.738	1.010	0.874	1.144	0.682	0.791	0.562	0.716	0.639
347582617	NP_033139.2#S100a13#20196# protein S100-A13 [Mus musculus]	1.039	0.977	0.789	0.959	0.874	1.144	0.804	1.012	0.792	0.983	0.888
6754024	NP_034447.1#Gng4#14706# guanine nucleotide-binding protein G(I)/G(S)/G(O) subunit gamma-4 precursor [Mus musculus]	1.114	1.091	0.754	0.994	0.874	1.144	0.847	1.100	0.856	1.127	0.992
568979680	XP_006515947.1#Psma6#26443# PREDICTED: proteasome subunit alpha type-6 isoform X1 [Mus musculus]	1.010	0.767	0.786	0.962	0.874	1.144	0.795	0.983	0.575	0.773	0.674

6671519	NP_031447.1#Adssl1#11565# adenylosuccinate synthetase isozyme 1 [Mus musculus]	1.050	0.970	0.752	0.997	0.874	1.144	0.813	1.069	0.765	1.035	0.900
13385680	NP_080448.1#Decr1#67460# 2,4-dienoyl-CoA reductase, mitochondrial precursor [Mus musculus]	0.965	0.949	0.772	0.977	0.875	1.143	0.790	0.898	0.723	0.879	0.801
6679821	NP_032059.1#Fnta#14272# protein farnesyltransferase/geranylgeranyltransferase type-1 subunit alpha [Mus musculus]	1.349	0.877	0.671	1.079	0.875	1.143	1.065	1.294	0.815	1.255	1.035
241982771	NP_001001983.2#Pi4ka#224020# phosphatidylinositol 4-kinase alpha [Mus musculus]	1.154	0.880	0.768	0.981	0.875	1.143	0.963	1.125	0.753	0.994	0.873
568918685	XP_006500377.1#Scrn3#74616# PREDICTED: secernin-3 isoform X1 [Mus musculus]	1.242	0.849	0.805	0.945	0.875	1.143	1.009	1.163	0.769	1.076	0.922
262263372	NP_057870.3#Ahcy#269378# adenosylhomocysteinase [Mus musculus]	1.034	0.916	0.788	0.962	0.875	1.143	0.813	0.998	0.703	0.955	0.829
569011419	XP_006528639.1#Tceal5#331532# PREDICTED: transcription elongation factor A protein-like 5 isoform X1 [Mus musculus]	1.096	0.953	0.767	0.983	0.875	1.143	0.774	1.035	0.785	0.981	0.883
755544182	XP_011242598.1#Ryr2#20191# PREDICTED: ryanodine receptor 2 isoform X2 [Mus musculus]	1.133	0.838	0.791	0.959	0.875	1.143	0.895	1.090	0.722	0.952	0.837
162417975	NP_034627.3#Idh1#15926# isocitrate dehydrogenase [NADP] cytoplasmic [Mus musculus]	0.907	0.960	0.812	0.938	0.875	1.143	0.701	0.887	0.625	0.900	0.762
9790125	NP_062728.1#Tagln3#56370# transgelin-3 [Mus musculus]	1.069	0.887	0.760	0.990	0.875	1.143	0.813	0.929	0.684	0.876	0.780
19527140	NP_598687.1#Camk1#52163# calcium/calmodulin-dependent protein kinase type 1 [Mus musculus]	1.076	0.926	0.771	0.979	0.875	1.143	0.894	0.988	0.667	1.075	0.871
6753136	NP_033850.1#Atox1#11927# copper transport protein ATOX1 [Mus musculus]	1.062	0.948	0.790	0.960	0.875	1.143	0.852	1.013	0.788	1.043	0.916
6680722	NP_031506.1#Arf5#11844# ADP-ribosylation factor 5 [Mus musculus]	1.052	0.955	0.775	0.975	0.875	1.143	0.759	1.082	0.749	1.009	0.879
356640210	NP_001239262.1#Sept8#20362# septin-8 isoform 3 [Mus musculus]	1.061	0.810	0.761	0.989	0.875	1.143	0.866	0.992	0.664	0.841	0.753
188035915	NP_001120810.1#Aldh7a1#110695# alpha-aminoadipic semialdehyde dehydrogenase isoform b [Mus musculus]	1.167	0.887	0.783	0.967	0.875	1.142	0.944	1.099	0.765	1.045	0.905
56790900	NP_001007222.1#Adam22#11496# disintegrin and metalloproteinase domain-containing protein 22 isoform b precursor [Mus musculus]	1.036	0.884	0.786	0.965	0.876	1.142	0.818	0.933	0.662	0.852	0.757
568926471	XP_006537875.1#N28178#230085# PREDICTED: protein KIAA1045 isoform X3 [Mus musculus]	1.019	0.822	0.747	1.005	0.876	1.142	0.793	0.933	0.620	0.826	0.723
227330561	NP_034227.3#Sparcl1#13602# SPARC-like protein 1 precursor [Mus musculus]	1.077	0.939	0.732	1.020	0.876	1.142	0.887	1.001	0.813	0.960	0.886
755512767	XP_011247846.1#Slc4a4#54403# PREDICTED: electrogenic sodium bicarbonate cotransporter 1 isoform X6 [Mus musculus]	1.136	1.062	0.796	0.955	0.876	1.142	0.859	1.007	0.848	1.075	0.962
21450277	NP_659149.1#Atp1a1#11928# sodium/potassium-transporting ATPase subunit alpha-1 precursor [Mus musculus]	1.351	0.794	0.764	0.988	0.876	1.142	1.053	1.260	0.791	1.117	0.954
31982091	NP_766197.2#Tomm22#223696# mitochondrial import receptor subunit TOM22 homolog [Mus musculus]	1.189	0.903	0.790	0.962	0.876	1.141	0.884	1.200	0.843	1.039	0.941
551894157	NP_001272754.1#Snrpa#53607# U1 small nuclear ribonucleoprotein A [Mus musculus]	1.138	0.791	0.742	1.011	0.876	1.141	0.982	1.013	0.698	0.881	0.789
160333280	NP_001103746.1#Itsn1#16443# intersectin-1 isoform 3 [Mus musculus]	1.034	0.937	0.735	1.018	0.876	1.141	0.794	1.018	0.740	0.957	0.849
261878543	NP_620084.2#Mthfd1#108156# C-1-tetrahydrofolate synthase, cytoplasmic [Mus musculus]	0.860	0.812	0.812	0.941	0.876	1.141	0.766	0.969	0.594	0.797	0.695
255958318	NP_001157670.1#Prkacb#18749# cAMP-dependent protein kinase catalytic subunit beta isoform 2 [Mus musculus]	0.757	0.865	0.769	0.984	0.876	1.141	0.690	0.674	0.510	0.670	0.590
33667095	NP_766604.2#Vat1l#270097# synaptic vesicle membrane protein VAT-1 homolog-like [Mus musculus]	0.930	1.036	0.800	0.954	0.877	1.140	0.760	0.869	0.721	0.968	0.844
33468857	NP_032274.1#Hint1#15254# histidine triad nucleotide-binding protein 1 [Mus musculus]	1.018	0.933	0.814	0.940	0.877	1.140	0.846	0.978	0.720	0.896	0.808
755499054	XP_011237700.1#Slc1a2#20511# PREDICTED: excitatory amino acid transporter 2 isoform X1 [Mus musculus]	1.593	0.836	0.775	0.979	0.877	1.140	1.167	1.429	1.004	1.279	1.142
171543899	NP_786926.2#Plxna4#243743# plexin-A4 precursor [Mus musculus]	1.228	0.918	0.807	0.948	0.877	1.140	0.948	1.053	0.785	0.963	0.874
158966704	NP_038675.2#Rps16#20055# 40S ribosomal protein S16 [Mus musculus]	0.898	0.878	0.771	0.984	0.877	1.140	0.700	0.829	0.563	0.744	0.654
568908101	XP_006529181.1#Dhx9#13211# PREDICTED: ATP-dependent RNA helicase A isoform X2 [Mus musculus]	1.298	0.846	0.772	0.983	0.878	1.140	0.997	1.199	0.862	1.028	0.945
33942059	NP_033384.2#Ppp2r5d#21770# serine/threonine-protein phosphatase 2A 56 kDa regulatory subunit delta isoform [Mus musculus]	1.194	0.892	0.738	1.017	0.878	1.140	0.903	1.192	0.740	1.128	0.934
120300980	NP_683745.2#Usp9y#107868# ubiquitin specific peptidase 9, Y chromosome [Mus musculus]	1.504	0.926	0.824	0.932	0.878	1.140	1.236	1.404	1.071	1.375	1.223
33504483	NP_084043.1#Rps9#76846# 40S ribosomal protein S9 [Mus musculus]	0.860	0.842	0.762	0.994	0.878	1.139	0.696	0.836	0.584	0.725	0.654
568995065	XP_006522067.1#Ube2l3#22195# PREDICTED: ubiquitin-conjugating enzyme E2 L3 isoform X1 [Mus musculus]	1.099	0.900	0.810	0.946	0.878	1.139	0.820	1.014	0.734	0.952	0.843
755560327	XP_011245096.1#Abhd3#106861# PREDICTED: abhydrolase domain-containing protein 3 isoform X2 [Mus musculus]	1.096	0.970	0.705	1.051	0.878	1.139	0.871	1.054	0.780	1.088	0.934
755565097	XP_011245805.1#Ogt#108155# PREDICTED: UDP-N-acetylglucosamine--peptide N-acetylglucosaminyltransferase 110 kDa subunit isoform X1 [Mus m	1.051	0.866	0.792	0.964	0.878	1.139	0.831	0.973	0.683	0.882	0.782
568904641	XP_006544819.1#LOC102642938#102642938# PREDICTED: heterogeneous nuclear ribonucleoproteins A2/B1 isoform X2 [Mus musculus]	1.010	0.912	0.760	0.998	0.879	1.138	0.828	1.071	0.755	0.948	0.852
29789289	NP_444349.1#Echs1#93747# enoyl-CoA hydratase, mitochondrial precursor [Mus musculus]	0.992	0.935	0.828	0.930	0.879	1.138	0.903	1.035	0.858	1.015	0.936
116256516	NP_001070735.1#Hnrnpd#11991# heterogeneous nuclear ribonucleoprotein D0 isoform d [Mus musculus]	1.001	0.886	0.789	0.969	0.879	1.138	0.827	0.932	0.627	0.931	0.779
31981939	NP_033477.2#Tubb4a#22153# tubulin beta-4A chain [Mus musculus]	1.172	0.934	0.742	1.016	0.879	1.138	0.927	1.199	0.943	1.045	0.994
176865892	NP_038534.2#Eif4a2#13682# eukaryotic initiation factor 4A-II isoform a [Mus musculus]	1.144	0.893	0.854	0.905	0.879	1.137	0.914	1.108	0.812	1.017	0.914
568975949	XP_006534343.1#Btbd17#72014# PREDICTED: BTB/POZ domain-containing protein 17 isoform X3 [Mus musculus]	1.197	0.847	0.811	0.947	0.879	1.137	0.989	1.115	0.822	0.960	0.891
569010246	XP_006528149.1#Atp2b3#320707# PREDICTED: plasma membrane calcium-transporting ATPase 3 isoform X6 [Mus musculus]	1.085	0.899	0.721	1.038	0.879	1.137	0.831	1.003	0.650	0.952	0.801
162138894	NP_079924.2#Farsa#66590# phenylalanine--tRNA ligase alpha subunit [Mus musculus]	1.040	0.835	0.762	0.997	0.879	1.137	0.903	0.925	0.613	0.915	0.764
755566692	XP_011246162.1#Huwe1#59026# PREDICTED: E3 ubiquitin-protein ligase HUWE1 isoform X9 [Mus musculus]	1.050	0.927	0.762	0.997	0.879	1.137	0.764	1.101	0.614	0.893	0.754
6754994	NP_035995.1#Pcbp1#23983# poly(rC)-binding protein 1 [Mus musculus]	1.186	0.836	0.800	0.960	0.880	1.136	0.970	1.175	0.849	1.154	1.002
116256491	NP_733924.2#Ank3#11735# ankyrin-3 isoform a [Mus musculus]	0.936	0.908	0.825	0.935	0.880	1.136	0.807	0.882	0.713	0.791	0.752
31560819	NP_032446.2#Kcnb1#16500# potassium voltage-gated channel subfamily B member 1 [Mus musculus]	1.079	0.894	0.765	0.995	0.880	1.136	0.899	0.999	0.775	0.923	0.849
568980440	XP_006516316.1#Snx6#72183# PREDICTED: sorting nexin-6 isoform X1 [Mus musculus]	0.927	0.930	0.795	0.965	0.880	1.136	0.799	0.833	0.701	0.817	0.759
568988763	XP_006519603.1#Ipo5#70572# PREDICTED: importin-5 isoform X3 [Mus musculus]	1.071	0.854	0.776	0.984	0.880	1.136	0.887	1.070	0.770	0.982	0.876
568916866	XP_006499503.1#Stx16#228960# PREDICTED: syntaxin-16 isoform X2 [Mus musculus]	1.150	0.856	0.775	0.986	0.880	1.136	0.875	1.148	0.793	0.939	0.866
171906578	NP_033037.2#Rad23b#19359# UV excision repair protein RAD23 homolog B [Mus musculus]	0.934	0.890	0.768	0.993	0.880	1.136	0.731	0.935	0.699	0.829	0.764
569012474	XP_006531674.1#Eif2s3y#26908# PREDICTED: eukaryotic translation initiation factor 2 subunit 3, Y-linked isoform X3 [Mus musculus]	1.129	0.892	0.777	0.984	0.880	1.136	0.932	1.056	0.809	0.963	0.886
88900515	NP_082185.2#C2cd2l#71764# C2 domain-containing protein 2-like [Mus musculus]	1.048	0.894	0.762	0.999	0.881	1.136	0.894	0.951	0.678	0.971	0.825
319402143	NP_001188320.1#Hbb-bs#100503605# hemoglobin, beta adult s chain [Mus musculus]	**2.094**	1.002	0.818	0.943	0.881	1.136	1.636	2.050	1.478	2.158	1.818
21313162	NP_083852.1#Rab1b#76308# ras-related protein Rab-1B [Mus musculus]	1.068	0.903	0.799	0.962	0.881	1.135	0.828	0.953	0.689	0.961	0.825
568979727	XP_006515970.1#Gphn#268566# PREDICTED: gephyrin isoform X9 [Mus musculus]	1.116	0.902	0.808	0.954	0.881	1.135	0.884	1.101	0.746	1.079	0.913
568965600	XP_006512894.1#Soga3#67412# PREDICTED: protein SOGA3 isoform X2 [Mus musculus]	1.014	0.943	0.789	0.973	0.881	1.135	0.821	0.965	0.736	0.948	0.842
23956058	NP_035253.1#Plp1#18823# myelin proteolipid protein isoform 1 [Mus musculus]	0.978	1.098	0.788	0.975	0.881	1.135	0.792	0.916	0.811	1.086	0.948
568980537	XP_006516361.1#Isca2#74316# PREDICTED: iron-sulfur cluster assembly 2 homolog, mitochondrial isoform X3 [Mus musculus]	1.115	0.860	0.767	0.996	0.881	1.135	0.847	1.117	0.696	0.993	0.845
6754220	NP_034577.1#Hnrnpa1#15382# heterogeneous nuclear ribonucleoprotein A1 isoform a [Mus musculus]	1.069	0.830	0.777	0.986	0.881	1.135	0.787	0.985	0.626	0.897	0.761
71043944	NP_062701.2#Snx1#56440# sorting nexin-1 [Mus musculus]	1.014	0.911	0.820	0.942	0.881	1.135	0.838	0.954	0.733	0.916	0.824
568909683	XP_006529945.1#Cyb5r1#72017# PREDICTED: NADH-cytochrome b5 reductase 1 isoform X1 [Mus musculus]	1.156	0.883	0.814	0.949	0.882	1.134	0.917	1.122	0.761	1.039	0.900
161484668	NP_766295.2#Ppp1cb#19046# serine/threonine-protein phosphatase PP1-beta catalytic subunit [Mus musculus]	1.007	0.937	0.762	1.001	0.882	1.134	0.789	0.957	0.691	0.959	0.825
568993633	XP_006521604.1#Sbf1#77980# PREDICTED: myotubularin-related protein 5 isoform X5 [Mus musculus]	1.180	0.892	0.783	0.980	0.882	1.134	0.950	1.145	0.851	1.067	0.959
88014720	NP_032405.3#Kpnb1#16211# importin subunit beta-1 [Mus musculus]	1.154	0.913	0.778	0.985	0.882	1.134	0.927	1.104	0.823	1.104	0.963
568979000	XP_006515626.1#Nrxn3#18191# PREDICTED: neurexin 3 isoform X21 [Mus musculus]	1.003	0.863	0.752	1.011	0.882	1.134	0.842	0.926	0.696	0.831	0.763
568974509	XP_006533648.1#Myo1d#338367# PREDICTED: unconventional myosin-Id isoform X1 [Mus musculus]	1.087	1.073	0.828	0.935	0.882	1.134	0.857	1.137	0.917	1.210	1.064
257900520	NP_064323.2#Mpp6#56524# MAGUK p55 subfamily member 6 isoform b [Mus musculus]	1.024	0.929	0.789	0.975	0.882	1.134	0.835	0.972	0.784	0.892	0.838
157266281	NP_079977.3#Trappc5#66682# trafficking protein particle complex subunit 5 [Mus musculus]	0.861	0.905	0.820	0.944	0.882	1.134	0.659	0.859	0.608	0.765	0.687
568982851	XP_006517069.1#Hist1h2al#667728# PREDICTED: histone H2A type 2-A, partial [Mus musculus]	0.740	1.022	0.811	0.954	0.882	1.133	0.616	0.733	0.597	0.778	0.688
251823874	NP_001156507.1#Enoph1#67870# enolase-phosphatase E1 [Mus musculus]	0.931	0.900	0.680	1.085	0.882	1.133	0.727	0.916	0.637	0.842	0.739
89001109	NP_034433.3#Gna13#14674# guanine nucleotide-binding protein subunit alpha-13 [Mus musculus]	1.125	0.915	0.824	0.941	0.883	1.133	0.853	1.059	0.748	1.003	0.876
255308888	NP_062704.2#Nme3#79059# nucleoside diphosphate kinase 3 precursor [Mus musculus]	1.005	1.036	0.777	0.988	0.883	1.133	0.827	0.948	0.786	1.053	0.919
85861186	NP_031493.2#Aplp1#11803# amyloid-like protein 1 precursor [Mus musculus]	1.116	0.815	0.677	1.089	0.883	1.133	0.912	1.059	0.705	0.902	0.803
16716503	NP_444429.1#Cadm3#94332# cell adhesion molecule 3 precursor [Mus musculus]	1.306	0.890	0.785	0.982	0.883	1.132	1.013	1.346	0.926	1.174	1.050
12963737	NP_076054.1#Cse1l#110750# exportin-2 [Mus musculus]	1.114	0.874	0.766	1.001	0.883	1.132	0.897	1.068	0.769	0.951	0.860
451327597	NP_001263390.1#Asap1#13196# arf-GAP with SH3 domain, ANK repeat and PH domain-containing protein 1 isoform b [Mus musculus]	1.074	0.916	0.778	0.989	0.884	1.132	0.918	0.980	0.726	1.013	0.870
31982724	NP_058056.2#Mybbp1a#18432# myb-binding protein 1A [Mus musculus]	1.095	0.885	0.809	0.958	0.884	1.132	0.836	1.099	0.754	0.959	0.857
594191015	NP_001277432.1#Zer1#227693# protein zer-1 homolog isoform b [Mus musculus]	1.019	0.839	0.726	1.042	0.884	1.131	0.834	0.968	0.619	0.894	0.756
113199755	NP_598733.2#Armc6#76813# armadillo repeat-containing protein 6 [Mus musculus]	1.003	0.837	0.733	1.036	0.884	1.131	0.722	1.051	0.649	0.834	0.742
568992018	XP_006520824.1#Fam49b#223601# PREDICTED: protein FAM49B isoform X1 [Mus musculus]	0.942	0.933	0.749	1.019	0.884	1.131	0.797	0.894	0.676	0.922	0.799

568913154	XP_006497848.1#Psmb7#19177# PREDICTED: proteasome subunit beta type-7 isoform X1 [Mus musculus]	0.894	0.730	0.800	0.969	0.884	1.131	0.667	0.914	0.508	0.645	0.577
568995370	XP_006522210.1#Mapk1#26413# PREDICTED: mitogen-activated protein kinase 1 isoform X1 [Mus musculus]	0.939	0.917	0.745	1.024	0.884	1.131	0.796	0.908	0.705	0.835	0.770
37700232	NP_032730.1#Nme1#18102# nucleoside diphosphate kinase A [Mus musculus]	1.022	0.912	0.784	0.986	0.885	1.130	0.829	0.978	0.697	0.903	0.800
755547658	XP_011243224.1#Pcca#110821# PREDICTED: propionyl-CoA carboxylase alpha chain, mitochondrial isoform X4 [Mus musculus]	0.980	0.890	0.831	0.938	0.885	1.130	0.848	0.971	0.763	0.943	0.853
163954948	NP_034743.3#Khsrp#16549# far upstream element-binding protein 2 [Mus musculus]	1.046	0.880	0.783	0.986	0.885	1.130	0.875	0.990	0.738	0.997	0.867
228008337	NP_032974.2#Psmc3#19182# 26S protease regulatory subunit 6A [Mus musculus]	1.027	0.896	0.803	0.967	0.885	1.130	0.855	0.988	0.775	1.025	0.900
568969703	XP_006514495.1#Xpo1#103573# PREDICTED: exportin-1 isoform X3 [Mus musculus]	1.070	0.819	0.774	0.996	0.885	1.130	0.957	0.937	0.612	0.938	0.775
568965315	XP_006512756.1#Clvs2#215890# PREDICTED: clavesin-2 isoform X1 [Mus musculus]	1.091	0.836	0.839	0.932	0.885	1.130	0.822	1.109	0.648	0.968	0.808
134053905	NP_848731.2#Psmd11#69077# 26S proteasome non-ATPase regulatory subunit 11 [Mus musculus]	0.983	0.935	0.775	0.995	0.885	1.130	0.785	0.955	0.726	0.900	0.813
568940569	XP_006505556.1#Dctn1#13191# PREDICTED: dynactin subunit 1 isoform X9 [Mus musculus]	1.095	0.884	0.791	0.979	0.885	1.130	0.872	1.075	0.713	0.963	0.838
568965813	XP_006512991.1#Stxbp5#78808# PREDICTED: syntaxin-binding protein 5 isoform X3 [Mus musculus]	1.302	0.782	0.712	1.059	0.885	1.129	0.991	1.199	0.792	0.962	0.877
358439483	NP_001240610.1#Immt#76614# MICOS complex subunit Mic60 isoform 2 [Mus musculus]	1.116	0.914	0.766	1.004	0.885	1.129	0.922	1.073	0.787	1.046	0.916
31560239	NP_079886.2#Asrgl1#66514# isoaspartyl peptidase/L-asparaginase [Mus musculus]	0.856	0.937	0.737	1.033	0.885	1.129	0.644	0.871	0.608	0.813	0.710
483968030	NP_001264827.1#Prkaca#18747# cAMP-dependent protein kinase catalytic subunit alpha isoform 2 [Mus musculus]	1.044	0.913	0.782	0.989	0.886	1.129	0.856	0.993	0.716	0.972	0.844
158711686	NP_065066.2#Slc12a5#57138# solute carrier family 12 member 5 [Mus musculus]	1.103	0.891	0.785	0.987	0.886	1.129	0.940	1.063	0.763	0.985	0.874
30520131	NP_848818.1#Slc6a1#232333# sodium- and chloride-dependent GABA transporter 1 [Mus musculus]	0.976	0.852	0.785	0.987	0.886	1.129	0.761	0.969	0.633	0.840	0.737
165377065	NP_079923.3#Cmpk1#66588# UMP-CMP kinase [Mus musculus]	0.958	0.928	0.742	1.030	0.886	1.128	0.850	0.893	0.654	0.926	0.790
28372479	NP_077228.1#Rps25#75617# 40S ribosomal protein S25 [Mus musculus]	1.025	0.892	0.791	0.981	0.886	1.128	0.883	0.990	0.705	0.960	0.833
6755983	NP_036168.1#Vsnl1#26950# visinin-like protein 1 [Mus musculus]	1.251	0.877	0.790	0.983	0.886	1.128	0.990	1.226	0.843	1.123	0.983
241982783	NP_766086.2#Ptk2b#19229# protein-tyrosine kinase 2-beta isoform 3 [Mus musculus]	1.034	0.894	0.786	0.987	0.887	1.128	0.853	0.984	0.732	0.911	0.821
283135110	NP_758512.3#Mthfd1l#270685# monofunctional C1-tetrahydrofolate synthase, mitochondrial precursor [Mus musculus]	1.217	0.872	0.781	0.993	0.887	1.128	0.943	1.199	0.751	1.045	0.898
58037465	NP_084027.1#Rpl18a#76808# 60S ribosomal protein L18a [Mus musculus]	1.369	1.003	0.820	0.954	0.887	1.128	1.020	1.408	0.981	1.455	1.218
568942420	XP_006506449.1#Copg1#54161# PREDICTED: coatomer subunit gamma-1 isoform X1 [Mus musculus]	1.242	0.935	0.783	0.992	0.887	1.127	1.003	1.172	0.865	1.156	1.010
459683868	NP_001264051.1#Hnrnpr#74326# heterogeneous nuclear ribonucleoprotein R isoform b [Mus musculus]	0.984	0.802	0.764	1.011	0.887	1.127	0.833	0.948	0.582	0.819	0.700
568961907	XP_006511433.1#Pccb#66904# PREDICTED: propionyl-CoA carboxylase beta chain, mitochondrial isoform X2 [Mus musculus]	1.075	0.922	0.756	1.019	0.887	1.127	0.823	0.993	0.762	0.951	0.856
569007898	XP_006527442.1#Hspa12a#73442# PREDICTED: heat shock 70 kDa protein 12A isoform X4 [Mus musculus]	1.113	0.917	0.795	0.981	0.888	1.127	0.897	1.079	0.762	1.033	0.898
7305107	NP_038563.1#Grcc10#14790# protein C10 [Mus musculus]	0.958	0.933	0.767	1.009	0.888	1.127	0.698	1.004	0.705	0.882	0.794
568967854	XP_006513852.1#Ppfia2#327814# PREDICTED: liprin-alpha-2 isoform X16 [Mus musculus]	1.106	0.869	0.757	1.019	0.888	1.126	0.851	1.113	0.742	0.965	0.853
755513544	XP_006530271.2#Rasal1#19415# PREDICTED: rasGAP-activating-like protein 1 isoform X2 [Mus musculus]	1.121	0.830	0.701	1.075	0.888	1.126	0.839	1.037	0.682	0.827	0.755
755525950	XP_011240608.1#Hapln4#330790# PREDICTED: hyaluronan and proteoglycan link protein 4 isoform X1 [Mus musculus]	1.058	0.840	0.750	1.026	0.888	1.126	0.865	1.014	0.725	0.854	0.790
568973607	XP_006533222.1#Ubb#22187# PREDICTED: polyubiquitin-B isoform X1 [Mus musculus]	1.222	0.952	0.838	0.939	0.888	1.126	0.953	1.229	0.793	1.155	0.974
36054013	NP_109614.2#Nptxr#73340# neuronal pentraxin receptor [Mus musculus]	1.018	0.803	0.795	0.981	0.888	1.126	0.802	1.006	0.680	0.772	0.726
263195218	NP_001160108.1#Gm4312#100043247# uncharacterized protein LOC100043247 [Mus musculus]	1.132	0.768	0.815	0.962	0.888	1.126	0.930	1.082	0.693	0.852	0.772
21539599	NP_079917.1#Uqcrh#66576# cytochrome b-c1 complex subunit 6, mitochondrial [Mus musculus]	1.228	0.891	0.764	1.013	0.889	1.125	1.044	1.189	0.872	1.173	1.023
38142460	NP_080971.2#Etfb#110826# electron transfer flavoprotein subunit beta [Mus musculus]	1.251	0.866	0.811	0.966	0.889	1.125	1.066	1.210	0.848	1.123	0.986
255069715	NP_077754.3#Lap3#66988# cytosol aminopeptidase [Mus musculus]	0.855	0.995	0.746	1.031	0.889	1.125	0.721	0.800	0.665	0.847	0.756
12025542	NP_062626.1#Asna1#56495# ATPase Asna1 [Mus musculus]	1.071	0.943	0.808	0.970	0.889	1.125	0.889	1.015	0.778	0.995	0.886
148238335	NP_062357.3#Alyref2#56009# aly/REF export factor 2 [Mus musculus]	0.995	0.937	0.744	1.033	0.889	1.125	0.802	0.967	0.738	0.920	0.829
117938284	NP_033188.3#Scg5#20394# neuroendocrine protein 7B2 precursor [Mus musculus]	1.244	0.869	0.810	0.968	0.889	1.125	0.978	1.235	0.828	1.095	0.961
568931106	XP_006538866.1#Crocc#230872# PREDICTED: rootletin isoform X3 [Mus musculus]	0.710	0.817	0.946	0.832	0.889	1.125	0.625	0.638	0.467	0.564	0.516
568965003	XP_006512604.1#Gja1#14609# PREDICTED: gap junction alpha-1 protein isoform X1 [Mus musculus]	1.157	0.972	0.754	1.024	0.889	1.125	0.838	0.971	0.785	0.973	0.879
17933768	NP_525028.1#Grhpr#76238# glyoxylate reductase/hydroxypyruvate reductase [Mus musculus]	1.093	0.925	0.807	0.971	0.889	1.125	0.831	1.017	0.746	0.927	0.836
300388153	NP_001177956.1#Dync1i1#13426# cytoplasmic dynein 1 intermediate chain 1 isoform 5 [Mus musculus]	1.104	0.914	0.806	0.972	0.889	1.125	0.889	1.050	0.786	0.997	0.891
111955312	NP_034948.3#Cd200#17470# OX-2 membrane glycoprotein precursor [Mus musculus]	1.002	0.837	0.798	0.980	0.889	1.124	0.837	1.038	0.672	0.937	0.805
112293266	NP_032326.3#Hspa4#15525# heat shock 70 kDa protein 4 [Mus musculus]	1.096	0.896	0.798	0.982	0.890	1.124	0.825	1.060	0.695	0.932	0.813
62526118	NP_780660.1#Ckap4#216197# cytoskeleton-associated protein 4 [Mus musculus]	1.129	0.882	0.756	1.024	0.890	1.124	0.961	1.049	0.760	1.013	0.886
18087805	NP_032529.2#Rps2#16898# 40S ribosomal protein S2 [Mus musculus]	1.046	0.848	0.816	0.964	0.890	1.124	0.811	0.994	0.645	0.872	0.759
30409956	NP_848492.1#Atp1a2#98660# sodium/potassium-transporting ATPase subunit alpha-2 precursor [Mus musculus]	1.261	0.986	0.783	0.997	0.890	1.124	0.992	1.205	0.914	1.208	1.061
568957920	XP_006531602.1#Zfp423#94187# PREDICTED: zinc finger protein 423 isoform X6 [Mus musculus]	1.075	0.850	0.745	1.035	0.890	1.123	0.829	1.085	0.719	0.909	0.814
755499302	XP_011237757.1#Madd#228355# PREDICTED: MAP kinase-activating death domain protein isoform X19 [Mus musculus]	1.133	0.845	0.774	1.006	0.890	1.123	0.847	1.172	0.770	0.937	0.853
6680083	NP_032189.1#Grb2#14784# growth factor receptor-bound protein 2 [Mus musculus]	1.052	0.975	0.828	0.953	0.890	1.123	0.815	1.058	0.805	1.022	0.914
6681095	NP_031834.1#Cycs#13063# cytochrome c, somatic [Mus musculus]	1.253	0.937	0.784	0.997	0.890	1.123	1.048	1.204	0.910	1.188	1.049
568949567	XP_006507378.1#Ctbp2#13017# PREDICTED: C-terminal-binding protein 2 isoform X3 [Mus musculus]	1.068	0.810	0.747	1.034	0.890	1.123	0.939	0.963	0.623	0.918	0.770
30520239	NP_848887.1#Nceh1#320024# neutral cholesterol ester hydrolase 1 [Mus musculus]	1.108	0.874	0.777	1.004	0.891	1.123	0.914	1.082	0.782	0.986	0.884
568933657	XP_006503744.1#Add1#11518# PREDICTED: alpha-adducin isoform X13 [Mus musculus]	1.132	0.901	0.778	1.003	0.891	1.122	0.960	1.139	0.827	1.017	0.922
575501696	NP_001276460.1#Cndp2#66054# cytosolic non-specific dipeptidase [Mus musculus]	1.068	0.908	0.805	0.977	0.891	1.122	0.873	1.069	0.748	1.008	0.878
258547146	NP_001158143.1#Dnaja1#15502# dnaJ homolog subfamily A member 1 [Mus musculus]	1.030	0.893	0.789	0.994	0.892	1.122	0.761	0.999	0.702	0.935	0.819
22122795	NP_666341.1#Dync1li1#235661# cytoplasmic dynein 1 light intermediate chain 1 [Mus musculus]	1.055	0.879	0.785	0.999	0.892	1.121	0.830	0.968	0.666	0.963	0.815
568939077	XP_006504927.1#Tecpr1#70381# PREDICTED: tectonin beta-propeller repeat-containing protein 1 isoform X1 [Mus musculus]	0.999	0.864	0.766	1.017	0.892	1.121	0.781	1.000	0.667	0.872	0.770
568944898	XP_006539622.1#Hnrnpl#15388# PREDICTED: heterogeneous nuclear ribonucleoprotein L isoform X4 [Mus musculus]	1.110	0.920	0.794	0.990	0.892	1.121	0.896	1.020	0.775	0.998	0.887
568972945	XP_006532907.1#Acsl6#216739# PREDICTED: long-chain-fatty-acid--CoA ligase 6 isoform X1 [Mus musculus]	1.125	0.925	0.799	0.985	0.892	1.121	0.975	1.102	0.812	1.126	0.969
755519133	XP_011248743.1#Mag#17136# PREDICTED: myelin-associated glycoprotein isoform X1 [Mus musculus]	0.937	1.040	0.776	1.008	0.892	1.121	0.791	0.872	0.809	1.009	0.909
254675251	NP_958792.2#Plec#18810# plectin isoform 1d [Mus musculus]	1.026	0.916	0.790	0.995	0.892	1.121	0.839	1.027	0.718	0.936	0.827
806776653	NP_001292917.1#Cnpy3#72029# protein canopy homolog 3 isoform 2 precursor [Mus musculus]	1.093	0.819	0.795	0.991	0.893	1.120	0.822	1.129	0.692	0.906	0.799
568929710	XP_006503443.1#Smap2#69780# PREDICTED: stromal membrane-associated protein 2 isoform X3 [Mus musculus]	1.123	0.847	0.730	1.056	0.893	1.120	0.935	1.070	0.787	0.911	0.849
255003777	NP_080305.2#Glod4#67201# glyoxalase domain-containing protein 4 [Mus musculus]	1.041	0.913	0.751	1.034	0.893	1.120	0.864	0.959	0.715	0.917	0.816
160298217	NP_034487.2#Gsta4#14860# glutathione S-transferase A4 [Mus musculus]	0.943	0.880	0.804	0.982	0.893	1.120	0.701	0.811	0.639	0.728	0.683
257153448	NP_038920.2#Pcsk1n#30052# proSAAS precursor [Mus musculus]	0.859	1.106	0.771	1.015	0.893	1.120	0.684	0.914	0.691	1.044	0.868
6754086	NP_034490.1#Gstm5#14866# glutathione S-transferase Mu 5 [Mus musculus]	1.071	0.887	0.815	0.970	0.893	1.120	0.852	1.059	0.780	0.962	0.871
196115327	NP_001124492.1#Gfap#14580# glial fibrillary acidic protein isoform 1 [Mus musculus]	0.724	1.216	0.806	0.980	0.893	1.120	0.607	0.741	0.676	0.873	0.775
160333789	NP_035597.2#Spr#20751# sepiapterin reductase [Mus musculus]	1.097	0.896	0.803	0.983	0.893	1.120	0.834	1.127	0.687	0.950	0.819
54873617	NP_796141.2#Ppm1e#320472# protein phosphatase 1E [Mus musculus]	1.018	0.987	0.760	1.028	0.894	1.119	0.857	0.963	0.734	1.057	0.895
244791124	NP_598564.2#Dpp3#75221# dipeptidyl peptidase 3 [Mus musculus]	1.145	0.903	0.769	1.019	0.894	1.119	0.943	1.104	0.785	1.064	0.925
568923063	XP_006501675.1#Pigk#329777# PREDICTED: GPI-anchor transamidase isoform X1 [Mus musculus]	0.990	0.899	0.760	1.028	0.894	1.118	0.819	0.952	0.718	0.874	0.796
116734870	NP_032543.2#Lta4h#16993# leukotriene A-4 hydrolase [Mus musculus]	1.176	0.906	0.788	1.001	0.894	1.118	0.928	1.176	0.815	1.092	0.953
568958996	XP_006510147.1#Pafah1b2#18475# PREDICTED: platelet-activating factor acetylhydrolase IB subunit beta isoform X1 [Mus musculus]	1.101	1.104	0.741	1.048	0.894	1.118	0.825	1.143	1.003	1.171	1.087
45597447	NP_035564.1#Sod1#20655# superoxide dismutase [Cu-Zn] [Mus musculus]	0.863	0.889	0.802	0.987	0.894	1.118	0.723	0.807	0.588	0.747	0.667
6678768	NP_032564.1#Marcks#17118# myristoylated alanine-rich C-kinase substrate [Mus musculus]	1.034	0.949	0.774	1.014	0.894	1.118	0.885	0.962	0.734	1.023	0.878
161484634	NP_031770.2#Comt#12846# catechol O-methyltransferase [Mus musculus]	1.116	0.856	0.868	0.921	0.895	1.118	0.882	1.115	0.735	0.975	0.855
31981310	NP_036190.2#Bcap31#27061# B-cell receptor-associated protein 31 [Mus musculus]	1.193	0.783	0.718	1.072	0.895	1.118	0.975	1.159	0.768	0.903	0.835
37202121	NP_766549.2#Abat#268860# 4-aminobutyrate aminotransferase, mitochondrial isoform 1 precursor [Mus musculus]	1.197	1.070	0.839	0.951	0.895	1.118	0.977	1.103	0.960	1.286	1.123
26006861	NP_742146.1#Pdxk#216134# pyridoxal kinase [Mus musculus]	1.145	1.002	0.754	1.036	0.895	1.117	0.818	1.043	0.806	1.088	0.947
597955354	NP_001278040.1#Gss#14854# glutathione synthetase isoform b [Mus musculus]	1.039	0.935	0.719	1.071	0.895	1.117	0.819	1.040	0.762	0.975	0.869

568917957	XP_006500030.1#Sqrdl#59010# PREDICTED: sulfide:quinone oxidoreductase, mitochondrial isoform X2 [Mus musculus]	1.091	0.897	0.844	0.946	0.895	1.117	0.903	1.050	0.705	1.048	0.876
29243942	NP_808254.1#Eif5a2#208691# eukaryotic translation initiation factor 5A-2 [Mus musculus]	1.085	0.972	0.771	1.020	0.896	1.117	0.777	1.070	0.745	0.913	0.829
6753086	NP_033817.1#Apex1#11792# DNA-(apurinic or apyrimidinic site) lyase [Mus musculus]	1.085	0.920	0.797	0.995	0.896	1.116	0.831	1.114	0.794	0.992	0.893
226342960	NP_081455.1#Tecr#106529# very-long-chain enoyl-CoA reductase isoform 2 [Mus musculus]	0.930	0.995	0.773	1.019	0.896	1.116	0.811	0.856	0.724	0.936	0.830
21313144	NP_080218.1#Ola1#67059# obg-like ATPase 1 isoform a [Mus musculus]	0.951	0.915	0.789	1.004	0.897	1.115	0.788	0.904	0.669	0.863	0.766
197304780	NP_001127855.1#Iqsec1#232227# IQ motif and SEC7 domain-containing protein 1 isoform a [Mus musculus]	1.022	0.844	0.831	0.962	0.897	1.115	0.885	0.948	0.633	0.913	0.773
6679337	NP_032876.1#Pitpna#18738# phosphatidylinositol transfer protein alpha isoform [Mus musculus]	1.046	0.967	0.788	1.005	0.897	1.115	0.805	0.995	0.746	0.911	0.829
19527102	NP_598648.1#Stx12#100226# syntaxin-12 [Mus musculus]	0.968	0.931	0.778	1.015	0.897	1.115	0.795	0.942	0.679	0.937	0.808
568941990	XP_006506245.1#Dysf#26903# PREDICTED: dysferlin isoform X20 [Mus musculus]	1.407	0.840	0.711	1.083	0.897	1.115	1.107	1.416	0.901	1.219	1.060
166295175	NP_057872.3#Syt3#20981# synaptotagmin-3 [Mus musculus]	1.554	0.835	0.829	0.965	0.897	1.115	1.231	1.556	1.069	1.258	1.164
6755863	NP_035761.1#Hsp90b1#22027# endoplasmin precursor [Mus musculus]	1.002	0.893	0.791	1.003	0.897	1.115	0.866	1.050	0.753	0.943	0.848
568955180	XP_006509601.1#Ap1m1#11767# PREDICTED: AP-1 complex subunit mu-1 isoform X1 [Mus musculus]	1.166	0.959	0.764	1.030	0.897	1.114	0.944	1.149	0.867	1.140	1.003
258547156	NP_035182.2#Pdcd6ip#18571# programmed cell death 6-interacting protein isoform 3 [Mus musculus]	0.938	0.967	0.788	1.007	0.897	1.114	0.769	0.916	0.724	0.905	0.814
270309117	NP_001161941.1#Ube2m#22192# NEDD8-conjugating enzyme Ubc12 isoform 2 [Mus musculus]	1.004	0.964	0.763	1.033	0.898	1.114	0.781	1.022	0.706	1.033	0.869
568995055	XP_006522062.1#Tfrc#22042# PREDICTED: transferrin receptor protein 1 isoform X1 [Mus musculus]	0.960	0.823	0.711	1.085	0.898	1.114	0.877	0.861	0.618	0.814	0.716
133778983	NP_032477.2#Klc2#16594# kinesin light chain 2 [Mus musculus]	1.053	0.891	0.729	1.067	0.898	1.114	0.847	1.045	0.760	0.926	0.843
31543330	NP_032736.2#Nnt#18115# NAD(P) transhydrogenase, mitochondrial isoform 1 precursor [Mus musculus]	1.163	0.941	0.775	1.022	0.898	1.113	0.990	1.135	0.849	1.163	1.006
170763483	NP_001116292.1#Ap3m2#64933# AP-3 complex subunit mu-2 [Mus musculus]	0.945	0.901	0.814	0.983	0.898	1.113	0.787	0.912	0.680	0.851	0.765
255522931	NP_848507.2#Nlrx1#270151# NLR family member X1 [Mus musculus]	1.130	0.811	0.709	1.088	0.898	1.113	0.940	1.090	0.696	0.950	0.823
84662745	NP_032165.3#Gnaq#14682# guanine nucleotide-binding protein G(q) subunit alpha [Mus musculus]	1.029	0.949	0.764	1.033	0.898	1.113	0.765	1.061	0.808	1.004	0.906
672424492	NP_001288274.1#Hnrnpk#15387# heterogeneous nuclear ribonucleoprotein K isoform 4 [Mus musculus]	1.104	0.922	0.807	0.990	0.898	1.113	0.902	1.017	0.791	0.970	0.880
261823963	NP_115770.2#Myo7b#17922# unconventional myosin-VIIb [Mus musculus]	0.930	0.860	0.821	0.977	0.899	1.113	0.630	1.041	0.718	0.719	0.718
165377150	NP_001004357.2#Cntnap2#66797# contactin-associated protein-like 2 isoform a precursor [Mus musculus]	1.013	0.852	0.779	1.019	0.899	1.113	0.903	1.000	0.707	0.904	0.806
33563282	NP_036095.1#Psma1#26440# proteasome subunit alpha type-1 [Mus musculus]	0.855	0.847	0.765	1.033	0.899	1.112	0.739	0.838	0.587	0.801	0.694
755564945	XP_011249311.1#Ocrl#320634# PREDICTED: inositol polyphosphate 5-phosphatase OCRL-1 isoform X2 [Mus musculus]	1.375	0.876	0.757	1.042	0.899	1.112	1.052	1.421	0.907	1.258	1.083
568967951	XP_006513896.1#H2afy2#404634# PREDICTED: core histone macro-H2A.2 isoform X1 [Mus musculus]	0.874	0.914	0.801	0.997	0.899	1.112	0.745	0.826	0.608	0.828	0.718
13324686	NP_077176.1#Tomm20#67952# mitochondrial import receptor subunit TOM20 homolog [Mus musculus]	1.169	0.855	0.807	0.991	0.899	1.112	0.940	1.149	0.799	0.994	0.896
755506697	XP_011248385.1#Nudt2#66401# PREDICTED: bis(5'-nucleosyl)-tetraphosphatase [asymmetrical] isoform X1 [Mus musculus]	1.109	0.820	0.782	1.017	0.899	1.112	0.945	1.051	0.715	0.923	0.819
171184435	NP_067320.2#Hacd3#57874# very-long-chain (3R)-3-hydroxyacyl-CoA dehydratase 3 [Mus musculus]	0.932	0.909	0.783	1.016	0.900	1.112	0.759	0.933	0.680	0.898	0.789
568990347	XP_006520079.1#Cct5#12465# PREDICTED: T-complex protein 1 subunit epsilon isoform X1 [Mus musculus]	1.029	1.007	0.793	1.006	0.900	1.111	0.815	1.036	0.804	1.082	0.943
755538972	XP_011247395.1#Myo18a#360013# PREDICTED: unconventional myosin-XVIIIa isoform X25 [Mus musculus]	1.138	0.875	0.780	1.019	0.900	1.111	0.912	1.130	0.803	0.962	0.882
568990629	XP_006520215.1#Mtdh#67154# PREDICTED: protein LYRIC isoform X5 [Mus musculus]	1.724	0.867	0.796	1.003	0.900	1.111	1.451	1.652	1.144	1.546	1.345
34328471	NP_062364.3#Rala#56044# ras-related protein Ral-A precursor [Mus musculus]	0.984	1.000	0.786	1.015	0.900	1.111	0.806	0.911	0.840	0.873	0.857
261824000	NP_035314.3#Psma3#19167# proteasome subunit alpha type-3 [Mus musculus]	1.040	0.790	0.794	1.006	0.900	1.111	0.821	0.994	0.636	0.781	0.708
18699726	NP_569053.1#Vps4a#116733# vacuolar protein sorting-associated protein 4A [Mus musculus]	1.053	0.976	0.832	0.968	0.900	1.111	0.874	1.021	0.803	1.047	0.925
568908693	XP_006529469.1#Rab3gap1#226407# PREDICTED: rab3 GTPase-activating protein catalytic subunit isoform X2 [Mus musculus]	1.432	0.790	0.783	1.018	0.900	1.111	1.183	1.396	0.815	1.224	1.020
85861231	NP_001034290.1#Hdhd2#76987# haloacid dehalogenase-like hydrolase domain-containing protein 2 isoform 1 [Mus musculus]	0.917	1.011	0.814	0.987	0.901	1.110	0.738	0.914	0.689	0.981	0.835
568983230	XP_006517254.1#Exoc3#211446# PREDICTED: exocyst complex component 3 isoform X1 [Mus musculus]	0.981	0.933	0.791	1.011	0.901	1.110	0.816	0.950	0.653	0.995	0.824
31981810	NP_034153.2#Eci1#13177# enoyl-CoA delta isomerase 1, mitochondrial precursor [Mus musculus]	1.052	1.002	0.773	1.029	0.901	1.110	0.846	1.050	0.814	1.086	0.950
568912786	XP_006497671.1#Ppp2r4#110854# PREDICTED: serine/threonine-protein phosphatase 2A activator isoform X1 [Mus musculus]	1.059	0.993	0.829	0.973	0.901	1.110	0.853	1.057	0.846	1.050	0.948
27754065	NP_080714.2#Ppa1#67895# inorganic pyrophosphatase [Mus musculus]	0.893	0.850	0.820	0.983	0.901	1.109	0.765	0.845	0.617	0.752	0.684
85701644	NP_001028308.1#Rmdn3#67809# regulator of microtubule dynamics protein 3 [Mus musculus]	1.035	0.912	0.730	1.073	0.902	1.109	0.829	1.037	0.770	0.932	0.851
755553824	XP_011244272.1#Kalrn#545156# PREDICTED: kalirin isoform X26 [Mus musculus]	1.136	0.814	0.826	0.977	0.902	1.109	0.993	1.055	0.749	0.918	0.833
227330544	NP_031782.3#Cplx1#12889# complexin-1 [Mus musculus]	1.331	0.979	0.826	0.977	0.902	1.109	1.163	1.393	1.018	1.471	1.244
7549752	NP_036172.1#Cul1#26965# cullin-1 [Mus musculus]	1.073	0.902	0.816	0.987	0.902	1.109	0.879	1.055	0.796	0.950	0.873
568986840	XP_006518676.1#Kcnma1#16531# PREDICTED: calcium-activated potassium channel subunit alpha-1 isoform X16 [Mus musculus]	0.930	0.916	0.765	1.039	0.902	1.109	0.740	0.939	0.699	0.838	0.769
12963757	NP_076116.1#Isyna1#71780# inositol-3-phosphate synthase 1 [Mus musculus]	0.761	0.945	0.826	0.980	0.903	1.108	0.632	0.742	0.575	0.723	0.649
29126205	NP_803421.1#Acaa2#52538# 3-ketoacyl-CoA thiolase, mitochondrial [Mus musculus]	1.127	0.913	0.809	0.997	0.903	1.108	0.954	1.084	0.772	1.087	0.930
6753022	NP_033777.1#Ak4#11639# adenylate kinase 4, mitochondrial [Mus musculus]	0.980	1.022	0.834	0.971	0.903	1.108	0.812	0.956	0.775	1.032	0.904
482677662	NP_001264804.1#Tpm2#22004# tropomyosin beta chain isoform Tpm2.3 [Mus musculus]	1.126	0.995	0.723	1.082	0.903	1.108	0.932	1.102	0.901	1.123	1.012
171906557	NP_032838.2#Padi2#18600# protein-arginine deiminase type-2 [Mus musculus]	1.031	1.192	0.744	1.062	0.903	1.108	0.830	1.032	0.974	1.245	1.110
568907697	XP_006496561.1#Cul3#26554# PREDICTED: cullin-3 isoform X3 [Mus musculus]	1.149	0.909	0.782	1.025	0.903	1.107	0.968	1.061	0.782	1.104	0.943
568923787	XP_006502015.1#S100a16#67860# PREDICTED: protein S100-A16 isoform X1 [Mus musculus]	1.070	0.949	0.723	1.084	0.904	1.107	0.939	0.995	0.773	1.063	0.918
161484656	NP_034353.2#Fkbp8#14232# peptidyl-prolyl cis-trans isomerase FKBP8 isoform b [Mus musculus]	1.149	0.885	0.815	0.994	0.904	1.106	0.901	1.177	0.835	1.004	0.919
755496667	XP_011237254.1#Celf2#14007# PREDICTED: CUGBP Elav-like family member 2 isoform X10 [Mus musculus]	1.027	1.012	0.732	1.076	0.904	1.106	0.791	1.067	0.870	1.011	0.940
552953784	NP_001272821.1#Sae1#56459# SUMO-activating enzyme subunit 1 isoform b [Mus musculus]	0.903	0.964	0.787	1.022	0.905	1.105	0.708	0.925	0.683	0.891	0.787
569001531	XP_006524940.1#2310061I04Rik#69662# PREDICTED: uncharacterized protein C6orf136 homolog isoform X4 [Mus musculus]	1.018	0.906	0.695	1.115	0.905	1.105	0.827	1.014	0.657	1.012	0.834
12963571	NP_075691.1#Ndufa7#66416# NADH dehydrogenase [ubiquinone] 1 alpha subcomplex subunit 7 [Mus musculus]	1.118	1.003	0.731	1.078	0.905	1.105	0.898	1.151	0.686	1.088	0.887
31981945	NP_033464.2#Rpl13a#22121# 60S ribosomal protein L13a [Mus musculus]	0.824	0.916	0.856	0.953	0.905	1.105	0.679	0.877	0.667	0.866	0.766
227330633	NP_082408.3#Pgm2#72157# phosphoglucomutase-2 [Mus musculus]	1.138	0.970	0.796	1.014	0.905	1.105	0.958	1.056	0.814	1.090	0.952
569003063	XP_006525609.1#Camk4#12326# PREDICTED: calcium/calmodulin-dependent protein kinase type IV isoform X1 [Mus musculus]	1.062	0.939	0.801	1.010	0.905	1.105	0.810	1.012	0.703	0.916	0.810
31542602	NP_034615.2#Elavl1#15568# ELAV-like protein 1 [Mus musculus]	1.101	0.874	0.811	1.000	0.905	1.105	0.910	1.093	0.739	0.986	0.863
755497209	XP_011237332.1#Kcnj3#16519# PREDICTED: G protein-activated inward rectifier potassium channel 1 isoform X3 [Mus musculus]	1.137	0.769	0.763	1.048	0.905	1.104	0.980	1.078	0.686	0.897	0.792
594150372	NP_001277293.1#Aifm1#26926# apoptosis-inducing factor 1, mitochondrial isoform 2 [Mus musculus]	0.945	0.900	0.819	0.993	0.906	1.104	0.821	0.891	0.640	0.901	0.770
225543196	NP_001139431.1#Ndrg2#29811# protein NDRG2 isoform 2 [Mus musculus]	1.242	0.950	0.821	0.990	0.906	1.104	0.953	1.201	0.932	1.117	1.025
595582387	NP_001277680.1#Tmx2#66958# thioredoxin-related transmembrane protein 2 isoform b [Mus musculus]	1.080	0.928	0.780	1.031	0.906	1.104	0.922	1.033	0.792	1.023	0.907
569012136	XP_006528983.1#Acot9#56360# PREDICTED: acyl-coenzyme A thioesterase 9, mitochondrial isoform X1 [Mus musculus]	1.131	0.833	0.816	0.996	0.906	1.104	0.916	1.031	0.788	0.973	0.880
19527256	NP_598801.1#Ddx1#104721# ATP-dependent RNA helicase DDX1 [Mus musculus]	1.161	0.945	0.755	1.057	0.906	1.104	0.928	1.152	0.818	1.071	0.944
568971604	XP_006532263.1#Gabrg2#14406# PREDICTED: gamma-aminobutyric acid receptor subunit gamma-2 isoform X2 [Mus musculus]	1.110	0.803	0.790	1.022	0.906	1.104	0.960	1.051	0.720	0.894	0.807
113195686	NP_034852.2#Lmnb2#16907# lamin-B2 [Mus musculus]	1.089	0.838	0.779	1.033	0.906	1.104	0.913	1.060	0.699	0.954	0.826
755496932	XP_011237298.1#Gm13194#381346# PREDICTED: 60S ribosomal protein L36a-like [Mus musculus]	0.991	0.863	0.821	0.993	0.907	1.103	0.845	0.952	0.712	0.839	0.775
30519925	NP_848720.1#Nop16#28126# nucleolar protein 16 [Mus musculus]	1.281	0.858	0.794	1.020	0.907	1.103	1.082	1.242	0.883	1.112	0.997
13507666	NP_109642.1#Lactb#80907# serine beta-lactamase-like protein LACTB, mitochondrial precursor [Mus musculus]	1.469	0.875	0.861	0.953	0.907	1.103	1.245	1.418	0.918	1.411	1.164
165	CYB5_HUMAN	1.019	0.949	0.826	0.987	0.907	1.103	0.846	1.002	0.739	1.015	0.877
28316760	NP_783595.1#Hist1h2bb#319178# histone H2B type 1-B [Mus musculus]	1.124	1.058	0.757	1.058	0.907	1.102	0.859	1.180	0.939	1.218	1.079
100817933	NP_766266.3#Acad9#229211# acyl-CoA dehydrogenase family member 9, mitochondrial [Mus musculus]	1.144	0.882	0.805	1.010	0.908	1.101	0.915	1.199	0.918	1.120	1.019
6755364	NP_035424.1#Sub1#20024# activated RNA polymerase II transcriptional coactivator p15 [Mus musculus]	0.923	0.893	0.756	1.061	0.908	1.101	0.750	0.927	0.699	0.798	0.749
6678449	NP_033463.1#Tst#22117# thiosulfate sulfurtransferase [Mus musculus]	1.024	0.870	0.824	0.993	0.908	1.101	0.862	0.998	0.714	0.904	0.809
227499988	NP_001153105.1#Slc44a1#100434# choline transporter-like protein 1 isoform B [Mus musculus]	1.000	1.094	0.859	0.958	0.909	1.101	0.792	0.991	0.970	1.100	1.035
158186704	NP_001103383.1#Hnrnpm#76936# heterogeneous nuclear ribonucleoprotein M isoform b [Mus musculus]	1.094	0.911	0.843	0.976	0.909	1.100	0.898	1.047	0.778	1.047	0.913
568927577	XP_006538412.1#Slc24a2#76376# PREDICTED: sodium/potassium/calcium exchanger 2 isoform X7 [Mus musculus]	0.753	0.868	0.872	0.946	0.909	1.100	0.618	0.750	0.501	0.685	0.593
568911073	XP_006497013.1#Aldh9a1#56752# PREDICTED: 4-trimethylaminobutyraldehyde dehydrogenase isoform X1 [Mus musculus]	1.139	0.966	0.835	0.983	0.909	1.100	0.987	1.084	0.883	1.114	0.999
160333923	NP_058085.2#Hnrnpu#51810# heterogeneous nuclear ribonucleoprotein U [Mus musculus]	1.126	0.905	0.815	1.004	0.909	1.100	0.890	1.132	0.777	1.049	0.913

70909327	NP_034069.2#Cops2#12848# COP9 signalosome complex subunit 2 isoform b [Mus musculus]	1.003	0.940	0.748	1.071	0.910	1.099	0.863	1.054	0.673	0.997	0.835
568938961	XP_006504870.1#Hmgb1#15289# PREDICTED: high mobility group protein B1 isoform X1 [Mus musculus]	1.049	0.951	0.820	1.001	0.910	1.099	0.911	1.080	0.767	0.961	0.864
755558845	XP_011244878.1#Slc25a23#66972# PREDICTED: calcium-binding mitochondrial carrier protein SCaMC-3 isoform X2 [Mus musculus]	0.804	0.923	0.810	1.011	0.911	1.098	0.631	0.834	0.598	0.753	0.676
225735655	NP_001139595.1#Psap#19156# prosaposin isoform E preproprotein [Mus musculus]	1.777	0.930	0.777	1.044	0.911	1.098	1.434	1.628	1.224	1.630	1.427
6755202	NP_036101.1#Psmb3#26446# proteasome subunit beta type-3 [Mus musculus]	1.006	0.856	0.815	1.007	0.911	1.098	0.731	1.033	0.648	0.921	0.784
170650632	NP_001116237.1#Sirt2#64383# NAD-dependent protein deacetylase sirtuin-2 isoform 2 [Mus musculus]	1.009	1.035	0.838	0.985	0.911	1.097	0.826	1.016	0.840	1.072	0.956
568946281	XP_006540290.1#Clptm1#56457# PREDICTED: cleft lip and palate transmembrane protein 1 homolog isoform X1 [Mus musculus]	1.229	0.873	0.800	1.022	0.911	1.097	1.029	1.210	0.792	1.162	0.977
126032329	NP_034236.2#Eef1a1#13627# elongation factor 1-alpha 1 [Mus musculus]	1.236	0.859	0.863	0.960	0.912	1.097	0.912	1.258	0.792	0.992	0.892
568960685	XP_006510846.1#Atp1b3#11933# PREDICTED: sodium/potassium-transporting ATPase subunit beta-3 isoform X1 [Mus musculus]	1.016	1.011	0.772	1.052	0.912	1.097	0.927	1.083	0.828	1.155	0.991
13569841	NP_056577.2#Txnrd1#50493# thioredoxin reductase 1, cytoplasmic isoform 2 [Mus musculus]	1.015	0.877	0.772	1.052	0.912	1.097	0.870	0.949	0.657	0.899	0.778
281485615	NP_035017.2#Ndufs4#17993# NADH dehydrogenase [ubiquinone] iron-sulfur protein 4, mitochondrial [Mus musculus]	0.978	0.995	0.839	0.985	0.912	1.096	0.812	1.011	0.758	1.042	0.900
568975194	XP_006533978.1#Fn3k#63828# PREDICTED: fructosamine-3-kinase isoform X2 [Mus musculus]	0.904	0.998	0.790	1.034	0.912	1.096	0.735	0.913	0.700	0.945	0.823
6680894	NP_031683.1#Cd9#12527# CD9 antigen [Mus musculus]	1.022	1.214	0.814	1.010	0.912	1.096	0.866	1.000	0.938	1.328	1.133
40807498	NP_032313.2#Hrsp12#15473# ribonuclease UK114 [Mus musculus]	1.051	0.992	0.819	1.005	0.912	1.096	0.837	1.028	0.784	1.037	0.911
242397487	NP_001156318.1#Phyhipl#70911# phytanoyl-CoA hydroxylase-interacting protein-like isoform 2 [Mus musculus]	1.199	0.988	0.815	1.009	0.912	1.096	0.975	1.159	0.895	1.224	1.060
594591591	NP_001277563.1#Ugp2#216558# UTP--glucose-1-phosphate uridylyltransferase isoform 2 [Mus musculus]	1.036	0.976	0.848	0.977	0.912	1.096	0.861	1.008	0.843	1.021	0.932
205361114	NP_001128584.1#Dnaja3#83945# dnaJ homolog subfamily A member 3, mitochondrial isoform 2 [Mus musculus]	1.035	0.941	0.765	1.060	0.913	1.096	0.870	1.018	0.797	0.977	0.887
112181167	NP_031599.2#C1qbp#12261# complement component 1 Q subcomponent-binding protein, mitochondrial [Mus musculus]	1.134	0.909	0.828	0.998	0.913	1.096	0.899	1.148	0.782	1.033	0.907
33859801	NP_789818.1#Fbxo2#230904# F-box only protein 2 [Mus musculus]	1.114	0.951	0.768	1.058	0.913	1.095	0.909	1.168	0.813	1.124	0.968
113680352	NP_031646.2#Cbr1#12408# carbonyl reductase [NADPH] 1 [Mus musculus]	1.055	0.912	0.793	1.033	0.913	1.095	0.862	1.042	0.730	0.963	0.846
568921903	XP_006501115.1#Kcna2#16490# PREDICTED: potassium voltage-gated channel subfamily A member 2 isoform X1 [Mus musculus]	1.124	0.992	0.860	0.967	0.913	1.095	0.924	1.129	0.911	1.126	1.018
568954159	XP_006509214.1#Hook3#320191# PREDICTED: protein Hook homolog 3 isoform X4 [Mus musculus]	1.210	0.835	0.850	0.976	0.913	1.095	1.052	1.160	0.879	0.955	0.917
7657011	NP_056550.1#Ddb1#13194# DNA damage-binding protein 1 [Mus musculus]	1.010	0.857	0.812	1.015	0.913	1.095	0.873	0.971	0.677	0.904	0.790
31981458	NP_444338.2#Glrx#93692# glutaredoxin-1 [Mus musculus]	1.114	0.956	0.891	0.935	0.913	1.095	0.978	1.067	0.855	1.067	0.961
821324946	NP_001295574.1#Aqp4#11829# aquaporin-4 isoform M23A [Mus musculus]	1.050	1.187	0.807	1.020	0.914	1.095	0.989	0.930	1.037	1.241	1.139
568972828	XP_006532849.1#Skp1a#21402# PREDICTED: S-phase kinase-associated protein 1 isoform X1 [Mus musculus]	1.067	0.951	0.782	1.047	0.915	1.093	0.884	1.075	0.851	1.086	0.968
13384700	NP_079610.1#Txndc12#66073# thioredoxin domain-containing protein 12 precursor [Mus musculus]	0.989	1.052	0.827	1.004	0.915	1.093	0.751	1.059	0.836	1.068	0.952
6679120	NP_032756.1#Nptx1#18164# neuronal pentraxin-1 precursor [Mus musculus]	1.214	0.815	0.786	1.044	0.915	1.093	0.978	1.244	0.710	1.100	0.905
13385392	NP_080174.1#Napa#108124# alpha-soluble NSF attachment protein [Mus musculus]	1.183	0.814	0.818	1.013	0.916	1.092	0.931	1.136	0.759	0.941	0.850
755559149	XP_011244962.1#Slc25a27#74011# PREDICTED: mitochondrial uncoupling protein 4 isoform X5 [Mus musculus]	0.896	0.987	0.882	0.949	0.916	1.092	0.769	0.872	0.704	0.916	0.810
69885049	NP_001020426.1#Mbp#17196# myelin basic protein isoform 3 [Mus musculus]	0.840	1.395	0.812	1.020	0.916	1.092	0.738	0.831	0.961	1.296	1.128
568918221	XP_006500154.1#Fahd2a#68126# PREDICTED: fumarylacetoacetate hydrolase domain-containing protein 2A isoform X2 [Mus musculus]	1.279	0.922	0.784	1.048	0.916	1.092	1.057	1.146	0.965	1.139	1.052
568915283	XP_006498743.1#Dync1i2#13427# PREDICTED: cytoplasmic dynein 1 intermediate chain 2 isoform X6 [Mus musculus]	1.013	0.982	0.832	1.001	0.917	1.091	0.865	0.992	0.809	1.015	0.912
254675279	NP_001157046.1#Tjp1#21872# tight junction protein ZO-1 isoform 2 [Mus musculus]	0.930	0.923	0.813	1.020	0.917	1.091	0.795	0.910	0.688	0.887	0.787
568907510	XP_006496471.1#Epha4#13838# PREDICTED: ephrin type-A receptor 4 isoform X2 [Mus musculus]	1.038	0.835	0.808	1.026	0.917	1.091	0.876	1.059	0.638	0.894	0.766
227499240	NP_001153098.1#Hagh#14651# hydroxyacylglutathione hydrolase, mitochondrial isoform 2 [Mus musculus]	0.994	0.899	0.740	1.094	0.917	1.090	0.796	1.034	0.733	0.913	0.823
600971669	NP_001278115.1#Abr#109934# active breakpoint cluster region-related protein isoform 4 [Mus musculus]	1.150	0.854	0.806	1.029	0.918	1.090	1.055	1.173	0.916	1.133	1.024
6754090	NP_034492.1#Gsto1#14873# glutathione S-transferase omega-1 [Mus musculus]	1.044	0.938	0.804	1.031	0.918	1.090	0.829	1.086	0.795	1.001	0.898
153791468	NP_082716.1#Abracl#73112# costars family protein ABRACL [Mus musculus]	1.224	0.865	0.903	0.932	0.918	1.090	1.061	1.185	0.844	1.097	0.971
226823266	NP_766407.2#Dip2b#239667# disco-interacting protein 2 homolog B isoform 2 [Mus musculus]	1.130	0.985	0.829	1.007	0.918	1.090	1.010	1.065	0.896	1.148	1.022
22203763	NP_038522.2#Cpe#12876# carboxypeptidase E precursor [Mus musculus]	1.040	0.891	0.825	1.011	0.918	1.089	0.890	1.004	0.767	0.934	0.850
21553309	NP_659146.1#Apoa1bp#246703# NAD(P)H-hydrate epimerase precursor [Mus musculus]	1.008	0.921	0.814	1.023	0.918	1.089	0.886	0.965	0.784	0.921	0.852
58037481	NP_084101.1#Scfd1#76983# sec1 family domain-containing protein 1 isoform 1 [Mus musculus]	1.052	1.198	0.816	1.021	0.918	1.089	0.917	1.015	0.881	1.435	1.158
238814391	NP_031664.3#Cct7#12468# T-complex protein 1 subunit eta [Mus musculus]	0.988	0.912	0.823	1.014	0.919	1.089	0.762	0.846	0.682	0.893	0.788
569003863	XP_006525998.1#Slc6a7#240332# PREDICTED: sodium-dependent proline transporter isoform X5 [Mus musculus]	0.917	0.905	0.824	1.013	0.919	1.088	0.750	0.935	0.666	0.860	0.763
568916096	XP_006499132.1#Ssb#20823# PREDICTED: lupus La protein homolog isoform X1 [Mus musculus]	1.002	0.842	0.817	1.020	0.919	1.088	0.879	1.070	0.722	0.919	0.821
247269309	NP_034038.2#Cmas#12764# N-acylneuraminate cytidylyltransferase [Mus musculus]	0.987	0.896	0.842	0.996	0.919	1.088	0.814	1.000	0.711	0.913	0.812
262050556	NP_080402.3#Fundc2#67391# FUN14 domain-containing protein 2 [Mus musculus]	1.240	0.853	0.805	1.034	0.919	1.088	1.066	1.214	0.792	1.050	0.921
568963230	XP_006511925.1#Nckipsd#80987# PREDICTED: NCK-interacting protein with SH3 domain isoform X3 [Mus musculus]	1.147	0.828	0.791	1.048	0.920	1.087	0.953	1.157	0.766	0.982	0.874
568941109	XP_006505819.1#Luc7l2#192196# PREDICTED: putative RNA-binding protein Luc7-like 2 isoform X8 [Mus musculus]	1.048	0.938	0.796	1.044	0.920	1.087	0.852	1.028	0.811	0.955	0.883
227116275	NP_001153037.1#Dctn3#53598# dynactin subunit 3 isoform B [Mus musculus]	1.051	0.887	0.782	1.058	0.920	1.087	0.860	1.073	0.763	0.952	0.857
11528516	NP_071312.1#Nrgn#64011# neurogranin [Mus musculus]	0.934	0.803	0.826	1.014	0.920	1.087	0.766	0.955	0.581	0.802	0.691
568890009	XP_006543837.1#Gm6570#625281# PREDICTED: 60S ribosomal protein L30-like [Mus musculus]	0.981	0.862	0.868	0.974	0.921	1.086	0.770	1.036	0.648	0.909	0.778
755548669	XP_011243434.1#Xpo7#65246# PREDICTED: exportin-7 isoform X4 [Mus musculus]	1.146	0.940	0.890	0.952	0.921	1.085	1.023	1.085	0.961	1.024	0.993
568908524	XP_006529387.1#Syt2#20980# PREDICTED: synaptotagmin-2 isoform X2 [Mus musculus]	1.218	1.007	0.824	1.019	0.922	1.085	0.955	1.290	0.964	1.297	1.130
568947586	XP_006540813.1#Abcc8#20927# PREDICTED: ATP-binding cassette sub-family C member 8 isoform X9 [Mus musculus]	1.261	0.936	0.883	0.960	0.922	1.085	1.132	1.192	0.926	1.249	1.087
755551244	XP_011243826.1#Rangap1#19387# PREDICTED: ran GTPase-activating protein 1 isoform X2 [Mus musculus]	1.146	0.859	0.770	1.076	0.923	1.084	0.972	1.172	0.755	1.107	0.931
8394493	NP_059075.1#Tuba8#53857# tubulin alpha-8 chain [Mus musculus]	1.209	0.974	0.829	1.017	0.923	1.084	1.135	1.098	0.966	1.209	1.087
6755408	NP_035452.1#Scn1b#20266# sodium channel subunit beta-1 precursor [Mus musculus]	1.006	0.913	0.832	1.016	0.924	1.082	0.848	1.012	0.780	0.917	0.849
148747331	NP_084481.3#Coro7#78885# coronin-7 [Mus musculus]	1.023	0.894	0.837	1.012	0.924	1.082	0.835	1.056	0.710	0.981	0.846
357197139	NP_001239398.1#Bag6#224727# large proline-rich protein BAG6 isoform 3 [Mus musculus]	1.067	0.878	0.823	1.025	0.924	1.082	0.990	1.065	0.775	0.951	0.863
33859604	NP_035318.1#Psmc2#19181# 26S protease regulatory subunit 7 [Mus musculus]	1.076	0.860	0.791	1.058	0.925	1.081	0.868	1.122	0.746	0.966	0.856
122937361	NP_032162.2#Gnl1#14670# guanine nucleotide-binding protein-like 1 [Mus musculus]	1.012	0.990	0.865	0.984	0.925	1.081	0.886	0.986	0.760	1.094	0.927
568947550	XP_006540796.1#Snrnp70#20637# PREDICTED: U1 small nuclear ribonucleoprotein 70 kDa isoform X2 [Mus musculus]	1.109	0.902	0.792	1.059	0.925	1.081	0.921	1.131	0.814	1.037	0.926
160707896	NP_035629.2#Strap#20901# serine-threonine kinase receptor-associated protein [Mus musculus]	1.095	0.907	0.861	0.991	0.926	1.080	0.988	1.040	0.794	1.046	0.920
568924859	XP_006502532.1#Sec24b#99683# PREDICTED: protein transport protein Sec24B isoform X3 [Mus musculus]	1.175	0.931	0.966	0.886	0.926	1.080	1.001	1.177	0.823	1.205	1.014
124487263	NP_001074610.1#Gabbr2#242425# gamma-aminobutyric acid type B receptor subunit 2 precursor [Mus musculus]	1.362	0.796	0.836	1.017	0.927	1.079	1.179	1.355	0.879	1.136	1.007
568903083	XP_006543485.1#LOC102642058#102642058# PREDICTED: uncharacterized protein LOC102642058 isoform X1 [Mus musculus]	0.934	0.875	0.835	1.019	0.927	1.079	0.857	0.874	0.692	0.822	0.757
755507949	XP_011238779.1#Scp2#20280# PREDICTED: non-specific lipid-transfer protein isoform X1 [Mus musculus]	1.323	1.010	0.838	1.016	0.927	1.079	1.148	1.305	1.166	1.312	1.239
568950272	XP_006507720.1#Ipo7#233726# PREDICTED: importin-7 isoform X1 [Mus musculus]	0.991	0.903	0.828	1.027	0.927	1.078	0.839	1.053	0.726	0.885	0.805
21536256	NP_659077.1#Ppp1r1b#19049# protein phosphatase 1 regulatory subunit 1B [Mus musculus]	0.999	1.140	0.890	0.964	0.927	1.078	0.883	0.944	0.949	1.190	1.070
6753242	NP_033918.1#Calb1#12307# calbindin [Mus musculus]	0.918	0.774	0.825	1.029	0.927	1.078	0.774	0.944	0.586	0.834	0.710
19527388	NP_598911.1#Otub1#107260# ubiquitin thioesterase OTUB1 [Mus musculus]	0.875	0.914	0.834	1.021	0.927	1.078	0.699	0.847	0.644	0.804	0.724
7106331	NP_034566.1#H2afx#15270# histone H2AX [Mus musculus]	0.820	0.938	0.860	0.995	0.928	1.078	0.557	0.708	0.477	0.785	0.631
19527154	NP_598698.1#Brk1#101314# protein BRICK1 [Mus musculus]	**2.099**	0.806	0.882	0.974	0.928	1.078	1.499	2.251	1.351	1.846	1.598
568954511	XP_006509328.1#Acsl1#14081# PREDICTED: long-chain-fatty-acid--CoA ligase 1 isoform X1 [Mus musculus]	1.133	0.873	0.836	1.021	0.928	1.077	0.922	1.182	0.771	1.065	0.918
6996917	NP_032088.1#G6pdx#14381# glucose-6-phosphate 1-dehydrogenase X [Mus musculus]	1.094	1.035	0.881	0.976	0.928	1.077	0.956	1.075	0.943	1.161	1.052
568999488	XP_006523949.1#Ptprs#19280# PREDICTED: receptor-type tyrosine-protein phosphatase S isoform X10 [Mus musculus]	1.129	0.897	0.807	1.050	0.929	1.077	0.949	1.149	0.831	1.050	0.941
120407050	NP_061356.2#Nop58#55989# nucleolar protein 58 [Mus musculus]	1.030	0.799	0.813	1.045	0.929	1.077	0.933	0.979	0.796	0.731	0.764
755537039	XP_011246966.1#Rars#104458# PREDICTED: arginine--tRNA ligase, cytoplasmic isoform X1 [Mus musculus]	0.981	0.894	0.812	1.046	0.929	1.077	0.785	1.037	0.679	0.949	0.814
31982664	NP_080198.2#Itpa#16434# inosine triphosphate pyrophosphatase [Mus musculus]	0.692	0.890	0.781	1.077	0.929	1.076	0.588	0.699	0.489	0.656	0.573
6755256	NP_035354.1#Pygm#19309# glycogen phosphorylase, muscle form [Mus musculus]	1.133	0.946	0.810	1.049	0.929	1.076	1.008	1.237	0.879	1.111	0.995
568915490	XP_006498839.1#Gnas#14683# PREDICTED: protein GNAS isoform X5 [Mus musculus]	1.186	0.917	0.799	1.060	0.930	1.076	0.994	1.134	0.842	1.093	0.968

568939016	XP_006504897.1#Wasf3#245880# PREDICTED: wiskott-Aldrich syndrome protein family member 3 isoform X2 [Mus musculus]	1.088	0.857	0.782	1.077	0.930	1.076	0.914	1.108	0.752	0.981	0.866
225703070	NP_082724.1#Cenpv#73139# centromere protein V [Mus musculus]	0.883	0.909	0.851	1.009	0.930	1.075	0.793	0.846	0.654	0.839	0.747
6754084	NP_034488.1#Gstm1#14862# glutathione S-transferase Mu 1 [Mus musculus]	1.016	0.984	0.824	1.036	0.930	1.075	0.848	0.994	0.808	1.119	0.964
568915725	XP_006498949.1#Myef2#17876# PREDICTED: myelin expression factor 2 isoform X7 [Mus musculus]	0.949	0.966	0.831	1.030	0.930	1.075	0.764	1.001	0.792	0.913	0.853
13385872	NP_080650.1#Ilf2#67781# interleukin enhancer-binding factor 2 [Mus musculus]	0.913	0.931	0.779	1.082	0.931	1.075	0.781	0.921	0.693	0.892	0.792
21703972	NP_663469.1#Me2#107029# NAD-dependent malic enzyme, mitochondrial precursor [Mus musculus]	1.066	0.929	0.778	1.083	0.931	1.074	0.909	1.080	0.774	1.073	0.923
357197145	NP_001239400.1#Sh3gl1#20405# endophilin-A2 isoform 2 [Mus musculus]	1.036	0.834	0.782	1.080	0.931	1.074	0.875	1.053	0.700	0.907	0.804
283945577	NP_001164424.1#Rnmt#67897# mRNA cap guanine-N7 methyltransferase isoform 2 [Mus musculus]	1.068	0.826	0.845	1.017	0.931	1.074	0.919	1.069	0.716	0.925	0.821
345842402	NP_001171101.2#Etl4#208618# sickle tail protein isoform d [Mus musculus]	0.951	0.805	0.739	1.125	0.932	1.073	0.772	0.999	0.629	0.798	0.713
13399310	NP_080239.1#Rps10#67097# 40S ribosomal protein S10 [Mus musculus]	0.842	0.838	0.813	1.051	0.932	1.073	0.710	0.861	0.582	0.733	0.658
568978528	XP_006515398.1#Ttc7b#104718# PREDICTED: tetratricopeptide repeat protein 7B isoform X13 [Mus musculus]	1.067	0.910	0.872	0.993	0.932	1.073	0.968	1.021	0.754	1.056	0.905
548923858	NP_001271327.1#Sept4#18952# septin-4 isoform 4 [Mus musculus]	1.005	1.049	0.869	0.996	0.932	1.073	0.876	1.130	0.857	1.100	0.979
356995868	NP_001239386.1#Ddx39b#53817# spliceosome RNA helicase Ddx39b [Mus musculus]	1.139	0.896	0.857	1.008	0.932	1.072	0.997	1.126	0.885	1.017	0.951
298231238	NP_001177187.1#Rps27rt#100043813# ribosomal protein S27-like [Mus musculus]	1.045	0.935	0.820	1.045	0.933	1.072	0.843	1.106	0.818	1.004	0.911
124248575	NP_001074265.1#G3bp2#23881# ras GTPase-activating protein-binding protein 2 isoform b [Mus musculus]	1.155	0.858	0.840	1.029	0.934	1.070	0.939	1.218	0.776	1.074	0.925
568928890	XP_006503056.1#Nrd1#230598# PREDICTED: nardilysin isoform X4 [Mus musculus]	1.041	0.926	0.922	0.947	0.934	1.070	0.918	1.027	0.848	0.953	0.901
568958970	XP_006510134.1#Ncam1#17967# PREDICTED: neural cell adhesion molecule 1 isoform X17 [Mus musculus]	0.954	0.808	0.816	1.053	0.935	1.070	0.822	0.959	0.674	0.766	0.720
125490380	NP_033534.2#Slc32a1#22348# vesicular inhibitory amino acid transporter [Mus musculus]	0.818	1.055	0.818	1.052	0.935	1.070	0.735	0.839	0.675	0.911	0.793
244789999	NP_619611.3#Mpst#246221# 3-mercaptopyruvate sulfurtransferase [Mus musculus]	1.015	0.913	0.785	1.087	0.936	1.068	0.857	1.035	0.731	0.944	0.838
15426055	NP_203534.1#Copb1#70349# coatomer subunit beta [Mus musculus]	0.969	0.842	0.886	0.986	0.936	1.068	0.860	0.954	0.650	0.877	0.764
83921618	NP_033536.2#Ezr#22350# ezrin [Mus musculus]	1.125	0.922	0.817	1.056	0.937	1.068	1.037	1.008	0.767	1.152	0.959
160333837	NP_061200.2#Rpl36#54217# 60S ribosomal protein L36 [Mus musculus]	1.033	0.803	0.762	1.111	0.937	1.068	0.883	1.051	0.671	0.883	0.777
22094989	NP_079892.1#Timm50#66525# mitochondrial import inner membrane translocase subunit TIM50 precursor [Mus musculus]	1.158	0.841	0.790	1.084	0.937	1.067	1.073	1.096	0.788	1.036	0.912
45433590	NP_062292.1#Arl6ip1#54208# ADP-ribosylation factor-like protein 6-interacting protein 1 [Mus musculus]	0.987	0.878	0.782	1.092	0.937	1.067	0.853	0.997	0.749	0.875	0.812
334688858	NP_001229310.1#Slc4a10#94229# sodium-driven chloride bicarbonate exchanger isoform 5 [Mus musculus]	1.271	0.786	0.826	1.048	0.937	1.067	1.013	1.283	0.798	0.979	0.889
568915467	XP_006498830.1#Gad1#14415# PREDICTED: glutamate decarboxylase 1 isoform X2 [Mus musculus]	1.035	0.979	0.880	0.995	0.938	1.066	0.879	1.060	0.820	1.067	0.943
261878591	NP_038912.3#Cspg5#29873# chondroitin sulfate proteoglycan 5 isoform a precursor [Mus musculus]	1.094	0.931	0.880	0.995	0.938	1.066	0.984	1.069	0.822	1.088	0.955
67906177	NP_033173.2#Sec23a#20334# protein transport protein Sec23A [Mus musculus]	1.085	0.960	0.910	0.966	0.938	1.066	0.930	1.107	0.772	1.183	0.977
568971480	XP_006532202.1#Mpp3#13384# PREDICTED: MAGUK p55 subfamily member 3 isoform X2 [Mus musculus]	1.074	0.832	0.804	1.075	0.939	1.065	0.955	1.108	0.758	0.958	0.858
356640163	NP_001239245.1#Shmt2#108037# serine hydroxymethyltransferase, mitochondrial isoform 2 [Mus musculus]	1.031	0.905	0.881	0.998	0.940	1.064	0.884	1.053	0.736	1.018	0.877
254540212	NP_001156925.1#Mapk8ip3#30957# C-Jun-amino-terminal kinase-interacting protein 3 isoform g [Mus musculus]	1.011	0.933	0.851	1.030	0.940	1.064	0.844	1.056	0.759	1.014	0.887
568995944	XP_006522489.1#Gsk3b#56637# PREDICTED: glycogen synthase kinase-3 beta isoform X2 [Mus musculus]	1.098	0.765	0.925	0.957	0.941	1.063	0.856	1.210	0.654	0.926	0.790
568981493	XP_006516581.1#Epdr1#105298# PREDICTED: mammalian ependymin-related protein 1 isoform X1 [Mus musculus]	1.100	0.858	0.859	1.023	0.941	1.062	0.885	1.186	0.801	0.975	0.888
569020729	XP_006544960.1#Coro1b#23789# PREDICTED: coronin-1B isoform X1 [Mus musculus]	0.966	0.937	0.822	1.061	0.942	1.062	0.816	1.003	0.735	0.970	0.852
260166719	NP_083713.2#Ckap5#75786# cytoskeleton-associated protein 5 isoform 2 [Mus musculus]	1.133	0.933	0.862	1.023	0.943	1.061	0.993	1.137	0.976	1.243	1.109
78126155	NP_031622.2#Caml#12328# calcium signal-modulating cyclophilin ligand [Mus musculus]	0.953	0.876	0.938	0.948	0.943	1.060	0.827	0.970	0.660	0.913	0.787
6679078	NP_032731.1#Nme2#18103# nucleoside diphosphate kinase B [Mus musculus]	0.921	1.036	0.865	1.023	0.944	1.060	0.814	0.924	0.769	1.032	0.900
568994806	XP_006521942.1#Tra2b#20462# PREDICTED: transformer-2 protein homolog beta isoform X2 [Mus musculus]	0.891	0.843	0.815	1.072	0.944	1.059	0.783	0.898	0.634	0.784	0.709
6753140	NP_033853.1#Atp2b2#11941# plasma membrane calcium-transporting ATPase 2 [Mus musculus]	1.364	0.836	0.865	1.024	0.944	1.059	1.137	1.440	0.970	1.184	1.077
755537693	XP_011247108.1#Cyth1#19157# PREDICTED: cytohesin-1 isoform X5 [Mus musculus]	0.988	0.946	1.006	0.885	0.945	1.058	0.982	0.885	0.642	1.124	0.883
568924485	XP_006502350.1#Agl#77559# PREDICTED: glycogen debranching enzyme isoform X1 [Mus musculus]	1.169	0.844	0.851	1.042	0.947	1.056	1.004	1.208	0.802	1.065	0.934
226823309	NP_001152847.1#Eif4a1#13681# eukaryotic initiation factor 4A-I isoform 2 [Mus musculus]	1.168	0.855	0.860	1.034	0.947	1.056	0.971	1.239	0.793	1.096	0.945
170763467	NP_035722.2#Timm44#21856# mitochondrial import inner membrane translocase subunit TIM44 [Mus musculus]	1.150	0.925	0.850	1.044	0.947	1.056	0.959	1.082	0.877	1.089	0.983
568934164	XP_006503989.1#Ablim2#231148# PREDICTED: actin-binding LIM protein 2 isoform X21 [Mus musculus]	1.029	0.888	0.864	1.030	0.947	1.056	0.870	1.076	0.719	1.011	0.865
155	ANXA5_HUMAN	1.096	0.965	0.828	1.068	0.948	1.055	0.904	1.066	0.951	1.099	1.025
568961845	XP_006511406.1#Commd4#66199# PREDICTED: COMM domain-containing protein 4 isoform X1 [Mus musculus]	0.949	0.841	0.901	0.996	0.948	1.055	0.822	0.977	0.689	0.824	0.756
153792526	NP_080135.2#Arl1#104303# ADP-ribosylation factor-like protein 1 [Mus musculus]	0.736	0.964	0.814	1.083	0.949	1.054	0.657	0.739	0.526	0.820	0.673
7242138	NP_038201.1#Atp1b2#11932# sodium/potassium-transporting ATPase subunit beta-2 [Mus musculus]	1.058	0.919	0.887	1.010	0.949	1.054	0.922	1.169	0.889	1.186	1.038
568941196	XP_006505862.1#Mical3#194401# PREDICTED: protein-methionine sulfoxide oxidase MICAL3 isoform X1 [Mus musculus]	1.033	0.821	0.961	0.937	0.949	1.054	0.969	0.991	0.758	0.852	0.805
755492347	XP_011236787.1#Actr1b#226977# PREDICTED: beta-centractin isoform X2 [Mus musculus]	1.092	0.945	0.806	1.093	0.949	1.053	0.899	1.175	0.830	1.130	0.980
557878608	NP_001273658.1#Glcci1#170772# glucocorticoid-induced transcript 1 protein isoform 3 [Mus musculus]	0.897	0.823	0.996	0.904	0.950	1.053	0.923	0.780	0.669	0.733	0.701
226246660	NP_001139696.1#Dpy30#66310# protein dpy-30 homolog [Mus musculus]	0.990	1.071	0.828	1.073	0.951	1.052	0.856	1.026	0.850	1.161	1.006
568967304	XP_006513588.1#Eea1#216238# PREDICTED: early endosome antigen 1 isoform X4 [Mus musculus]	1.067	0.996	0.781	1.121	0.951	1.052	0.978	1.052	0.851	1.171	1.011
807066384	NP_001292910.1#Diap1#13367# protein diaphanous homolog 1 isoform 3 [Mus musculus]	0.953	0.941	0.749	1.156	0.952	1.050	0.832	0.986	0.731	0.981	0.856
568908913	XP_006529574.1#2310035C23Rik#227446# PREDICTED: lisH domain and HEAT repeat-containing protein KIAA1468 isoform X11 [Mus musculus]	1.096	0.782	0.831	1.075	0.953	1.049	0.962	1.081	0.683	0.952	0.817
188219589	NP_034851.2#Lmnb1#16906# lamin-B1 [Mus musculus]	1.193	0.898	0.805	1.103	0.954	1.049	0.969	1.307	0.862	1.183	1.022
164698448	NP_598819.2#Pelo#105083# protein pelota homolog [Mus musculus]	1.443	1.086	0.821	1.087	0.954	1.048	1.272	1.482	1.247	1.742	1.495
51491852	NP_001003913.1#Mars#216443# methionine--tRNA ligase, cytoplasmic isoform 2 [Mus musculus]	1.115	0.993	0.887	1.022	0.954	1.048	0.946	1.183	0.886	1.227	1.056
158187517	NP_001094941.1#Akap5#238276# A-kinase anchor protein 5 [Mus musculus]	0.932	1.012	0.904	1.005	0.955	1.048	0.801	0.978	0.766	1.034	0.900
21314826	NP_080886.1#Ndufb4#68194# NADH dehydrogenase [ubiquinone] 1 beta subcomplex subunit 4 [Mus musculus]	0.836	0.934	0.781	1.129	0.955	1.047	0.724	0.873	0.616	0.876	0.746
133922578	NP_613069.3#Ccdc22#54638# coiled-coil domain-containing protein 22 [Mus musculus]	1.095	0.934	0.791	1.121	0.956	1.046	0.933	1.162	0.870	1.085	0.978
188035926	NP_001120818.1#Ndufaf2#75597# mimitin, mitochondrial [Mus musculus]	0.930	0.904	0.747	1.166	0.957	1.045	0.836	0.944	0.713	0.897	0.805
6755376	NP_035430.1#Rps7#20115# 40S ribosomal protein S7 [Mus musculus]	0.880	0.879	0.867	1.048	0.957	1.045	0.690	0.922	0.574	0.737	0.655
6755204	NP_035316.1#Psmb5#19173# proteasome subunit beta type-5 [Mus musculus]	0.970	0.918	0.893	1.024	0.959	1.043	0.808	1.052	0.773	0.935	0.854
84872187	NP_001034167.1#Prosc#114863# proline synthase co-transcribed bacterial homolog protein isoform c [Mus musculus]	1.013	1.026	0.882	1.035	0.959	1.043	0.889	1.054	0.867	1.127	0.997
568954733	XP_006509438.1#Sorbs2#234214# PREDICTED: sorbin and SH3 domain-containing protein 2 isoform X27 [Mus musculus]	0.792	0.836	0.823	1.097	0.960	1.042	0.711	0.809	0.568	0.703	0.635
568928764	XP_006502995.1#Urod#22275# PREDICTED: uroporphyrinogen decarboxylase isoform X2 [Mus musculus]	1.160	0.872	0.846	1.074	0.960	1.041	1.088	1.139	0.844	1.097	0.971
21704100	NP_663533.1#Hadhb#231086# trifunctional enzyme subunit beta, mitochondrial precursor [Mus musculus]	1.145	0.982	0.845	1.077	0.961	1.041	0.978	1.222	0.917	1.242	1.080
568955211	XP_006509614.1#Eps15l1#13859# PREDICTED: epidermal growth factor receptor substrate 15-like 1 isoform X8 [Mus musculus]	1.204	0.852	0.838	1.084	0.961	1.040	1.093	1.222	0.733	1.241	0.987
6754186	NP_034552.1#Hexb#15212# beta-hexosaminidase subunit beta precursor [Mus musculus]	1.843	0.882	0.872	1.051	0.962	1.040	1.710	1.836	1.438	1.691	1.564
755525542	XP_011240529.1#Enpp6#320981# PREDICTED: ectonucleotide pyrophosphatase/phosphodiesterase family member 6 isoform X1 [Mus musculus]	0.903	1.088	0.800	1.124	0.962	1.039	0.797	0.940	0.785	1.104	0.945
568913158	XP_006497850.1#Ptgds#19215# PREDICTED: prostaglandin-H2 D-isomerase isoform X1 [Mus musculus]	0.941	0.858	0.836	1.090	0.963	1.038	0.768	1.044	0.645	0.910	0.778
568985564	XP_006518143.1#Rpl15#66480# PREDICTED: 60S ribosomal protein L15 isoform X1 [Mus musculus]	0.996	0.939	0.888	1.038	0.963	1.038	0.939	0.987	0.827	0.980	0.903
21312256	NP_081306.1#Dcps#69305# m7GpppX diphosphatase [Mus musculus]	1.031	0.883	0.857	1.071	0.964	1.037	0.853	1.135	0.728	1.027	0.877
568912435	XP_006497552.1#Upf2#326622# PREDICTED: regulator of nonsense transcripts 2 isoform X5 [Mus musculus]	0.955	0.850	0.888	1.040	0.964	1.037	0.808	1.033	0.625	0.940	0.783
568973161	XP_006533012.1#Git1#216963# PREDICTED: ARF GTPase-activating protein GIT1 isoform X1 [Mus musculus]	1.064	0.893	0.822	1.110	0.966	1.035	0.792	1.091	0.863	1.019	0.941
771916686	NP_001292478.1#Apoa2#11807# apolipoprotein A-II preproprotein [Mus musculus]	1.048	1.013	0.915	1.017	0.966	1.035	0.947	1.078	0.963	1.089	1.026
33468923	NP_035413.1#Rp1#19888# oxygen-regulated protein 1 isoform 1 [Mus musculus]	1.095	0.838	0.796	1.136	0.966	1.035	0.969	1.147	0.757	1.017	0.887
226423909	NP_001139790.1#Cnp#12799# 2',3'-cyclic-nucleotide 3'-phosphodiesterase isoform 1 [Mus musculus]	1.031	1.270	0.856	1.077	0.966	1.035	0.895	1.031	1.030	1.348	1.189
568923910	XP_006502075.1#Bola1#69168# PREDICTED: bolA-like protein 1 isoform X1 [Mus musculus]	0.981	0.991	0.881	1.052	0.967	1.034	0.780	1.117	0.800	1.080	0.940
13384820	NP_079701.1#Rpl3l#66211# 60S ribosomal protein L3-like isoform 2 [Mus musculus]	1.188	0.841	0.849	1.085	0.967	1.034	0.987	1.311	0.890	1.043	0.967
31982755	NP_035831.2#Vim#22352# vimentin [Mus musculus]	0.939	0.982	0.865	1.072	0.969	1.032	0.774	1.045	0.729	1.057	0.893
309270949	XP_001480330.2#Gm10045#100043348# PREDICTED: 60S ribosomal protein L21-like [Mus musculus]	0.946	0.938	0.878	1.061	0.969	1.032	0.813	0.988	0.810	1.033	0.921

568999607	XP_006524007.1#Slc8a1#20541# PREDICTED: sodium/calcium exchanger 1 isoform X12 [Mus musculus]	0.816	1.020	0.888	1.055	0.972	1.029	0.740	0.847	0.767	0.851	0.809
20279132	NP_080661.1#Pllp#67801# plasmolipin [Mus musculus]	0.564	1.311	0.907	1.038	0.973	1.028	0.533	0.565	0.721	0.719	0.720
568981388	XP_006516544.1#Tubal3#238463# PREDICTED: tubulin alpha chain-like 3 isoform X2 [Mus musculus]	**2.214**	0.883	0.884	1.061	0.973	1.028	1.777	2.530	1.659	2.143	1.901
359718922	NP_001240773.1#Prpf19#28000# pre-mRNA-processing factor 19 isoform 3 [Mus musculus]	1.189	0.879	0.819	1.129	0.974	1.027	1.076	1.240	0.876	1.161	1.018
40068491	NP_951061.1#Ddx17#67040# probable ATP-dependent RNA helicase DDX17 isoform 2 [Mus musculus]	0.947	0.926	0.756	1.194	0.975	1.026	0.848	0.998	0.700	1.009	0.855
22726191	NP_689420.1#Ubqln1#56085# ubiquilin-1 isoform 2 [Mus musculus]	1.054	0.931	0.969	0.983	0.976	1.025	1.017	1.041	0.839	1.078	0.958
530678008	NP_001268990.1#Rgs6#50779# regulator of G-protein signaling 6 [Mus musculus]	1.179	0.811	0.885	1.068	0.976	1.024	1.039	1.262	0.770	1.096	0.933
21312520	NP_077198.1#Qdpr#110391# dihydropteridine reductase [Mus musculus]	1.036	1.088	0.900	1.054	0.977	1.024	0.928	1.102	0.961	1.295	1.128
568924345	XP_006502280.1#Ppa2#74776# PREDICTED: inorganic pyrophosphatase 2, mitochondrial isoform X3 [Mus musculus]	0.910	0.962	0.861	1.095	0.978	1.023	0.858	0.922	0.786	0.927	0.856
569000378	XP_006524379.1#Caskin1#268932# PREDICTED: caskin-1 isoform X4 [Mus musculus]	1.017	0.862	0.855	1.104	0.979	1.021	0.910	1.082	0.699	1.020	0.859
569004381	XP_006526249.1#Slc25a46#67453# PREDICTED: solute carrier family 25 member 46 isoform X1 [Mus musculus]	1.041	0.884	0.763	1.196	0.980	1.021	0.968	1.072	0.796	1.007	0.902
459215046	NP_001264009.1#Gas7#14457# growth arrest-specific protein 7 isoform d [Mus musculus]	1.114	0.826	0.897	1.064	0.981	1.020	1.084	1.101	0.772	1.033	0.902
211065507	NP_803228.2#Dars#226414# aspartate--tRNA ligase, cytoplasmic isoform 1 [Mus musculus]	0.886	0.912	0.858	1.104	0.981	1.020	0.834	1.013	0.725	0.932	0.828
168	FABPH_HUMAN	0.775	0.849	0.850	1.115	0.983	1.018	0.720	0.803	0.547	0.746	0.646
146219849	NP_001078918.1#Ctnnd1#12388# catenin delta-1 isoform 3 [Mus musculus]	1.118	0.851	0.842	1.123	0.983	1.017	1.039	1.198	0.729	1.158	0.943
21539587	NP_079624.1#Ndufa3#66091# NADH dehydrogenase [ubiquinone] 1 alpha subcomplex subunit 3 [Mus musculus]	1.194	0.802	0.885	1.081	0.983	1.017	1.131	1.218	0.805	1.079	0.942
6755210	NP_036005.1#Psmd13#23997# 26S proteasome non-ATPase regulatory subunit 13 [Mus musculus]	0.955	0.944	0.857	1.110	0.983	1.017	0.875	1.004	0.793	0.981	0.887
6755212	NP_035319.1#Psme1#19186# proteasome activator complex subunit 1 [Mus musculus]	1.187	0.959	0.895	1.073	0.984	1.016	1.045	1.290	1.066	1.174	1.120
124286811	NP_035034.2#Nefh#380684# neurofilament heavy polypeptide [Mus musculus]	0.803	1.240	0.871	1.100	0.986	1.014	0.738	0.864	0.826	1.166	0.996
568956483	XP_006530907.1#Tom1#21968# PREDICTED: target of Myb protein 1 isoform X1 [Mus musculus]	0.962	0.883	0.810	1.167	0.989	1.011	0.859	1.043	0.956	0.723	0.840
6678079	NP_033269.1#Serpina1a#20700# alpha-1-antitrypsin 1-1 isoform 1 precursor [Mus musculus]	0.877	0.932	0.908	1.070	0.989	1.011	0.781	0.982	0.804	0.897	0.850
156447048	NP_001095941.1#Cnnm2#94219# metal transporter CNNM2 isoform b [Mus musculus]	1.038	0.815	0.859	1.120	0.989	1.011	0.960	1.093	0.738	0.935	0.837
148762971	NP_001005424.2#Gm996#381353# uncharacterized protein C9orf172 homolog [Mus musculus]	1.066	0.931	0.846	1.135	0.991	1.010	0.870	1.104	0.835	0.978	0.907
6679186	NP_032796.1#Cldn11#18417# claudin-11 [Mus musculus]	0.974	1.095	0.876	1.109	0.992	1.008	0.879	1.113	0.902	1.136	1.019
31560404	NP_062795.2#Gltp#56356# glycolipid transfer protein [Mus musculus]	0.912	1.273	0.940	1.046	0.993	1.007	0.819	0.992	1.019	1.286	1.153
6754222	NP_034578.1#Hnrnpab#15384# heterogeneous nuclear ribonucleoprotein A/B isoform 2 [Mus musculus]	0.831	1.022	0.882	1.108	0.995	1.005	0.750	0.902	0.708	0.981	0.844
14149750	NP_075368.1#Arl6ip5#65106# PRA1 family protein 3 [Mus musculus]	1.182	0.821	0.875	1.116	0.996	1.004	1.096	1.259	0.825	1.107	0.966
755494341	XP_011246314.1#Nfasc#269116# PREDICTED: neurofascin isoform X3 [Mus musculus]	0.876	1.038	0.976	1.015	0.996	1.004	0.817	0.927	0.800	1.012	0.906
652698252	NP_001280590.1#Carkd#69225# ATP-dependent (S)-NAD(P)H-hydrate dehydratase isoform 3 [Mus musculus]	1.050	0.940	0.894	1.100	0.997	1.003	0.858	1.091	0.798	1.033	0.916
7949055	NP_057886.1#Hpcal1#53602# hippocalcin-like protein 1 [Mus musculus]	0.759	1.058	0.894	1.100	0.997	1.003	0.680	0.834	0.701	0.901	0.801
21746161	NP_076205.1#Tubb2b#73710# tubulin beta-2B chain [Mus musculus]	0.840	0.924	0.931	1.064	0.997	1.003	0.747	0.929	0.689	0.859	0.774
568889647	XP_006543795.1#Armc1#74252# PREDICTED: armadillo repeat-containing protein 1 isoform X1 [Mus musculus]	0.891	0.892	0.860	1.135	0.998	1.002	0.804	0.974	0.671	0.914	0.793
149363634	NP_001092280.1#Ptrh2#217057# peptidyl-tRNA hydrolase 2, mitochondrial isoform b [Mus musculus]	1.412	0.868	1.047	0.962	1.004	0.996	1.341	1.496	1.047	1.416	1.232
161016820	NP_058574.3#Dnpep#13437# aspartyl aminopeptidase isoform b [Mus musculus]	1.223	0.879	0.926	1.084	1.005	0.995	1.129	1.327	0.911	1.251	1.081
6680988	NP_031774.1#Cox6a1#12861# cytochrome c oxidase subunit 6A1, mitochondrial [Mus musculus]	0.828	0.905	0.992	1.024	1.008	0.992	0.686	0.970	0.603	0.907	0.755
40254507	NP_080230.2#Pgp#67078# phosphoglycolate phosphatase [Mus musculus]	1.126	0.861	0.901	1.117	1.009	0.991	1.051	1.221	0.850	1.106	0.978
237858791	NP_001153825.1#Grm2#108068# metabotropic glutamate receptor 2 precursor [Mus musculus]	1.156	0.673	0.916	1.102	1.009	0.991	1.118	1.215	0.607	0.956	0.782
228480241	NP_058084.3#Mtx2#53375# metaxin-2 [Mus musculus]	0.771	1.203	0.724	1.297	1.011	0.989	0.700	0.858	0.828	1.046	0.937
755515956	XP_011239472.1#Atp2b2#11941# PREDICTED: plasma membrane calcium-transporting ATPase 2 isoform X7 [Mus musculus]	0.987	0.951	0.903	1.129	1.016	0.985	0.880	1.066	0.748	0.989	0.868
359279956	NP_001240686.1#Anp32e#66471# acidic leucine-rich nuclear phosphoprotein 32 family member E isoform 2 [Mus musculus]	1.315	0.738	0.782	1.251	1.016	0.984	1.163	1.510	0.806	1.166	0.986
112363107	NP_032717.2#Nefm#18040# neurofilament medium polypeptide [Mus musculus]	0.749	1.301	0.920	1.114	1.017	0.983	0.713	0.827	0.846	1.155	1.001
95113671	NP_082707.1#Pmpcb#73078# mitochondrial-processing peptidase subunit beta precursor [Mus musculus]	1.170	0.829	0.852	1.195	1.023	0.977	1.247	1.147	0.850	1.134	0.992
315711043	NP_001186877.1#Dynlt1f#100040531# dynein light chain Tctex-type 1F isoform 2 [Mus musculus]	0.634	0.811	1.090	0.960	1.025	0.976	0.634	0.665	0.497	0.556	0.527
30519953	NP_848734.1#1810026J23Rik#69773# uncharacterized protein C19orf52 homolog [Mus musculus]	0.896	0.915	0.853	1.199	1.026	0.975	0.822	1.017	0.796	0.888	0.842
21644575	NP_660254.1#Lgi3#213469# leucine-rich repeat LGI family member 3 precursor [Mus musculus]	1.138	1.008	0.878	1.179	1.028	0.972	0.979	1.271	1.004	1.346	1.175
568993748	XP_006521660.1#Rbfox2#93686# PREDICTED: RNA binding protein fox-1 homolog 2 isoform X26 [Mus musculus]	0.969	0.868	0.864	1.198	1.031	0.970	0.856	1.143	0.736	0.999	0.868
568899577	XP_006543123.1#Gm8674#667507# PREDICTED: spermatogenesis-associated protein 31A6-like [Mus musculus]	0.860	0.906	0.826	1.269	1.047	0.955	0.808	0.994	0.734	0.898	0.816
13385624	NP_080390.1#Eif2s1#13665# eukaryotic translation initiation factor 2 subunit 1 [Mus musculus]	0.889	0.813	0.996	1.100	1.048	0.954	0.923	0.941	0.689	0.827	0.758
86355503	NP_001034454.1#Mobp#17433# myelin-associated oligodendrocyte basic protein isoform b [Mus musculus]	0.810	1.021	0.795	1.312	1.053	0.949	0.767	0.936	0.722	1.017	0.869
31980767	NP_038673.2#Pvalb#19293# parvalbumin alpha [Mus musculus]	**0.377**	0.847	0.937	1.185	1.061	0.942	0.377	0.422	0.306	0.371	0.338
8394490	NP_058605.1#Tsnax#53424# translin-associated protein X [Mus musculus]	1.087	0.923	0.744	1.399	1.072	0.933	1.124	1.207	0.937	1.214	1.075
755531599	XP_011241233.1#Ptpn23#104831# PREDICTED: tyrosine-protein phosphatase non-receptor type 23 isoform X1 [Mus musculus]	1.199	0.834	0.986	1.178	1.082	0.924	1.129	1.467	0.975	1.191	1.083
755514416	XP_011239203.1#Gtf2ird1#57080# PREDICTED: general transcription factor II-I repeat domain-containing protein 1 isoform X15 [Mus musculus]	1.147	1.068	0.967	1.215	1.091	0.917	1.190	1.313	1.139	1.533	1.336
568938125	XP_006504497.1#Vgf#381677# PREDICTED: neurosecretory protein VGF isoform X1 [Mus musculus]	1.067	0.792	0.890	1.296	1.093	0.915	1.157	1.174	0.808	1.037	0.923
568939614	XP_006505145.1#Ppp1r9a#243725# PREDICTED: neurabin-1 isoform X9 [Mus musculus]	0.976	1.013	0.942	1.264	1.103	0.907	0.998	1.156	0.951	1.231	1.091
568953852	XP_006509066.1#Ank1#11733# PREDICTED: ankyrin-1 isoform X16 [Mus musculus]	1.042	1.061	1.001	1.207	1.104	0.906	1.126	1.173	1.085	1.355	1.220
39204499	NP_035040.1#Nefl#18039# neurofilament light polypeptide [Mus musculus]	0.597	1.438	0.999	1.231	1.115	0.897	0.628	0.710	0.792	1.064	0.928
755531806	XP_011241250.1#Exog#208194# PREDICTED: nuclease EXOG, mitochondrial isoform X2 [Mus musculus]	1.283	0.731	0.874	1.365	1.120	0.893	1.060	1.814	0.884	1.218	1.051
568918287	XP_006500185.1#Rpap1#68925# PREDICTED: RNA polymerase II-associated protein 1 isoform X2 [Mus musculus]	0.869	0.906	1.109	1.136	1.123	0.891	0.962	0.990	0.783	0.986	0.884
568979594	XP_006515904.1#Syt16#238266# PREDICTED: synaptotagmin-16 isoform X3 [Mus musculus]	0.664	0.875	0.764	1.494	1.129	0.886	0.669	0.830	0.571	0.742	0.656
148539957	NP_666212.3#Ina#226180# alpha-internexin [Mus musculus]	0.641	1.358	1.034	1.243	1.138	0.878	0.674	0.831	0.880	1.195	1.038
62460366	NP_081264.1#Ptms#69202# parathymosin [Mus musculus]	0.700	0.973	0.743	1.607	1.175	0.851	0.651	0.993	0.670	0.931	0.800
72004262	NP_001025445.1#Ndufs5#595136# NADH dehydrogenase [ubiquinone] iron-sulfur protein 5 [Mus musculus]	0.832	0.842	0.745	1.632	1.189	0.841	0.947	1.126	0.738	1.009	0.874
167234372	NP_663600.2#Eif4b#75705# eukaryotic translation initiation factor 4B [Mus musculus]	0.971	0.847	1.191	1.404	1.298	0.771	1.020	1.499	0.954	1.179	1.066

index

upregulated in kostress/ko	20
downregulated in kostress/ko	994
upregulated in kostress/wtstress	20
downregulated in kostress/wtstress	280
upregulated in wtstress/wt	275
downregulated in wtstress/wt	63
upregulated in ko/wt	751
downregulated in ko/wt	1
