# Supplementary material for: Absence of Wdr13 Gene Predisposes Mice to Mild Social Isolation – Chronic Stress, Leading to Depression-Like Phenotype Associated With Differential Expression of Synaptic Proteins
Source: Front Mol Neurosci. 2018 Apr 25;11:133. doi: 10.3389/fnmol.2018.00133 (PMC5930177; doi:10.3389/fnmol.2018.00133)
Supplement: TABLE S2 — Proteomics (8 plex iTRAQ) of PFC from Wdr13+/0 and Wdr13-/0 mice before and after 3 weeks social isolation (at-least two unique peptides). [file Table_2.PDF]

Accession	Description
72004262	NP_001025445.1#Ndufs5#595136# NADH dehydrogenase [ubiquinone] iron-sulfur
148539957	NP_666212.3#Ina#226180# alpha-internexin [Mus musculus]
39204499	NP_035040.1#Nefl#18039# neurofilament light polypeptide [Mus musculus]
86355503	NP_001034454.1#Mobp#17433# myelin-associated oligodendrocyte basic protein i
112363107	NP_032717.2#Nefm#18040# neurofilament medium polypeptide [Mus musculus]
161016820	NP_058574.3#Dnpep#13437# aspartyl aminopeptidase isoform b [Mus musculus]
7949055	NP_057886.1#Hpcal1#53602# hippocalcin-like protein 1 [Mus musculus]
31560404	NP_062795.2#Gltp#56356# glycolipid transfer protein [Mus musculus]
148762971	NP_001005424.2#Gm996#381353# uncharacterized protein C9orf172 homolog [Mu
6678079	NP_033269.1#Serpina1a#20700# alpha-1-antitrypsin 1-1 isoform 1 precursor [Mus
124286811	NP_035034.2#Nefh#380684# neurofilament heavy polypeptide [Mus musculus]
146219849	NP_001078918.1#Ctnnd1#12388# catenin delta-1 isoform 3 [Mus musculus]
211065507	NP_803228.2#Dars#226414# aspartate--tRNA ligase, cytoplasmic isoform 1 [Mus m
569000378	XP_006524379.1#Caskin1#268932# PREDICTED: caskin-1 isoform X4 [Mus musculus
21312520	NP_077198.1#Qdpr#110391# dihydropteridine reductase [Mus musculus]
530678008	NP_001268990.1#Rgs6#50779# regulator of G-protein signaling 6 [Mus musculus]
309270949	XP_001480330.2#Gm10045#100043348# PREDICTED: 60S ribosomal protein L21-lik
226423909	NP_001139790.1#Cnp#12799# 2',3'-cyclic-nucleotide 3'-phosphodiesterase isoform
568973161	XP_006533012.1#Git1#216963# PREDICTED: ARF GTPase-activating protein GIT1 isc
568985564	XP_006518143.1#Rpl15#66480# PREDICTED: 60S ribosomal protein L15 isoform X1
21704100	NP_663533.1#Hadhb#231086# trifunctional enzyme subunit beta, mitochondrial pr
568928764	XP_006502995.1#Urod#22275# PREDICTED: uroporphyrinogen decarboxylase isofo
6755376	NP_035430.1#Rps7#20115# 40S ribosomal protein S7 [Mus musculus]
133922578	NP_613069.3#Ccdc22#54638# coiled-coil domain-containing protein 22 [Mus musc
188219589	NP_034851.2#Lmnb1#16906# lamin-B1 [Mus musculus]
568908913	XP_006529574.1#2310035C23Rik#227446# PREDICTED: lisH domain and HEAT repe
807066384	NP_001292910.1#Diap1#13367# protein diaphanous homolog 1 isoform 3 [Mus mu
7242138	NP_038201.1#Atp1b2#11932# sodium/potassium-transporting ATPase subunit beta
155	ANXA5_HUMAN
170763467	NP_035722.2#Timm44#21856# mitochondrial import inner membrane translocase
226823309	NP_001152847.1#Eif4a1#13681# eukaryotic initiation factor 4A-I isoform 2 [Mus m
260166719	NP_083713.2#Ckap5#75786# cytoskeleton-associated protein 5 isoform 2 [Mus mu
568971480	XP_006532202.1#Mpp3#13384# PREDICTED: MAGUK p55 subfamily member 3 isof
568915467	XP_006498830.1#Gad1#14415# PREDICTED: glutamate decarboxylase 1 isoform X2
334688858	NP_001229310.1#Slc4a10#94229# sodium-driven chloride bicarbonate exchanger is
83921618	NP_033536.2#Ezr#22350# ezrin [Mus musculus]
244789999	NP_619611.3#Mpst#246221# 3-mercaptopyruvate sulfurtransferase [Mus musculus
125490380	NP_033534.2#Slc32a1#22348# vesicular inhibitory amino acid transporter [Mus mu
548923858	NP_001271327.1#Sept4#18952# septin-4 isoform 4 [Mus musculus]
13385872	NP_080650.1#Ilf2#67781# interleukin enhancer-binding factor 2 [Mus musculus]
6754084	NP_034488.1#Gstm1#14862# glutathione S-transferase Mu 1 [Mus musculus]
568939016	XP_006504897.1#Wasf3#245880# PREDICTED: wiskott-Aldrich syndrome protein fa
568915490	XP_006498839.1#Gnas#14683# PREDICTED: protein GNAS isoform X5 [Mus musculu
6755256	NP_035354.1#Pygm#19309# glycogen phosphorylase, muscle form [Mus musculus]
6996917	NP_032088.1#G6pdx#14381# glucose-6-phosphate 1-dehydrogenase X [Mus muscu

568954511 XP_006509328.1#Acsl1#14081# PREDICTED: long-chain-fatty-acid--CoA ligase 1 isof
19527154 NP_598698.1#Brk1#101314# protein BRICK1 [Mus musculus]
7106331 NP_034566.1#H2afx#15270# histone H2AX [Mus musculus]
19527388 NP_598911.1#Otub1#107260# ubiquitin thioesterase OTUB1 [Mus musculus]
6753242 NP_033918.1#Calb1#12307# calbindin [Mus musculus]
21536256 NP_659077.1#Ppp1r1b#19049# protein phosphatase 1 regulatory subunit 1B [Mus
568950272 XP_006507720.1#Ipo7#233726# PREDICTED: importin-7 isoform X1 [Mus musculus]
357197139 NP_001239398.1#Bag6#224727# large proline-rich protein BAG6 isoform 3 [Mus m
568908524 XP_006529387.1#Syt2#20980# PREDICTED: synaptotagmin-2 isoform X2 [Mus musc
568916096 XP_006499132.1#Ssb#20823# PREDICTED: lupus La protein homolog isoform X1 [M
238814391 NP_031664.3#Cct7#12468# T-complex protein 1 subunit eta [Mus musculus]
22203763 NP_038522.2#Cpe#12876# carboxypeptidase E precursor [Mus musculus]
600971669 NP_001278115.1#Abr#109934# active breakpoint cluster region-related protein iso
227499240 NP_001153098.1#Hagh#14651# hydroxyacylglutathione hydrolase, mitochondrial is
568907510 XP_006496471.1#Epha4#13838# PREDICTED: ephrin type-A receptor 4 isoform X2 [
568918221 XP_006500154.1#Fahd2a#68126# PREDICTED: fumarylacetoacetate hydrolase dom
69885049 NP_001020426.1#Mbp#17196# myelin basic protein isoform 3 [Mus musculus]
13385392 NP_080174.1#Napa#108124# alpha-soluble NSF attachment protein [Mus musculus
568972828 XP_006532849.1#Skp1a#21402# PREDICTED: S-phase kinase-associated protein 1 is
31981458 NP_444338.2#Glrx#93692# glutaredoxin-1 [Mus musculus]
568954159 XP_006509214.1#Hook3#320191# PREDICTED: protein Hook homolog 3 isoform X4
568921903 XP_006501115.1#Kcna2#16490# PREDICTED: potassium voltage-gated channel sub
113680352 NP_031646.2#Cbr1#12408# carbonyl reductase [NADPH] 1 [Mus musculus]
33859801 NP_789818.1#Fbxo2#230904# F-box only protein 2 [Mus musculus]
112181167 NP_031599.2#C1qbp#12261# complement component 1 Q subcomponent-binding
594591591 NP_001277563.1#Ugp2#216558# UTP--glucose-1-phosphate uridylyltransferase iso
242397487 NP_001156318.1#Phyhipl#70911# phytanoyl-CoA hydroxylase-interacting protein-li
40807498 NP_032313.2#Hrsp12#15473# ribonuclease UK114 [Mus musculus]
281485615 NP_035017.2#Ndufs4#17993# NADH dehydrogenase [ubiquinone] iron-sulfur prote
13569841 NP_056577.2#Txnrd1#50493# thioredoxin reductase 1, cytoplasmic isoform 2 [Mus
568960685 XP_006510846.1#Atp1b3#11933# PREDICTED: sodium/potassium-transporting ATP
126032329 NP_034236.2#Eef1a1#13627# elongation factor 1-alpha 1 [Mus musculus]
170650632 NP_001116237.1#Sirt2#64383# NAD-dependent protein deacetylase sirtuin-2 isofo
6755202 NP_036101.1#Psmb3#26446# proteasome subunit beta type-3 [Mus musculus]
225735655 NP_001139595.1#Psap#19156# prosaposin isoform E preproprotein [Mus musculus
568938961 XP_006504870.1#Hmgb1#15289# PREDICTED: high mobility group protein B1 isofor
70909327 NP_034069.2#Cops2#12848# COP9 signalosome complex subunit 2 isoform b [Mus
160333923 NP_058085.2#Hnrnpu#51810# heterogeneous nuclear ribonucleoprotein U [Mus m
568911073 XP_006497013.1#Aldh9a1#56752# PREDICTED: 4-trimethylaminobutyraldehyde de
568927577 XP_006538412.1#Slc24a2#76376# PREDICTED: sodium/potassium/calcium exchang
158186704 NP_001103383.1#Hnrnpm#76936# heterogeneous nuclear ribonucleoprotein M isc
227499988 NP_001153105.1#Slc44a1#100434# choline transporter-like protein 1 isoform B [M
100817933 NP_766266.3#Acad9#229211# acyl-CoA dehydrogenase family member 9, mitocho
19527256 NP_598801.1#Ddx1#104721# ATP-dependent RNA helicase DDX1 [Mus musculus]
569012136 XP_006528983.1#Acot9#56360# PREDICTED: acyl-coenzyme A thioesterase 9, mito
225543196 NP_001139431.1#Ndrg2#29811# protein NDRG2 isoform 2 [Mus musculus]

31542602	NP_034615.2#Elavl1#15568# ELAV-like protein 1 [Mus musculus]
569003063	XP_006525609.1#Camk4#12326# PREDICTED: calcium/calmodulin-dependent prote
227330633	NP_082408.3#Pgm2#72157# phosphoglucomutase-2 [Mus musculus]
31981945	NP_033464.2#Rpl13a#22121# 60S ribosomal protein L13a [Mus musculus]
12963571	NP_075691.1#Ndufa7#66416# NADH dehydrogenase [ubiquinone] 1 alpha subcom
568907697	XP_006496561.1#Cul3#26554# PREDICTED: cullin-3 isoform X3 [Mus musculus]
6753022	NP_033777.1#Ak4#11639# adenylate kinase 4, mitochondrial [Mus musculus]
7549752	NP_036172.1#Cul1#26965# cullin-1 [Mus musculus]
227330544	NP_031782.3#Cplx1#12889# complexin-1 [Mus musculus]
85861231	NP_001034290.1#Hdhd2#76987# haloacid dehalogenase-like hydrolase domain-co
568908693	XP_006529469.1#Rab3gap1#226407# PREDICTED: rab3 GTPase-activating protein c
261824000	NP_035314.3#Psma3#19167# proteasome subunit alpha type-3 [Mus musculus]
34328471	NP_062364.3#Rala#56044# ras-related protein Ral-A precursor [Mus musculus]
755538972	XP_011247395.1#Myo18a#360013# PREDICTED: unconventional myosin-XVIIIa isof
568990347	XP_006520079.1#Cct5#12465# PREDICTED: T-complex protein 1 subunit epsilon iso
171184435	NP_067320.2#Hacd3#57874# very-long-chain (3R)-3-hydroxyacyl-CoA dehydratase
755506697	XP_011248385.1#Nudt2#66401# PREDICTED: bis(5'-nucleosyl)-tetraphosphatase [a
33563282	NP_036095.1#Psma1#26440# proteasome subunit alpha type-1 [Mus musculus]
165377150	NP_001004357.2#Cntnap2#66797# contactin-associated protein-like 2 isoform a pr
672424492	NP_001288274.1#Hnrnpk#15387# heterogeneous nuclear ribonucleoprotein K isof
84662745	NP_032165.3#Gnaq#14682# guanine nucleotide-binding protein G(q) subunit alpha
170763483	NP_001116292.1#Ap3m2#64933# AP-3 complex subunit mu-2 [Mus musculus]
31543330	NP_032736.2#Nnt#18115# NAD(P) transhydrogenase, mitochondrial isoform 1 prec
568995055	XP_006522062.1#Tfrc#22042# PREDICTED: transferrin receptor protein 1 isoform X
258547156	NP_035182.2#Pdcd6ip#18571# programmed cell death 6-interacting protein isofor
568955180	XP_006509601.1#Ap1m1#11767# PREDICTED: AP-1 complex subunit mu-1 isoform
6755863	NP_035761.1#Hsp90b1#22027# endoplasmin precursor [Mus musculus]
6679337	NP_032876.1#Pitpna#18738# phosphatidylinositol transfer protein alpha isoform [
21313144	NP_080218.1#Ola1#67059# obg-like ATPase 1 isoform a [Mus musculus]
26006861	NP_742146.1#Pdxk#216134# pyridoxal kinase [Mus musculus]
37202121	NP_766549.2#Abat#268860# 4-aminobutyrate aminotransferase, mitochondrial iso
6678768	NP_032564.1#Marcks#17118# myristoylated alanine-rich C-kinase substrate [Mus r
45597447	NP_035564.1#Sod1#20655# superoxide dismutase [Cu-Zn] [Mus musculus]
116734870	NP_032543.2#Lta4h#16993# leukotriene A-4 hydrolase [Mus musculus]
160333789	NP_035597.2#Spr#20751# sepiapterin reductase [Mus musculus]
196115327	NP_001124492.1#Gfap#14580# glial fibrillary acidic protein isoform 1 [Mus muscul
6754086	NP_034490.1#Gstm5#14866# glutathione S-transferase Mu 5 [Mus musculus]
257153448	NP_038920.2#Pcsk1n#30052# proSAAS precursor [Mus musculus]
160298217	NP_034487.2#Gsta4#14860# glutathione S-transferase A4 [Mus musculus]
255003777	NP_080305.2#Glod4#67201# glyoxalase domain-containing protein 4 [Mus muscul
568929710	XP_006503443.1#Smap2#69780# PREDICTED: stromal membrane-associated protei
254675251	NP_958792.2#Plec#18810# plectin isoform 1d [Mus musculus]
755519133	XP_011248743.1#Mag#17136# PREDICTED: myelin-associated glycoprotein isoform
568972945	XP_006532907.1#Acsl6#216739# PREDICTED: long-chain-fatty-acid--CoA ligase 6 isc
568944898	XP_006539622.1#Hnrnpl#15388# PREDICTED: heterogeneous nuclear ribonucleopr
22122795	NP_666341.1#Dync1li1#235661# cytoplasmic dynein 1 light intermediate chain 1 [

258547146	NP_001158143.1#Dnaja1#15502# dnaJ homolog subfamily A member 1 [Mus musc
575501696	NP_001276460.1#Cndp2#66054# cytosolic non-specific dipeptidase [Mus musculus
568933657	XP_006503744.1#Add1#11518# PREDICTED: alpha-adducin isoform X13 [Mus musc
30520239	NP_848887.1#Nceh1#320024# neutral cholesterol ester hydrolase 1 [Mus musculus
6681095	NP_031834.1#Cycs#13063# cytochrome c, somatic [Mus musculus]
755499302	XP_011237757.1#Madd#228355# PREDICTED: MAP kinase-activating death domain
30409956	NP_848492.1#Atp1a2#98660# sodium/potassium-transporting ATPase subunit alph
18087805	NP_032529.2#Rps2#16898# 40S ribosomal protein S2 [Mus musculus]
112293266	NP_032326.3#Hspa4#15525# heat shock 70 kDa protein 4 [Mus musculus]
111955312	NP_034948.3#Cd200#17470# OX-2 membrane glycoprotein precursor [Mus muscul
300388153	NP_001177956.1#Dync1i1#13426# cytoplasmic dynein 1 intermediate chain 1 isofo
17933768	NP_525028.1#Grhpr#76238# glyoxylate reductase/hydroxypyruvate reductase [Mu
568965003	XP_006512604.1#Gja1#14609# PREDICTED: gap junction alpha-1 protein isoform X
12025542	NP_062626.1#Asna1#56495# ATPase Asna1 [Mus musculus]
38142460	NP_080971.2#Etfb#110826# electron transfer flavoprotein subunit beta [Mus musc
21539599	NP_079917.1#Uqcrh#66576# cytochrome b-c1 complex subunit 6, mitochondrial [
568973607	XP_006533222.1#Ubb#22187# PREDICTED: polyubiquitin-B isoform X1 [Mus muscu
755513544	XP_006530271.2#Rasal1#19415# PREDICTED: rasGAP-activating-like protein 1 isofo
569007898	XP_006527442.1#Hspa12a#73442# PREDICTED: heat shock 70 kDa protein 12A isof
568961907	XP_006511433.1#Pccb#66904# PREDICTED: propionyl-CoA carboxylase beta chain,
459683868	NP_001264051.1#Hnrnpr#74326# heterogeneous nuclear ribonucleoprotein R isofo
568942420	XP_006506449.1#Copg1#54161# PREDICTED: coatomer subunit gamma-1 isoform
283135110	NP_758512.3#Mthfd1l#270685# monofunctional C1-tetrahydrofolate synthase, mit
241982783	NP_766086.2#Ptk2b#19229# protein-tyrosine kinase 2-beta isoform 3 [Mus muscul
6755983	NP_036168.1#Vsnl1#26950# visinin-like protein 1 [Mus musculus]
28372479	NP_077228.1#Rps25#75617# 40S ribosomal protein S25 [Mus musculus]
165377065	NP_079923.3#Cmpk1#66588# UMP-CMP kinase [Mus musculus]
30520131	NP_848818.1#Slc6a1#232333# sodium- and chloride-dependent GABA transporter
158711686	NP_065066.2#Slc12a5#57138# solute carrier family 12 member 5 [Mus musculus]
31560239	NP_079886.2#Asrgl1#66514# isoaspartyl peptidase/L-asparaginase [Mus musculus]
358439483	NP_001240610.1#Immt#76614# MICOS complex subunit Mic60 isoform 2 [Mus mu
568940569	XP_006505556.1#Dctn1#13191# PREDICTED: dynactin subunit 1 isoform X9 [Mus m
228008337	NP_032974.2#Psmc3#19182# 26S protease regulatory subunit 6A [Mus musculus]
163954948	NP_034743.3#Khsrp#16549# far upstream element-binding protein 2 [Mus muscul
755547658	XP_011243224.1#Pcca#110821# PREDICTED: propionyl-CoA carboxylase alpha chai
37700232	NP_032730.1#Nme1#18102# nucleoside diphosphate kinase A [Mus musculus]
568995370	XP_006522210.1#Mapk1#26413# PREDICTED: mitogen-activated protein kinase 1 is
568992018	XP_006520824.1#Fam49b#223601# PREDICTED: protein FAM49B isoform X1 [Mus
12963737	NP_076054.1#Cse1l#110750# exportin-2 [Mus musculus]
16716503	NP_444429.1#Cadm3#94332# cell adhesion molecule 3 precursor [Mus musculus]
89001109	NP_034433.3#Gna13#14674# guanine nucleotide-binding protein subunit alpha-13
568982851	XP_006517069.1#Hist1h2al#667728# PREDICTED: histone H2A type 2-A, partial [M
257900520	NP_064323.2#Mpp6#56524# MAGUK p55 subfamily member 6 isoform b [Mus mus
568974509	XP_006533648.1#Myo1d#338367# PREDICTED: unconventional myosin-Id isoform
88014720	NP_032405.3#Kpnb1#16211# importin subunit beta-1 [Mus musculus]
568993633	XP_006521604.1#Sbf1#77980# PREDICTED: myotubularin-related protein 5 isoform

161484668 NP_766295.2#Ppp1cb#19046# serine/threonine-protein phosphatase PP1-beta cata
71043944 NP_062701.2#Snx1#56440# sorting nexin-1 [Mus musculus]
6754220 NP_034577.1#Hnrnpa1#15382# heterogeneous nuclear ribonucleoprotein A1 isofo
23956058 NP_035253.1#Plp1#18823# myelin proteolipid protein isoform 1 [Mus musculus]
568979727 XP_006515970.1#Gphn#268566# PREDICTED: gephyrin isoform X9 [Mus musculus]
21313162 NP_083852.1#Rab1b#76308# ras-related protein Rab-1B [Mus musculus]
319402143 NP_001188320.1#Hbb-bs#100503605# hemoglobin, beta adult s chain [Mus muscu
171906578 NP_033037.2#Rad23b#19359# UV excision repair protein RAD23 homolog B [Mus n
568988763 XP_006519603.1#Ipo5#70572# PREDICTED: importin-5 isoform X3 [Mus musculus]
31560819 NP_032446.2#Kcnb1#16500# potassium voltage-gated channel subfamily B membe
116256491 NP_733924.2#Ank3#11735# ankyrin-3 isoform a [Mus musculus]
6754994 NP_035995.1#Pcbp1#23983# poly(rC)-binding protein 1 [Mus musculus]
755566692 XP_011246162.1#Huwe1#59026# PREDICTED: E3 ubiquitin-protein ligase HUWE1 is
569010246 XP_006528149.1#Atp2b3#320707# PREDICTED: plasma membrane calcium-transpo
176865892 NP_038534.2#Eif4a2#13682# eukaryotic initiation factor 4A-II isoform a [Mus musc
31981939 NP_033477.2#Tubb4a#22153# tubulin beta-4A chain [Mus musculus]
29789289 NP_444349.1#Echs1#93747# enoyl-CoA hydratase, mitochondrial precursor [Mus m
568904641 XP_006544819.1#LOC102642938#102642938# PREDICTED: heterogeneous nuclear
755565097 XP_011245805.1#Ogt#108155# PREDICTED: UDP-N-acetylglucosamine--peptide N-a
33504483 NP_084043.1#Rps9#76846# 40S ribosomal protein S9 [Mus musculus]
568908101 XP_006529181.1#Dhx9#13211# PREDICTED: ATP-dependent RNA helicase A isoforn
158966704 NP_038675.2#Rps16#20055# 40S ribosomal protein S16 [Mus musculus]
171543899 NP_786926.2#Plxna4#243743# plexin-A4 precursor [Mus musculus]
755499054 XP_011237700.1#Slc1a2#20511# PREDICTED: excitatory amino acid transporter 2 is
33468857 NP_032274.1#Hint1#15254# histidine triad nucleotide-binding protein 1 [Mus musc
33667095 NP_766604.2#Vat1l#270097# synaptic vesicle membrane protein VAT-1 homolog-li
21450277 NP_659149.1#Atp1a1#11928# sodium/potassium-transporting ATPase subunit alph
755512767 XP_011247846.1#Slc4a4#54403# PREDICTED: electrogenic sodium bicarbonate cotr
227330561 NP_034227.3#Sparcl1#13602# SPARC-like protein 1 precursor [Mus musculus]
568926471 XP_006537875.1#N28178#230085# PREDICTED: protein KIAA1045 isoform X3 [Mus
56790900 NP_001007222.1#Adam22#11496# disintegrin and metalloproteinase domain-cont
6753136 NP_033850.1#Atox1#11927# copper transport protein ATOX1 [Mus musculus]
9790125 NP_062728.1#Tagln3#56370# transgelin-3 [Mus musculus]
162417975 NP_034627.3#Idh1#15926# isocitrate dehydrogenase [NADP] cytoplasmic [Mus mu
755544182 XP_011242598.1#Ryr2#20191# PREDICTED: ryanodine receptor 2 isoform X2 [Mus
569011419 XP_006528639.1#Tceal5#331532# PREDICTED: transcription elongation factor A pro
262263372 NP_057870.3#Ahcy#269378# adenosylhomocysteinase [Mus musculus]
241982771 NP_001001983.2#Pi4ka#224020# phosphatidylinositol 4-kinase alpha [Mus muscul
568979680 XP_006515947.1#Psma6#26443# PREDICTED: proteasome subunit alpha type-6 isof
6754024 NP_034447.1#Gng4#14706# guanine nucleotide-binding protein G(I)/G(S)/G(O) sub
9790141 NP_062798.1#Arpc3#56378# actin-related protein 2/3 complex subunit 3 [Mus mus
24233554 NP_683740.1#Slc1a3#20512# excitatory amino acid transporter 1 [Mus musculus]
226823367 NP_034339.2#Fh1#14194# fumarate hydratase, mitochondrial precursor [Mus mus
25141233 NP_034901.2#Matr3#17184# matrin-3 [Mus musculus]
356582492 NP_001239217.1#Ogdh#18293# 2-oxoglutarate dehydrogenase, mitochondrial isof
568962755 XP_006511697.1#Bsn#12217# PREDICTED: protein bassoon isoform X1 [Mus muscu

18859597	NP_077182.1#Ndufc2#68197# NADH dehydrogenase [ubiquinone] 1 subunit C2 [Mu
568961317	XP_006511152.1#Lingo1#235402# PREDICTED: leucine-rich repeat and immunoglol
6681137	NP_031856.1#Dbi#13167# acyl-CoA-binding protein isoform 2 [Mus musculus]
6680832	NP_031615.1#Calm2#12314# calmodulin [Mus musculus]
226443091	NP_084148.1#Hnrnpa0#77134# heterogeneous nuclear ribonucleoprotein A0 [Mus
568979853	XP_006516032.1#Nrcam#319504# PREDICTED: neuronal cell adhesion molecule iso
411147387	NP_598912.4#Yars#107271# tyrosine--tRNA ligase, cytoplasmic [Mus musculus]
407027854	NP_001258335.1#Capzb#12345# F-actin-capping protein subunit beta isoform d [M
70778812	NP_780421.2#Tmem65#74868# transmembrane protein 65 [Mus musculus]
134031994	NP_032970.2#Psma2#19166# proteasome subunit alpha type-2 [Mus musculus]
30794450	NP_077174.1#Rpl4#67891# 60S ribosomal protein L4 [Mus musculus]
326381098	NP_001191908.1#Sars#20226# serine--tRNA ligase, cytoplasmic isoform 2 [Mus mu
313151222	NP_001186225.1#Acly#104112# ATP-citrate synthase isoform 1 [Mus musculus]
569003472	XP_006525808.1#Mapre2#212307# PREDICTED: microtubule-associated protein RP
24418919	NP_722476.1#Pygb#110078# glycogen phosphorylase, brain form [Mus musculus]
568904952	XP_006544850.1#LOC102641949#102641949# PREDICTED: ES1 protein homolog, m
569004246	XP_006526183.1#Ube2d2a#56550# PREDICTED: ubiquitin-conjugating enzyme E2 D
122	K22E_HUMAN
31982520	NP_031407.2#Acadl#11363# long-chain specific acyl-CoA dehydrogenase, mitochor
595763404	NP_001277728.1#Inpp4a#269180# type I inositol 3,4-bisphosphate 4-phosphatase
41281679	NP_619733.1#Gnb5#14697# guanine nucleotide-binding protein subunit beta-5 iso
755553088	XP_011244110.1#Arvcf#11877# PREDICTED: armadillo repeat protein deleted in vel
239985479	NP_001155268.1#Gucy1b3#54195# guanylate cyclase soluble subunit beta-1 isofor
568978506	XP_006515387.1#Rtn1#104001# PREDICTED: reticulon-1 isoform X1 [Mus musculus
6680502	NP_032436.1#Itm2b#16432# integral membrane protein 2B [Mus musculus]
755531683	XP_011241239.1#Map4#17758# PREDICTED: microtubule-associated protein 4 isof
116268115	NP_057918.2#Auh#11992# methylglutaconyl-CoA hydratase, mitochondrial precurs
225007593	NP_034573.2#Hmox2#15369# heme oxygenase 2 [Mus musculus]
13195604	NP_077137.1#Rps23#66475# 40S ribosomal protein S23 [Mus musculus]
10946940	NP_067493.1#Rab2a#59021# ras-related protein Rab-2A [Mus musculus]
568929440	XP_006503316.1#Akr1a1#58810# PREDICTED: alcohol dehydrogenase [NADP(+)] isc
164565394	NP_766292.2#Dnajc11#230935# dnaJ homolog subfamily C member 11 [Mus muscu
568936184	XP_006535270.1#Sec31a#69162# PREDICTED: protein transport protein Sec31A iso
13430890	NP_056602.1#Hist1h1e#50709# histone H1.4 [Mus musculus]
23346461	NP_694704.1#Ndufs2#226646# NADH dehydrogenase [ubiquinone] iron-sulfur prot
755555583	XP_011244521.1#Mpc1#55951# PREDICTED: mitochondrial pyruvate carrier 1 isofo
27734986	NP_663517.2#Ahcyl1#229709# putative adenosylhomocysteinase 2 [Mus musculus
6681069	NP_031817.1#Csrp1#13007# cysteine and glycine-rich protein 1 [Mus musculus]
124487313	NP_001074550.1#Gls#14660# glutaminase kidney isoform, mitochondrial isoform 1
124339829	NP_034609.2#Hspa1a#193740# heat shock 70 kDa protein 1A [Mus musculus]
569003989	XP_006526059.1#Rpl17#319195# PREDICTED: 60S ribosomal protein L17 isoform X
21728376	NP_075891.1#Myl12b#67938# myosin regulatory light chain 12B [Mus musculus]
427918099	NP_001258790.1#Add2#11519# beta-adducin isoform 2 [Mus musculus]
161086893	NP_598542.3#Cab39#12283# calcium-binding protein 39 [Mus musculus]
651164671	NP_001280550.1#Ganab#14376# neutral alpha-glucosidase AB isoform 2 precursor
13385374	NP_080163.1#Rab5a#271457# ras-related protein Rab-5A [Mus musculus]

30061351	NP_835582.1#Hist1h4j#319159# histone H4 [Mus musculus]
6753966	NP_034401.1#Gpd1#14555# glycerol-3-phosphate dehydrogenase [NAD(+)], cytopl
6677839	NP_033141.1#S100b#20203# protein S100-B [Mus musculus]
219275596	NP_081626.2#Nars#70223# asparagine--tRNA ligase, cytoplasmic isoform 2 [Mus m
568975422	XP_006534088.1#Snap47#67826# PREDICTED: synaptosomal-associated protein 47
357394768	NP_001239422.1#Osbpl1a#64291# oxysterol-binding protein-related protein 1 isof
755538368	XP_011247262.1#Ube2o#217342# PREDICTED: E2/E3 hybrid ubiquitin-protein ligas
411147445	NP_001258657.1#Trim2#80890# tripartite motif-containing protein 2 isoform 2 [M
568970049	XP_006514664.1#Sptbn1#20742# PREDICTED: spectrin beta chain, non-erythrocytic
568928284	XP_006502760.1#Cap1#12331# PREDICTED: adenylyl cyclase-associated protein 1 is
6680047	NP_032169.1#Gnb2l1#14694# guanine nucleotide-binding protein subunit beta-2-li
9790285	NP_062754.1#Vps29#56433# vacuolar protein sorting-associated protein 29 [Mus r
56790921	NP_780398.2#Hepacam#72927# hepatocyte cell adhesion molecule precursor [Mus
33859811	NP_849209.1#Hadha#97212# trifunctional enzyme subunit alpha, mitochondrial pr
189409138	NP_082270.1#Cand1#71902# cullin-associated NEDD8-dissociated protein 1 [Mus n
254675270	NP_033121.2#Rps5#20103# 40S ribosomal protein S5 [Mus musculus]
568938650	XP_006504732.1#Adap1#231821# PREDICTED: arf-GAP with dual PH domain-contai
568933716	XP_006503773.1#Ctbp1#13016# PREDICTED: C-terminal-binding protein 1 isoform
165932333	NP_001107032.1#Glo1#109801# lactoylglutathione lyase [Mus musculus]
30089688	NP_835738.1#Arrb1#109689# beta-arrestin-1 isoform B [Mus musculus]
6755901	NP_035783.1#Tuba1a#22142# tubulin alpha-1A chain [Mus musculus]
110625979	NP_080283.3#Eef1g#67160# elongation factor 1-gamma [Mus musculus]
84000009	NP_036144.2#Gprin1#26913# G protein-regulated inducer of neurite outgrowth 1 [
255759902	NP_776101.3#Maoa#17161# amine oxidase [flavin-containing] A [Mus musculus]
6680724	NP_031507.1#Arf6#11845# ADP-ribosylation factor 6 [Mus musculus]
21450129	NP_659033.1#Acat1#110446# acetyl-CoA acetyltransferase, mitochondrial precurs
157951596	NP_033931.4#Car2#12349# carbonic anhydrase 2 [Mus musculus]
19526936	NP_598498.1#Lancl2#71835# lanC-like protein 2 [Mus musculus]
30519971	NP_848743.1#Atl1#73991# atlastin-1 [Mus musculus]
71061455	NP_001021118.1#Dnm1l#74006# dynamin-1-like protein isoform b [Mus musculus]
569002922	XP_006525541.1#Wdr7#104082# PREDICTED: WD repeat-containing protein 7 isofc
54607098	NP_075770.1#Sdha#66945# succinate dehydrogenase [ubiquinone] flavoprotein su
7305395	NP_038660.1#Pnp#18950# purine nucleoside phosphorylase [Mus musculus]
552953687	NP_001272802.1#Trim3#55992# tripartite motif-containing protein 3 isoform b [M
112293264	NP_031978.2#Pdia3#14827# protein disulfide-isomerase A3 precursor [Mus muscul
85362729	NP_001034228.1#Camk2g#12325# calcium/calmodulin-dependent protein kinase t
74315975	NP_081633.1#Psmd1#70247# 26S proteasome non-ATPase regulatory subunit 1 [M
568956344	XP_006530838.1#Rbmxl1#19656# PREDICTED: RNA binding motif protein, X-linked-
164698474	NP_035500.2#Cyfip1#20430# cytoplasmic FMR1-interacting protein 1 isoform a [M
569003981	XP_006526055.1#Bin1#30948# PREDICTED: myc box-dependent-interacting protein
27370360	NP_766478.1#Slc6a11#243616# sodium- and chloride-dependent GABA transporte
7710086	NP_057885.1#Rab10#19325# ras-related protein Rab-10 [Mus musculus]
568931796	XP_006539191.1#Sdhb#67680# PREDICTED: succinate dehydrogenase [ubiquinone]
34996495	NP_062616.2#Rpn2#20014# dolichyl-diphosphooligosaccharide--protein glycosyltra
569008633	XP_006527629.1#Ddx3x#13205# PREDICTED: ATP-dependent RNA helicase DDX3X i
7305031	NP_038841.1#Epb4.1l3#13823# band 4.1-like protein 3 [Mus musculus]

31543940	NP_062780.2#Vapb#56491# vesicle-associated membrane protein-associated prote
6754910	NP_035078.1#Nudc#18221# nuclear migration protein nudC [Mus musculus]
111494223	NP_780540.2#Nt5dc3#103466# 5'-nucleotidase domain-containing protein 3 [Mus r
9789995	NP_062615.1#Stmn1#16765# stathmin [Mus musculus]
568986774	XP_006518644.1#Hnrnpc#15381# PREDICTED: heterogeneous nuclear ribonucleopr
568935657	XP_006535014.1#Mapk10#26414# PREDICTED: mitogen-activated protein kinase 1(
134288917	NP_084514.2#Dync1h1#13424# cytoplasmic dynein 1 heavy chain 1 [Mus musculus
6680139	NP_032220.1#Gk#14933# glycerol kinase isoform 1 [Mus musculus]
568974718	XP_006533748.1#Mpp2#50997# PREDICTED: MAGUK p55 subfamily member 2 isof
568915799	XP_006498986.1#Pde1a#18573# PREDICTED: calcium/calmodulin-dependent 3',5'-c
27754103	NP_080235.2#Psmc6#67089# 26S protease regulatory subunit 10B [Mus musculus]
755496301	XP_011237210.1#Eprs#107508# PREDICTED: bifunctional glutamate/proline--tRNA
568929156	XP_006503181.1#Ncdn#26562# PREDICTED: neurochondrin isoform X2 [Mus muscu
755506225	XP_011248279.1#Cdk5rap2#214444# PREDICTED: CDK5 regulatory subunit-associat
46593021	NP_079683.2#Uqcrc1#22273# cytochrome b-c1 complex subunit 1, mitochondrial p
6753510	NP_034077.1#Cpne6#12891# copine-6 isoform a [Mus musculus]
46195430	NP_659119.2#Ndufs8#225887# NADH dehydrogenase [ubiquinone] iron-sulfur prot
6671569	NP_031501.1#Rplp0#11837# 60S acidic ribosomal protein P0 [Mus musculus]
568966705	XP_006513298.1#Gnaz#14687# PREDICTED: guanine nucleotide-binding protein G(a
30519943	NP_848729.1#Samm50#68653# sorting and assembly machinery component 50 hoi
28077013	NP_082810.1#Smap1#98366# stromal membrane-associated protein 1 isoform 1 [N
569005645	XP_006531742.1#Pcx#18563# PREDICTED: pyruvate carboxylase, mitochondrial isof
568964960	XP_006512583.1#Epb4.1l2#13822# PREDICTED: band 4.1-like protein 2 isoform X12
45598372	NP_081671.1#Basp1#70350# brain acid soluble protein 1 [Mus musculus]
160707894	NP_033788.3#Akr1b3#11677# aldose reductase [Mus musculus]
18700024	NP_570954.1#Idh3b#170718# isocitrate dehydrogenase [NAD] subunit beta, mitocl
167716841	YP_001686702.1#ATP8#5912284# ATP synthase F0 subunit 8 [Mus musculus muscu
8394027	NP_058587.1#Ppp2r1a#51792# serine/threonine-protein phosphatase 2A 65 kDa re
116089273	NP_032138.3#Gdi2#14569# rab GDP dissociation inhibitor beta [Mus musculus]
568940123	XP_006505342.1#Aldh1l1#107747# PREDICTED: cytosolic 10-formyltetrahydrofolat
238637279	NP_032603.3#Slc3a2#17254# 4F2 cell-surface antigen heavy chain isoform b [Mus r
568999766	XP_006524083.1#Fbxl16#214931# PREDICTED: F-box/LRR-repeat protein 16 isoforn
568971697	XP_006532308.1#Guk1#14923# PREDICTED: guanylate kinase isoform X1 [Mus mus
6753320	NP_033966.1#Cct3#12462# T-complex protein 1 subunit gamma [Mus musculus]
45433560	NP_032475.2#Kif5c#16574# kinesin heavy chain isoform 5C [Mus musculus]
755509987	XP_006539192.2#Tprgl#67808# PREDICTED: tumor protein p63-regulated gene 1-lik
6681273	NP_031932.1#Eef1a2#13628# elongation factor 1-alpha 2 [Mus musculus]
31543971	NP_062635.2#Ykt6#56418# synaptobrevin homolog YKT6 [Mus musculus]
13385536	NP_080310.1#Armc10#67211# armadillo repeat-containing protein 10 [Mus muscu
112181194	NP_035165.2#Pak1#18479# serine/threonine-protein kinase PAK 1 [Mus musculus]
46049022	NP_079826.2#Psmd6#66413# 26S proteasome non-ATPase regulatory subunit 6 [M
112363072	NP_083987.1#Arpc2#76709# actin-related protein 2/3 complex subunit 2 [Mus mus
6678359	NP_033414.1#Tkt#21881# transketolase [Mus musculus]
13385554	NP_080332.1#Cap2#67252# adenylyl cyclase-associated protein 2 [Mus musculus]
256773295	NP_001157715.1#Prpsap2#212627# phosphoribosyl pyrophosphate synthase-assoc
240120054	NP_079613.3#Akr7a5#110198# aflatoxin B1 aldehyde reductase member 2 [Mus m

569019198	XP_006537191.1#Dlg4#13385# PREDICTED: disks large homolog 4 isoform X8 [Mus
568909147	XP_006529690.1#Dpp10#269109# PREDICTED: inactive dipeptidyl peptidase 10 isof
13385942	NP_080720.1#Cs#12974# citrate synthase, mitochondrial precursor [Mus musculus]
226453485	YP_002791052.1#ND5#7668600# NADH dehydrogenase subunit 5 [Mus musculus c
755539074	XP_011247425.1#Nt5c#50773# PREDICTED: 5'(3')-deoxyribonucleotidase, cytosolic
255522823	NP_775278.3#Tbc1d24#224617# TBC1 domain family member 24 isoform b [Mus m
568960787	XP_006510895.1#Myo5a#17918# PREDICTED: unconventional myosin-Va isoform X
68226731	NP_032968.2#Npepps#19155# puromycin-sensitive aminopeptidase [Mus musculus
40068507	NP_031791.3#Crmp1#12933# dihydropyrimidinase-related protein 1 isoform 2 [Mu
291045426	NP_001166977.1#Map1a#17754# microtubule-associated protein 1A isoform 2 [Mu
755544665	XP_011242675.1#Sfxn1#14057# PREDICTED: sideroflexin-1 isoform X2 [Mus muscul
344217717	NP_001230686.1#Pde2a#207728# cGMP-dependent 3',5'-cyclic phosphodiesterase
160333216	NP_001103970.1#Canx#12330# calnexin precursor [Mus musculus]
568942796	XP_006506634.1#Agk#69923# PREDICTED: acylglycerol kinase, mitochondrial isofor
183396771	NP_034607.3#Hspd1#15510# 60 kDa heat shock protein, mitochondrial [Mus muscu
568985464	XP_006518099.1#Cadps#27062# PREDICTED: calcium-dependent secretion activato
148747410	NP_666097.3#Arcn1#213827# coatomer subunit delta [Mus musculus]
444299620	NP_001263245.1#Uba1#22201# ubiquitin-like modifier-activating enzyme 1 isoform
148747526	NP_033031.2#Rab7#19349# ras-related protein Rab-7a [Mus musculus]
254540168	NP_001156906.1#Hspa5#14828# 78 kDa glucose-regulated protein precursor [Mus
6678437	NP_033455.1#Tpt1#22070# translationally-controlled tumor protein [Mus musculus
568968863	XP_006514333.1#Ndufs7#75406# PREDICTED: NADH dehydrogenase [ubiquinone] i
84794597	NP_077779.2#Ppp3r1#19058# calcineurin subunit B type 1 [Mus musculus]
23956084	NP_059062.1#Acadvl#11370# very long-chain specific acyl-CoA dehydrogenase, mit
93102415	NP_034386.2#Gart#14450# trifunctional purine biosynthetic protein adenosine-3 [
33859751	NP_077774.1#Rab21#216344# ras-related protein Rab-21 [Mus musculus]
568915235	XP_006498719.1#Ckmt1#12716# PREDICTED: creatine kinase U-type, mitochondria
42415473	NP_032939.1#Ppp3ca#19055# serine/threonine-protein phosphatase 2B catalytic s
29893569	NP_775539.1#Eif5#217869# eukaryotic translation initiation factor 5 [Mus musculu
6680908	NP_031694.1#Cdk5#12568# cyclin-dependent kinase 5 [Mus musculus]
755553212	XP_011244136.1#Kpna1#16646# PREDICTED: importin subunit alpha-5 isoform X2 [
158635979	NP_001103610.1#Atp2a2#11938# sarcoplasmic/endoplasmic reticulum calcium AT
148747558	NP_035693.3#Prdx2#21672# peroxiredoxin-2 [Mus musculus]
162461907	NP_034611.2#Hspa9#15526# stress-70 protein, mitochondrial [Mus musculus]
148747424	NP_031476.3#Slc25a4#11739# ADP/ATP translocase 1 [Mus musculus]
116174793	NP_115999.2#Sptbn4#80297# spectrin beta chain, non-erythrocytic 4 isoform sigm
568910562	XP_006496765.1#Pea15a#18611# PREDICTED: astrocytic phosphoprotein PEA-15 is
6755566	NP_036160.1#Slc9a3r1#26941# Na(+)/H(+) exchange regulatory cofactor NHE-RF1 [
9790019	NP_062708.1#Asah1#11886# acid ceramidase precursor [Mus musculus]
312433957	NP_001186066.1#Macf1#11426# microtubule-actin cross-linking factor 1 isoform 2
568921291	XP_006500830.1#Hspa4l#18415# PREDICTED: heat shock 70 kDa protein 4L isoform
568981400	XP_006516550.1#Pfkp#56421# PREDICTED: ATP-dependent 6-phosphofructokinase
568951405	XP_006508268.1#Pgm2l1#70974# PREDICTED: glucose 1,6-bisphosphate synthase i
311771690	NP_001185720.1#Ak1#11636# adenylate kinase isoenzyme 1 isoform 2 [Mus muscu
755565294	XP_011245855.1#Rps4x#20102# PREDICTED: 40S ribosomal protein S4, X isoform is
31560656	NP_032800.2#Pabpc1#18458# polyadenylate-binding protein 1 [Mus musculus]

568922710	XP_006501503.1#Ak5#229949# PREDICTED: adenylate kinase isoenzyme 5 isoform
6755114	NP_036151.1#Prdx5#54683# peroxiredoxin-5, mitochondrial precursor [Mus musc
568960899	XP_006510947.1#Nptn#20320# PREDICTED: neuroplastin isoform X2 [Mus muscul
11528518	NP_071313.1#Sv2a#64051# synaptic vesicle glycoprotein 2A [Mus musculus]
23956214	NP_076092.1#Sfpq#71514# splicing factor, proline- and glutamine-rich [Mus musc
7305521	NP_038789.1#Srr#27364# serine racemase [Mus musculus]
30794138	NP_082568.1#Ppme1#72590# protein phosphatase methylesterase 1 [Mus muscul
61888838	NP_058043.3#Hsd17b10#15108# 3-hydroxyacyl-CoA dehydrogenase type-2 [Mus m
42415475	NP_035162.1#P4hb#18453# protein disulfide-isomerase precursor [Mus musculus]
7305581	NP_038923.1#Timm13#30055# mitochondrial import inner membrane translocase
755497436	XP_011237380.1#Sh3glb2#227700# PREDICTED: endophilin-B2 isoform X6 [Mus mu
803378413	NP_001292773.1#Apoe#11816# apolipoprotein E precursor [Mus musculus]
152	TRYP_PIG
161086902	NP_001104316.1#Cacna2d1#12293# voltage-dependent calcium channel subunit al
325910859	NP_001191804.1#Set#56086# protein SET isoform 2 [Mus musculus]
568911813	XP_006497279.1#Rab3gap2#98732# PREDICTED: rab3 GTPase-activating protein nc
568946338	XP_006540318.1#Actn4#60595# PREDICTED: alpha-actinin-4 isoform X2 [Mus musc
568994470	XP_006521784.1#Atp6v1a#11964# PREDICTED: V-type proton ATPase catalytic sub
18079339	NP_542364.1#Aco2#11429# aconitate hydratase, mitochondrial precursor [Mus mu
6753324	NP_033968.1#Cct6a#12466# T-complex protein 1 subunit zeta [Mus musculus]
110625902	NP_082293.1#Napg#108123# gamma-soluble NSF attachment protein [Mus muscul
755519194	XP_011248758.1#Pld3#18807# PREDICTED: phospholipase D3 isoform X3 [Mus mus
357394934	NP_001239447.1#Nono#53610# non-POU domain-containing octamer-binding prot
254281331	NP_061349.3#Slc1a4#55963# neutral amino acid transporter A [Mus musculus]
111038118	NP_032238.2#Hadh#15107# hydroxyacyl-coenzyme A dehydrogenase, mitochondri
34098931	NP_031612.1#Calb2#12308# calretinin [Mus musculus]
19527258	NP_598803.1#Aldh6a1#104776# methylmalonate-semialdehyde dehydrogenase [a
569009753	XP_006527907.1#Idh3g#15929# PREDICTED: isocitrate dehydrogenase [NAD] subur
110625948	NP_694791.2#Acat3#224530# acetyl-Coenzyme A acetyltransferase 3 [Mus muscul
31542413	NP_035909.2#Coro1c#23790# coronin-1C [Mus musculus]
254540027	NP_032644.3#Mdh1#17449# malate dehydrogenase, cytoplasmic [Mus musculus]
10946574	NP_067248.1#Ckb#12709# creatine kinase B-type [Mus musculus]
31982393	NP_031966.2#Ephx2#13850# bifunctional epoxide hydrolase 2 isoform a [Mus mus
6754254	NP_034610.1#Hsp90aa1#15519# heat shock protein HSP 90-alpha [Mus musculus]
14389431	NP_058017.1#Stip1#20867# stress-induced-phosphoprotein 1 [Mus musculus]
597517936	NP_001277985.1#Snap25#20614# synaptosomal-associated protein 25 isoform b [
359279952	NP_001240685.1#Gpm6a#234267# neuronal membrane glycoprotein M6-a isoforn
22267442	NP_080175.1#Uqcrc2#67003# cytochrome b-c1 complex subunit 2, mitochondrial
568987554	XP_006519014.1#Ogdhl#239017# PREDICTED: 2-oxoglutarate dehydrogenase-like,
568968072	XP_006513956.1#Nap1l1#53605# PREDICTED: nucleosome assembly protein 1-like
18250284	NP_083849.1#Idh3a#67834# isocitrate dehydrogenase [NAD] subunit alpha, mitoc
755554060	XP_011244319.1#Bdh1#71911# PREDICTED: D-beta-hydroxybutyrate dehydrogena
6754004	NP_034431.1#Gna11#14672# guanine nucleotide-binding protein subunit alpha-11
568906168	XP_006495948.1#Tpp2#22019# PREDICTED: tripeptidyl-peptidase 2 isoform X1 [Mu
9790055	NP_062732.1#Mtch2#56428# mitochondrial carrier homolog 2 [Mus musculus]
14192922	NP_033738.1#Actc1#11464# actin, alpha cardiac muscle 1 [Mus musculus]

266458391	NP_067259.4#Kras#16653# GTPase KRas [Mus musculus]
568904643	XP_006544594.1#LOC102642619#102642619# PREDICTED: peroxiredoxin-6 [Mus m
568952615	XP_006508536.1#Cttn#13043# PREDICTED: src substrate cortactin isoform X1 [Mus
126723336	NP_031557.2#Phb2#12034# prohibitin-2 [Mus musculus]
568994456	XP_006521777.1#Alcam#11658# PREDICTED: CD166 antigen isoform X1 [Mus musc
31980648	NP_058054.2#Atp5b#11947# ATP synthase subunit beta, mitochondrial precursor [
160298209	NP_034454.2#Got1#14718# aspartate aminotransferase, cytoplasmic [Mus muscul
21489933	NP_036082.1#Mapk3#26417# mitogen-activated protein kinase 3 [Mus musculus]
6753322	NP_033967.1#Cct4#12464# T-complex protein 1 subunit delta [Mus musculus]
33859662	NP_036167.1#Vat1#26949# synaptic vesicle membrane protein VAT-1 homolog [M
225579033	NP_766599.2#Idh2#269951# isocitrate dehydrogenase [NADP], mitochondrial prec
167716840	YP_001686701.1#COX2#5912281# cytochrome c oxidase subunit II [Mus musculus
260166642	NP_001159374.1#Ctnnb1#12387# catenin beta-1 [Mus musculus]
93102409	NP_032014.3#Fasn#14104# fatty acid synthase [Mus musculus]
110625624	NP_038714.2#Tcp1#21454# T-complex protein 1 subunit alpha isoform 1 [Mus mus
22550094	NP_032950.1#Prkar2a#19087# cAMP-dependent protein kinase type II-alpha regula
13386054	NP_080828.1#Arpc4#68089# actin-related protein 2/3 complex subunit 4 isoform 1
33859482	NP_031933.1#Eef2#13629# elongation factor 2 [Mus musculus]
568999008	XP_006523719.1#Flot1#14251# PREDICTED: flotillin-1 isoform X1 [Mus musculus]
13385322	NP_080119.1#Ndufb7#66916# NADH dehydrogenase [ubiquinone] 1 beta subcomp
45504359	NP_031536.2#Atp6v1e1#11973# V-type proton ATPase subunit E 1 [Mus musculus]
568904377	XP_006544804.1#LOC102642137#102642137# PREDICTED: 40S ribosomal protein S
9789997	NP_062668.1#Letm1#56384# LETM1 and EF-hand domain-containing protein 1, mit
9789991	NP_062631.1#Hsd17b12#56348# very-long-chain 3-oxoacyl-CoA reductase [Mus m
126521835	NP_031662.2#Cct2#12461# T-complex protein 1 subunit beta [Mus musculus]
545746274	NP_001271118.1#Homer1#26556# homer protein homolog 1 isoform b [Mus musc
7106439	NP_035785.1#Tubb5#22154# tubulin beta-5 chain [Mus musculus]
568979363	XP_006515792.1#Ahsa1#217737# PREDICTED: activator of 90 kDa heat shock prote
253735731	NP_001156715.1#Fis1#66437# mitochondrial fission 1 protein isoform 2 [Mus musc
13507622	NP_109617.1#Sacm1l#83493# phosphatidylinositide phosphatase SAC1 [Mus musc
568991661	XP_006520649.1#Pcbp2#18521# PREDICTED: poly(rC)-binding protein 2 isoform X1
239051082	NP_001155090.1#Cul5#75717# cullin-5 isoform 2 [Mus musculus]
27369581	NP_766024.1#Slc25a12#78830# calcium-binding mitochondrial carrier protein Aral
13928670	NP_075373.1#Vps35#65114# vacuolar protein sorting-associated protein 35 [Mus r
226442759	NP_001140159.1#Pip5k1c#18717# phosphatidylinositol 4-phosphate 5-kinase type-
56682931	NP_001003899.1#Tardbp#230908# TAR DNA-binding protein 43 isoform 3 [Mus mu
568965829	XP_006512996.1#Wasf1#83767# PREDICTED: wiskott-Aldrich syndrome protein fan
159110562	NP_032345.2#Icam5#15898# intercellular adhesion molecule 5 precursor [Mus mus
295054266	NP_001171138.1#Sptan1#20740# spectrin alpha chain, non-erythrocytic 1 isoform
33859640	NP_035658.1#Taldo1#21351# transaldolase [Mus musculus]
568939485	XP_006505081.1#Ptprz1#19283# PREDICTED: receptor-type tyrosine-protein phosp
568944716	XP_006539535.1#Slc8a2#110891# PREDICTED: sodium/calcium exchanger 2 isoforn
568960677	XP_006510842.1#Anp32a#11737# PREDICTED: acidic leucine-rich nuclear phospho
13385854	NP_080628.1#Ppid#67738# peptidyl-prolyl cis-trans isomerase D [Mus musculus]
58037267	NP_082235.1#Pdia6#71853# protein disulfide-isomerase A6 precursor [Mus muscul
31981304	NP_038505.2#Atp6v0d1#11972# V-type proton ATPase subunit d 1 [Mus musculus]

283945579	NP_001152987.1#H2afy#26914# core histone macro-H2A.1 isoform 4 [Mus muscul
568967017	XP_006513447.1#Rab5b#19344# PREDICTED: ras-related protein Rab-5B isoform X1
18390323	NP_080973.1#Rab14#68365# ras-related protein Rab-14 [Mus musculus]
568991693	XP_006520665.1#Pfkm#18642# PREDICTED: ATP-dependent 6-phosphofructokinase
13385976	NP_080766.1#Mrpl19#56284# 39S ribosomal protein L19, mitochondrial [Mus mus
31982186	NP_032643.2#Mdh2#17448# malate dehydrogenase, mitochondrial precursor [Mus
568930614	XP_006538626.1#Hmgcl#15356# PREDICTED: hydroxymethylglutaryl-CoA lyase, mit
84370256	NP_598530.2#Cyfip2#76884# cytoplasmic FMR1-interacting protein 2 [Mus muscul
94721328	NP_038961.2#Vapa#30960# vesicle-associated membrane protein-associated prote
163838648	NP_001106209.1#Atp5c1#11949# ATP synthase subunit gamma, mitochondrial isof
50053703	NP_758465.2#Ppp1r9b#217124# neurabin-2 [Mus musculus]
46195798	NP_031864.2#Ddost#13200# dolichyl-diphosphooligosaccharide--protein glycosyltr
568916013	XP_006499092.1#Scn1a#20265# PREDICTED: sodium channel protein type 1 subuni
27552760	NP_613065.2#Tomm70a#28185# mitochondrial import receptor subunit TOM70 [M
6680027	NP_032159.1#Glud1#14661# glutamate dehydrogenase 1, mitochondrial precursor
568921751	XP_006501042.1#Dclk1#13175# PREDICTED: serine/threonine-protein kinase DCLK1
568903782	XP_006543536.1#LOC102642689#102642689# PREDICTED: 40S ribosomal protein S
19482160	NP_082347.1#Cotl1#72042# coactosin-like protein [Mus musculus]
262118191	NP_766520.2#Nlgn3#245537# neuroligin-3 precursor [Mus musculus]
114326446	NP_071855.2#Myh9#17886# myosin-9 [Mus musculus]
21312036	NP_081806.1#Rufy3#52822# protein RUFY3 isoform 4 [Mus musculus]
21312314	NP_082483.1#Dusp3#72349# dual specificity protein phosphatase 3 [Mus musculus
27370092	NP_766333.1#Tufm#233870# elongation factor Tu, mitochondrial isoform 1 [Mus m
6755372	NP_036182.1#Rps3#27050# 40S ribosomal protein S3 [Mus musculus]
47059486	NP_064667.2#Pdxp#57028# pyridoxal phosphate phosphatase [Mus musculus]
755530066	XP_006511148.2#Dmxl2#235380# PREDICTED: dmX-like protein 2 isoform X3 [Mus
568940591	XP_006505567.1#Eno2#13807# PREDICTED: gamma-enolase isoform X1 [Mus musc
6678674	NP_032518.1#Ldhb#16832# L-lactate dehydrogenase B chain isoform 1 [Mus muscu
22122825	NP_666355.1#Actr2#66713# actin-related protein 2 [Mus musculus]
254692859	NP_079634.2#Ndufa9#66108# NADH dehydrogenase [ubiquinone] 1 alpha subcom
568973007	XP_006532937.1#Arhgap44#216831# PREDICTED: rho GTPase-activating protein 44
755544221	XP_011242606.1#Amph#218038# PREDICTED: amphiphysin isoform X1 [Mus muscu
568921739	XP_006501036.1#Cryz#12972# PREDICTED: quinone oxidoreductase isoform X1 [Mu
31088914	NP_852063.1#Anks1b#77531# ankyrin repeat and sterile alpha motif domain-conta
54292132	NP_803155.1#Psat1#107272# phosphoserine aminotransferase isoform 1 [Mus mu
116256510	NP_001070732.1#Ap2a1#11771# AP-2 complex subunit alpha-1 isoform b [Mus mu
6755040	NP_035202.1#Pfn1#18643# profilin-1 [Mus musculus]
568986628	XP_006518571.1#Dpysl2#12934# PREDICTED: dihydropyrimidinase-related protein
568962941	XP_006511785.1#Camkv#235604# PREDICTED: caM kinase-like vesicle-associated p
19527228	NP_598768.1#Cisd1#52637# CDGSH iron-sulfur domain-containing protein 1 [Mus r
755497126	XP_006497717.2#Dnm1#13429# PREDICTED: dynamin-1 isoform X6 [Mus musculus
568934035	XP_006503926.1#Wdr1#22388# PREDICTED: WD repeat-containing protein 1 isofor
294774562	NP_001171030.1#Gng12#14701# guanine nucleotide-binding protein G(I)/G(S)/G(C
113204613	NP_034967.2#Map6#17760# microtubule-associated protein 6 isoform 1 [Mus mus
6679261	NP_032836.1#Pdha1#18597# pyruvate dehydrogenase E1 component subunit alph
157823795	NP_001102512.1#Kif21a#16564# kinesin-like protein KIF21A isoform 4 [Mus muscu

568974806	XP_006533792.1#Cntnap1#53321# PREDICTED: contactin-associated protein 1 isofc
13385168	NP_079986.1#Uqcrfs1#66694# cytochrome b-c1 complex subunit Rieske, mitochon
255003735	NP_035417.2#Rpl10a#19896# 60S ribosomal protein L10a [Mus musculus]
568961539	XP_006511259.1#Map2k1#26395# PREDICTED: dual specificity mitogen-activated p
568965113	XP_006512658.1#Pcmt1#18537# PREDICTED: protein-L-isoaspartate(D-aspartate) C
568931690	XP_006539140.1#Mtor#56717# PREDICTED: serine/threonine-protein kinase mTOR
7110703	NP_032976.1#Psmc5#19184# 26S protease regulatory subunit 8 [Mus musculus]
9938002	NP_064674.1#Lgi1#56839# leucine-rich glioma-inactivated protein 1 precursor [Mu
34328251	NP_062787.2#Dbn1#56320# drebrin isoform 3 [Mus musculus]
113199771	NP_034944.2#Mog#17441# myelin-oligodendrocyte glycoprotein precursor [Mus m
224922803	NP_034404.3#Gpd2#14571# glycerol-3-phosphate dehydrogenase, mitochondrial p
568939259	XP_006504978.1#Hsph1#15505# PREDICTED: heat shock protein 105 kDa isoform X
12746424	NP_075534.1#Dpysl5#65254# dihydropyrimidinase-related protein 5 [Mus musculu
9506971	NP_062283.1#Pfn2#18645# profilin-2 [Mus musculus]
33859560	NP_034403.1#Gdi1#14567# rab GDP dissociation inhibitor alpha [Mus musculus]
225637531	NP_079620.2#Eif3f#66085# eukaryotic translation initiation factor 3 subunit F [Mus
157951727	NP_663785.2#Ctnna2#12386# catenin alpha-2 isoform 1 [Mus musculus]
84490421	NP_001033726.1#Gng2#14702# guanine nucleotide-binding protein G(I)/G(S)/G(O)
755522937	XP_011240168.1#Stx1b#56216# PREDICTED: syntaxin-1B isoform X2 [Mus musculus
31560686	NP_032327.2#Hspa2#15512# heat shock-related 70 kDa protein 2 [Mus musculus]
31980942	NP_061352.2#Impa1#55980# inositol monophosphatase 1 [Mus musculus]
146219837	NP_034253.3#Eif3a#13669# eukaryotic translation initiation factor 3 subunit A [Mu
755522634	XP_011240088.1#Dlg2#23859# PREDICTED: disks large homolog 2 isoform X22 [Mu
238624114	NP_598949.3#Acot2#171210# acyl-coenzyme A thioesterase 2, mitochondrial precu
22122625	NP_666220.1#Hibch#227095# 3-hydroxyisobutyryl-CoA hydrolase, mitochondrial pr
755543957	XP_011242554.1#Wdr37#207615# PREDICTED: WD repeat-containing protein 37 iso
755516671	XP_011239634.1#Mgll#23945# PREDICTED: monoglyceride lipase isoform X1 [Mus m
568994656	XP_006521871.1#Cd47#16423# PREDICTED: leukocyte surface antigen CD47 isoforr
18017605	NP_542127.1#Ube2n#93765# ubiquitin-conjugating enzyme E2 N [Mus musculus]
755513560	XP_011246490.1#Rph3a#19894# PREDICTED: rabphilin-3A isoform X2 [Mus musculu
294997259	NP_001171128.1#Grin1#14810# glutamate receptor ionotropic, NMDA 1 isoform 3
755497651	XP_011237415.1#Scai#320271# PREDICTED: protein SCAI isoform X1 [Mus musculu
6679299	NP_032857.1#Phb#18673# prohibitin [Mus musculus]
149273202	XP_001476757.1#Gm20899#100042025# PREDICTED: glyceraldehyde-3-phosphate
214010170	NP_038520.2#Clu#12759# clusterin precursor [Mus musculus]
28076935	NP_081427.1#Dctn2#69654# dynactin subunit 2 isoform 3 [Mus musculus]
31980939	NP_080719.2#Mtfp1#67900# mitochondrial fission process protein 1 [Mus musculu
19526912	NP_598487.1#St13#70356# hsc70-interacting protein [Mus musculus]
568908350	XP_006529301.1#Ncl#17975# PREDICTED: nucleolin isoform X1 [Mus musculus]
549806742	NP_001272357.1#Cacnb4#12298# voltage-dependent L-type calcium channel subu
27229101	NP_081740.2#Fam213a#70564# redox-regulatory protein FAM213A [Mus musculus
13385384	NP_080170.1#Psmd12#66997# 26S proteasome non-ATPase regulatory subunit 12
6753556	NP_034113.1#Ctsd#13033# cathepsin D precursor [Mus musculus]
340007377	NP_001229979.1#Atp6v0a1#11975# V-type proton ATPase 116 kDa subunit a isofo
568961798	XP_006511383.1#Scamp5#56807# PREDICTED: secretory carrier-associated membr
27923929	NP_778163.1#Hpcal4#170638# hippocalcin-like protein 4 [Mus musculus]

568999382	XP_006523896.1#Prkce#18754# PREDICTED: protein kinase C epsilon type isoform
46849708	NP_035636.1#Sucla2#20916# succinyl-CoA ligase [ADP-forming] subunit beta, mito
755498668	XP_011237583.1#Epb4.1l1#13821# PREDICTED: band 4.1-like protein 1 isoform X8
7106257	NP_033857.1#Atp8a1#11980# phospholipid-transporting ATPase IA isoform b [Mus
6680748	NP_031531.1#Atp5a1#11946# ATP synthase subunit alpha, mitochondrial precurso
13277394	NP_077798.1#Grpel1#17713# grpE protein homolog 1, mitochondrial precursor [M
442535508	NP_001259026.1#Fabp5#16592# fatty acid-binding protein, epidermal isoform 2 [
568907993	XP_006529127.1#Cops8#108679# PREDICTED: COP9 signalosome complex subunit
8393866	NP_058674.1#Oat#18242# ornithine aminotransferase, mitochondrial precursor [
755509924	XP_011248613.1#Park7#57320# PREDICTED: protein DJ-1 isoform X1 [Mus musculu
31981690	NP_112442.2#Hspa8#15481# heat shock cognate 71 kDa protein [Mus musculus]
124248577	NP_036004.2#Psmc4#23996# 26S protease regulatory subunit 6B [Mus musculus]
19526818	NP_598429.1#Slc25a3#18674# phosphate carrier protein, mitochondrial precursor
13385472	NP_080250.1#Rpl14#67115# 60S ribosomal protein L14 [Mus musculus]
568959251	XP_006510273.1#Ntm#235106# PREDICTED: neurotrimin isoform X6 [Mus musculu
31560697	NP_032223.2#H1f0#14958# histone H1.0 [Mus musculus]
60687506	NP_033787.2#Aldoc#11676# fructose-bisphosphate aldolase C [Mus musculus]
755498690	XP_011237589.1#Elmo2#140579# PREDICTED: engulfment and cell motility protein
568967045	XP_006513461.1#Sar1a#20224# PREDICTED: GTP-binding protein SAR1a isoform X1
568984240	XP_006517602.1#Kif2a#16563# PREDICTED: kinesin-like protein KIF2A isoform X6 [
27229051	NP_080543.2#Necap1#67602# adaptin ear-binding coat-associated protein 1 [Mus
12963569	NP_075689.1#Ppp1r7#66385# protein phosphatase 1 regulatory subunit 7 [Mus m
568889869	XP_006543820.1#LOC102641678#102641678# PREDICTED: ATP synthase subunit O
31981269	NP_075629.2#Glrx3#30926# glutaredoxin-3 [Mus musculus]
6671672	NP_031630.1#Capza2#12343# F-actin-capping protein subunit alpha-2 [Mus muscu
568915813	XP_006498993.1#Plcb1#18795# PREDICTED: 1-phosphatidylinositol 4,5-bisphospha
31560737	NP_031448.2#Adss#11566# adenylosuccinate synthetase isozyme 2 [Mus musculus
239985643	NP_001155219.1#Palm#18483# paralemmin-1 isoform 2 [Mus musculus]
755560613	XP_011245162.1#Pura#19290# PREDICTED: transcriptional activator protein Pur-al
755521369	XP_011249216.1#Ppfia3#76787# PREDICTED: liprin-alpha-3 isoform X2 [Mus muscu
568971592	XP_006532257.1#Flot2#14252# PREDICTED: flotillin-2 isoform X3 [Mus musculus]
6671509	NP_031419.1#Actb#11461# actin, cytoplasmic 1 [Mus musculus]
58037117	NP_080964.1#Ndufs3#68349# NADH dehydrogenase [ubiquinone] iron-sulfur prote
339895916	NP_001229973.1#Ap1b1#11764# AP-1 complex subunit beta-1 isoform 3 [Mus mus
568930664	XP_006538651.1#Kcnab2#16498# PREDICTED: voltage-gated potassium channel su
71774133	NP_035279.2#Ppib#19035# peptidyl-prolyl cis-trans isomerase B precursor [Mus m
110625954	NP_082664.1#Ndufv2#72900# NADH dehydrogenase [ubiquinone] flavoprotein 2,
158508501	NP_998779.2#Sept5#18951# septin-5 [Mus musculus]
568972134	XP_006532520.1#Pdk2#18604# PREDICTED: pyruvate dehydrogenase kinase, isoen
568962761	XP_006511700.1#Gnai2#14678# PREDICTED: guanine nucleotide-binding protein G
755522217	XP_011239997.1#Prkcb#18751# PREDICTED: protein kinase C beta type isoform X2
117606375	NP_036019.2#Sept3#24050# neuronal-specific septin-3 [Mus musculus]
18266680	NP_077150.1#Oxct1#67041# succinyl-CoA:3-ketoacid coenzyme A transferase 1, mi
13385492	NP_080263.1#Ndufa6#67130# NADH dehydrogenase [ubiquinone] 1 alpha subcom
6756037	NP_035868.1#Ywhah#22629# 14-3-3 protein eta [Mus musculus]
10092608	NP_038569.1#Gstp1#14870# glutathione S-transferase P 1 [Mus musculus]

255069795	NP_035820.3#Vars#22321# valine--tRNA ligase [Mus musculus]
31980806	NP_058063.2#Pgrmc1#53328# membrane-associated progesterone receptor comp[illegible]
113680348	NP_032010.2#Fscn1#14086# fascin [Mus musculus]
6754524	NP_034829.1#Ldha#16828# L-lactate dehydrogenase A chain isoform 1 [Mus muscu[illegible]
568991032	XP_006520346.1#Cyb5r3#109754# PREDICTED: NADH-cytochrome b5 reductase 3 i[illegible]
667751602	NP_001288140.1#Ap1g1#11765# AP-1 complex subunit gamma-1 isoform 2 [Mus m[illegible]
7305631	NP_038869.1#Vps45#22365# vacuolar protein sorting-associated protein 45 [Mus m[illegible]
568956646	XP_006530987.1#Aars#234734# PREDICTED: alanine--tRNA ligase, cytoplasmic isof[illegible]
257796245	NP_663589.3#Dlat#235339# dihydrolipoyllysine-residue acetyltransferase compone[illegible]
568966884	XP_006513387.1#Myl6#17904# PREDICTED: myosin light polypeptide 6 isoform X5
6753138	NP_033851.1#Atp1b1#11931# sodium/potassium-transporting ATPase subunit bet[illegible]
568975126	XP_006533946.1#Hnrnph1#59013# PREDICTED: heterogeneous nuclear ribonucleo[illegible]
40556608	NP_032328.2#Hsp90ab1#15516# heat shock protein HSP 90-beta [Mus musculus]
31980762	NP_038699.2#Sod2#20656# superoxide dismutase [Mn], mitochondrial precursor [[illegible]
27369922	NP_766234.1#Dnm3#103967# dynamin-3 isoform 2 [Mus musculus]
70778976	NP_032854.2#Pgk1#18655# phosphoglycerate kinase 1 [Mus musculus]
63999380	NP_038615.2#Lrpap1#16976# alpha-2-macroglobulin receptor-associated protein p[illegible]
755539138	XP_006533808.2#Sept9#53860# PREDICTED: septin-9 isoform X1 [Mus musculus]
30519995	NP_848754.1#Sfxn5#94282# sideroflexin-5 [Mus musculus]
13385006	NP_079843.1#Cyc1#66445# cytochrome c1, heme protein, mitochondrial [Mus mus[illegible]
6679587	NP_033022.1#Rab1#19324# ras-related protein Rab-1A [Mus musculus]
568952416	XP_006536280.1#Cend1#57754# PREDICTED: cell cycle exit and neuronal differenti[illegible]
568983158	XP_006517219.1#Pdcd6#18570# PREDICTED: programmed cell death protein 6 isof[illegible]
13386338	NP_082988.1#Rap2b#74012# ras-related protein Rap-2b precursor [Mus musculus]
568968038	XP_006513939.1#Sgta#52551# PREDICTED: small glutamine-rich tetratricopeptide r[illegible]
568917832	XP_006499972.1#Ywhab#54401# PREDICTED: 14-3-3 protein beta/alpha isoform X1
215983062	NP_065603.2#Ehd3#57440# EH domain-containing protein 3 [Mus musculus]
755495449	XP_011237059.1#Igsf8#140559# PREDICTED: immunoglobulin superfamily member
163644296	NP_081902.1#Psd3#234353# PH and SEC7 domain-containing protein 3 isoform 3 [[illegible]
568966266	XP_006513094.1#Lin7a#108030# PREDICTED: protein lin-7 homolog A isoform X1 [[illegible]
58037109	NP_080960.1#Ndufb10#68342# NADH dehydrogenase [ubiquinone] 1 beta subcom[illegible]
568936990	XP_006530290.1#Rpl6#19988# PREDICTED: 60S ribosomal protein L6 isoform X1 [[illegible]
28916677	NP_803126.1#Camk2a#12322# calcium/calmodulin-dependent protein kinase type
12963799	NP_076210.1#Atp6v1d#73834# V-type proton ATPase subunit D [Mus musculus]
84875526	NP_001034182.1#Tom1l2#216810# TOM1-like protein 2 isoform c [Mus musculus]
568967149	XP_006513512.1#Syt1#20979# PREDICTED: synaptotagmin-1 isoform X2 [Mus musc[illegible]
281182473	NP_001161943.1#Dynll2#68097# dynein light chain 2, cytoplasmic [Mus musculus]
51093840	NP_660121.2#Eif3l#223691# eukaryotic translation initiation factor 3 subunit L [Mu[illegible]
254939522	NP_001157095.1#Prepl#213760# prolyl endopeptidase-like isoform b [Mus muscul[illegible]
6755967	NP_035826.1#Vdac3#22335# voltage-dependent anion-selective channel protein 3
31980744	NP_038823.2#Atp5l#27425# ATP synthase subunit g, mitochondrial [Mus musculus[illegible]
6755963	NP_035824.1#Vdac1#22333# voltage-dependent anion-selective channel protein 1
100818462	NP_663519.2#Rap1gds1#229877# rap1 GTPase-GDP dissociation stimulator 1 isofo[illegible]
31982273	NP_032318.2#Hsd17b4#15488# peroxisomal multifunctional enzyme type 2 [Mus n[illegible]
568986366	XP_006518443.1#Phyhip#105653# PREDICTED: phytanoyl-CoA hydroxylase-interact[illegible]
224809382	NP_080645.2#Arpc5#67771# actin-related protein 2/3 complex subunit 5 [Mus mus[illegible]

18152793	NP_077183.1#Pdhb#68263# pyruvate dehydrogenase E1 component subunit beta,
145301549	NP_001077424.1#Hba-a2#110257# hemoglobin alpha, adult chain 2 [Mus musculus
568952448	XP_006536295.1#Slc25a22#68267# PREDICTED: mitochondrial glutamate carrier 1 i
134053913	NP_542121.2#Psmd5#66998# 26S proteasome non-ATPase regulatory subunit 5 [M
6755224	NP_036006.1#Twf2#23999# twinfilin-2 [Mus musculus]
329299065	NP_001192296.1#Sept7#235072# septin-7 isoform 2 [Mus musculus]
569019357	XP_006537268.1#Slc25a11#67863# PREDICTED: mitochondrial 2-oxoglutarate/mala
19882201	NP_598862.1#Psmd2#21762# 26S proteasome non-ATPase regulatory subunit 2 [M
126723461	NP_033970.3#Cct8#12469# T-complex protein 1 subunit theta [Mus musculus]
227500281	NP_663590.3#Etfa#110842# electron transfer flavoprotein subunit alpha, mitochon
37693505	NP_937760.1#Abi2#329165# abl interactor 2 isoform 3 [Mus musculus]
6996911	NP_031520.1#Ass1#11898# argininosuccinate synthase [Mus musculus]
283837783	NP_001025044.2#Unc13a#382018# protein unc-13 homolog A [Mus musculus]
755551082	XP_011243780.1#Oxr1#170719# PREDICTED: oxidation resistance protein 1 isoform
74271899	NP_034435.1#Gnai1#14677# guanine nucleotide-binding protein G(i) subunit alpha
327180707	NP_001192117.1#Ppp2r2a#71978# serine/threonine-protein phosphatase 2A 55 kD
6679583	NP_033023.1#Rab11b#19326# ras-related protein Rab-11B [Mus musculus]
6680954	NP_031753.1#Cntn1#12805# contactin-1 precursor [Mus musculus]
547235396	NP_001271257.1#Syncrip#56403# heterogeneous nuclear ribonucleoprotein Q isof
256773218	NP_001038980.1#Synj1#104015# synaptojanin-1 isoform b [Mus musculus]
568974067	XP_006533435.1#Map2k4#26398# PREDICTED: dual specificity mitogen-activated p
755561214	XP_011245294.1#Ccny#67974# PREDICTED: cyclin-Y isoform X2 [Mus musculus]
51511741	NP_001003933.1#Rtn3#20168# reticulon-3 isoform 2 [Mus musculus]
755530455	XP_011241115.1#Tln2#70549# PREDICTED: talin-2 isoform X4 [Mus musculus]
257153390	NP_001158057.1#Dnajc6#72685# putative tyrosine-protein phosphatase auxilin iso
229892320	NP_001153511.1#Ndufs1#227197# NADH-ubiquinone oxidoreductase 75 kDa subu
124486895	NP_001074743.1#Pgd#110208# 6-phosphogluconate dehydrogenase, decarboxylat
6753492	NP_034028.1#Coro1a#12721# coronin-1A isoform 1 [Mus musculus]
568990382	XP_006520096.1#Ctnnd2#18163# PREDICTED: catenin delta-2 isoform X2 [Mus mus
569008135	XP_006527556.1#Sfxn3#94280# PREDICTED: sideroflexin-3 isoform X1 [Mus muscul
31559891	NP_067511.4#Rhot1#59040# mitochondrial Rho GTPase 1 isoform 1 [Mus musculus
6754036	NP_034455.1#Got2#14719# aspartate aminotransferase, mitochondrial [Mus musc
33563266	NP_035016.1#Ndufa4#17992# cytochrome c oxidase subunit NDUFA4 [Mus muscul
594190942	NP_001277398.1#Atp1a3#232975# sodium/potassium-transporting ATPase subuni
255958286	NP_063932.2#Suclg1#56451# succinyl-CoA ligase [ADP/GDP-forming] subunit alpha
21314824	NP_079657.1#Atp6v1f#66144# V-type proton ATPase subunit F [Mus musculus]
6680690	NP_031478.1#Prdx3#11757# thioredoxin-dependent peroxide reductase, mitochon
649572215	NP_001280488.1#Cox4i1#12857# cytochrome c oxidase subunit 4 isoform 1, mitoc
755529755	XP_011240968.1#Me1#17436# PREDICTED: NADP-dependent malic enzyme isoform
755498625	XP_011237574.1#Csnk2a1#12995# PREDICTED: casein kinase II subunit alpha isofor
283806722	NP_001164339.1#Ncald#52589# neurocalcin-delta [Mus musculus]
24762230	NP_733769.1#Rps15a#267019# 40S ribosomal protein S15a [Mus musculus]
6754976	NP_035164.1#Prdx1#18477# peroxiredoxin-1 [Mus musculus]
31542143	NP_058082.2#Rhoa#11848# transforming protein RhoA precursor [Mus musculus]
568908602	XP_006529424.1#Cntn2#21367# PREDICTED: contactin-2 isoform X1 [Mus musculu
568945146	XP_006539743.1#Rps19#20085# PREDICTED: 40S ribosomal protein S19 isoform X1

19526960	NP_598513.1#Opa1#74143# dynamin-like 120 kDa protein, mitochondrial isoform
6680309	NP_032329.1#Hspe1#15528# 10 kDa heat shock protein, mitochondrial [Mus musc
568978634	XP_006515450.1#Actn1#109711# PREDICTED: alpha-actinin-1 isoform X4 [Mus mus
568995482	XP_006522265.1#Lsamp#268890# PREDICTED: limbic system-associated membrane
31560792	NP_062408.2#Sh3gl2#20404# endophilin-A1 [Mus musculus]
12963495	NP_075611.1#Gpm6b#14758# neuronal membrane glycoprotein M6-b isoform 4 [
34787412	NP_038914.2#Hdgfrp3#29877# hepatoma-derived growth factor-related protein 3
40254577	NP_035425.2#Rps12#20042# 40S ribosomal protein S12 [Mus musculus]
15011853	NP_058081.2#Stx1a#20907# syntaxin-1A [Mus musculus]
37718983	NP_937806.1#Rab35#77407# ras-related protein Rab-35 [Mus musculus]
28076897	NP_080914.1#Cdipt#52858# CDP-diacylglycerol--inositol 3-phosphatidyltransferase
21313640	NP_082191.1#Ap2b1#71770# AP-2 complex subunit beta isoform b [Mus musculus
755561255	XP_011245300.1#Afg3l2#69597# PREDICTED: AFG3-like protein 2 isoform X1 [Mus
568921574	XP_006500959.1#Ank2#109676# PREDICTED: ankyrin-2 isoform X25 [Mus musculu
218156282	NP_892038.2#Slc17a7#72961# vesicular glutamate transporter 1 [Mus musculus]
568966729	XP_006513310.1#Hk1#15275# PREDICTED: hexokinase-1 isoform X4 [Mus musculu
27228985	NP_079827.2#Ndufa12#66414# NADH dehydrogenase [ubiquinone] 1 alpha subcon
568969997	XP_006514639.1#Nipsnap1#18082# PREDICTED: protein NipSnap homolog 1 isofor
73695877	NP_808430.2#Aak1#269774# AP2 associated kinase 1 isoform 2 [Mus musculus]
13385726	NP_080495.1#Uqcrb#67530# cytochrome b-c1 complex subunit 7 [Mus musculus]
226874906	NP_033562.3#Ywhae#22627# 14-3-3 protein epsilon [Mus musculus]
755553155	XP_011244125.1#Dlg1#13383# PREDICTED: disks large homolog 1 isoform X14 [Mu
257467604	NP_780589.3#Gpd1l#333433# glycerol-3-phosphate dehydrogenase 1-like protein
7106387	NP_036097.1#Psma5#26442# proteasome subunit alpha type-5 [Mus musculus]
568974918	XP_006533846.1#Srcin1#56013# PREDICTED: SRC kinase signaling inhibitor 1 isofor
568902472	XP_006543439.1#LOC100862455#100862455# PREDICTED: 60S ribosomal protein L
226443015	NP_034303.3#Faah#14073# fatty-acid amide hydrolase 1 [Mus musculus]
568905907	XP_006495820.1#Map2#17756# PREDICTED: microtubule-associated protein 2 isof
21312062	NP_081051.1#Tmed10#68581# transmembrane emp24 domain-containing protein
226958349	NP_033441.2#Tpi1#21991# triosephosphate isomerase [Mus musculus]
568931876	XP_006539230.1#Acot7#70025# PREDICTED: cytosolic acyl coenzyme A thioester h
33598964	NP_780469.1#Myh10#77579# myosin-10 [Mus musculus]
755550334	XP_011243654.1#Ywhaz#22631# PREDICTED: 14-3-3 protein zeta/delta isoform X1
13386026	NP_080804.1#2700060E02Rik#68045# UPF0568 protein C14orf166 homolog [Mus
755530267	XP_011241071.1#Tmod2#50876# PREDICTED: tropomodulin-2 isoform X1 [Mus mu
357527382	NP_001239484.1#Gstz1#14874# maleylacetoacetate isomerase isoform 2 [Mus mu
21311873	NP_077181.1#Hsbp1#68196# heat shock factor-binding protein 1 [Mus musculus]
56711244	NP_035018.1#Ndufs6#407785# NADH dehydrogenase [ubiquinone] iron-sulfur prot
568926120	XP_006537702.1#Alad#17025# PREDICTED: delta-aminolevulinic acid dehydratase i
189339262	NP_079770.2#Atp6v1c1#66335# V-type proton ATPase subunit C 1 [Mus musculus]
227908823	NP_080471.2#Atic#108147# bifunctional purine biosynthesis protein PURH [Mus m
569009783	XP_006527922.1#L1cam#16728# PREDICTED: neural cell adhesion molecule L1 isof
26986569	NP_758475.1#Slc6a17#229706# sodium-dependent neutral amino acid transporter
17298686	NP_473438.1#Pip4k2c#117150# phosphatidylinositol 5-phosphate 4-kinase type-2
163644277	NP_031485.3#Ap2a2#11772# AP-2 complex subunit alpha-2 [Mus musculus]
568922992	XP_006501641.1#Negr1#320840# PREDICTED: neuronal growth regulator 1 isoform

254553458	NP_032181.2#Gpi1#14751# glucose-6-phosphate isomerase [Mus musculus]
51491845	NP_001003908.1#Cltc#67300# clathrin heavy chain 1 [Mus musculus]
569007769	XP_006527383.1#Hnrnpul2#68693# PREDICTED: heterogeneous nuclear ribonucleo
96975138	NP_038584.2#Hprt#15452# hypoxanthine-guanine phosphoribosyltransferase [Mus
22219434	NP_671734.1#Nrbp1#192292# nuclear receptor-binding protein [Mus musculus]
114326546	NP_075907.2#Pgam1#18648# phosphoglycerate mutase 1 [Mus musculus]
255759890	NP_001157572.1#Add3#27360# gamma-adducin isoform b [Mus musculus]
161086984	NP_941015.2#Ap2s1#232910# AP-2 complex subunit sigma [Mus musculus]
124494256	NP_032538.2#Lrp1#16971# prolow-density lipoprotein receptor-related protein 1 p
755530077	XP_006511157.2#Coro2b#235431# PREDICTED: coronin-2B isoform X1 [Mus muscu
134031976	NP_082509.2#Lrpprc#72416# leucine-rich PPR motif-containing protein, mitochond
568930415	XP_006538530.1#Atad3a#108888# PREDICTED: ATPase family AAA domain-contain
27369748	NP_766120.1#Aldh5a1#214579# succinate-semialdehyde dehydrogenase, mitochor
550544190	NP_001272385.1#Mapt#17762# microtubule-associated protein tau isoform e [Mus
6754724	NP_034947.1#Psmd7#17463# 26S proteasome non-ATPase regulatory subunit 7 [M
228480253	NP_001153190.1#Sept2#18000# septin-2 isoform b [Mus musculus]
6753364	NP_033991.1#Cdc42#12540# cell division control protein 42 homolog isoform 1 pre
110347469	NP_031402.3#Pzp#11287# alpha-2-macroglobulin precursor [Mus musculus]
226874869	NP_062282.2#Omg#18377# oligodendrocyte-myelin glycoprotein precursor [Mus m
113866024	NP_077776.2#Rab5c#19345# ras-related protein Rab-5C isoform 1 [Mus musculus]
568933539	XP_006503687.1#Mapre3#100732# PREDICTED: microtubule-associated protein RP
31982856	NP_031887.2#Dld#13382# dihydrolipoyl dehydrogenase, mitochondrial precursor [
754170607	NP_001291648.1#Atp5f1#11950# ATP synthase F(0) complex subunit B1, mitochon
6680924	NP_031713.1#Cfl1#12631# cofilin-1 [Mus musculus]
568980730	XP_006516455.1#Dlst#78920# PREDICTED: dihydrolipoyllysine-residue succinyltran
34328236	NP_061268.2#Ubqln2#54609# ubiquilin-2 [Mus musculus]
52353955	NP_058662.2#Phgdh#236539# D-3-phosphoglycerate dehydrogenase [Mus muscul
31559995	NP_598417.2#Crk#12928# adapter molecule crk isoform 2 [Mus musculus]
33468999	NP_081241.1#Comtd1#69156# catechol O-methyltransferase domain-containing pr
31982030	NP_598557.3#Arhgdia#192662# rho GDP-dissociation inhibitor 1 [Mus musculus]
568997209	XP_006522937.1#App#11820# PREDICTED: amyloid beta A4 protein isoform X2 [Mu
754170335	NP_001291503.1#Arf2#11841# ADP-ribosylation factor 2 [Mus musculus]
6679935	NP_032109.1#Gap43#14432# neuromodulin [Mus musculus]
569007565	XP_006527286.1#Arl3#56350# PREDICTED: ADP-ribosylation factor-like protein 3 is
356582426	NP_001239190.1#Npm1#18148# nucleophosmin isoform 3 [Mus musculus]
9790017	NP_062740.1#Ptges3#56351# prostaglandin E synthase 3 [Mus musculus]
731184185	NP_001289899.1#Ap2m1#11773# AP-2 complex subunit mu isoform 2 [Mus muscu
163792196	NP_058582.3#Gria3#53623# glutamate receptor 3 isoform a precursor [Mus muscu
312032384	NP_001185815.1#Dpp6#13483# dipeptidyl aminopeptidase-like protein 6 isoform 4
10181184	NP_065607.1#Atp5j2#57423# ATP synthase subunit f, mitochondrial [Mus musculus
84370353	NP_001033702.1#Olfm1#56177# noelin isoform c precursor [Mus musculus]
188219524	NP_036123.3#Dpysl4#26757# dihydropyrimidinase-related protein 4 [Mus musculu
568997213	XP_006522939.1#Atp5j#11957# PREDICTED: ATP synthase-coupling factor 6, mitoch
568938111	XP_006504490.1#Clip2#269713# PREDICTED: CAP-Gly domain-containing linker pro
85861220	NP_001034284.1#Gria2#14800# glutamate receptor 2 isoform 3 precursor [Mus mu
568896389	XP_006544502.1#LOC102641332#102641332# PREDICTED: GTP-binding nuclear pro

12963615	NP_075768.1#Tubb3#22152# tubulin beta-3 chain [Mus musculus]
6678347	NP_033408.1#Thy1#21838# thy-1 membrane glycoprotein preproprotein [Mus mus
550821975	NP_001272410.1#Hp1bp3#15441# heterochromatin protein 1-binding protein 3 iso
568942584	XP_006506531.1#Chchd3#66075# PREDICTED: MICOS complex subunit Mic19 isofo
45592934	NP_033033.1#Rac1#19353# ras-related C3 botulinum toxin substrate 1 precursor [
158966670	NP_001103681.1#Anxa6#11749# annexin A6 isoform b [Mus musculus]
29789104	NP_062606.1#Napb#17957# beta-soluble NSF attachment protein [Mus musculus]
6755196	NP_036096.1#Psma4#26441# proteasome subunit alpha type-4 [Mus musculus]
160333553	NP_033102.2#Rpl12#269261# 60S ribosomal protein L12 [Mus musculus]
254553321	NP_058655.3#Rps3a1#20091# 40S ribosomal protein S3a [Mus musculus]
31982332	NP_032157.2#Glul#14645# glutamine synthetase [Mus musculus]
755503893	XP_011238429.1#Nbea#26422# PREDICTED: neurobeachin isoform X6 [Mus muscul
568941435	XP_006505979.1#Usp5#22225# PREDICTED: ubiquitin carboxyl-terminal hydrolase
7242197	NP_035315.1#Psmb1#19170# proteasome subunit beta type-1 precursor [Mus mus
76881807	NP_033270.3#Serpina1b#20701# alpha-1-antitrypsin 1-2 precursor [Mus musculus]
8567410	NP_038709.1#Syn2#20965# synapsin-2 isoform IIb [Mus musculus]
31543349	NP_032766.2#Nsf#18195# vesicle-fusing ATPase [Mus musculus]
569005698	XP_006531767.1#Sptbn2#20743# PREDICTED: spectrin beta chain, non-erythrocytic
22094075	NP_031477.1#Slc25a5#11740# ADP/ATP translocase 2 [Mus musculus]
13385054	NP_079873.1#Ndufb3#66495# NADH dehydrogenase [ubiquinone] 1 beta subcomp
166851828	NP_079589.2#Atp5d#66043# ATP synthase subunit delta, mitochondrial precursor
6671539	NP_031464.1#Aldoa#11674# fructose-bisphosphate aldolase A isoform 2 [Mus mus
568958751	XP_006510025.1#Hyou1#12282# PREDICTED: hypoxia up-regulated protein 1 isofor
569009085	XP_006541454.1#Sept6#56526# PREDICTED: septin-6 isoform X5 [Mus musculus]
158303337	NP_001103445.1#Synpo#104027# synaptopodin isoform B [Mus musculus]
225543319	NP_033529.3#Vcp#269523# transitional endoplasmic reticulum ATPase [Mus muscu
568935665	XP_006535018.1#Cops4#26891# PREDICTED: COP9 signalosome complex subunit 4
34610235	NP_918943.1#Rtn4#68585# reticulon-4 isoform A [Mus musculus]
31981830	NP_034075.2#Cox7a2#12866# cytochrome c oxidase subunit 7A2, mitochondrial pr
568952442	XP_006536292.1#Rplp2#67186# PREDICTED: 60S acidic ribosomal protein P2 isofor
6754240	NP_034601.1#Hpca#15444# neuron-specific calcium-binding protein hippocalcin [M
112807195	NP_034072.2#Cox5b#12859# cytochrome c oxidase subunit 5B, mitochondrial [Mus
568917807	XP_006499960.1#Stk39#53416# PREDICTED: STE20/SPS1-related proline-alanine-ric
227499234	NP_001153096.1#Rnpep#215615# aminopeptidase B isoform 2 [Mus musculus]
568983700	XP_006517472.1#Tppp#72948# PREDICTED: tubulin polymerization-promoting prot
568910653	XP_006496809.1#Tnr#21960# PREDICTED: tenascin-R isoform X3 [Mus musculus]
39652626	NP_035924.2#Bpnt1#23827# 3'(2'),5'-bisphosphate nucleotidase 1 [Mus musculus]
576067874	NP_001276603.1#Fbxo41#330369# F-box only protein 41 [Mus musculus]
568952682	XP_006508569.1#Nap1l4#17955# PREDICTED: nucleosome assembly protein 1-like
9790219	NP_062745.1#Dstn#56431# destrin [Mus musculus]
13195624	NP_077159.1#Ndufa10#67273# NADH dehydrogenase [ubiquinone] 1 alpha subcon
6753618	NP_034157.1#Ddt#13202# D-dopachrome decarboxylase [Mus musculus]
172072657	NP_064382.3#Alg2#56737# alpha-1,3/1,6-mannosyltransferase ALG2 [Mus muscul
27754144	NP_079592.2#Ndufb5#66046# NADH dehydrogenase [ubiquinone] 1 beta subcomp
568955190	XP_006509606.1#Ncan#13004# PREDICTED: neurocan core protein isoform X1 [Mu
269784615	NP_001161421.1#Atp2b4#381290# plasma membrane calcium-transporting ATPas

6677801	NP_033118.1#Rps17#20068# 40S ribosomal protein S17 [Mus musculus]
6753508	NP_034076.1#Cplx2#12890# complexin-2 [Mus musculus]
83649713	NP_001032843.1#Baiap2#108100# brain-specific angiogenesis inhibitor 1-associate
21313679	NP_082138.1#Atp5h#71679# ATP synthase subunit d, mitochondrial [Mus musculus
46559412	NP_848142.1#Pacsin1#23969# protein kinase C and casein kinase substrate in neur
568907654	XP_006496541.1#Tuba4a#22145# PREDICTED: tubulin alpha-4A chain isoform X1 [M
568960933	XP_006510964.1#Snap91#20616# PREDICTED: clathrin coat assembly protein AP18
6754012	NP_034438.1#Gnao1#14681# guanine nucleotide-binding protein G(o) subunit alph
85861218	NP_001028472.2#Gmps#229363# GMP synthase [glutamine-hydrolyzing] [Mus mus
160707901	NP_038708.3#Syn1#20964# synapsin-1 isoform a [Mus musculus]
568972574	XP_006532728.1#Eftud2#20624# PREDICTED: 116 kDa U5 small nuclear ribonucleo
755519087	XP_006539595.2#Epn1#13854# PREDICTED: epsin-1 isoform X2 [Mus musculus]
54291704	NP_694769.2#Pacs1#107975# phosphofurin acidic cluster sorting protein 1 [Mus m
40254160	NP_083795.2#Rap2a#76108# ras-related protein Rap-2a precursor [Mus musculus]
568984258	XP_006517611.1#Map1b#17755# PREDICTED: microtubule-associated protein 1B is
357394970	NP_001239453.1#Picalm#233489# phosphatidylinositol-binding clathrin assembly p
568965313	XP_006512755.1#D10Bwg1379e#215821# PREDICTED: brefeldin A-inhibited guanin
817379474	NP_001295379.1#Aldh2#11669# aldehyde dehydrogenase, mitochondrial isoform 2
19526814	NP_598427.1#Ndufv1#17995# NADH dehydrogenase [ubiquinone] flavoprotein 1, r
755510673	XP_006503649.2#Pclo#26875# PREDICTED: protein piccolo isoform X4 [Mus muscul
9910452	NP_064304.1#Lamtor3#56692# ragulator complex protein LAMTOR3 [Mus musculu
19705578	NP_031535.2#Atp6v1b2#11966# V-type proton ATPase subunit B, brain isoform [M
755495853	XP_006497018.2#Sdhc#66052# PREDICTED: succinate dehydrogenase cytochrome l
209862992	NP_001129558.1#Dpysl3#22240# dihydropyrimidinase-related protein 3 isoform 1
568982871	XP_006517079.1#Sncb#104069# PREDICTED: beta-synuclein isoform X1 [Mus musc
7710012	NP_057878.1#Crym#12971# ketimine reductase mu-crystallin [Mus musculus]
21312012	NP_080979.1#Ndufa8#68375# NADH dehydrogenase [ubiquinone] 1 alpha subcom
568988256	XP_006519357.1#Rabggta#56187# PREDICTED: geranylgeranyl transferase type-2 su
31543976	NP_061359.2#Ywhag#22628# 14-3-3 protein gamma [Mus musculus]
12963633	NP_075801.1#Ndufa13#67184# NADH dehydrogenase [ubiquinone] 1 alpha subcon
13386100	NP_080890.1#Ndufa5#68202# NADH dehydrogenase [ubiquinone] 1 alpha subcom
568950486	XP_006507823.1#Fus#233908# PREDICTED: RNA-binding protein FUS isoform X2 [M
18158420	NP_076302.1#Camk2d#108058# calcium/calmodulin-dependent protein kinase typ
162417957	NP_001104784.1#Ngef#53972# ephexin-1 isoform 1 [Mus musculus]
13384778	NP_079672.1#Pgls#66171# 6-phosphogluconolactonase isoform 1 [Mus musculus]
116014342	NP_001070652.1#Bsg#12215# basigin isoform 2 precursor [Mus musculus]
6755965	NP_035825.1#Vdac2#22334# voltage-dependent anion-selective channel protein 2
6677813	NP_033124.1#Rps8#20116# 40S ribosomal protein S8 [Mus musculus]
10946854	NP_067438.1#Prps1#19139# ribose-phosphate pyrophosphokinase 1 [Mus musculu
359279908	NP_001240669.1#Tpm3#59069# tropomyosin alpha-3 chain isoform Tpm3.2cy [Mu
6753960	NP_034396.1#Gda#14544# guanine deaminase [Mus musculus]
229608940	NP_001153489.1#Gnb1#14688# guanine nucleotide-binding protein G(I)/G(S)/G(T)
226693349	NP_031621.3#Camk2b#12323# calcium/calmodulin-dependent protein kinase type
568915917	XP_006499045.1#Sirpa#19261# PREDICTED: tyrosine-protein phosphatase non-rece
33186863	NP_058018.2#Rpl13#270106# 60S ribosomal protein L13 [Mus musculus]
254675178	NP_032949.3#Prkar1b#19085# cAMP-dependent protein kinase type I-beta regulat

568929824	XP_006503492.1#Sgip1#73094# PREDICTED: SH3-containing GRB2-like protein 3-int
38371755	NP_081269.1#Ddah1#69219# N(G),N(G)-dimethylarginine dimethylaminohydrolase
13195674	NP_077249.1#Rab6a#19346# ras-related protein Rab-6A isoform 2 [Mus musculus]
157277969	NP_444493.1#Hnrnpa3#229279# heterogeneous nuclear ribonucleoprotein A3 isof
568905198	XP_006495497.1#Atp6v1h#108664# PREDICTED: V-type proton ATPase subunit H is
568955318	XP_006509666.1#Rab3a#19339# PREDICTED: ras-related protein Rab-3A isoform X1
256000796	NP_001157728.1#Tpm1#22003# tropomyosin alpha-1 chain isoform Tpm1.12br [M
31981086	NP_080270.2#Efhd2#27984# EF-hand domain-containing protein D2 [Mus musculu
755509256	XP_006538588.2#Eno1#13806# PREDICTED: alpha-enolase isoform X1 [Mus muscul
6679501	NP_032973.1#Psmc1#19179# 26S protease regulatory subunit 4 [Mus musculus]
568977589	XP_006515077.1#Prkar2b#19088# PREDICTED: cAMP-dependent protein kinase typ
568951392	XP_006508263.1#Ndufab1#70316# PREDICTED: acyl carrier protein, mitochondrial i
51092272	NP_808574.2#Opcml#330908# opioid-binding protein/cell adhesion molecule prec
568982992	XP_006517138.1#Hapln1#12950# PREDICTED: hyaluronan and proteoglycan link pr
261862282	NP_035531.3#Slc2a3#20527# solute carrier family 2, facilitated glucose transporter
166235165	NP_033331.2#Syp#20977# synaptophysin [Mus musculus]
254587958	NP_079793.2#Rtca#66368# RNA 3'-terminal phosphate cyclase [Mus musculus]
227430399	NP_001153084.1#Lrrc57#66606# leucine-rich repeat-containing protein 57 isoform
13385558	NP_080337.1#Ndufb8#67264# NADH dehydrogenase [ubiquinone] 1 beta subcomp
6678551	NP_033523.1#Vamp2#22318# vesicle-associated membrane protein 2 [Mus muscul
6754920	NP_035084.1#Ncam2#17968# neural cell adhesion molecule 2 isoform b precursor
33859690	NP_080728.1#Coq9#67914# ubiquinone biosynthesis protein COQ9, mitochondrial
568948233	XP_006541123.1#Sv2b#64176# PREDICTED: synaptic vesicle glycoprotein 2B isofor
161016826	NP_031900.3#Reep5#13476# receptor expression-enhancing protein 5 [Mus musc
568945112	XP_006539726.1#Ppp5c#19060# PREDICTED: serine/threonine-protein phosphatas
83715998	NP_031533.2#Atp5k#11958# ATP synthase subunit e, mitochondrial [Mus musculus
568995358	XP_006522204.1#Usp7#252870# PREDICTED: ubiquitin carboxyl-terminal hydrolase
568917774	XP_006499945.1#Nckap1#50884# PREDICTED: nck-associated protein 1 isoform X2
238231384	NP_032972.3#Psmb6#19175# proteasome subunit beta type-6 precursor [Mus mus
568986489	XP_006518504.1#Adk#11534# PREDICTED: adenosine kinase isoform X2 [Mus musc
329664963	NP_001192314.1#Actr3#74117# actin-related protein 3 [Mus musculus]
312596932	NP_001186115.1#Slc44a2#68682# choline transporter-like protein 2 isoform 1 [Mu
6679911	NP_032097.1#Gabrb3#14402# gamma-aminobutyric acid receptor subunit beta-3 i
568983719	XP_006517481.1#Cltb#74325# PREDICTED: clathrin light chain B isoform X1 [Mus m
755502503	XP_011246447.1#Tpd52#21985# PREDICTED: tumor protein D52 isoform X8 [Mus n
755533985	XP_011241621.1#Ap3d1#11776# PREDICTED: AP-3 complex subunit delta-1 isofor
19705424	NP_033465.1#Psmd3#22123# 26S proteasome non-ATPase regulatory subunit 3 [M
569000227	XP_006524307.1#Syngap1#240057# PREDICTED: ras/Rap GTPase-activating protein
9506983	NP_062284.1#Ppp2ca#19052# serine/threonine-protein phosphatase 2A catalytic s
568970106	XP_006514692.1#Lgalsl#216551# PREDICTED: galectin-related protein isoform X2 [
569003077	XP_006525616.1#Cdh2#12558# PREDICTED: cadherin-2 isoform X1 [Mus musculus]
6755911	NP_035790.1#Txn1#22166# thioredoxin [Mus musculus]
112181182	NP_031773.2#Cox5a#12858# cytochrome c oxidase subunit 5A, mitochondrial prec
6678047	NP_033247.1#Snca#20617# alpha-synuclein [Mus musculus]
6681079	NP_031824.1#Ctsb#13030# cathepsin B preproprotein [Mus musculus]
568967188	XP_006513531.1#Mcu#215999# PREDICTED: calcium uniporter protein, mitochond

188219614	NP_035800.2#Uchl1#22223# ubiquitin carboxyl-terminal hydrolase isozyme L1 [Mu
6680836	NP_031617.1#Calr#12317# calreticulin precursor [Mus musculus]
7305443	NP_038749.1#Rpl7a#27176# 60S ribosomal protein L7a [Mus musculus]
6755080	NP_035232.1#Prkcg#18752# protein kinase C gamma type isoform 1 [Mus musculu
568978770	XP_006515515.1#Cfl2#12632# PREDICTED: cofilin-2 isoform X1 [Mus musculus]
568968337	XP_006514080.1#Atp2b1#67972# PREDICTED: plasma membrane calcium-transpor
84794552	NP_061346.2#Pebp1#23980# phosphatidylethanolamine-binding protein 1 [Mus m
13937391	NP_034442.1#Gnb2#14693# guanine nucleotide-binding protein G(I)/G(S)/G(T) sub
52317148	NP_067467.2#Ap3b2#11775# AP-3 complex subunit beta-2 [Mus musculus]
225543378	NP_001139449.1#Cadm2#239857# cell adhesion molecule 2 isoform 2 precursor [M
160707958	NP_001104264.1#Anxa7#11750# annexin A7 [Mus musculus]
167716839	YP_001686700.1#COX1#5912286# cytochrome c oxidase subunit I [Mus musculus n
568984656	XP_006517804.1#Rab3c#67295# PREDICTED: ras-related protein Rab-3C isoform X2
31560653	NP_032852.2#Pfkl#18641# ATP-dependent 6-phosphofructokinase, liver type [Mus
506326231	NP_001265185.1#Prnp#19122# major prion protein precursor [Mus musculus]
58219050	NP_081226.1#Ociad2#433904# OCIA domain-containing protein 2 [Mus musculus]
38372905	NP_075615.2#Rab8a#17274# ras-related protein Rab-8A [Mus musculus]
568946346	XP_006540322.1#Gsk3a#606496# PREDICTED: glycogen synthase kinase-3 alpha isc
13385090	NP_079904.1#Cox6b1#110323# cytochrome c oxidase subunit 6B1 [Mus musculus]
226437665	NP_081520.1#Ndufa11#69875# NADH dehydrogenase [ubiquinone] 1 alpha subcon
20330802	NP_598738.1#Trf#22041# serotransferrin precursor [Mus musculus]
123	K2C1_HUMAN
568900009	XP_006543175.1#Gm10257#544973# PREDICTED: histone H3.3-like [Mus musculus
568921663	XP_006500999.1#Bcan#12032# PREDICTED: brevican core protein isoform X1 [Mus
568949306	XP_006507253.1#Me3#109264# PREDICTED: NADP-dependent malic enzyme, mito
157951643	NP_150371.4#Actn2#11472# alpha-actinin-2 [Mus musculus]
185	PPIA_HUMAN
62000629	NP_084137.1#Cnrip1#380686# CB1 cannabinoid receptor-interacting protein 1 [Mu
755569271	XP_011249834.1#Mat2a#232087# PREDICTED: S-adenosylmethionine synthase isof
755491057	XP_011236578.1#LOC102642577#102642577# PREDICTED: serine protease inhibitc
6679227	NP_032817.1#Pcp4#18546# Purkinje cell protein 4 [Mus musculus]
108	K1C10_HUMAN
163310765	NP_033784.2#Alb#11657# serum albumin precursor [Mus musculus]
17647499	NP_058652.1#Hbb-b2#15130# hemoglobin subunit beta-2 [Mus musculus]
19526794	NP_598416.1#Cd81#12520# CD81 antigen [Mus musculus]
498752597	NP_001265090.1#Hbb-b1#15129# hemoglobin subunit beta-1 [Mus musculus]

Wt stressed / Wt	Knockout stressed/ WT stressed	Knockout/ WT	WT stressed / Knockout
0.832351416	0.841737799	0.841177243	1.036643488
0.641175258	1.35784576	0.878402481	0.752217282
0.596942124	1.437883703	0.896850436	0.669233299
0.809632281	1.020564304	0.949361898	0.851796346
0.748652224	1.300937547	0.983128969	0.76995862
1.222629928	0.879247229	0.994840418	1.227989255
0.758766045	1.058381557	1.002672718	0.756832154
0.911834905	1.273047523	1.007208075	0.905547377
1.065727509	0.930846114	1.009543793	0.987082649
0.877444557	0.932417352	1.011106249	0.881563073
0.80324609	1.239664446	1.014472254	0.801014101
1.118025988	0.851254457	1.017427443	1.118246492
0.885934625	0.911500456	1.019596128	0.923670749
1.017353551	0.86240955	1.021169439	0.995943027
1.036210437	1.087818168	1.02374166	1.014730033
1.17885611	0.810723441	1.024082352	1.150466643
0.945952568	0.937514831	1.031652637	0.900481933
1.030991871	1.269506939	1.034916648	0.963017158
1.064443636	0.892968802	1.035430556	0.941834957
0.99616121	0.93929546	1.038379321	0.962701074
1.144801623	0.981631165	1.040533119	1.099965773
1.159900259	0.871648185	1.041477656	1.113403122
0.879668841	0.879135507	1.044872245	0.805871535
1.09545974	0.933576705	1.046369651	1.047235955
1.192846572	0.89802617	1.048620043	1.138328889
1.096315649	0.782078411	1.049038582	1.021795363
0.953003472	0.941141946	1.050212467	0.908911732
1.058446457	0.919444374	1.05387495	1.045345887
1.095723748	0.965058826	1.055054992	0.984989213
1.150372943	0.925412889	1.05607078	1.020312845
1.167504612	0.854804908	1.056331086	1.104985526
1.132952446	0.932768738	1.060813467	1.065092069
1.074402781	0.831878754	1.064601797	1.031219243
1.034632798	0.978568232	1.066334243	0.969516452
1.270929024	0.786028999	1.066867664	1.148022015
1.125069539	0.922063125	1.067574904	1.022870614
1.014845469	0.912995706	1.068495207	0.945881218
0.817745327	1.054790371	1.069635339	0.786802233
1.004518728	1.049418167	1.072640043	1.002831058
0.913300953	0.930668979	1.07457454	0.85116821
1.015946445	0.983922476	1.075154149	0.921045003
1.087605633	0.857315026	1.075664972	1.011094166
1.185724535	0.917070595	1.075729692	1.064422066
1.133439515	0.945918962	1.076012817	1.122370596
1.094158329	1.035235078	1.077099671	1.015742689

1.132993999	0.872772883	1.077229815	1.052069952
2.099009713	0.805961643	1.077599048	1.875151589
0.819734475	0.938453944	1.077832731	0.632421021
0.874579306	0.91448944	1.078278599	0.772971084
0.917956158	0.773768434	1.078301361	0.85856696
0.999200379	1.140413483	1.078380435	0.913352395
0.991212358	0.902568591	1.07846886	0.946007447
1.067224522	0.878169037	1.081814788	1.027112901
1.217966631	1.00729811	1.085072833	1.122490596
1.00200628	0.841810684	1.088490918	0.974804634
0.987596327	0.911864138	1.088653485	0.804354778
1.039830618	0.891129539	1.089404018	0.946860954
1.150353558	0.853622217	1.089874676	1.113593487
0.994397223	0.899203669	1.090386793	0.914924455
1.038210232	0.834638279	1.090561607	0.967411995
1.279368432	0.921980492	1.091725966	1.101810989
0.840106909	1.394752757	1.091821357	0.784619248
1.182830758	0.814471528	1.092080248	1.033510239
1.066890621	0.951273503	1.093472165	0.979787047
1.114390369	0.955798802	1.09477597	1.022802623
1.209571001	0.834989507	1.094827336	1.106067726
1.124348963	0.991634966	1.094893029	1.026903025
1.054939205	0.912253165	1.09500657	0.951708867
1.114368464	0.950647405	1.095420786	1.038723869
1.134216005	0.908697528	1.095626996	1.023511914
1.035702173	0.976317102	1.096062705	0.93459354
1.199374325	0.98809693	1.096384355	1.066691564
1.05084795	0.991633203	1.096433127	0.93270023
0.978433352	0.99548483	1.096492193	0.911286543
1.014503596	0.876670469	1.096685113	0.909477695
1.016473454	1.011338463	1.096851593	1.004996694
1.235517726	0.858865529	1.096877068	1.084775194
1.009069316	1.035197015	1.097390041	0.921163175
1.005705779	0.856149057	1.097569692	0.882421582
1.777219023	0.929951155	1.098063535	1.531157358
1.048798414	0.950980243	1.098544327	0.995156515
1.003147966	0.939707056	1.099439813	0.958720287
1.126092806	0.904992491	1.09957361	1.010973207
1.138525205	0.965662382	1.099711452	1.035277564
0.753353853	0.867899328	1.099868127	0.684298599
1.094200956	0.910648633	1.099889155	0.972765825
1.000390279	1.094137168	1.100660023	0.891358443
1.144183676	0.8821717	1.101471013	1.05740503
1.161367036	0.944744002	1.103895869	1.040057891
1.13148361	0.832574669	1.103948928	0.973729695
1.24201976	0.950090652	1.104152017	1.077046319

1.100568868	0.873921077	1.104688053	1.00158125
1.061952406	0.939195715	1.104868194	0.911207539
1.137948856	0.970001968	1.105086537	1.00664658
0.823796246	0.9161287	1.105252844	0.777888357
1.118478379	1.002687878	1.105333797	1.024762848
1.149359115	0.909318102	1.106887013	1.014465891
0.979992033	1.021698756	1.107863566	0.884212653
1.072776557	0.901873856	1.108821792	0.966955268
1.330568368	0.979368579	1.10895706	1.278123582
0.917182996	1.011333813	1.110218226	0.826122653
1.431722629	0.790393883	1.110527222	1.289147594
1.039997191	0.790089829	1.110986566	0.907308839
0.983696567	1.000343408	1.111015488	0.858590098
1.137606278	0.875268929	1.111430441	1.021227722
1.029204465	1.006697746	1.111483522	0.925500447
0.931766087	0.908779459	1.11169732	0.845973821
1.10905622	0.820232243	1.111858518	0.99833854
0.854740739	0.847305007	1.112283932	0.788545677
1.012636928	0.85176799	1.112565672	0.951502653
1.103874709	0.922438564	1.112984048	0.959400251
1.02940402	0.949492309	1.113009322	0.913233642
0.94522716	0.900864736	1.113326215	0.849309189
1.162909771	0.940685828	1.113369565	1.062417159
0.960187336	0.822949079	1.113659706	0.868681702
0.938476168	0.967285763	1.114299273	0.842292332
1.165708011	0.958564262	1.114358578	1.046531918
1.00181648	0.892805876	1.114751188	0.958269169
1.045642643	0.967235403	1.115256437	0.900161986
0.950982335	0.914756332	1.115298766	0.84599107
1.144580835	1.00165211	1.11731106	0.930405636
1.197174781	1.069605507	1.117532312	1.040121267
1.034341793	0.949419022	1.118000503	0.923413801
0.863150088	0.888811546	1.118020918	0.764883342
1.175520995	0.906170447	1.118210608	1.052148573
1.096998181	0.895598636	1.119788823	0.98023836
0.724273476	1.216102238	1.119818249	0.673702308
1.070872966	0.886616368	1.119886257	0.955366016
0.859443183	1.105647955	1.119949624	0.799215498
0.942763235	0.880332796	1.12009995	0.756239095
1.041351456	0.912931431	1.120173884	0.911808779
1.122616338	0.847021743	1.120215482	1.002584033
1.026266416	0.916334565	1.120658246	0.933269804
0.937219784	1.040133537	1.120836145	0.831226943
1.124512159	0.925421991	1.121074763	1.038646661
1.110335991	0.920457399	1.121093519	0.957843679
1.054948916	0.878565576	1.121463275	0.899093136

1.029927003	0.893446581	1.121622027	0.879589102
1.068454681	0.907678782	1.121931717	0.971149085
1.131912959	0.901234377	1.122495094	1.04943102
1.107809964	0.873948412	1.122854499	0.998159971
1.253268304	0.936706173	1.122993336	1.12640648
1.133248657	0.844790277	1.123233443	1.009331928
1.261424103	0.986410023	1.123533261	1.098299046
1.045843438	0.847996645	1.123643205	0.902428826
1.095673535	0.895747497	1.123916203	0.942259483
1.002460859	0.836743918	1.124452328	0.937545722
1.104213866	0.914207029	1.124585283	0.969490903
1.092897916	0.925381012	1.124597077	0.923573923
1.157069617	0.972298817	1.124679004	0.90421315
1.071486918	0.942949816	1.125078243	0.952042908
1.251089489	0.866069034	1.125349278	1.137915853
1.228351625	0.89106716	1.125371759	1.116365926
1.222188011	0.951972237	1.125765726	1.090816489
1.120984544	0.829989432	1.126419653	0.937605063
1.113309999	0.91688562	1.126559126	0.988053904
1.074546434	0.921864474	1.126811467	0.907997023
0.984391404	0.801877618	1.126855126	0.89003754
1.242170685	0.934985705	1.126956242	1.087711185
1.217149625	0.872213987	1.127711928	1.071079454
1.034205294	0.893941902	1.12771649	0.918230569
1.250834555	0.876883834	1.128180105	1.107825703
1.025405216	0.892040421	1.128363463	0.936269055
0.957952564	0.927557447	1.128457185	0.871672112
0.975733142	0.852407107	1.128546882	0.864719185
1.103258688	0.890855724	1.128776965	1.001711338
0.856220268	0.937271399	1.129430273	0.757776528
1.116037283	0.913805119	1.129449834	0.9974624
1.095191778	0.884141358	1.129631219	0.973285099
1.026856372	0.895561846	1.129964126	0.921819739
1.04590107	0.880247921	1.13025868	0.932325808
0.97965811	0.890201848	1.130271445	0.909500003
1.022295927	0.912090312	1.130296494	0.903569069
0.93885286	0.917405907	1.130630605	0.852304604
0.941986964	0.9329879	1.13094912	0.845380359
1.114250851	0.874283869	1.13223578	0.982440255
1.305669907	0.890011886	1.132276652	1.179656103
1.12479652	0.914766258	1.133115737	0.956356131
0.739812987	1.022217507	1.133182952	0.674190742
1.024475577	0.928505254	1.133946922	0.903583788
1.086816744	1.072778541	1.134051171	0.997043264
1.1538853	0.912609417	1.134121724	1.015639475
1.18028953	0.892102157	1.134287693	1.047810477

1.007348589	0.937417484	1.134397879	0.872731358
1.014245159	0.910858106	1.13459199	0.895826974
1.068886538	0.829747103	1.134643158	0.886363984
0.978347379	1.097966327	1.134928333	0.853927897
1.115887553	0.90150127	1.135185173	0.992381686
1.067500751	0.902634485	1.135268143	0.890692051
2.094183569	1.002011703	1.135638341	1.842566594
0.933851208	0.890095012	1.136032187	0.833110417
1.070833185	0.853746323	1.136231368	0.978327248
1.078510923	0.893996085	1.136298771	0.948989766
0.93590712	0.907610441	1.136322296	0.84475713
1.185858904	0.835681472	1.136384242	1.072624589
1.049941256	0.926529869	1.137122271	0.932300424
1.084508791	0.899023717	1.137283784	0.916879064
1.143954858	0.893282285	1.137381031	1.010991738
1.17165696	0.934233367	1.137516994	1.062939581
0.992207	0.935284575	1.137664534	0.968756549
1.010439602	0.911970464	1.137665161	0.949304592
1.050814493	0.865728452	1.138545021	0.901768701
0.859888348	0.841952714	1.139184989	0.765580245
1.297781828	0.846300508	1.139553257	1.09769233
0.89795565	0.877819034	1.139722808	0.764231646
1.227748995	0.918108531	1.139870722	1.000249871
1.592935405	0.835913615	1.139963157	1.297778948
1.017904197	0.932601187	1.140077316	0.912351315
0.929966988	1.035701072	1.140388977	0.814288714
1.351314371	0.793928752	1.141660614	1.156728948
1.13617624	1.061947872	1.141845402	0.932759629
1.077330761	0.93901496	1.141879374	0.943834593
1.019492399	0.822230532	1.142088927	0.863127988
1.035779522	0.884233442	1.14217646	0.875411368
1.061933937	0.948137796	1.14275343	0.932516814
1.069327078	0.887241936	1.142784798	0.871259662
0.907437585	0.960476153	1.142852462	0.793906484
1.13279208	0.837585597	1.142941648	0.992545452
1.096141148	0.952704054	1.142961645	0.904512704
1.034486608	0.915728831	1.143066422	0.905366818
1.154097022	0.879536492	1.143217659	1.044386922
1.009779837	0.767034157	1.143790519	0.888739225
1.113765632	1.090761864	1.14396305	0.973505311
0.812190348	0.862679318	1.1445879	0.693785135
1.041360789	0.915554858	1.144826756	0.90821379
1.095756964	0.924290679	1.14483814	0.912152166
1.131766576	0.885251475	1.144841651	0.973346568
1.254088224	0.931468099	1.144849647	1.077154631
1.190318248	0.857489845	1.145112034	1.041312126

1.051552968	0.941624846	1.145148656	0.879850857
1.011346234	0.821107238	1.14531755	0.937142062
0.882423632	1.113097157	1.145454456	0.761102485
0.535289233	0.858992979	1.145790417	0.474518515
1.036770773	0.893603844	1.145988279	0.905472623
0.988046645	0.921739384	1.14601204	0.895495393
1.170377498	0.968802982	1.146691064	1.046784355
1.014370515	0.897681185	1.146731018	0.835809697
1.040414974	0.949528602	1.148065347	0.920725095
0.872094273	0.660794886	1.148265017	0.752178046
1.017693057	0.907437222	1.148362573	0.875590557
1.019860369	0.908774426	1.148403328	0.903900639
1.128540988	0.969061863	1.14843436	0.972800364
1.060393325	0.87239059	1.148486385	0.961610169
1.124161125	0.935392013	1.148530672	0.979877309
1.155080489	0.909553984	1.148617571	0.996482759
1.176256217	0.899424519	1.148823434	1.023075583
0.766342853	1.148527959	1.148953449	0.647702848
1.050011009	1.079494376	1.149424273	0.913469547
1.028470266	0.857749529	1.149586562	0.894718565
1.059029161	0.836288935	1.15028465	0.987394903
1.174557669	0.893145889	1.150541183	1.020116146
1.136991513	0.907924272	1.150560269	0.961379734
1.067992414	0.865755754	1.150687488	0.898083695
1.195812289	0.959176509	1.150791505	1.068728273
1.139330754	1.002542178	1.150937843	0.98978692
1.184250455	0.925101435	1.150978692	1.055940765
1.146261637	0.940308496	1.150994654	1.007851909
0.939685252	0.893026661	1.151281286	0.821301742
0.87897235	0.890161426	1.151370869	0.796396984
1.043673755	0.938789184	1.151494725	0.897273828
1.140468838	0.909948762	1.152097212	0.988039516
1.105391728	0.880757386	1.152190164	1.019381158
1.06577862	1.104709046	1.152208362	0.902021847
1.113347844	0.977378387	1.152522646	0.968845088
0.821738256	0.926481561	1.153091116	0.735756421
1.159123307	0.899562055	1.153279712	0.992315766
1.269036452	0.967677327	1.153285746	1.078942196
1.164969613	0.849568086	1.153463569	1.036710186
0.957084158	0.873702277	1.153609661	0.804912266
0.987560585	0.912380651	1.153989978	0.857157179
1.051386962	0.928998947	1.154225459	0.931144997
1.124674709	0.852687857	1.154394125	0.935050396
1.105860921	0.770668889	1.154996083	1.021485017
0.999279973	0.885654245	1.155352535	0.843246471
1.177030605	0.935107639	1.15540326	0.95707397

0.68159126	0.964366195	1.155588115	0.575833332
1.04482934	0.843990853	1.156012249	0.93307693
0.952423013	0.965471046	1.156190858	0.800432953
1.146019277	0.981292757	1.156309618	0.992476709
1.03133723	0.873857364	1.156485902	0.877958257
1.073508794	0.843781678	1.156489561	0.993425291
1.175454113	0.866281538	1.156800002	1.015822839
1.099378835	0.867788544	1.156951183	0.947371385
1.165534029	0.936287106	1.157150189	1.005443221
1.146265613	0.853426187	1.157305106	0.947286836
1.120347447	0.861839004	1.157530095	0.972673648
0.862830897	0.979615317	1.157665307	0.794747994
1.068982262	1.012122005	1.157711924	0.91650801
1.167793516	0.94396991	1.157984788	0.975663536
1.148112137	0.88740883	1.158605467	1.015426065
1.271730805	0.861002784	1.158865157	1.090633461
1.094161473	0.829219546	1.159065222	0.962165504
0.996708737	0.763616405	1.159067976	0.875092997
0.919244543	1.121354877	1.159288076	0.777184009
0.865005736	1.002680629	1.159319854	0.746074063
1.107154761	0.917845324	1.159362806	0.941457978
1.00562543	0.893149755	1.160027826	0.890476067
1.149008017	0.864547063	1.160873096	1.007465602
0.999757107	0.944478421	1.160973125	0.888026784
0.988752211	0.85044497	1.161575158	0.881391341
1.1760541	0.915723503	1.161868011	1.053527989
1.010176324	1.02873019	1.162293142	0.882779592
1.32463465	0.77856358	1.162300874	1.108784389
1.077507206	0.931286469	1.162355139	0.892069263
1.065909434	0.862917926	1.162731118	0.871805164
1.127614855	0.841010592	1.16273569	1.007744711
1.22487187	0.936463096	1.162762159	1.043910438
1.047624002	0.916477851	1.163131984	0.841954357
1.006089412	0.868029243	1.163754855	0.976345481
1.151888467	0.923583183	1.163825157	0.95739462
1.156185986	0.757119667	1.163838048	1.070052116
1.107869757	0.868993054	1.164019086	0.975917126
0.789523258	0.862180242	1.164074552	0.719169965
1.207610625	0.929848661	1.164103641	1.037326859
1.094797516	0.933441234	1.16456029	0.932274174
0.959103929	1.032986684	1.16466278	0.84239045
0.999362962	0.866403029	1.16479957	0.849060274
1.141102168	0.944499453	1.165119211	1.002330821
0.974100099	0.932533296	1.165546163	0.765353081
1.080401619	0.855717538	1.165560213	0.964649191
1.117218323	0.971697705	1.165600992	0.937940577

1.018851409	0.992580159	1.16561951	0.90859685
1.05312249	0.899727067	1.165951396	0.936359374
0.817080422	1.110258999	1.166036353	0.702717391
1.068641545	0.877780408	1.166132717	0.889640715
1.260242066	0.756625975	1.166563099	1.062921499
1.078552949	0.816447756	1.166893835	0.88843849
1.172938741	0.903397943	1.166908997	1.006245693
1.149347357	0.89910222	1.167008874	0.984865996
1.116692029	0.79724568	1.167237927	0.934121376
1.137014784	0.860913147	1.167237949	0.939276759
1.079929941	0.934014762	1.167526278	0.9631888
1.161037982	0.874470269	1.167686908	0.940113631
1.026340202	0.883608184	1.168115553	0.85859575
1.07265429	0.894504756	1.168180608	0.915516965
1.186565677	0.907377437	1.168260842	1.044864798
1.056665986	0.858952145	1.168573863	0.890592396
1.370952118	0.96395552	1.168617578	1.162552955
1.038026337	0.843301282	1.168620663	0.882311653
1.165094897	0.889905518	1.168675089	1.059329894
1.093520775	0.946510625	1.169058577	0.885956938
1.059173428	0.919240945	1.169135487	0.918421211
1.264590072	0.892594175	1.169189689	1.073617086
0.870045819	1.052243022	1.169399942	0.758845069
1.121431768	0.87113617	1.16941048	0.951563372
1.040414064	0.943078343	1.169516841	0.88101283
1.071849072	0.871715965	1.169692882	0.926818395
0.953047866	1.017303122	1.170105628	0.749299764
1.229586111	0.887687215	1.170118595	1.026385161
0.998747224	0.91850834	1.170139389	0.800664284
1.181150624	0.845294891	1.170146669	1.043557323
1.087937428	0.921570942	1.170629357	0.963608946
1.220347781	0.937217583	1.170725266	0.982026804
0.955755513	0.945657209	1.170741992	0.816284123
1.06763803	0.896943507	1.17088516	0.908891645
1.091955779	0.918811333	1.170942866	0.95196481
1.137153219	0.843410505	1.17100939	0.971070995
1.060558682	0.922706395	1.171164776	0.895711433
1.090076026	0.928794184	1.171549866	0.930467689
1.308720206	0.810575578	1.171883036	1.116753746
0.991730741	0.998535517	1.171949523	0.845976159
0.963343371	0.811086243	1.172044389	0.822869764
0.977138765	0.887966469	1.172603474	0.887906853
1.114439793	0.947935888	1.172848285	0.928238414
1.075965941	0.84824122	1.172972344	0.906059099
1.069972514	0.952165068	1.173383097	0.943119787
1.131621748	0.858300907	1.173480191	0.964027479

1.008571652	0.812212047	1.173722766	0.852255622
0.996290916	0.876625359	1.174433986	0.811497068
0.993755811	0.929816724	1.174528639	0.864903674
1.016239397	0.907599538	1.174960492	0.783511061
0.871687603	1.007485078	1.17514332	0.793203776
1.239204779	0.825905348	1.175242592	1.054518965
1.175717183	0.900379883	1.175734847	1.005767784
1.058742046	0.908167093	1.175740441	0.868410114
1.026616935	0.87474928	1.175770171	0.876351268
1.158790322	0.884299566	1.175787566	0.992313366
1.000241329	0.93369062	1.175816174	0.817821505
1.199013894	0.881831228	1.175902103	1.017347988
1.077735499	0.940563266	1.175948126	0.924268667
1.136367383	0.943204223	1.17597144	0.930811727
1.071735039	0.918789125	1.175990433	0.886493818
1.260125852	0.90899182	1.176135991	1.11710101
1.056470399	0.912216417	1.176144396	0.940615825
1.141217261	0.900664264	1.176470221	0.972177795
0.99006294	0.888616943	1.176632186	0.844345277
1.048965215	0.934497535	1.177100965	0.901228204
1.269251672	0.914212843	1.177159235	1.081571809
1.148364947	0.997615271	1.177160144	0.925486921
1.242778165	0.842326413	1.177256993	0.957526618
1.070050312	0.956328419	1.177391003	0.852125433
1.168779612	0.936411135	1.177418485	1.004622057
1.045791867	0.931862152	1.177589147	0.8694718
1.20070525	0.921704478	1.177883698	1.020022316
1.159021907	0.843803368	1.177889808	0.98346746
1.113023246	0.919402461	1.177905	0.968088535
1.311037577	0.894816296	1.178289136	1.088458343
1.137420688	0.908601713	1.178452375	0.966571765
1.176396612	0.829684606	1.178570209	1.019732372
0.83141282	0.938052589	1.178620542	0.704781391
1.168467551	0.924508895	1.178630314	0.997777985
1.129063257	0.909999182	1.178847466	0.956747332
1.058824497	0.940493543	1.178923885	0.89795005
0.947681186	0.923512012	1.179205013	0.808658139
1.112411379	0.928147537	1.179384708	0.954096569
1.000919683	0.900617497	1.179489253	0.846886529
0.97239093	0.898717965	1.17958039	0.824177581
1.153679007	0.905116757	1.179816711	0.97507569
1.110445057	0.879534164	1.18003076	0.948086228
1.121067643	0.901844683	1.180067401	0.938364947
1.087953422	0.891056536	1.180104857	0.931994387
1.067826225	0.87300058	1.18013193	0.88931233
0.999806195	0.906706404	1.180144112	0.784422646

0.958207226	0.90073362	1.180152412	0.811656543
1.054750988	0.944148372	1.180197294	0.896615721
1.077585849	0.846358179	1.180294372	0.94062305
1.052567685	0.901778076	1.180451325	0.880906802
1.014692215	0.836804681	1.180725528	0.899428685
1.058184731	0.81566436	1.180991414	0.872843494
1.094417848	0.825243831	1.181039983	0.946774418
1.131941294	0.879427529	1.181070937	1.031376931
1.071992339	1.003986181	1.181286845	0.881076078
1.031564418	0.913275803	1.181288913	0.96107208
1.098090581	0.855900066	1.18133069	0.93043363
1.113272659	0.88759022	1.181402043	0.931119784
1.000768884	0.947484447	1.181524589	0.814922855
1.049478093	0.930386607	1.18159272	0.91353738
0.991141267	0.903411477	1.181657667	0.833385938
1.100775779	0.852010474	1.181956625	0.934077059
1.071513673	0.849855883	1.182121728	0.910236818
1.077318073	0.836232228	1.182213099	0.901050102
1.200000103	0.941264287	1.182380081	1.013352988
1.054267904	0.926788022	1.182423576	0.892702899
1.169612159	0.915759362	1.182459265	0.997038
1.061973288	0.797300774	1.182594096	0.909402141
1.080981443	0.933286323	1.182890663	0.897313505
0.990244825	0.947742132	1.18293948	0.811568315
1.286738412	0.947688537	1.183284643	1.080629098
0.798251762	1.202182561	1.183293328	0.709563523
1.119289077	0.923916676	1.183318907	0.965031286
1.068798955	0.957092699	1.183852729	0.946438156
1.127781408	0.931306356	1.183930695	0.978561672
1.040414171	0.876858139	1.183955058	0.834494977
0.970030031	0.925585672	1.184055295	0.830426668
1.079343626	0.934364132	1.184066187	0.910230644
1.120424973	0.959689964	1.184215333	0.946132804
1.05873915	0.907033623	1.184781518	0.914069842
1.106657399	0.952318512	1.18522599	0.912284496
1.165245656	0.866166325	1.18529169	1.011579418
0.848044012	0.887231935	1.185614205	0.717464887
1.088873961	0.916714526	1.185652646	0.920349484
1.167956077	0.9234504	1.18621333	1.007533915
1.083527079	0.968001707	1.186324126	0.914265083
1.046806703	0.905144622	1.18633051	0.912739987
1.113165699	0.878897785	1.186340784	0.93296267
1.210680833	0.872710404	1.186367214	0.994291683
1.23855189	0.882234502	1.186568496	1.060171758
1.018598045	0.91242022	1.186629337	0.858356596
1.22899973	0.862037099	1.186712125	1.073514446

0.961112418	0.90571782	1.186884217	0.805024651
1.075524567	0.94967128	1.187073689	0.904927724
0.991160841	0.815080606	1.187530653	0.834852266
1.101528103	0.894902495	1.188358063	0.914792249
1.074395624	0.993574341	1.188364785	0.859653227
1.01655455	0.93181685	1.188500463	0.864765068
1.017538875	0.940005968	1.188776089	0.874663101
1.110038939	0.888560246	1.188853931	0.933732838
1.110243656	0.906563246	1.189528137	0.923094126
1.065134203	0.93870924	1.189576043	0.892382262
1.122011513	0.883065647	1.189631545	0.961140578
0.882905288	0.94973072	1.189742129	0.734834412
1.121071961	0.834027951	1.190110145	0.938751872
1.214558301	0.944258333	1.190231034	0.98539181
1.074457205	0.885667134	1.190419459	0.894487676
1.078715581	0.868135011	1.190738474	0.889421383
1.056761436	0.796020472	1.19083615	0.797054927
1.179842948	0.91156387	1.190917077	1.007813992
1.063880369	0.845818105	1.190955276	0.839165558
1.198775063	0.913987249	1.191017073	0.973245202
1.068596154	0.887126137	1.191118587	0.96052971
1.029048574	0.880459278	1.191510493	0.862366087
1.179194617	0.917417203	1.191567854	0.993309632
1.013362832	0.881981202	1.191630356	0.850400317
1.082474237	0.935055956	1.191891122	0.92353645
1.004128423	0.836119269	1.192447214	0.801194
1.175522868	0.886549291	1.192521875	0.951781354
1.083369376	0.877301501	1.192538022	0.908544207
1.046015537	0.96385334	1.192711645	0.88160645
1.206610624	0.891695823	1.192837638	0.946481092
1.27641262	0.813207789	1.192917096	1.094161102
1.073597359	0.87712789	1.193244802	0.898331319
1.168307081	0.908985843	1.193392915	0.976196846
1.174609468	0.831437616	1.193415598	0.963154396
1.093264786	0.8589165	1.193477245	0.920951804
1.145421548	0.831332256	1.193493307	0.947754178
1.099674857	0.86395047	1.193619582	0.907453257
1.102162489	0.735192481	1.193640166	0.951491585
1.219890225	0.908833959	1.194277729	1.018720821
1.201227346	0.956011968	1.194334634	0.941873941
0.922609253	0.991675723	1.194450957	0.783947235
1.092150798	0.848567852	1.194468616	0.900480791
1.172589925	0.864603853	1.194496459	0.938369069
1.074494834	0.924163784	1.194760694	0.915031324
1.022185767	0.857614241	1.194859613	0.885431493
1.109351843	0.814666396	1.194879527	0.895481441

0.958708484	0.990041031	1.194977673	0.78161737
1.035827535	0.92573469	1.195018961	0.868710029
0.999299594	0.827485387	1.195029892	0.82594064
1.070341319	0.900325199	1.195081141	0.876048738
1.054257437	0.88582699	1.1951827	0.912703338
1.083303271	0.914530164	1.195220009	0.901257356
1.107956301	0.906976932	1.195265456	0.928973075
1.22360003	0.843716957	1.195471269	1.040287673
1.142251165	0.901937145	1.195471817	0.970733925
0.985165727	0.93183803	1.195674232	0.780581827
1.107894936	0.819864566	1.195748673	0.964535728
1.015839222	0.866681864	1.195841472	0.849465668
0.754869406	0.952558144	1.195919608	0.631789797
1.10764572	0.938543087	1.196078464	0.938536807
1.101289717	0.955417181	1.196502344	0.928383036
1.102211641	0.856455752	1.196699075	0.876763829
0.814737396	0.895833115	1.196856706	0.722682576
0.975832567	0.869061745	1.196940078	0.889336712
1.141469538	0.852339918	1.197262602	0.953397257
1.080038918	0.924012236	1.197290486	0.901863901
0.983616052	0.954271152	1.197316142	0.850596896
0.928200517	0.939699647	1.197668958	0.810466294
1.145354096	0.921260021	1.197939957	0.928412336
1.063452377	0.832571009	1.198124049	0.875657732
0.95502138	0.913330661	1.198841375	0.751070356
1.244426089	0.815368558	1.198873134	1.035357428
0.97796601	0.945570848	1.198926971	0.838257838
1.005019038	0.975751456	1.198942086	0.820641902
1.092651924	0.883005481	1.199005092	0.92002682
1.077771619	0.916687063	1.199066935	0.904506869
1.036732884	0.855980597	1.199136012	0.864317925
1.097418864	0.847501982	1.199163547	0.913907289
1.039999355	0.933230981	1.199267481	0.849651189
1.028595278	0.790457665	1.19938282	0.797796387
0.998969646	0.986227771	1.199528159	0.861578753
1.243459194	0.828768752	1.199655083	1.030200357
0.967009886	0.914214846	1.199678575	0.825731552
1.083100805	0.897789983	1.199928189	0.902414988
0.88197972	0.803846667	1.199972745	0.726922715
1.160320028	0.924741855	1.200015308	0.949533643
1.193414657	0.79605944	1.200207488	0.996767678
1.007415798	0.865462463	1.200422701	0.857668531
1.356714443	1.020320388	1.200448299	1.149759409
1.107540678	0.90024842	1.200553403	0.93944175
1.112563348	0.860335454	1.200718309	0.944606104
0.975707603	0.963214637	1.200979804	0.813136545

1.125046554	0.868852635	1.201091856	0.968986767
0.993840388	0.935186386	1.201106148	0.81136687
1.012483733	0.872563081	1.201383022	0.914129873
1.046241757	0.865512503	1.201416468	0.876203016
0.941388317	0.894485444	1.201650677	0.76328338
1.242186422	0.820580026	1.201745837	0.935646495
0.945907711	0.842155868	1.201801544	0.930643704
1.002588811	0.826333437	1.202022444	0.834841058
1.153142673	0.852849133	1.202323926	0.970035252
1.009914791	1.20703683	1.202646668	0.860006839
1.187129632	0.828128373	1.202957033	0.988933506
1.109084763	0.891440712	1.202978803	0.924703572
0.992418196	1.027835572	1.203023444	0.834206789
0.907158349	0.872990676	1.203061931	0.828923674
1.011860135	0.926054083	1.20311509	0.873130105
1.018394302	0.832085922	1.203548015	0.843393304
1.150787422	0.857408908	1.203880715	0.988705576
1.440160541	0.797648761	1.203922191	1.184342166
1.269784607	0.928103729	1.203931533	1.050382779
1.046028139	0.931198901	1.204094775	0.869458136
1.031461777	0.845931408	1.204110382	0.820534203
1.046828974	0.826859979	1.204448998	0.916203865
0.940104548	0.90377236	1.204635223	0.78148758
1.164316744	0.901141219	1.204651428	0.9660047
1.056191476	0.881211451	1.204717618	0.878326727
1.275570892	0.836580076	1.204746048	1.052581977
1.155271705	0.818660557	1.204760485	0.909959431
1.137716306	0.864329693	1.204760524	0.953867471
1.056636606	0.925959772	1.204812822	0.847417136
1.240204869	0.876323461	1.204828755	1.007804074
0.929687777	0.796163654	1.205030107	0.771750427
1.125123761	0.880991148	1.2051273	0.933733015
1.038163463	0.923180389	1.205308348	0.87063497
0.979173866	0.927710164	1.205802979	0.832441546
1.203181124	0.819876538	1.205820405	0.998132763
1.096310877	0.891287799	1.20610006	0.865709387
0.946600236	0.869396477	1.20614656	0.795628166
0.949067395	0.964047253	1.206173635	0.816393286
1.127413313	0.862627063	1.206532875	0.934291115
0.961921591	0.802985258	1.207061502	0.797232746
1.1629431	0.990341875	1.207066795	0.944423998
0.984488852	0.940790461	1.207148436	0.82947289
1.132285132	0.878482294	1.207415172	0.924633351
1.084365266	0.847789521	1.207513868	0.962723393
0.967537058	0.880908876	1.207627451	0.78449085
1.183484352	0.766406783	1.207758108	0.971722564

1.103975106	0.829286927	1.207846241	0.897747573
1.083163148	0.89070319	1.208353661	0.891756985
1.081204635	0.91049158	1.208429246	0.91992632
1.093464196	0.829624764	1.208505076	0.904534839
1.095420559	0.916682562	1.20869984	0.90644718
1.14522535	0.824495556	1.209081096	0.941053552
1.116367516	1.000971925	1.209132201	0.924341542
1.002836787	0.939905922	1.209250988	0.830118467
1.090520114	0.974588967	1.209334249	0.902579939
0.911711695	0.99266814	1.209629123	0.753972513
1.078676699	0.905812879	1.209958994	0.874641531
1.001115393	0.94511329	1.210143615	0.827269905
1.249908721	0.903078529	1.210451139	1.05519832
0.990311281	0.879480724	1.210721965	0.808005308
1.007765902	0.780143734	1.210743969	0.903033006
1.093633242	0.91997384	1.210748162	0.929097252
1.044200484	0.91999455	1.210819258	0.873424949
1.140462176	0.8863547	1.211331009	0.925883183
0.963643828	0.998684565	1.211412905	0.794899832
1.029179587	0.925704219	1.211853236	0.849802655
1.064739508	0.896584727	1.21194667	0.865181648
0.869119025	0.884224393	1.212025634	0.724440521
1.037951774	0.921676722	1.212059651	0.83570759
1.078626191	0.8971592	1.212160748	0.896773729
0.963066682	0.861077034	1.212237936	0.837949995
1.278801693	0.789714363	1.21237926	1.033656841
1.198250505	0.932823989	1.212462107	0.96545203
1.104541992	0.880789201	1.212658852	0.912616092
0.986350224	0.884816902	1.212742235	0.771794704
1.274901254	0.879300879	1.21279754	1.051119007
1.055057067	0.854502007	1.213111601	0.894686924
1.149761372	0.874450335	1.213233655	0.956552193
1.158226887	0.908767268	1.213411754	0.958946828
1.060177826	0.851960266	1.213604963	0.870053839
1.1577963	0.866082885	1.213648117	1.006430351
1.007591583	0.986250497	1.21368093	0.830330708
1.162090801	0.960619719	1.213791107	0.944651631
1.128936073	0.806537339	1.21383642	0.947438996
1.180241114	0.886449457	1.213959191	0.973490325
1.112154228	0.963073293	1.214091506	1.033514064
1.19918422	0.796051949	1.214233239	0.987606155
1.162145935	0.835347553	1.214762934	0.914927051
1.092538658	0.872021656	1.214889281	0.903468227
1.030092538	0.915004832	1.215028676	0.828087603
1.05879519	0.819477809	1.215124805	0.768894605
0.863274721	1.137215744	1.215171289	0.740228543

1.240798538	0.81538934	1.215186925	0.968631272
0.928732582	0.890986746	1.215298509	0.763965965
1.063247973	0.872827236	1.215593439	0.879564655
0.980250282	0.877892268	1.215903152	0.793427034
1.045028221	0.928396343	1.215924635	0.915625622
1.09973625	0.830434046	1.216345749	0.930516399
1.065536686	0.89838149	1.216640495	0.861324828
1.190185574	0.88273761	1.216727545	0.980788127
1.181421643	0.877059161	1.216964706	0.972694753
0.979613475	0.88568823	1.217051487	0.804247466
1.054591573	0.929908915	1.217212995	0.902718595
1.037432687	0.971411255	1.217414977	0.851038141
1.007137023	0.893927855	1.217492312	0.804104505
1.075899601	0.977673753	1.217496216	0.867089615
1.189052584	0.842557544	1.217541678	0.950509233
0.942323329	0.924085165	1.217582999	0.795467803
1.02832488	0.986457376	1.217611644	0.845115444
1.066920419	0.818296576	1.217651303	0.876130861
1.134306908	0.924488118	1.217816018	0.918788566
1.263340388	1.02654945	1.217844847	1.019098643
1.158367849	0.862801473	1.217853224	0.93875037
1.269909248	0.958549191	1.217873003	0.994627362
1.141031746	0.997002476	1.217958323	0.936558486
1.027003383	0.895312632	1.218218058	0.872963133
1.044566939	0.957969004	1.218350283	0.857361756
1.011447411	0.841340982	1.218513674	0.835646864
1.057511822	0.88746113	1.219078456	0.846295231
1.014877315	0.944268087	1.219240435	0.832451853
0.96809831	0.83117288	1.219490742	0.793849656
1.458041466	0.873187994	1.219649081	1.197193474
1.192302941	0.944308226	1.219709023	0.92468574
1.038801996	0.911791418	1.2199917	0.847046417
1.04773625	0.779993866	1.220175193	0.881235576
0.886055457	0.86707075	1.220591945	0.715894802
1.196567495	0.90829281	1.220909069	0.949124534
1.049895285	0.797306984	1.221021188	0.863631006
0.769640465	0.881761326	1.221173548	0.628311492
0.850138908	0.857053356	1.221333758	0.708560944
1.200204406	0.855937457	1.221727886	0.984262851
0.92874423	0.915117409	1.221759796	0.799965015
0.962593485	0.957578118	1.221959731	0.775912799
1.041021867	0.871071179	1.222233219	0.855703818
1.143712408	0.858928616	1.22227248	0.91956011
1.100060419	0.849608501	1.2222757	0.89528431
1.488997405	0.71538486	1.22235309	1.171354229
0.990260655	0.850953548	1.222375627	0.803025671

1.183016731	0.916818568	1.222401604	0.933928553
1.129472816	0.923944974	1.222479	0.911902147
0.958652396	0.923784348	1.222534789	0.895660393
0.982078862	0.982811873	1.222991326	0.800977067
1.119712357	0.850492768	1.222994791	0.89431583
1.026305439	0.821023469	1.223504892	0.927877989
0.90152513	0.911401153	1.223520122	0.705721345
1.065256691	0.883796287	1.223993677	0.916828283
1.105465522	0.909710274	1.224132394	0.899488341
1.081615122	0.942719	1.224238775	0.864319773
1.046797041	0.863866043	1.224296522	0.834150047
1.186078411	0.878990037	1.224489674	0.965980729
1.369654824	0.852442575	1.224884161	1.119422875
1.108816176	0.916074148	1.224897471	0.90577527
0.986354951	0.833960861	1.225002704	0.821967074
0.978820909	0.894463079	1.225021737	0.864130433
0.830074475	0.881523626	1.225483221	0.67055904
1.039482324	0.886374112	1.225592644	0.869728077
0.916391126	0.852274965	1.225751485	0.786743331
1.104489696	0.873239976	1.225809003	0.918373857
1.179983177	0.882594177	1.225858924	0.876160646
0.970850137	0.924122771	1.226700884	0.791215151
1.236510951	0.857460589	1.226911422	0.953053786
1.127864469	0.825409991	1.226995227	0.972705663
1.179260208	0.881255349	1.227047553	0.988786776
1.31830102	0.932743294	1.227222439	1.069725952
1.129782635	0.979639342	1.227345229	0.974705375
1.16563478	0.829141651	1.227395995	0.93561148
1.038380305	0.870384846	1.22740724	0.847092606
1.069623134	0.835130025	1.227553048	0.933642668
1.044615944	0.896510951	1.227560366	0.802850318
1.167008822	0.924619352	1.227623376	0.953274538
1.02400491	0.932715963	1.227633754	0.802850739
1.235880807	0.986992597	1.228131536	1.043775727
1.221661497	0.968413475	1.228181408	0.990343697
1.062913845	0.874528691	1.22869481	0.846188757
1.008395775	0.967974479	1.228708387	0.826048981
1.15628247	0.973334751	1.228710175	0.941205317
1.141505447	0.8655366	1.228743226	0.904928815
1.078595102	0.909709256	1.22891735	0.898560829
2.197267479	0.81197575	1.22907236	1.955287581
1.253394785	0.890537728	1.229228452	1.000847598
0.929086342	0.901682897	1.229290091	0.740002774
1.433826589	0.82021315	1.229333354	1.143603903
1.152854715	0.852757544	1.229673935	0.938387541
0.95749876	0.934014507	1.229792362	0.814465909

1.238321821	0.887747148	1.230073178	1.033423094
1.065312491	0.969925006	1.230328815	0.851347627
1.155599793	0.798159166	1.230434301	0.987782946
1.027294307	0.852389369	1.230656608	0.813631856
1.083540469	0.814222586	1.230741509	0.869455168
1.188714523	0.8254323	1.230829675	0.975566562
1.151847994	0.923007226	1.23088586	0.93580478
1.319429418	0.865365887	1.231006754	1.068869586
1.173598998	0.809271097	1.231233264	0.919106232
1.235767315	0.855225042	1.231490273	0.92001164
1.001771087	0.88734494	1.231490679	0.815526947
1.176971729	0.851921212	1.231521697	0.949937473
1.17629779	0.854091897	1.231712468	0.938941622
1.215122272	0.898258934	1.232026949	0.959502213
1.085741722	0.82977718	1.232499969	0.831889986
1.146944367	0.825983766	1.233349859	0.954179373
1.022817036	0.905530456	1.233366048	0.829289113
1.087984453	0.921206062	1.233468728	0.890071418
1.263752202	0.793208674	1.233473791	0.999003832
1.095120382	0.953764987	1.233842399	0.930067304
0.956881196	0.88321521	1.234172038	0.776661775
1.225603584	0.804877493	1.234340051	1.01904253
1.239748096	0.848674604	1.234371992	1.028728118
0.915733553	0.727602066	1.234472014	0.738499195
1.041368754	0.858871482	1.234660617	0.848489201
1.134075519	0.886641118	1.234791286	0.950192399
1.221749411	0.807145537	1.235749469	0.987784238
1.09972009	0.855205248	1.236198557	0.897030375
1.042462939	0.914487312	1.236260059	0.917635854
1.078177652	0.946118461	1.236551029	0.871300713
0.967346384	0.963328213	1.236551467	0.773610711
1.096478257	0.903819087	1.236896759	0.893274564
1.074010366	0.843037891	1.236901179	0.861959293
1.154090096	0.920876569	1.237151934	0.904433495
1.067482334	0.901174339	1.237256403	0.795333303
1.068408021	0.896565075	1.238119111	0.862928301
1.349330809	0.782381689	1.238204645	0.964234139
1.367663868	0.914454185	1.238395464	0.972713614
1.204428242	0.87873114	1.23840394	0.972379537
1.577758112	0.837152209	1.238491308	1.284264953
1.05227635	0.923360561	1.238857559	0.849059846
1.111987454	0.909653251	1.239368903	0.844748324
1.121269775	0.837205498	1.239393857	0.905042328
1.097765462	0.814049794	1.239644957	0.885548282
1.172037213	0.839371224	1.240046822	0.949924045
0.964961442	0.82146168	1.24016109	0.783778203

0.949736189	0.924705716	1.240563948	0.751692338
1.29064587	0.855559545	1.241018774	1.037393084
1.305001041	0.787303405	1.241056044	1.100211513
0.989286257	0.95071908	1.241151792	0.791308692
1.106111664	0.867846808	1.241357328	0.893992063
0.97911093	0.935356453	1.24139059	0.81363906
0.999774957	0.865776729	1.241433323	0.804170381
1.045624758	0.825529799	1.24144402	0.899092134
1.223157764	0.882894554	1.241467995	1.016537713
1.201146127	0.83632133	1.241672144	0.953699071
1.158643592	0.925664705	1.241693247	1.093270472
1.106565211	0.853958867	1.242723943	0.877925904
1.147367152	1.002922564	1.243012955	0.964838065
1.177708284	0.88720268	1.243289493	0.968297973
1.008148638	0.932825245	1.243542663	0.80751322
1.044242473	0.897878913	1.243583201	0.831344251
1.187116903	0.932851858	1.243614519	1.033159223
1.171312881	0.938285278	1.243657951	0.924881888
1.351756804	0.966819606	1.243990011	1.074138237
1.174935845	0.891933454	1.244376081	0.991376385
1.118750633	0.863051757	1.244528699	0.924263566
1.056979675	0.933869878	1.245363631	0.831518858
1.010592053	0.904593368	1.245506369	0.80344224
1.104415125	0.855864994	1.245826435	0.888849902
0.939561301	0.905561441	1.246067563	0.733997822
1.129366387	0.939589112	1.246091386	0.830389334
1.173335517	0.904132275	1.246616114	0.961245545
1.020646103	0.83351144	1.246618592	0.823233534
1.100636399	0.959732849	1.247047164	0.963393883
0.96341909	0.910483312	1.247671863	0.770667623
1.090444568	0.801399561	1.247724502	0.928587072
0.90871242	0.853492385	1.248163727	0.694678713
0.943600254	1.063349975	1.248468641	0.736503611
1.060809094	0.89238422	1.24871067	0.853683513
1.157017885	0.800060366	1.249293587	0.938579243
1.062099313	0.911171513	1.249315364	0.900147047
1.101517514	0.840099359	1.249372686	0.892491808
1.074599781	0.804505085	1.249525375	0.896694153
1.010609121	0.895893954	1.249580238	0.790195471
1.195690134	0.943680238	1.249645782	0.957358312
1.178857822	0.792404812	1.249661768	0.938890585
1.079057889	0.936631705	1.249818599	0.892394928
1.183111803	0.927646605	1.250147637	0.965029277
1.12202539	0.890226145	1.250319751	0.920689149
1.233004138	0.73061933	1.250485347	1.003826278
1.049217538	0.877444199	1.250633192	0.886851211

1.10913421	0.979838032	1.251478802	0.890330381
1.007748721	0.884788691	1.251587285	0.807868082
1.175994794	0.946447497	1.251813037	0.915148371
1.118454276	0.953368328	1.251848889	0.894403501
1.079096647	0.883916318	1.251899864	0.859849936
0.875111598	1.03857307	1.252270471	0.74977421
1.064024403	0.871723584	1.252699667	0.860980048
1.013558122	0.707370501	1.252722519	0.809682091
1.187839889	0.859856911	1.253120424	0.947882407
1.12115284	0.840179483	1.253200276	0.884508753
1.229084357	0.886529538	1.253986007	0.977230007
0.974062514	0.843211836	1.254677126	0.798326253
1.188295328	0.897834451	1.254738323	0.930219005
1.024137934	0.768613142	1.254889284	0.755746312
1.024506826	0.994354784	1.255038781	0.821623931
1.046520771	0.888559181	1.255145616	0.834756517
1.142817717	0.897595797	1.255426983	0.928386025
1.218913637	0.804954245	1.255670093	0.954077273
1.036429312	0.926231983	1.255683996	0.863318334
1.000107983	0.814683508	1.255852795	0.751987009
1.097364823	0.933316545	1.256044253	0.861615818
1.052471654	0.893007117	1.256186094	0.865954429
1.034315164	0.892432972	1.256315305	0.822292877
1.198449158	0.793192805	1.256763609	0.954566809
0.992181835	0.832254491	1.256924498	0.78416519
1.206716644	0.879590215	1.256983464	0.969887341
1.060112079	0.956849514	1.257016066	0.827192173
1.020822973	0.904856584	1.257569559	0.81897992
1.054780115	0.920213591	1.25810048	0.916711476
1.117555612	0.914047501	1.258177203	0.901490216
1.452946593	0.81744134	1.259689233	1.101937787
1.335051497	0.916003464	1.259956822	1.045963855
1.04870035	0.865532451	1.260193394	0.901718887
1.14982899	0.882942242	1.260338701	0.922162113
1.141099364	0.867656996	1.260608515	0.896987869
1.139543303	0.919159719	1.260627077	0.917867742
1.16201738	0.831286143	1.261361901	0.902107529
1.215140598	0.71079353	1.261701748	0.938711712
1.032163922	0.917871374	1.262414776	0.803068051
1.004060366	0.899040165	1.262447237	0.849646644
1.210321618	0.89831905	1.263174894	0.973700212
0.547694045	0.845053713	1.263792885	0.442958169
1.09366075	0.873692357	1.264185648	0.876021985
0.930556959	0.930217941	1.264289823	0.768910672
1.026072378	0.914623866	1.265422593	0.784572156
1.085595911	0.810138728	1.265436238	0.852885008

1.205506308	0.898313323	1.265715792	0.896294114
1.126088776	0.792152461	1.265933042	0.937192724
1.081572237	0.861435897	1.266489297	0.850055149
1.230567809	1.003292585	1.266751544	0.944803923
1.183150536	0.818758322	1.267142561	0.934938975
1.205389769	0.815277992	1.267471928	0.934573038
1.175016244	0.856014308	1.267906356	0.960033961
1.152802474	0.845491133	1.268148251	0.969465253
1.127071581	0.917979151	1.26849809	0.916055336
1.145048859	0.811125681	1.268901644	0.884652172
1.114878578	0.846675941	1.270260625	0.877034546
1.173900413	0.868444596	1.270308573	0.935691237
1.235863209	0.774000747	1.27082792	0.972954744
1.153320772	0.86815374	1.272515723	0.8951746
1.123525841	0.950418614	1.272676495	0.882314622
1.246289305	0.95392278	1.273138915	0.98011021
1.231533112	0.892277995	1.273307159	0.909434475
1.200065646	0.920746748	1.273317996	0.936537947
1.185873189	0.883195502	1.273370534	0.951757303
1.056173084	0.831492435	1.273686518	0.828299384
0.979618967	0.926032566	1.274098295	0.768396166
1.098844507	0.841059295	1.274351445	0.868064666
1.020611333	0.950219523	1.274374172	0.797631392
0.959238464	0.971105654	1.274606117	0.806429358
1.079923799	0.924240344	1.274980586	0.830431148
0.916451236	0.922666968	1.275013896	0.673065711
1.089469637	0.988321993	1.275060815	0.859053664
1.225940816	0.808299455	1.275223909	0.96129866
0.947359734	0.840496143	1.276059298	0.746291763
0.961667983	0.894167468	1.276252375	0.839072469
1.117404614	0.817649953	1.276407759	0.820967383
1.085200636	0.878050611	1.276863438	0.861070353
0.872068725	0.966390347	1.277433393	0.682890009
1.173169516	0.884839391	1.277889202	0.906184954
0.953747424	0.928977163	1.27800469	0.699345309
1.210906608	0.860605121	1.278862083	0.984130605
1.106917229	0.897139844	1.279278596	0.87045753
0.954595652	0.891891801	1.27939027	0.708463342
1.019173779	0.856263292	1.279604292	0.886013105
1.064084181	0.943759844	1.279615242	0.870180577
1.020555762	0.883835571	1.279811158	0.798850709
1.212545386	0.764638572	1.280335044	0.94316911
1.109476753	0.893442813	1.280527257	0.852357405
1.151227933	0.807829921	1.281777393	0.873074165
1.014521097	0.925864166	1.28199667	0.791637378
1.118253663	0.864131174	1.283312985	0.871416023

1.14086238	0.84837947	1.28387781	0.994004011
1.073146135	0.986729268	1.2841163	0.879170818
0.920278115	0.939962191	1.284229183	0.782069559
1.180880305	0.885865894	1.284755466	0.965682065
1.071951442	0.868289526	1.285517554	0.82614113
1.116809357	0.815512469	1.287212764	0.847697334
1.061066563	0.882765876	1.288778685	0.846126433
1.107898984	0.854138323	1.289109376	0.871910242
1.053894148	0.905194693	1.289641578	0.803561912
1.093564947	0.780355087	1.289679591	0.92432384
1.030969817	0.920116974	1.289843039	0.806281039
1.301960179	0.857209313	1.290156996	1.028099651
1.056447971	0.731798897	1.290382542	0.857621796
1.051612155	0.881563289	1.29072353	0.907174698
1.17747818	0.942274492	1.292155042	0.920704493
1.165896874	0.791705877	1.292703925	0.85003923
1.068809035	0.866892952	1.292996579	0.822422518
1.176232489	0.787866038	1.293786548	0.909187486
1.260259924	0.975459152	1.294118148	0.974139554
1.118954622	0.850838499	1.294162523	0.866355013
0.978103097	0.85137758	1.294910034	0.785422662
1.10260619	0.965502781	1.295215293	0.851489136
1.130024914	0.7461646	1.295286131	0.8478515
1.259927446	0.846824352	1.296125689	0.971925179
1.151947219	0.822533921	1.296327102	0.951599607
1.146191024	0.94315784	1.2969353	0.887460325
1.121889499	0.952510727	1.297021437	0.853656922
1.180711717	0.816840779	1.29813471	0.966837691
1.174295293	0.757174592	1.298898875	0.904287916
1.343405483	0.858331706	1.299030283	1.034106718
1.035138045	0.816229911	1.299244171	0.977788123
1.338157831	0.868945967	1.3023006	1.02344751
0.94192853	0.920288087	1.30313648	0.722433715
1.177868792	0.872483997	1.304372657	0.895055176
1.040825883	0.882037785	1.305152442	0.821956362
1.253270394	0.817993565	1.305294781	0.95797245
0.722762223	0.835078816	1.305693972	0.55924592
1.037385067	0.792128541	1.305937936	0.799329636
1.143065088	0.855455597	1.306697095	0.856064774
1.231409319	0.86340357	1.310577688	0.92463206
1.033970531	0.812432704	1.311229249	0.837669623
1.09693211	0.897266994	1.311680398	0.846547102
0.965546875	0.944873998	1.313192753	0.733242505
1.030187183	0.8560153	1.313402249	0.753535571
1.272513748	0.854311227	1.314431093	0.968163279
1.15752734	0.83081809	1.314581214	0.85497626

1.180138176	0.966067638	1.316362782	0.936356627
1.00145185	0.892182242	1.317019164	0.749812174
1.152188302	1.033547412	1.321990619	0.83907075
1.153984947	0.793310363	1.325164981	0.947386235
1.088212932	0.950385033	1.325924499	0.728212865
1.20983979	0.851426195	1.328353785	1.022562045
0.874284502	0.940849608	1.330622355	0.678299329
1.114327759	0.744915452	1.332954268	0.845245678
1.114616921	0.792293224	1.33485504	0.838811078
1.199513991	0.840010429	1.335016788	0.897234729
0.895467697	0.911662123	1.336550711	0.66851665
0.752007862	1.021946514	1.337971	0.567000976
1.083511317	0.868511863	1.342291793	0.849870169
1.150943374	0.867508466	1.345988285	0.875691963
1.051143605	0.848593759	1.349017207	0.779646291
1.039302367	0.915497203	1.3539695	0.857488095
1.001867746	0.826602176	1.357395237	0.738073871
1.159219144	0.929184248	1.362424512	0.851156559
1.146510632	0.885580219	1.36293747	0.848021164
1.144517318	0.896774137	1.363355197	0.817137927
1.236514708	0.845394911	1.366612645	0.904660813
0.809054023	1.237545639	1.367628918	0.60005383
0.888055819	0.915739298	1.370057855	0.648081495
1.076658451	0.920140512	1.374116175	0.767041885
1.188988552	0.897712705	1.375909229	0.828880204
1.02209068	0.948484657	1.378885797	0.741736088
0.877869712	0.93085634	1.382730284	0.665124229
1.087216875	0.873979856	1.407070704	0.772578646
1.181260567	0.661423722	1.44200753	0.803042653
1.07760125	0.991086282	1.442050309	0.773898949
1.048661541	0.832892751	1.448129299	0.734856484
0.893926456	1.514880157	1.505887787	0.545513941
1.218702951	0.869451937	1.514982916	0.84002009
1.206572579	0.867608971	1.54055718	0.780200841
1.394666741	0.726885628	1.5410447	0.960111834
1.355740192	0.80800955	1.723362484	0.791075206
