# Supplementary material for: Absence of Wdr13 Gene Predisposes Mice to Mild Social Isolation – Chronic Stress, Leading to Depression-Like Phenotype Associated With Differential Expression of Synaptic Proteins
Source: Front Mol Neurosci. 2018 Apr 25;11:133. doi: 10.3389/fnmol.2018.00133 (PMC5930177; doi:10.3389/fnmol.2018.00133)
Supplement: TABLE S3 — Delineation of downregulated proteins (at-least two unique peptides) from PFC of Wdr13-/0 mice after social isolation into Biological Processes using String analysis. [file Table_3.PDF]

#pathway ID	pathway description	observed ger	false discovery rate
GO.0050804	modulation of synaptic transmission	18	1.67E-10
GO.0051128	regulation of cellular component organization	39	2.66E-07
GO.0007268	synaptic transmission	16	4.77E-07
GO.0065008	regulation of biological quality	43	5.13E-07
GO.0032970	regulation of actin filament-based process	15	6.66E-07
GO.0016192	vesicle-mediated transport	24	2.91E-06
GO.0051049	regulation of transport	31	4.22E-06
GO.0048167	regulation of synaptic plasticity	10	5.77E-06
GO.0010499	proteasomal ubiquitin-independent protein cata	6	8.36E-06
GO.0050803	regulation of synapse structure or activity	12	8.36E-06
GO.0051649	establishment of localization in cell	28	8.36E-06
GO.0009987	cellular process	100	9.01E-06
GO.0048168	regulation of neuronal synaptic plasticity	7	1.16E-05
GO.0048489	synaptic vesicle transport	9	1.27E-05
GO.0043278	response to morphine	6	1.42E-05
GO.0097479	synaptic vesicle localization	9	1.42E-05
GO.0051641	cellular localization	31	1.61E-05
GO.0007267	cell-cell signaling	17	1.62E-05
GO.0016043	cellular component organization	51	1.78E-05
GO.0007399	nervous system development	32	1.80E-05
GO.0023051	regulation of signaling	37	1.80E-05
GO.0043269	regulation of ion transport	17	1.89E-05
GO.0046907	intracellular transport	23	1.89E-05
GO.0032956	regulation of actin cytoskeleton organization	12	2.07E-05
GO.0030003	cellular cation homeostasis	14	2.23E-05
GO.0032879	regulation of localization	34	2.45E-05
GO.0051493	regulation of cytoskeleton organization	14	2.98E-05
GO.0055080	cation homeostasis	15	3.54E-05
GO.0007154	cell communication	47	4.47E-05
GO.0044700	single organism signaling	46	4.47E-05
GO.0010646	regulation of cell communication	37	4.50E-05
GO.0098771	inorganic ion homeostasis	15	4.57E-05
GO.0051179	localization	49	4.59E-05
GO.0055082	cellular chemical homeostasis	15	4.75E-05
GO.0051130	positive regulation of cellular component organiz	23	5.10E-05
GO.0010959	regulation of metal ion transport	12	6.57E-05
GO.0007215	glutamate receptor signaling pathway	6	6.64E-05
GO.0035249	synaptic transmission, glutamatergic	6	6.64E-05
GO.0044708	single-organism behavior	14	6.64E-05
GO.0007270	neuron-neuron synaptic transmission	7	7.14E-05
GO.0044087	regulation of cellular component biogenesis	17	7.27E-05
GO.0019725	cellular homeostasis	16	7.54E-05
GO.0050789	regulation of biological process	79	9.36E-05
GO.0022607	cellular component assembly	27	0.000101
GO.0051656	establishment of organelle localization	11	0.000106

GO.0051495	positive regulation of cytoskeleton organization	9	0.00011
GO.0065007	biological regulation	81	0.000117
GO.0060359	response to ammonium ion	7	0.000127
GO.0006875	cellular metal ion homeostasis	12	0.000146
GO.1902578	single-organism localization	36	0.000154
GO.0055065	metal ion homeostasis	13	0.000156
GO.0006897	endocytosis	13	0.000157
GO.0051640	organelle localization	12	0.000157
GO.0007610	behavior	15	0.000191
GO.0034613	cellular protein localization	20	0.000191
GO.0006836	neurotransmitter transport	8	0.000206
GO.0048699	generation of neurons	23	0.000216
GO.0051588	regulation of neurotransmitter transport	6	0.000243
GO.0051234	establishment of localization	40	0.00029
GO.0071822	protein complex subunit organization	21	0.000304
GO.0034248	regulation of cellular amide metabolic process	11	0.000319
GO.0043933	macromolecular complex subunit organization	27	0.000319
GO.0060341	regulation of cellular localization	21	0.000337
GO.0008104	protein localization	25	0.000339
GO.0051924	regulation of calcium ion transport	9	0.000442
GO.0030838	positive regulation of actin filament polymerizati	6	0.000457
GO.0006874	cellular calcium ion homeostasis	10	0.000463
GO.0044085	cellular component biogenesis	27	0.000515
GO.0033036	macromolecule localization	27	0.00054
GO.0022008	neurogenesis	23	0.000595
GO.0006816	calcium ion transport	9	0.000642
GO.0006810	transport	38	0.000643
GO.0031344	regulation of cell projection organization	13	0.000643
GO.0048878	chemical homeostasis	17	0.000643
GO.0050767	regulation of neurogenesis	15	0.000643
GO.0030833	regulation of actin filament polymerization	7	0.00077
GO.0051960	regulation of nervous system development	16	0.000824
GO.0050768	negative regulation of neurogenesis	9	0.000882
GO.0044763	single-organism cellular process	80	0.00101
GO.1903530	regulation of secretion by cell	14	0.00101
GO.0010608	posttranscriptional regulation of gene expressior	11	0.00106
GO.0036465	synaptic vesicle recycling	4	0.00106
GO.0044089	positive regulation of cellular component biogen	11	0.00106
GO.0044765	single-organism transport	32	0.00106
GO.0051716	cellular response to stimulus	49	0.00106
GO.1901215	negative regulation of neuron death	8	0.00106
GO.0043279	response to alkaloid	7	0.00118
GO.0007626	locomotory behavior	9	0.00121
GO.0042592	homeostatic process	21	0.00123
GO.0046928	regulation of neurotransmitter secretion	5	0.00125
GO.0007265	Ras protein signal transduction	8	0.00161

GO.0033043	regulation of organelle organization	19	0.00161
GO.0060627	regulation of vesicle-mediated transport	11	0.00167
GO.0035235	ionotropic glutamate receptor signaling pathway	4	0.00169
GO.0043254	regulation of protein complex assembly	10	0.00169
GO.1901214	regulation of neuron death	9	0.00177
GO.0043161	proteasome-mediated ubiquitin-dependent prot	9	0.00181
GO.0048169	regulation of long-term neuronal synaptic plastic	4	0.00195
GO.0048731	system development	41	0.00203
GO.0007165	signal transduction	39	0.00205
GO.0006886	intracellular protein transport	13	0.00239
GO.0035556	intracellular signal transduction	21	0.0024
GO.0045176	apical protein localization	3	0.0024
GO.0006461	protein complex assembly	16	0.00258
GO.0070271	protein complex biogenesis	16	0.00258
GO.0065003	macromolecular complex assembly	18	0.00268
GO.1901698	response to nitrogen compound	15	0.00282
GO.0010243	response to organonitrogen compound	14	0.00317
GO.0023056	positive regulation of signaling	21	0.00317
GO.0044707	single-multicellular organism process	51	0.00331
GO.0048856	anatomical structure development	45	0.00342
GO.0071702	organic substance transport	23	0.00343
GO.0010647	positive regulation of cell communication	22	0.00353
GO.1902580	single-organism cellular localization	14	0.00358
GO.0017157	regulation of exocytosis	7	0.00381
GO.0010975	regulation of neuron projection development	10	0.00386
GO.0007417	central nervous system development	16	0.00396
GO.0007275	multicellular organismal development	44	0.00404
GO.0030534	adult behavior	7	0.00404
GO.0048172	regulation of short-term neuronal synaptic plasti	3	0.00404
GO.2000474	regulation of opioid receptor signaling pathway	2	0.00404
GO.0016079	synaptic vesicle exocytosis	5	0.00409
GO.0019722	calcium-mediated signaling	5	0.00409
GO.0032535	regulation of cellular component size	9	0.00409
GO.0006417	regulation of translation	9	0.00414
GO.0050896	response to stimulus	54	0.00418
GO.0010638	positive regulation of organelle organization	13	0.00435
GO.0030030	cell projection organization	16	0.00448
GO.0044767	single-organism developmental process	48	0.0045
GO.0019932	second-messenger-mediated signaling	6	0.00563
GO.0006511	ubiquitin-dependent protein catabolic process	10	0.00591
GO.0048518	positive regulation of biological process	48	0.00632
GO.0007010	cytoskeleton organization	15	0.00657
GO.0030001	metal ion transport	12	0.00691
GO.0045184	establishment of protein localization	18	0.00692
GO.0022604	regulation of cell morphogenesis	11	0.00738
GO.0051259	protein oligomerization	10	0.00738

GO.0048519	negative regulation of biological process	42	0.00753
GO.0010592	positive regulation of lamellipodium assembly	3	0.00761
GO.0048468	cell development	22	0.00766
GO.0048869	cellular developmental process	37	0.00766
GO.0008150	biological_process	94	0.00822
GO.0001505	regulation of neurotransmitter levels	6	0.00882
GO.0050770	regulation of axonogenesis	6	0.00882
GO.0009628	response to abiotic stimulus	16	0.00901
GO.0006904	vesicle docking involved in exocytosis	4	0.0102
GO.0048488	synaptic vesicle endocytosis	3	0.011
GO.0030154	cell differentiation	35	0.0117
GO.0034250	positive regulation of cellular amide metabolic pr	5	0.0119
GO.0007269	neurotransmitter secretion	5	0.0126
GO.1902582	single-organism intracellular transport	15	0.0128
GO.0050794	regulation of cellular process	68	0.013
GO.0045664	regulation of neuron differentiation	11	0.0133
GO.0007420	brain development	13	0.0135
GO.0061024	membrane organization	12	0.0151
GO.2000300	regulation of synaptic vesicle exocytosis	3	0.0151
GO.0031175	neuron projection development	11	0.0155
GO.0040008	regulation of growth	12	0.0155
GO.0043524	negative regulation of neuron apoptotic process	6	0.0163
GO.0016482	cytoplasmic transport	11	0.0171
GO.0008344	adult locomotory behavior	5	0.0174
GO.1904062	regulation of cation transmembrane transport	6	0.0174
GO.0008152	metabolic process	69	0.0182
GO.0044802	single-organism membrane organization	11	0.0186
GO.0031346	positive regulation of cell projection organization	8	0.0191
GO.0021700	developmental maturation	7	0.0194
GO.0044257	cellular protein catabolic process	10	0.0197
GO.0007015	actin filament organization	6	0.0203
GO.0010769	regulation of cell morphogenesis involved in diffe	8	0.0203
GO.0006996	organelle organization	29	0.0211
GO.0015031	protein transport	16	0.0213
GO.0060322	head development	13	0.0213
GO.0031334	positive regulation of protein complex assembly	6	0.0222
GO.0007611	learning or memory	7	0.023
GO.0048523	negative regulation of cellular process	38	0.0237
GO.0032412	regulation of ion transmembrane transporter act	6	0.0245
GO.0051125	regulation of actin nucleation	3	0.0245
GO.0007632	visual behavior	4	0.025
GO.0010256	endomembrane system organization	9	0.025
GO.0008277	regulation of G-protein coupled receptor protein	5	0.0259
GO.0050806	positive regulation of synaptic transmission	5	0.0259
GO.0051092	positive regulation of NF-kappaB transcription fa	5	0.0259
GO.1901575	organic substance catabolic process	17	0.0295

GO.0030182	neuron differentiation	14	0.0309
GO.0009719	response to endogenous stimulus	17	0.0312
GO.0010033	response to organic substance	24	0.0332
GO.0001558	regulation of cell growth	8	0.0357
GO.0048666	neuron development	12	0.0357
GO.0065009	regulation of molecular function	27	0.0363
GO.0002026	regulation of the force of heart contraction	3	0.0368
GO.0007616	long-term memory	3	0.0368
GO.0015991	ATP hydrolysis coupled proton transport	3	0.0368
GO.1902430	negative regulation of beta-amyloid formation	2	0.0368
GO.0050808	synapse organization	5	0.0408
GO.0009605	response to external stimulus	20	0.0439
GO.0044093	positive regulation of molecular function	19	0.0449
GO.0042325	regulation of phosphorylation	17	0.0483
GO.0031641	regulation of myelination	3	0.0493
GO.2000601	positive regulation of Arp2/3 complex-mediated	2	0.0493

matching prc	matching proteins in your network (labels)
ENSMUSP00	Atp2a2,Calb1,Camk2a,Cplx2,Gnai1,Gpm6b,Lgi1,Mgll,Napa,Ppp1r9b,Prkce,Rab3a
ENSMUSP00	Aak1,Actr3,Arpc4,Atp8a1,Brk1,Cd81,Cdh2,Clu,Coro1a,Crk,Ctnnb1,Cttn,Gpm6b,G
ENSMUSP00	Cacnb4,Cplx2,Ctnnb1,Dnm1,Gria2,Gria3,Grin1,Napa,Pclo,Prkcg,Rab8a,Slc17a7,S
ENSMUSP00	Aak1,Actr3,App,Arpc4,Atp8a1,Cacnb4,Calb1,Camk2a,Ccdc109a,Cdh2,Clu,Coro1a
ENSMUSP00	Actr3,Arpc4,Atp1a1,Atp2a2,Brk1,Coro1a,Crk,Cttn,Gpm6b,Mtor,Nckap1,Prkce,Rh
ENSMUSP00	Aak1,Ap1g1,Ap2a1,Ap2s1,Ap3b2,App,Coro1a,Cplx2,Ctnnb1,Cttn,Hook3,Icam5,N
ENSMUSP00	Aak1,Ap1g1,Atp1a1,Atp2a2,Atp8a1,Cacnb4,Camk2a,Ccdc109a,Coro1a,Ctnnb1,G
ENSMUSP00	Calb1,Camk2a,Cplx2,Grin1,Mgll,Rab3a,Rab8a,Syn1,Syngap1,Syp
ENSMUSP00	Psma2,Psma3,Psma4,Psma5,Psmb1,Psmb6
ENSMUSP00	App,Calb1,Camk2a,Cdh2,Cplx2,Grin1,Mgll,Rab3a,Rab8a,Syn1,Syngap1,Syp
ENSMUSP00	Actr3,Ap1g1,Ap2a1,Ap2s1,Ap3b2,App,Camk2a,Ccdc109a,Coro1a,Cplx2,Ctnnb1,C
ENSMUSP00	Aak1,Actr3,Ap1g1,Ap3b2,App,Atp6v1a,Atp8a1,Bpnt1,Brk1,Cab39,Cacnb4,Camk
ENSMUSP00	Camk2a,Grin1,Rab3a,Rab8a,Syn1,Syngap1,Syp
ENSMUSP00	Ap3b2,Cplx2,Ctnnb1,Pacsin1,Pclo,Rab3a,Rab8a,Sh3gl2,Syt1
ENSMUSP00	Grin1,Ppp1r9b,Ppp5c,Prkce,Prkcg,Srr
ENSMUSP00	Ap3b2,Cplx2,Ctnnb1,Pacsin1,Pclo,Rab3a,Rab8a,Sh3gl2,Syt1
ENSMUSP00	Actr3,Ap1g1,Ap2a1,Ap2s1,Ap3b2,App,Camk2a,Ccdc109a,Cdh2,Coro1a,Cplx2,Ctr
ENSMUSP00	Cacnb4,Cplx2,Ctnnb1,Dnm1,Gria2,Gria3,Grin1,Napa,Pclo,Prkcg,Rab8a,Ryr2,Slc1
ENSMUSP00	Actr3,App,Atp2a2,Atp6v0d1,Atp8a1,Brk1,Cacnb4,Camk2a,Camk2g,Ccdc109a,Cd
ENSMUSP00	App,Atp6v0d1,Camk2g,Cdh2,Clu,Cplx2,Ctnnb1,Cttn,Grin1,Hook3,Lgi1,Lingo1,Mg
ENSMUSP00	Aak1,App,Atp2a2,Calb1,Camk2a,Ccdc109a,Cd81,Clu,Cplx2,Crk,Ctnnb1,Cttn,Epha
ENSMUSP00	Atp1a1,Atp8a1,Cacnb4,Camk2a,Coro1a,Ctnnb1,Gnb5,Gpm6b,Grin1,Homer1,Pcp
ENSMUSP00	Ap1g1,Ap2a1,Ap2s1,Ap3b2,App,Camk2a,Ccdc109a,Coro1a,Cplx2,Cttn,Hnrnpa1,
ENSMUSP00	Actr3,Arpc4,Brk1,Coro1a,Crk,Cttn,Gpm6b,Mtor,Nckap1,Prkce,Rhoa,Synpo
ENSMUSP00	App,Atp1a1,Atp2a2,Cacnb4,Calb1,Ccdc109a,Gnb1,Grin1,Prkcb,Prkce,Ryr2,Slc17
ENSMUSP00	Aak1,Actn1,Ap1g1,Atp8a1,Cacnb4,Camk2a,Ccdc109a,Cd81,Cdh2,Coro1a,Ctnnb1
ENSMUSP00	Actr3,Arpc4,Brk1,Coro1a,Crk,Ctnnb1,Cttn,Gpm6b,Mtor,Nckap1,Npm1,Prkce,Rh
ENSMUSP00	App,Atp1a1,Atp2a2,Cacnb4,Calb1,Ccdc109a,Gnb1,Grin1,Prkcb,Prkce,Ryr2,Sfxn3
ENSMUSP00	App,Atp2a2,Brk1,Cab39,Cacnb4,Camk2a,Ccdc109a,Clu,Coro1a,Cplx2,Crk,Ctnnb1
ENSMUSP00	App,Atp2a2,Brk1,Cab39,Cacnb4,Camk2a,Ccdc109a,Clu,Coro1a,Cplx2,Crk,Ctnnb1
ENSMUSP00	Aak1,App,Atp2a2,Calb1,Camk2a,Ccdc109a,Cd81,Clu,Cplx2,Crk,Ctnnb1,Cttn,Epha
ENSMUSP00	App,Atp1a1,Atp2a2,Cacnb4,Calb1,Ccdc109a,Gnb1,Grin1,Prkcb,Prkce,Ryr2,Sfxn3
ENSMUSP00	Aak1,Actr3,Ap2s1,Ap3b2,App,Atp6v1a,Atp8a1,Brk1,Cacnb4,Camk2a,Camk2g,Cc
ENSMUSP00	App,Atp1a1,Atp2a2,Cacnb4,Calb1,Ccdc109a,Gnb1,Grin1,Hk1,Prkcb,Prkce,Ryr2,S
ENSMUSP00	Actr3,Arpc4,Atp8a1,Brk1,Cdh2,Clu,Ctnnb1,Cttn,Epha4,Mtor,Nckap1,Negr1,Npm
ENSMUSP00	Atp1a1,Cacnb4,Camk2a,Coro1a,Ctnnb1,Gnb5,Homer1,Plcb1,Prkce,Rhoa,Ryr2,Y
ENSMUSP00	App,Gria2,Gria3,Grin1,Homer1,Plcb1
ENSMUSP00	Cacnb4,Gria2,Gria3,Grin1,Napa,Slc17a7
ENSMUSP00	App,Atp8a1,Cacnb4,Calb1,Dnm1,Epha4,Grin1,Homer1,Plcb1,Prkce,Slc17a7,Slc1
ENSMUSP00	Cacnb4,Dnm1,Gria2,Gria3,Grin1,Napa,Slc17a7
ENSMUSP00	Actr3,Arpc4,Brk1,Cdh2,Clu,Coro1a,Cttn,Gpm6b,Grin1,Mtor,Napa,Nckap1,Npm1
ENSMUSP00	App,Atp1a1,Atp2a2,Cacnb4,Calb1,Ccdc109a,Gnb1,Grin1,Hk1,Npm1,Prkcb,Prkce
ENSMUSP00	Aak1,Actn1,Actr3,Ap1g1,Ap2a1,Armc10,Arpc4,Atp8a1,Brk1,Cab39,Cacnb4,Calb
ENSMUSP00	Actn1,App,Arpc4,Atp6v0d1,Brk1,Camk2g,Ccdc109a,Cdh2,Clu,Coro1a,Ctnnb1,Dn
ENSMUSP00	Actr3,Ap3b2,Cplx2,Ctnnb1,Npm1,Pacsin1,Pclo,Rab3a,Rab8a,Sh3gl2,Syt1

ENSMUSP00(	Actr3,Arpc4,Brk1,Cttn,Mtor,Nckap1,Npm1,Prkce,Synpo
ENSMUSP00(	Aak1,Actn1,Actr3,Ap1g1,Ap2a1,Armc10,Arpc4,Atp8a1,Brk1,Cab39,Cacnb4,Calb1
ENSMUSP00(	Grin1,Homer1,Ppp1r9b,Ppp5c,Prkce,Prkcg,Srr
ENSMUSP00(	App,Atp1a1,Atp2a2,Cacnb4,Calb1,Ccdc109a,Gnb1,Grin1,Prkcb,Prkce,Ryr2,Tfrc
ENSMUSP00(	Actr3,Ap1g1,Ap3b2,App,Atp6v1a,Atp8a1,Cacnb4,Camk2a,Camk2g,Ccdc109a,Cd
ENSMUSP00(	App,Atp1a1,Atp2a2,Cacnb4,Calb1,Ccdc109a,Gnb1,Grin1,Prkcb,Prkce,Ryr2,Sfxn3
ENSMUSP00(	Aak1,Ap2a1,Ap2s1,App,Coro1a,Cttn,Icam5,Pacsin1,Rab14,Sh3gl2,Syp,Syt1,Tfrc
ENSMUSP00(	Actr3,Ap3b2,Cplx2,Ctnnb1,Hook3,Npm1,Pacsin1,Pclo,Rab3a,Rab8a,Sh3gl2,Syt1
ENSMUSP00(	App,Atp8a1,Cacnb4,Calb1,Dnm1,Epha4,Grin1,Homer1,Negr1,Plcb1,Prkce,Slc17a
ENSMUSP00(	Ap1g1,Ap2a1,Ap2s1,Ap3b2,Cdh2,Ctnnb1,Cttn,Hook3,Napa,Pacs1,Pacsin1,Rab14
ENSMUSP00(	Cplx2,Pclo,Rab8a,Slc17a7,Slc6a17,Sv2b,Syn1,Syt1
ENSMUSP00(	App,Cdh2,Ctnnb1,Cttn,Grin1,Hook3,Lgi1,Lingo1,Mgll,Napa,Negr1,Olfm1,Pacsin1
ENSMUSP00(	Atp2a2,Camk2a,Gpm6b,Napa,Rab3a,Stx1a
ENSMUSP00(	Aak1,Actr3,Ap2s1,Ap3b2,App,Atp6v1a,Atp8a1,Cacnb4,Camk2a,Camk2g,Ccdc10
ENSMUSP00(	Actr3,App,Brk1,Camk2g,Ccdc109a,Cdh2,Clu,Coro1a,Cttn,Dnm1,Grin1,Hook3,Na
ENSMUSP00(	App,Clu,Eif3a,Eif3f,Mtor,Npm1,Olfm1,Pcbp1,Rhoa,Rps3,Vars
ENSMUSP00(	Actr3,App,Brk1,Camk2g,Ccdc109a,Cdh2,Clu,Coro1a,Ctnnb1,Cttn,Dnm1,Eif3a,Eif
ENSMUSP00(	Ap1g1,Atp2a2,Camk2a,Ccdc109a,Cdh2,Coro1a,Ctnnb1,Gpm6b,Mtor,Napa,Pclo,
ENSMUSP00(	Ap2s1,Ap3b2,Cd81,Cdh2,Clu,Ctnnb1,Cttn,Gpm6b,Homer1,Hook3,Napa,Nckap1,
ENSMUSP00(	Cacnb4,Camk2a,Coro1a,Ctnnb1,Gnb5,Homer1,Prkce,Rhoa,Ryr2
ENSMUSP00(	Actr3,Arpc4,Cttn,Mtor,Prkce,Rhoa
ENSMUSP00(	App,Atp2a2,Cacnb4,Calb1,Ccdc109a,Gnb1,Grin1,Prkcb,Prkce,Ryr2
ENSMUSP00(	Actn1,App,Arpc4,Atp6v0d1,Brk1,Camk2g,Ccdc109a,Cdh2,Clu,Coro1a,Ctnnb1,Dn
ENSMUSP00(	Ap2s1,Ap3b2,Atp8a1,Cd81,Cdh2,Clu,Ctnnb1,Cttn,Gpm6b,Hnrnpa1,Homer1,Hoo
ENSMUSP00(	App,Clu,Ctnnb1,Cttn,Grin1,Hook3,Lgi1,Lingo1,Mgll,Napa,Negr1,Olfm1,Pacsin1,P
ENSMUSP00(	Cacnb4,Camk2a,Camk2g,Ccdc109a,Coro1a,Grin1,Prkcb,Prkce,Ryr2
ENSMUSP00(	Aak1,Ap2s1,Ap3b2,App,Atp6v1a,Atp8a1,Cacnb4,Camk2a,Camk2g,Ccdc109a,Clu,
ENSMUSP00(	Brk1,Cdh2,Cttn,Grin1,Mgll,Mtor,Nckap1,Negr1,Pcp4,Rasal1,Syngap1,Syt1,Ywhal
ENSMUSP00(	App,Atp1a1,Atp2a2,Cacnb4,Calb1,Ccdc109a,Gnb1,Grin1,Hk1,Homer1,Prkcb,Prk
ENSMUSP00(	App,Cdh2,Ctnnb1,Cttn,Grin1,Hook3,Lingo1,Mgll,Negr1,Olfm1,Pcp4,Rasal1,Synga
ENSMUSP00(	Actr3,Arpc4,Coro1a,Cttn,Mtor,Prkce,Rhoa
ENSMUSP00(	App,Cdh2,Ctnnb1,Cttn,Grin1,Hook3,Lingo1,Mgll,Mtor,Negr1,Olfm1,Pcp4,Rasal1
ENSMUSP00(	App,Ctnnb1,Epha4,Hook3,Lingo1,Olfm1,Rhoa,Syngap1,Ywhah
ENSMUSP00(	Actr3,Ap1g1,Ap3b2,App,Atp6v1a,Atp8a1,Bpnt1,Brk1,Cab39,Cacnb4,Calb1,Camk
ENSMUSP00(	Ap1g1,Atp2a2,Camk2a,Ccdc109a,Napa,Pclo,Pcp4,Plcb1,Prkcb,Prkce,Rab8a,Stx1a
ENSMUSP00(	App,Eif3a,Eif3f,Hnrnpc,Mtor,Npm1,Pcbp1,Rhoa,Rps3,Tardbp,Vars
ENSMUSP00(	Pacsin1,Rab3a,Sh3gl2,Syt1
ENSMUSP00(	Actr3,Arpc4,Brk1,Cdh2,Clu,Cttn,Mtor,Nckap1,Prkce,Psmc1,Synpo
ENSMUSP00(	Ap1g1,Ap3b2,App,Atp6v1a,Atp8a1,Cacnb4,Camk2a,Camk2g,Ccdc109a,Clu,Coro
ENSMUSP00(	Ap1g1,App,Brk1,Cab39,Cacnb4,Calb1,Camk2a,Ccdc109a,Clu,Coro1a,Crk,Ctnnb1,
ENSMUSP00(	Coro1a,Ctnnb1,Grin1,Pcp4,Ppp5c,Prkcg,Rhoa,Syngap1
ENSMUSP00(	Grin1,Ppp1r9b,Ppp5c,Prkce,Prkcg,Ryr2,Srr
ENSMUSP00(	App,Cacnb4,Calb1,Dnm1,Epha4,Grin1,Negr1,Prkce,Slc4a10
ENSMUSP00(	App,Atp1a1,Atp2a2,Cacnb4,Calb1,Ccdc109a,Coro1a,Ctnnb1,Gnb1,Grin1,Hk1,Ho
ENSMUSP00(	Atp2a2,Camk2a,Napa,Rab3a,Stx1a
ENSMUSP00(	Brk1,Nckap1,Psd3,Rab14,Rab3a,Rab8a,Rhoa,Syngap1

ENSMUSP00(	Actr3,Arpc4,Brk1,Coro1a,Crk,Ctnnb1,Cttn,Gpm6b,Mtor,Nckap1,Npm1,Phyhip,Pl
ENSMUSP00(	Aak1,Ap1g1,Coro1a,Napa,Pacsin1,Pclo,Plcb1,Rab8a,Sh3gl2,Stx1a,Syt1
ENSMUSP00(	App,Gria2,Gria3,Grin1
ENSMUSP00(	Actr3,Arpc4,Clu,Coro1a,Cttn,Mtor,Napa,Prkce,Psmc1,Stx1a
ENSMUSP00(	Clu,Coro1a,Ctnnb1,Grin1,Npm1,Pcp4,Ppp5c,Prkcg,Syngap1
ENSMUSP00(	Pcbp2,Psma2,Psma3,Psma4,Psma5,Psmb1,Psmb6,Psmc1,Psmd6
ENSMUSP00(	Grin1,Rab8a,Syngap1,Syp
ENSMUSP00(	App,Atp6v0d1,Cacnb4,Calb1,Camk2g,Cdh2,Clu,Cplx2,Ctnnb1,Cttn,Gnb1,Gpd2,G
ENSMUSP00(	App,Atp2a2,Brk1,Cab39,Cacnb4,Camk2a,Ccdc109a,Clu,Coro1a,Crk,Ctnnb1,Epha
ENSMUSP00(	Ap1g1,Ap2a1,Ap2s1,Ap3b2,Cttn,Napa,Pacs1,Rab14,Rab3a,Rab8a,Stx1a,Vps35,Y
ENSMUSP00(	Atp2a2,Brk1,Cab39,Ccdc109a,Clu,Lingo1,Mtor,Ncald,Nckap1,Pclo,Plcb1,Ppp1r9b
ENSMUSP00(	Napa,Nckap1,Rab14
ENSMUSP00(	App,Arpc4,Brk1,Camk2g,Ccdc109a,Cdh2,Clu,Cttn,Dnm1,Grin1,Npm1,Olfm1,Ppp
ENSMUSP00(	App,Arpc4,Brk1,Camk2g,Ccdc109a,Cdh2,Clu,Cttn,Dnm1,Grin1,Npm1,Olfm1,Ppp
ENSMUSP00(	App,Arpc4,Brk1,Camk2g,Ccdc109a,Cdh2,Clu,Cttn,Dnm1,Eif3a,Eif3f,Grin1,Npm1,
ENSMUSP00(	Atp2a2,Ctnnb1,Grin1,Mtor,Npm1,Plcb1,Ppp1r9b,Ppp5c,Prkce,Prkcg,Rab8a,Rhoa
ENSMUSP00(	Atp2a2,Ctnnb1,Grin1,Mtor,Plcb1,Ppp1r9b,Ppp5c,Prkce,Prkcg,Rab8a,Rhoa,Ryr2,S
ENSMUSP00(	Aak1,Ccdc109a,Cd81,Cdh2,Clu,Crk,Ctnnb1,Epha4,Grin1,Lancl2,Lgi1,Mtor,Plcb1,P
ENSMUSP00(	App,Atp1a1,Atp2a2,Atp6v0d1,Atp8a1,Brk1,Cacnb4,Calb1,Camk2g,Cdh2,Clu,Cor
ENSMUSP00(	Actr3,App,Atp2a2,Brk1,Cacnb4,Calb1,Camk2g,Cdh2,Clu,Cplx2,Ctnnb1,Cttn,Gnb1
ENSMUSP00(	Ap2s1,Ap3b2,Atp8a1,Cacnb4,Clu,Cttn,Gpm6b,Hnrnpa1,Hook3,Napa,Npm1,Pacs
ENSMUSP00(	Aak1,Ccdc109a,Cd81,Cdh2,Clu,Crk,Ctnnb1,Epha4,Grin1,Lancl2,Lgi1,Mtor,Phyhip
ENSMUSP00(	Actr3,Ap3b2,Cdh2,Cplx2,Ctnnb1,Npm1,Pacs1,Pacsin1,Pclo,Rab3a,Rab8a,Sh3gl2,
ENSMUSP00(	Ap1g1,Napa,Pclo,Plcb1,Rab8a,Stx1a,Syt1
ENSMUSP00(	Cdh2,Cttn,Grin1,Mgll,Negr1,Pcp4,Rasal1,Syngap1,Syt1,Ywhah
ENSMUSP00(	App,Atp6v0d1,Clu,Ctnnb1,Epha4,Grin1,Hook3,Lingo1,Napa,Plcb1,Rhoa,Slc1a2,S
ENSMUSP00(	App,Atp6v0d1,Brk1,Cacnb4,Calb1,Camk2g,Cdh2,Clu,Cplx2,Ctnnb1,Cttn,Gnb1,Gp
ENSMUSP00(	App,Cacnb4,Dnm1,Epha4,Grin1,Homer1,Slc1a2
ENSMUSP00(	Rab3a,Syn1,Syp
ENSMUSP00(	Ppp1r9b,Syp
ENSMUSP00(	Cplx2,Pclo,Rab3a,Rab8a,Syt1
ENSMUSP00(	Atp2a2,Ccdc109a,Ncald,Ppp1r9b,Ryr2
ENSMUSP00(	Actr3,Arpc4,Coro1a,Cttn,Mgll,Mtor,Npm1,Prkce,Rhoa
ENSMUSP00(	App,Eif3a,Eif3f,Mtor,Npm1,Pcbp1,Rhoa,Rps3,Vars
ENSMUSP00(	Ap1g1,App,Brk1,Cab39,Cacnb4,Calb1,Camk2a,Ccdc109a,Coro1a,Cplx2,Crk,Ctnnb
ENSMUSP00(	Actr3,Arpc4,Brk1,Ctnnb1,Cttn,Mtor,Nckap1,Npm1,Phyhip,Plcb1,Prkce,Sfpq,Synp
ENSMUSP00(	Actr3,App,Atp6v0d1,Coro1a,Cttn,Epha4,Lgi1,Lingo1,Mtor,Nckap1,Pacsin1,Ppp1r
ENSMUSP00(	Actr3,Ap1g1,App,Atp2a2,Brk1,Cacnb4,Calb1,Camk2g,Cdh2,Clu,Cplx2,Ctnnb1,Ctt
ENSMUSP00(	Atp2a2,Ccdc109a,Ncald,Pclo,Ppp1r9b,Ryr2
ENSMUSP00(	Pcbp2,Psma2,Psma3,Psma4,Psma5,Psmb1,Psmb6,Psmc1,Psmd3,Psmd6
ENSMUSP00(	Aak1,Actr3,Ap1g1,Arpc4,Atp1a1,Atp8a1,Brk1,Cab39,Cacnb4,Camk2a,Ccdc109a,
ENSMUSP00(	Actr3,Brk1,Coro1a,Coro2b,Ctnnb1,Hook3,Nckap1,Npm1,Pacsin1,Pclo,Ppp1r9b,R
ENSMUSP00(	Cacnb4,Camk2a,Camk2g,Ccdc109a,Coro1a,Grin1,Prkcb,Prkce,Ryr2,Slc17a7,Slc4a
ENSMUSP00(	Ap2s1,Ap3b2,Cdh2,Clu,Cttn,Gpm6b,Hook3,Napa,Pacs1,Pclo,Rab14,Rab3a,Rab8a
ENSMUSP00(	Cdh2,Coro1a,Ctnnb1,Cttn,Grin1,Mgll,Olfm1,Rasal1,Syngap1,Syt1,Ywhah
ENSMUSP00(	App,Brk1,Camk2g,Cdh2,Dnm1,Grin1,Npm1,Olfm1,Ppp5c,Srr

ENSMUSP00	Actn1,Ap2a1,App,Atp1a1,Atp2a2,Cd200,Cdh2,Clu,Coro1a,Ctnnb1,Cttn,Epha4,Gr
ENSMUSP00	Brk1,Mtor,Nckap1
ENSMUSP00	App,Atp2a2,Cacnb4,Cdh2,Clu,Ctnnb1,Cttn,Epha4,Homer1,Hook3,Lgi1,Lingo1,M
ENSMUSP00	Actr3,Ap1g1,App,Atp2a2,Atp6v0d1,Cacnb4,Camk2g,Clu,Cplx2,Ctnnb1,Cttn,Grin
ENSMUSP00	Aak1,Actr3,Ap2s1,Ap3b2,Armc10,Atp6v1a,Atp8a1,Bpnt1,Brk1,Cab39,Cacnb4,Ca
ENSMUSP00	Cplx2,Pclo,Rab8a,Slc17a7,Syn1,Syt1
ENSMUSP00	Cdh2,Cttn,Epha4,Grin1,Mgll,Syngap1
ENSMUSP00	App,Atp1a1,Cacnb4,Gnb1,Grin1,Mtor,Pcp4,Prkcb,Prkce,Rhoa,Ryr2,Slc1a2,Slc4a
ENSMUSP00	Cplx2,Rab3a,Rab8a,Stx1a
ENSMUSP00	Pacsin1,Sh3gl2,Syt1
ENSMUSP00	Ap1g1,App,Atp2a2,Cacnb4,Camk2g,Clu,Cplx2,Ctnnb1,Cttn,Grin1,Hnrnpc,Homer
ENSMUSP00	Clu,Mtor,Npm1,Pcbp1,Rhoa
ENSMUSP00	Cplx2,Pclo,Rab8a,Syn1,Syt1
ENSMUSP00	Ap1g1,Ap3b2,App,Camk2a,Ccdc109a,Coro1a,Cplx2,Hook3,Npm1,Pacs1,Prkce,Ra
ENSMUSP00	Aak1,Actn1,Actr3,Ap1g1,Arpc4,Atp8a1,Brk1,Cab39,Cacnb4,Calb1,Camk2a,Ccdc1
ENSMUSP00	App,Cdh2,Cttn,Grin1,Mgll,Negr1,Pcp4,Rasal1,Syngap1,Syt1,Ywhah
ENSMUSP00	App,Atp6v0d1,Ctnnb1,Grin1,Hook3,Napa,Plcb1,Rhoa,Slc1a2,Slc4a10,Slc6a17,Sr
ENSMUSP00	Atp2a2,Atp8a1,Camk2a,Cdh2,Clu,Napa,Pacs1,Pacsin1,Rab8a,Stx1a,Syngap1,Tar
ENSMUSP00	Napa,Rab3a,Stx1a
ENSMUSP00	App,Cttn,Epha4,Lgi1,Lingo1,Pacsin1,Ppp1r9b,Rab8a,Rhoa,Syngap1,Tbc1d24
ENSMUSP00	App,Armc10,Cd81,Cttn,Lgi1,Mgll,Plcb1,Ppp1r9b,Prkcb,Rasal1,Rhoa,Syt1
ENSMUSP00	Coro1a,Grin1,Pcp4,Prkcg,Rhoa,Syngap1
ENSMUSP00	Ap1g1,Atp2a2,Ccdc109a,Coro1a,Hnrnpa1,Hook3,Npm1,Pacs1,Prkce,Rab14,Ryr2
ENSMUSP00	App,Cacnb4,Dnm1,Epha4,Grin1
ENSMUSP00	Cacnb4,Gnb5,Homer1,Plcb1,Ryr2,Ywhah
ENSMUSP00	Aak1,App,Atp1a1,Atp6v0d1,Atp6v1a,Atp6v1c1,Atp8a1,Bpnt1,Cab39,Cacnb4,Car
ENSMUSP00	Atp2a2,Atp8a1,Camk2a,Cdh2,Clu,Pacs1,Pacsin1,Rab8a,Stx1a,Syngap1,Tardbp
ENSMUSP00	Brk1,Epha4,Mtor,Nckap1,Negr1,Pcp4,Rasal1,Syt1
ENSMUSP00	Ap1g1,App,Ctnnb1,Grin1,Plcb1,Rhoa,Slc17a7
ENSMUSP00	Pcbp2,Psma2,Psma3,Psma4,Psma5,Psmb1,Psmb6,Psmc1,Psmd3,Psmd6
ENSMUSP00	Actr3,Coro1a,Cttn,Pacsin1,Ppp1r9b,Rhoa
ENSMUSP00	Cdh2,Ctnnb1,Cttn,Grin1,Mgll,Olfm1,Syngap1,Ywhah
ENSMUSP00	Actr3,Atp2a2,Atp6v0d1,Brk1,Camk2a,Coro1a,Coro2b,Ctnnb1,Dnm1,Hnrnpc,Ho
ENSMUSP00	Ap2s1,Ap3b2,Clu,Cttn,Gpm6b,Hook3,Napa,Pacs1,Pclo,Rab14,Rab3a,Rab8a,Stx1
ENSMUSP00	App,Atp6v0d1,Ctnnb1,Grin1,Hook3,Napa,Plcb1,Rhoa,Slc1a2,Slc4a10,Slc6a17,Sr
ENSMUSP00	Actr3,Arpc4,Cttn,Mtor,Prkce,Psmc1
ENSMUSP00	App,Atp8a1,Calb1,Grin1,Plcb1,Slc17a7,Syngap1
ENSMUSP00	Actn1,App,Atp1a1,Cd200,Cdh2,Clu,Coro1a,Ctnnb1,Cttn,Epha4,Gnai1,Gnb5,Gpm
ENSMUSP00	Cacnb4,Gnb5,Homer1,Plcb1,Ryr2,Ywhah
ENSMUSP00	Brk1,Coro1a,Nckap1
ENSMUSP00	App,Grin1,Slc1a2,Syngap1
ENSMUSP00	Atp2a2,Cdh2,Clu,Dnm1,Hook3,Pacs1,Pacsin1,Rab8a,Tardbp
ENSMUSP00	Mgll,Plcb1,Ppp1r9b,Rgs6,Syp
ENSMUSP00	Grin1,Lgi1,Prkce,Stx1a,Syt1
ENSMUSP00	Camk2a,Clu,Npm1,Prkcb,Rps3
ENSMUSP00	Faah,Gpd2,Hk1,Mgll,Pcbp2,Plcb1,Pld3,Psma2,Psma3,Psma4,Psma5,Psmb1,Psm

ENSMUSP00(	App,Cttn,Epha4,Lgi1,Lingo1,Napa,Pacsin1,Ppp1r9b,Psd3,Rab8a,Rhoa,Slc4a10,Sy
ENSMUSP00(	Atp1a1,Atp2a2,Coro1a,Ctnnb1,Grin1,Mtor,Plcb1,Ppp1r9b,Ppp5c,Prkce,Prkcg,Ra
ENSMUSP00(	Atp1a1,Atp2a2,Calb1,Clu,Coro1a,Ctnnb1,Grin1,Mtor,Npm1,Plcb1,Ppp1r9b,Ppp5
ENSMUSP00(	Cd81,Cttn,Lgi1,Mgll,Ppp1r9b,Rasal1,Rhoa,Syt1
ENSMUSP00(	App,Cttn,Epha4,Lgi1,Lingo1,Pacsin1,Ppp1r9b,Rab8a,Rhoa,Slc4a10,Syngap1,Tbc1
ENSMUSP00(	Cab39,Cacnb4,Camk2a,Cd81,Clu,Crk,Ctnnb1,Epha4,Gnb5,Grpel1,Homer1,Mtor,I
ENSMUSP00(	Atp1a1,Atp2a2,Ryr2
ENSMUSP00(	Calb1,Grin1,Slc17a7
ENSMUSP00(	Atp6v0d1,Atp6v1a,Atp6v1c1
ENSMUSP00(	Clu,Olfm1
ENSMUSP00(	App,Cacnb4,Cdh2,Ctnnb1,Pclo
ENSMUSP00(	Ap1g1,App,Atp1a1,Cacnb4,Clu,Coro1a,Epha4,Gnb1,Grin1,Lgi1,Mtor,Pcbp2,Prkcl
ENSMUSP00(	App,Cab39,Camk2a,Cd81,Clu,Ctnnb1,Epha4,Npm1,Pacs1,Plcb1,Prkcb,Prkce,Psd3
ENSMUSP00(	App,Cab39,Cd81,Cdh2,Clu,Ctnnb1,Epha4,Mtor,Npm1,Pcp4,Plcb1,Ppp1r9b,Ppp5
ENSMUSP00(	Cdh2,Ctnnb1,Mtor
ENSMUSP00(	Brk1,Nckap1

ı,Rab8a,Stx1a,Syn1,Syngap1,Syp,Syt1
ɜrin1,Lgi1,Mgll,Mtor,Napa,Nckap1,Negr1,Npm1,Olfm1,Pacsin1,Pcp4,Phyhip,Plcb1,Ppp1r9b,Prl
ɜv2b,Syn1,Syp,Syt1
ı,Cplx2,Ctnnb1,Cttn,Gnb1,Grin1,Hk1,Hnrnpc,Homer1,Mgll,Mtor,Ncald,Nckap1,Npm1,Pclo,Plcl
ıoa,Ryr2,Synpo
Japa,Pacs1,Pacsin1,Pclo,Rab14,Rab3a,Rab8a,Sh3gl2,Syp,Syt1,Tfrc,Vps35
ɜnb5,Gpm6b,Grin1,Homer1,Mtor,Napa,Pacsin1,Pclo,Pcp4,Plcb1,Prkcb,Prkce,Rhoa,Ryr2,Sh3gl2

Cttn,Hnrnpa1,Hook3,Napa,Npm1,Pacs1,Pacsin1,Pclo,Prkce,Rab14,Rab8a,Ryr2,Sh3gl2,Syn1,Syt
2a,Camk2g,Camkv,Ccdc109a,Cd81,Cdh2,Clu,Coro1a,Coro2b,Cplx2,Crk,Ctnnb1,Dnm1,Eif3a,Eif3

ınb1,Cttn,Hnrnpa1,Hook3,Napa,Npm1,Pacs1,Pacsin1,Pclo,Prkce,Rab14,Rab8a,Ryr2,Sh3gl2,Syı
7a7,Sv2b,Syn1,Syp,Syt1
lh2,Clu,Coro1a,Coro2b,Ctnnb1,Dnm1,Eif3a,Eif3f,Epha4,Gpm6b,Grin1,Hnrnpc,Hook3,Lgi1,Lingc
gll,Napa,Nckap1,Negr1,Olfm1,Pacsin1,Pclo,Pcp4,Plcb1,Ppp1r9b,Psd3,Rab8a,Rasal1,Slc1a2,Slc4
ı4,Gnai1,Gpm6b,Lancl2,Lgi1,Mgll,Mtor,Napa,Npm1,Plcb1,Prkcb,Prkce,Rab3a,Rab8a,Rasal1,Rg
ɔ4,Plcb1,Prkcb,Prkce,Rhoa,Ryr2,Ywhah
Hook3,Napa,Npm1,Pacs1,Prkce,Rab14,Rab3a,Rab8a,Ryr2,Stx1a,Vps35,Ywhah

a7,Slc4a10,Tfrc
l,Gnb5,Gpm6b,Grin1,Homer1,Mtor,Napa,Nckap1,Olfm1,Pacsin1,Pclo,Pcp4,Plcb1,Prkcb,Prkce,
oa,Synpo
;,Slc17a7,Slc4a10,Tfrc
l,Dnm1,Epha4,Gnai1,Gnb1,Gnb2,Gnb5,Gng2,Gria2,Gria3,Grin1,Homer1,Hpcal4,Lingo1,Mtor,N
l,Dnm1,Epha4,Gnai1,Gnb1,Gnb2,Gnb5,Gng2,Gria2,Gria3,Grin1,Homer1,Hpcal4,Lingo1,Mtor,N
ı4,Gnai1,Gpm6b,Lancl2,Lgi1,Mgll,Napa,Npm1,Phyhip,Plcb1,Prkcb,Prkce,Rab3a,Rab8a,Rasal1,F
;,Slc17a7,Slc4a10,Tfrc
dc109a,Cd81,Cdh2,Clu,Coro1a,Cplx2,Ctnnb1,Cttn,Epha4,Gpm6b,Gria2,Gria3,Hnrnpa1,Homer1
ɜlc17a7,Slc4a10,Tfrc
ı1,Olfm1,Pcp4,Phyhip,Plcb1,Prkce,Psmc1,Rasal1,Sfpq,Synpo,Syt1
vhah

a2,Slc4a10,Syngap1

,Prkce,Psmc1,Stx1a,Synpo
ɜ,Ryr2,Slc17a7,Slc4a10,Tfrc
1,Camk2a,Camk2g,Ccdc109a,Cd200,Cd81,Cdh2,Clu,Coro1a,Cplx2,Crk,Ctnnb1,Cttn,Eif3a,Eif3f,E
ım1,Eif3a,Eif3f,Gpm6b,Grin1,Nckap1,Npm1,Olfm1,Pclo,Ppp1r9b,Ppp5c,Rab14,Rab8a,Rhoa,Srr

1,Camk2a,Camk2g,Ccdc109a,Cd200,Cd81,Cdh2,Clu,Coro1a,Cplx2,Crk,Ctnnb1,Cttn,Eif3a,Eif3f,E

h2,Clu,Coro1a,Cplx2,Ctnnb1,Gria2,Gria3,Hook3,Icam5,Npm1,Pacs1,Pacsin1,Pclo,Prkcb,Prkce,F
;,Tfrc

a7,Slc1a2,Slc4a10,Syngap1
4,Rab3a,Rab8a,Ryr2,Stx1a,Syngap1,Tln2,Vps35,Ywhah

L,Pcp4,Ppp1r9b,Psd3,Rab8a,Rasal1,Slc4a10,Syngap1,Syt1,Tbc1d24,Ywhah

9a,Cdh2,Clu,Coro1a,Cplx2,Ctnnb1,Cttn,Gpm6b,Gria2,Gria3,Hnrnpa1,Hook3,Icam5,Napa,Npm1
pa,Npm1,Olfm1,Pacsin1,Ppp1r9b,Ppp5c,Rhoa,Srr,Tuba4a

3f,Grin1,Hnrnpc,Hook3,Napa,Npm1,Olfm1,Pacsin1,Ppp1r9b,Ppp5c,Prkcb,Rhoa,Sfpq,Srr,Tuba4
Pcp4,Plcb1,Prkcb,Prkce,Rab8a,Ryr2,Stx1a,Syt1,Tardbp,Vps35
Npm1,Pacs1,Pacsin1,Pclo,Rab3a,Rab8a,Ryr2,Stx1a,Syngap1,Tfrc,Tln2,Vps35,Ywhah

m1,Eif3a,Eif3f,Gpm6b,Grin1,Nckap1,Npm1,Olfm1,Pclo,Ppp1r9b,Ppp5c,Rab14,Rab8a,Rhoa,Srr
k3,Napa,Nckap1,Npm1,Pacs1,Pacsin1,Pclo,Rab3a,Rab8a,Ryr2,Stx1a,Syngap1,Tfrc,Tln2,Vps35,
cp4,Ppp1r9b,Psd3,Rab8a,Rasal1,Slc4a10,Syngap1,Syt1,Tbc1d24,Ywhah

,Coro1a,Cplx2,Ctnnb1,Cttn,Gpm6b,Gria2,Gria3,Hnrnpa1,Hook3,Icam5,Napa,Npm1,Pacs1,Pacs
h
ce,Ryr2,Sfxn3,Slc17a7,Slc4a10,Tfrc
ap1,Syt1,Ywhah

,Syngap1,Syt1,Ywhah

2a,Camk2g,Ccdc109a,Cd81,Cdh2,Clu,Coro1a,Coro2b,Cplx2,Crk,Ctnnb1,Dnm1,Faah,Gnai1,Gnb
a,Syt1,Tardbp

1a,Cplx2,Ctnnb1,Gria2,Gria3,Hook3,Icam5,Npm1,Pacs1,Pacsin1,Pclo,Prkcb,Prkce,Rab8a,Ryr2,S
,Epha4,Gnai1,Gnb1,Gnb2,Gnb5,Gng2,Gria2,Gria3,Grin1,Homer1,Hpcal4,Lingo1,Mtor,Ncald,Nc

mer1,Npm1,Plcb1,Prkcb,Prkce,Ryr2,Sfxn3,Slc17a7,Slc4a10,Tfrc

lcb1,Prkce,Rab3a,Rhoa,Sfpq,Synpo,Ywhah

rin1,Homer1,Hook3,Lgi1,Lingo1,Mgll,Mtor,Napa,Nckap1,Negr1,Olfm1,Pacsin1,Pclo,Pcp4,Plcb1
4,Gnai1,Gnb1,Gnb2,Gnb5,Gng2,Gria2,Gria3,Grin1,Homer1,Hpcal4,Lingo1,Mtor,Ncald,Nckap1,
whah
b,Prkce,Psd3,Rab14,Rab3a,Rab8a,Rasal1,Rgs6,Ryr2,Syngap1

5c,Rhoa,Srr,Tuba4a
5c,Rhoa,Srr,Tuba4a
Olfm1,Ppp5c,Rhoa,Srr,Tuba4a
a,Ryr2,Slc1a2,Srr
Slc1a2,Srr
Prkcb,Prkce,Rhoa,Rps3,Sfpq,Stx1a,Syt1,Tardbp
o1a,Cplx2,Ctnnb1,Cttn,Dnm1,Gnb1,Gpd2,Gpm6b,Grin1,Hnrnpc,Homer1,Hook3,Lgi1,Lingo1,M
l,Gpd2,Grin1,Homer1,Hook3,Hsbp1,Lgi1,Lingo1,Mgll,Mtor,Napa,Nckap1,Negr1,Olfm1,Pacsin1
1,Pclo,Rab14,Rab3a,Rab8a,Slc17a7,Slc4a10,Slc6a17,Stx1a,Tfrc,Vps35,Ywhah
,Plcb1,Prkcb,Prkce,Rhoa,Rps3,Sfpq,Stx1a,Syt1,Tardbp
,Syngap1,Syt1

lc4a10,Slc6a17,Srr,Ywhah
od2,Grin1,Homer1,Hook3,Hsbp1,Lgi1,Lingo1,Mgll,Mtor,Napa,Nckap1,Negr1,Olfm1,Pacsin1,Pcl

b1,Epha4,Gnai1,Gnb1,Gnb2,Gnb5,Gng2,Gria2,Gria3,Grin1,Hpcal4,Lgi1,Lingo1,Mtor,Ncald,Nck
oo
r9b,Rab8a,Rhoa,Syngap1,Tbc1d24
n,Gnb1,Gpd2,Grin1,Hnrnpc,Homer1,Hook3,Hsbp1,Lgi1,Lingo1,Mgll,Mtor,Napa,Nckap1,Negr1

Cd81,Cdh2,Clu,Coro1a,Crk,Ctnnb1,Cttn,Epha4,Gpm6b,Homer1,Lancl2,Lgi1,Nckap1,Negr1,Olfn
Rhoa,Synpo,Tln2,Ywhah
a10,Slc6a17
a,Ryr2,Stx1a,Tfrc,Vps35,Ywhah

nai1,Gnb5,Gpm6b,Grin1,Hook3,Lancl2,Lingo1,Mgll,Mtor,Npm1,Olfm1,Pacsin1,Pcbp2,Pcp4,Plc

tor,Olfm1,Pacsin1,Plcb1,Ppp1r9b,Rab8a,Rhoa,Slc4a10,Syngap1,Tbc1d24
1,Hnrnpc,Homer1,Hook3,Hsbp1,Lgi1,Lingo1,Mgll,Mtor,Napa,Negr1,Olfm1,Pacsin1,Pcp4,Plcb1,
mk2a,Camk2g,Camkv,Ccdc109a,Cd200,Cd81,Cdh2,Clu,Coro1a,Coro2b,Cplx2,Crk,Ctnnb1,Eif3a,

10,Stx1a,Syngap1,Tfrc

1,Hook3,Hsbp1,Lgi1,Lingo1,Mgll,Mtor,Napa,Negr1,Olfm1,Pacsin1,Pcp4,Plcb1,Ppp1r9b,Psd3,R

ab14,Rab8a,Ryr2,Vps35
109a,Cd200,Cd81,Cdh2,Clu,Coro1a,Cplx2,Crk,Ctnnb1,Cttn,Eif3a,Eif3f,Epha4,Gnb1,Gnb2,Gnb5,

r,Ywhah
dbp

2

mk2a,Camk2g,Camkv,Cd81,Clu,Ctnnb1,Dnm1,Eif3a,Eif3f,Epha4,Gnai1,Gnb1,Gnb2,Gng2,Gpd2,

ok3,Nckap1,Npm1,Pacsin1,Pclo,Ppp1r9b,Ppp5c,Prkcb,Rab14,Rab8a,Rhoa,Rps3,Sfpq,Slc17a7,St
a,Tfrc,Vps35,Ywhah
r,Ywhah

n6b,Grin1,Hook3,Lancl2,Lingo1,Mgll,Mtor,Npm1,Olfm1,Pacsin1,Pcp4,Plcb1,Ppp1r9b,Ppp5c,Prk

b6,Psmc1,Psmd3,Psmd6,Ywhah

ngap1,Tbc1d24
b14,Rab8a,Rhoa,Ryr2,Slc1a2,Srr
c,Prkcb,Prkce,Prkcg,Psmc1,Rab14,Rab8a,Rhoa,Ryr2,Slc1a2,Srr,Syp,Tfrc

d24
Npm1,Pacs1,Pcp4,Plcb1,Prkcb,Prkce,Psd3,Psmd3,Rasal1,Rgs6,Rps3,Ryr2,Syngap1,Tbc1d24,Yw

b,Prkce,Psma2,Rab14,Ryr2,Slc1a2,Srr,Tfrc
3,Rasal1,Rgs6,Rps3,Ryr2,Syngap1,Tbc1d24
c,Prkce,Rhoa,Syngap1,Tardbp

kce,Psmc1,Rab3a,Rasal1,Rhoa,Sfpq,Sh3gl2,Stx1a,Syngap1,Synpo,Syt1,Ywhah

o1,Prkcb,Prkce,Rab14,Rhoa,Ryr2,Sfxn3,Slc17a7,Slc4a10,Syngap1,Syp,Syt1,Tardbp,Tfrc,Tln2,Va

2,Slc1a2,Stx1a,Syt1,Tardbp,Vps35,Ywhah

1,Vps35,Ywhah
3f,Faah,Gnai1,Gnb2,Gnb5,Gng2,Gpd2,Gpm6b,Gria2,Gria3,Grin1,Grpel1,Hk1,Hnrnpa1,Hnrnpc,H

n1,Syngap1,Syt1,Tln2,Vps35,Ywhah

o1,Mgll,Mtor,Napa,Nckap1,Npm1,Olfm1,Pacs1,Pacsin1,Ppp1r9b,Ppp5c,Prkcb,Prkce,Rab14,Rab
4a10,Slc6a17,Srr,Syngap1,Syt1,Tbc1d24
s6,Rps3,Ryr2,Sfpq,Stx1a,Syn1,Syngap1,Syp,Syt1,Tardbp

Rab14,Ryr2,Sh3gl2,Slc1a2,Stx1a,Syt1,Tardbp,Vps35,Ywhah

Napa,Ncald,Nckap1,Pacsin1,Pclo,Plcb1,Ppp1r9b,Ppp5c,Psd3,Rab14,Rab8a,Rasal1,Ryr2,Slc17a7,
Napa,Ncald,Nckap1,Pacsin1,Pclo,Plcb1,Ppp1r9b,Ppp5c,Psd3,Rab14,Rab8a,Rasal1,Ryr2,Slc17a7,
Rgs6,Rps3,Ryr2,Sfpq,Stx1a,Syn1,Syngap1,Syp,Syt1,Tardbp

1,Hook3,Icam5,Napa,Nckap1,Npm1,Pacs1,Pacsin1,Pclo,Ppp1r9b,Prkcb,Prkce,Rab8a,Rhoa,Ryr2

pha4,Gnb1,Gnb2,Gnb5,Gng2,Gpm6b,Gria2,Gria3,Grpel1,Hnrnpc,Homer1,Hook3,Hpcal4,Lancl
,Tuba4a

:pha4,Gnb2,Gnb5,Gng2,Gpm6b,Gria2,Gria3,Grpel1,Hk1,Hnrnpc,Homer1,Hook3,Hpcal4,Lancl2,

Rab8a,Ryr2,Sfxn3,Sh3gl2,Slc4a10,Sv2b,Syn1,Syngap1,Syt1,Tln2,Vps35

L,Pacs1,Pacsin1,Pclo,Prkcb,Prkce,Rab8a,Ryr2,Sfxn3,Sh3gl2,Slc4a10,Sv2b,Syn1,Syt1,Vps35,Ywha

4a

;,Tuba4a
Ywhah

sin1,Pclo,Prkcb,Prkce,Rab8a,Ryr2,Sfxn3,Sh3gl2,Slc4a10,Sv2b,Syn1,Syt1,Vps35,Ywhah

o2,Gnb5,Gng2,Gpd2,Gpm6b,Gria2,Gria3,Hk1,Hnrnpc,Homer1,Hook3,Hpcal4,Hsbp1,Lgi1,Lingo1

Sfxn3,Sh3gl2,Slc4a10,Sv2b,Syn1,Syt1,Vps35
:kap1,Npm1,Pacsin1,Pclo,Pcp4,Phyhip,Plcb1,Ppp1r9b,Psd3,Psmc1,Rab14,Rab3a,Rab8a,Rasal1,

1,Ppp1r9b,Psd3,Rab8a,Rasal1,Rhoa,Ryr2,Slc1a2,Slc4a10,Slc6a17,Srr,Syngap1,Syt1,Tbc1d24,Tfr
,Pacsin1,Pclo,Plcb1,Ppp1r9b,Ppp5c,Psd3,Rab14,Rab3a,Rab8a,Rasal1,Ryr2,Syngap1,Ywhah

gll,Mtor,Napa,Ncald,Nckap1,Negr1,Olfm1,Pacsin1,Pclo,Pcp4,Plcb1,Ppp1r9b,Psd3,Rab8a,Rasal
.,Pclo,Pcp4,Plcb1,Ppp1r9b,Psd3,Rab14,Rab8a,Rasal1,Rhoa,Ryr2,Slc1a2,Slc4a10,Slc6a17,Srr,Syr

lo,Pcp4,Plcb1,Ppp1r9b,Psd3,Rab14,Rab8a,Rasal1,Rhoa,Ryr2,Slc1a2,Slc4a10,Slc6a17,Srr,Syngap

ap1,Npm1,Pacsin1,Pcbp2,Pclo,Pcp4,Phyhip,Plcb1,Ppp1r9b,Psd3,Psma2,Psmc1,Rab14,Rab8a,R

.,Npm1,Olfm1,Pacsin1,Pclo,Pcp4,Plcb1,Ppp1r9b,Psd3,Rab14,Rab8a,Rasal1,Rhoa,Ryr2,Slc17a7,

n1,Pcbp1,Pcp4,Phyhip,Plcb1,Prkcb,Prkce,Psd3,Psmc1,Rasal1,Rgs6,Rhoa,Rps3,Ryr2,Sfpq,Slc1a2

b1,Ppp1r9b,Ppp5c,Prkcb,Prkce,Prkcg,Rasal1,Rgs6,Rhoa,Rps3,Ryr2,Sfpq,Syngap1,Tardbp,Vps35

,Ppp1r9b,Psd3,Rab8a,Rasal1,Rhoa,Slc4a10,Syngap1,Syt1,Tbc1d24,Tfrc,Ywhah
,Eif3f,Epha4,Gnb2,Gnb5,Gng2,Gpd2,Gpm6b,Gria2,Gria3,Grin1,Grpel1,Hk1,Hnrnpa1,Hnrnpc,Ho

ab8a,Rasal1,Rhoa,Slc4a10,Syngap1,Syt1,Tbc1d24,Tfrc,Ywhah

Gng2,Gpm6b,Gria2,Gria3,Homer1,Hook3,Hpcal4,Lancl2,Lgi1,Lingo1,Mgll,Napa,Ncald,Nckap1,N

Grin1,Grpel1,Hk1,Hnrnpa1,Hnrnpc,Lancl2,Mgll,Mtor,Ndufa5,Ndufb3,Npm1,Nudt2,Pcbp1,Pcbp

tx1a,Synpo,Tardbp,Tln2,Ywhah

kcb,Prkce,Prkcg,Rasal1,Rgs6,Rhoa,Rps3,Ryr2,Sfpq,Syngap1,Tardbp,Ywhah

ʼhah

rs,Ywhah

Homer1,Hook3,Hpcal4,Hsbp1,Lgi1,Lingo1,Mgll,Mtor,Napa,Ncald,Nckap1,Ndufa5,Ndufb3,Negr

8a,Rps3,Sfpq,Slc17a7,Srr,Stx1a,Syngap1,Synpo,Tardbp,Tbc1d24,Tln2,Tuba4a,Ywhah

,Slc1a2,Sv2b,Syn1,Syngap1,Syp,Syt1,Ywhah
,Sv2b,Syn1,Syngap1,Syp,Syt1,Ywhah

,Sfxn3,Sh3gl2,Slc4a10,Sv2b,Syn1,Syngap1,Syt1,Tln2,Vps35,Ywhah

2,Lgi1,Lingo1,Mgll,Napa,Ncald,Nckap1,Negr1,Olfm1,Pacsin1,Pcbp1,Pcbp2,Pclo,Pcp4,Phyhip,Pl

,Lgi1,Lingo1,Mgll,Napa,Ncald,Nckap1,Negr1,Olfm1,Pacs1,Pacsin1,Pcbp1,Pcbp2,Pclo,Pcp4,Phyh

ah

l,Mgll,Mtor,Napa,Ncald,Nckap1,Ndufa5,Ndufb3,Negr1,Npm1,Nudt2,Olfm1,Pacs1,Pcp4,Pip4k2

,Rps3,Ryr2,Sfpq,Slc1a2,Syngap1,Syp,Tardbp,Tfrc,Ywhah

rc

1,Rhoa,Ryr2,Slc17a7,Slc1a2,Slc4a10,Slc6a17,Srr,Syngap1,Syt1,Tbc1d24,Tfrc
ngap1,Syt1,Tbc1d24,Tfrc

o1,Syt1,Tbc1d24,Tfrc

asal1,Rhoa,Rps3,Ryr2,Sfpq,Slc17a7,Slc1a2,Slc4a10,Srr,Stx1a,Syngap1,Syp,Tardbp,Ywhah

Slc1a2,Slc4a10,Slc6a17,Syngap1,Syt1,Tbc1d24,Tfrc

2,Stx1a,Syngap1,Synpo,Syt1,Tbc1d24,Tfrc,Ywhah

5,Ywhah

omer1,Hook3,Hpcal4,Icam5,Lgi1,Lingo1,Mgll,Mtor,Napa,Ncald,Nckap1,Ndufa5,Ndufb3,Negr1,

Negr1,Olfm1,Pacsin1,Pcbp1,Pclo,Pcp4,Phyhip,Plcb1,Prkce,Psd3,Psmc1,Rab14,Rasal1,Rhoa,Rps

p2,Pip4k2c,Plcb1,Pld3,Ppme1,Prkcb,Prkce,Psma2,Psma3,Psma4,Psma5,Psmb1,Psmb6,Psmc1,

1,Npm1,Nudt2,Olfm1,Pacs1,Pacsin1,Pcbp1,Pcbp2,Pcp4,Phyhip,Pip4k2c,Plcb1,Ppme1,Ppp1r9b

lcb1,Prkce,Psd3,Psmc1,Psmd3,Rab14,Rasal1,Rgs6,Rhoa,Rps3,Ryr2,Sfpq,Sh3gl2,Slc1a2,Stx1a,Sy

ip,Plcb1,Prkce,Psd3,Psmc1,Psmd3,Rasal1,Rgs6,Rhoa,Rps3,Ryr2,Sfpq,Sfxn3,Sh3gl2,Slc17a7,Slc

c,Plcb1,Ppme1,Ppp1r9b,Psd3,Psmc1,Rab14,Rab8a,Rhoa,Rps3,Ryr2,Sfpq,Sfxn3,Slc4a10,Slc6a1

,Nudt2,Olfm1,Pacsin1,Pcbp1,Pcbp2,Pcp4,Phyhip,Pip4k2c,Plcb1,Pld3,Ppme1,Prkce,Psd3,Psma3

s3,Ryr2,Sfpq,Sh3gl2,Stx1a,Syn1,Syngap1,Synpo,Syp,Syt1,Vars,Vps35,Ywhah

Psmd3,Psmd6,Rab14,Rab3a,Rab8a,Rabggta,Rhoa,Rps3,Sfpq,Slc4a10,Srr,Syn1,Tardbp,Tuba4a,V

[illegible],Psd3,Psma2,Psma3,Psma4,Psma5,Psmb1,Psmb6,Psmc1,Psmd3,Psmd6,Rab14,Rab8a,Rabggta

[illegible]n1,Syngap1,Synpo,Syp,Syt1,Tbc1d24,Tfrc,Vars,Vps35,Ywhah

1a2,Slc4a10,Syngap1,Synpo,Syp,Syt1,Tbc1d24,Tfrc,Tln2,Vars,Vps35,Ywhah

7,Srr,Syn1,Syngap1,Syp,Syt1,Tardbp,Tbc1d24,Tfrc,Tln2,Tuba4a,Vars,Ywhah

3,Psma4,Psma5,Psmb1,Psmb6,Psmc1,Psmd3,Psmd6,Rabggta,Rasal1,Rgs6,Rhoa,Rps3,Ryr2,Sfpc

/ars,Vps35,Ywhah

,Rhoa,Rps3,Ryr2,Sfpq,Sfxn3,Slc4a10,Slc6a17,Srr,Syn1,Syngap1,Synpo,Syp,Syt1,Tbc1d24,Tfrc,T

ı,Sfxn3,Sh3gl2,Slc4a10,Syngap1,Synpo,Syt1,Tbc1d24,Tfrc,Tln2,Tuba4a,Vars,Vps35,Ywhah

ln2,Tuba4a,Vars,Vps35,Ywhah
