# Supplementary material for: Absence of Wdr13 Gene Predisposes Mice to Mild Social Isolation – Chronic Stress, Leading to Depression-Like Phenotype Associated With Differential Expression of Synaptic Proteins
Source: Front Mol Neurosci. 2018 Apr 25;11:133. doi: 10.3389/fnmol.2018.00133 (PMC5930177; doi:10.3389/fnmol.2018.00133)
Supplement: TABLE S4 — Delineation of downregulated proteins from PFC (at-least two unique peptides) of Wdr13-/0 mice after social isolation into KEGG Pathways using String analysis. [file Table_4.PDF]

#pathway ID	pathway description	observed ger	false discove	matching pro
4721	Synaptic vesicle cycle	12	1.32E-12	ENSMUSP00(
4724	Glutamatergic synapse	14	2.79E-12	ENSMUSP00(
4713	Circadian entrainment	13	3.30E-12	ENSMUSP00(
4723	Retrograde endocannabinoid signaling	13	8.48E-12	ENSMUSP00(
3050	Proteasome	9	4.21E-10	ENSMUSP00(
4728	Dopaminergic synapse	11	2.50E-08	ENSMUSP00(
4961	Endocrine and other factor-regulated	8	9.86E-08	ENSMUSP00(
4911	Insulin secretion	9	1.37E-07	ENSMUSP00(
4972	Pancreatic secretion	9	6.29E-07	ENSMUSP00(
4725	Cholinergic synapse	9	1.20E-06	ENSMUSP00(
5031	Amphetamine addiction	7	3.94E-06	ENSMUSP00(
4726	Serotonergic synapse	9	4.24E-06	ENSMUSP00(
4727	GABAergic synapse	7	3.74E-05	ENSMUSP00(
4014	Ras signaling pathway	10	3.77E-05	ENSMUSP00(
5032	Morphine addiction	7	3.77E-05	ENSMUSP00(
4062	Chemokine signaling pathway	9	4.02E-05	ENSMUSP00(
4730	Long-term depression	6	4.02E-05	ENSMUSP00(
4530	Tight junction	8	4.15E-05	ENSMUSP00(
4720	Long-term potentiation	6	4.15E-05	ENSMUSP00(
4261	Adrenergic signaling in cardiomyocyte	8	5.61E-05	ENSMUSP00(
4971	Gastric acid secretion	6	8.46E-05	ENSMUSP00(
4921	Oxytocin signaling pathway	8	9.80E-05	ENSMUSP00(
4015	Rap1 signaling pathway	9	0.000106	ENSMUSP00(
5100	Bacterial invasion of epithelial cells	6	0.000106	ENSMUSP00(
5412	Arrhythmogenic right ventricular cardi	6	0.000106	ENSMUSP00(
4919	Thyroid hormone signaling pathway	7	0.000132	ENSMUSP00(
4020	Calcium signaling pathway	8	0.00022	ENSMUSP00(
4916	Melanogenesis	6	0.000391	ENSMUSP00(
4066	HIF-1 signaling pathway	6	0.000694	ENSMUSP00(
4022	cGMP-PKG signaling pathway	7	0.00102	ENSMUSP00(
4970	Salivary secretion	5	0.0011	ENSMUSP00(
5033	Nicotine addiction	4	0.00115	ENSMUSP00(
4670	Leukocyte transendothelial migration	6	0.0012	ENSMUSP00(
4012	ErbB signaling pathway	5	0.00183	ENSMUSP00(
4666	Fc gamma R-mediated phagocytosis	5	0.00188	ENSMUSP00(
4540	Gap junction	5	0.00193	ENSMUSP00(
4310	Wnt signaling pathway	6	0.00225	ENSMUSP00(
5034	Alcoholism	6	0.00225	ENSMUSP00(
5206	MicroRNAs in cancer	6	0.00225	ENSMUSP00(
4510	Focal adhesion	7	0.00277	ENSMUSP00(
4144	Endocytosis	7	0.00313	ENSMUSP00(
4810	Regulation of actin cytoskeleton	7	0.00332	ENSMUSP00(
5205	Proteoglycans in cancer	7	0.00378	ENSMUSP00(
5214	Glioma	4	0.00378	ENSMUSP00(
4145	Phagosome	6	0.00409	ENSMUSP00(

4150	mTOR signaling pathway	4	0.00411	ENSMUSP00(
4966	Collecting duct acid secretion	3	0.00413	ENSMUSP00(
5010	Alzheimer s disease	6	0.00413	ENSMUSP00(
5143	African trypanosomiasis	3	0.00553	ENSMUSP00(
5016	Huntington s disease	6	0.00568	ENSMUSP00(
4750	Inflammatory mediator regulation of T	5	0.00578	ENSMUSP00(
4918	Thyroid hormone synthesis	4	0.00578	ENSMUSP00(
4270	Vascular smooth muscle contraction	5	0.00602	ENSMUSP00(
4260	Cardiac muscle contraction	4	0.00759	ENSMUSP00(
190	Oxidative phosphorylation	5	0.00799	ENSMUSP00(
4070	Phosphatidylinositol signaling system	4	0.00847	ENSMUSP00(
4960	Aldosterone-regulated sodium reabso	3	0.011	ENSMUSP00(
4973	Carbohydrate digestion and absorptio	3	0.0125	ENSMUSP00(
5030	Cocaine addiction	3	0.018	ENSMUSP00(
4930	Type II diabetes mellitus	3	0.0187	ENSMUSP00(
5014	Amyotrophic lateral sclerosis (ALS)	3	0.0218	ENSMUSP00(
5146	Amoebiasis	4	0.0337	ENSMUSP00(
4152	AMPK signaling pathway	4	0.0414	ENSMUSP00(
5203	Viral carcinogenesis	5	0.0414	ENSMUSP00(
4611	Platelet activation	4	0.0437	ENSMUSP00(

matching proteins in your network (labels)
Ap2a1,Ap2s1,Atp6v0d1,Atp6v1a,Atp6v1c1,Cplx2,Dnm1,Napa,Rab3a,Slc17a7,Stx1a,Syt1
Gnai1,Gnb1,Gnb2,Gnb5,Gng2,Gria2,Gria3,Grin1,Homer1,Plcb1,Prkcb,Prkcg,Slc17a7,Slc1a2
Camk2g,Gnai1,Gnb1,Gnb2,Gnb5,Gng2,Gria2,Gria3,Grin1,Plcb1,Prkcb,Prkcg,Ryr2
Faah,Gnai1,Gnb1,Gnb2,Gnb5,Gng2,Gria2,Gria3,Mgll,Plcb1,Prkcb,Prkcg,Slc17a7
Psma2,Psma3,Psma4,Psma5,Psmb1,Psmb6,Psmc1,Psmd3,Psmd6
Camk2g,Gnai1,Gnb1,Gnb2,Gnb5,Gng2,Gria2,Gria3,Plcb1,Prkcb,Prkcg
Ap2a1,Ap2s1,Atp1a1,Calb1,Dnm1,Plcb1,Prkcb,Prkcg
Atp1a1,Camk2g,Pclo,Plcb1,Prkcb,Prkcg,Rab3a,Ryr2,Stx1a
Atp1a1,Atp2a2,Atp2b4,Plcb1,Prkcb,Prkcg,Rab8a,Rhoa,Ryr2
Camk2g,Gnai1,Gnb1,Gnb2,Gnb5,Gng2,Plcb1,Prkcb,Prkcg
Camk2g,Gria2,Gria3,Grin1,Prkcb,Prkcg,Stx1a
App,Gnai1,Gnb1,Gnb2,Gnb5,Gng2,Plcb1,Prkcb,Prkcg
Gnai1,Gnb1,Gnb2,Gnb5,Gng2,Prkcb,Prkcg
Gnb1,Gnb2,Gnb5,Gng2,Grin1,Prkcb,Prkcg,Rasal1,Rhoa,Syngap1
Gnai1,Gnb1,Gnb2,Gnb5,Gng2,Prkcb,Prkcg
Crk,Gnai1,Gnb1,Gnb2,Gnb5,Gng2,Plcb1,Prkcb,Rhoa
Gnai1,Gria2,Gria3,Plcb1,Prkcb,Prkcg
Actn1,Ctnnb1,Cttn,Gnai1,Prkcb,Prkce,Prkcg,Rhoa
Camk2g,Gria2,Grin1,Plcb1,Prkcb,Prkcg
Atp1a1,Atp2a2,Atp2b4,Cacnb4,Camk2g,Gnai1,Plcb1,Ryr2
Atp1a1,Camk2g,Gnai1,Plcb1,Prkcb,Prkcg
Cacnb4,Camk2g,Gnai1,Plcb1,Prkcb,Prkcg,Rhoa,Ryr2
Crk,Ctnnb1,Gnai1,Grin1,Plcb1,Prkcb,Prkcg,Rhoa,Tln2
Arpc4,Crk,Ctnnb1,Cttn,Dnm1,Rhoa
Actn1,Atp2a2,Cacnb4,Cdh2,Ctnnb1,Ryr2
Atp1a1,Atp2a2,Ctnnb1,Mtor,Plcb1,Prkcb,Prkcg
Atp2a2,Atp2b4,Camk2g,Grin1,Plcb1,Prkcb,Prkcg,Ryr2
Camk2g,Ctnnb1,Gnai1,Plcb1,Prkcb,Prkcg
Camk2g,Hk1,Mtor,Prkcb,Prkcg,Tfrc
Atp1a1,Atp2a2,Atp2b4,Gnai1,Plcb1,Prkce,Rhoa
Atp1a1,Atp2b4,Plcb1,Prkcb,Prkcg
Gria2,Gria3,Grin1,Slc17a7
Actn1,Ctnnb1,Gnai1,Prkcb,Prkcg,Rhoa
Camk2g,Crk,Mtor,Prkcb,Prkcg
Arpc4,Crk,Prkcb,Prkce,Prkcg
Gnai1,Plcb1,Prkcb,Prkcg,Tuba4a
Camk2g,Ctnnb1,Plcb1,Prkcb,Prkcg,Rhoa
Gnai1,Gnb1,Gnb2,Gnb5,Gng2,Grin1
Crk,Mtor,Prkcb,Prkce,Prkcg,Rhoa
Actn1,Crk,Ctnnb1,Prkcb,Prkcg,Rhoa,Tln2
Ap2a1,Ap2s1,Dnm1,Psd3,Rhoa,Sh3gl2,Tfrc
Actn1,Arpc4,Brk1,Crk,Nckap1,Pip4k2c,Rhoa
Camk2g,Ctnnb1,Cttn,Mtor,Prkcb,Prkcg,Rhoa
Camk2g,Mtor,Prkcb,Prkcg
Atp6v0d1,Atp6v1a,Atp6v1c1,Coro1a,Tfrc,Tuba4a

Cab39,Mtor,Prkcb,Prkcg
Atp6v0d1,Atp6v1a,Atp6v1c1
App,Atp2a2,Grin1,Ndufa5,Ndufb3,Plcb1
Plcb1,Prkcb,Prkcg
Ap2a1,Ap2s1,Grin1,Ndufa5,Ndufb3,Plcb1
Camk2g,Plcb1,Prkcb,Prkce,Prkcg
Atp1a1,Plcb1,Prkcb,Prkcg
Plcb1,Prkcb,Prkce,Prkcg,Rhoa
Atp1a1,Atp2a2,Cacnb4,Ryr2
Atp6v0d1,Atp6v1a,Atp6v1c1,Ndufa5,Ndufb3
Pip4k2c,Plcb1,Prkcb,Prkcg
Atp1a1,Prkcb,Prkcg
Atp1a1,Hk1,Prkcb
Gnai1,Gria2,Grin1
Hk1,Mtor,Prkce
Gria2,Grin1,Slc1a2
Actn1,Plcb1,Prkcb,Prkcg
Cab39,Mtor,Rab14,Rab8a
Actn1,Atp6v0d1,Psmc1,Rhoa,Ywhah
Gnai1,Plcb1,Rhoa,Tln2
