# Supplementary material for: Absence of Wdr13 Gene Predisposes Mice to Mild Social Isolation – Chronic Stress, Leading to Depression-Like Phenotype Associated With Differential Expression of Synaptic Proteins
Source: Front Mol Neurosci. 2018 Apr 25;11:133. doi: 10.3389/fnmol.2018.00133 (PMC5930177; doi:10.3389/fnmol.2018.00133)
Supplement: TABLE S5 — Proteomics (4 plex iTRAQ) of hippocampus from socially isolated Wdr13+/0 and Wdr13-/0 mice. [file Table_5.PDF]

Accession	Description	Coverage	# Proteins	# Unique Peptides	Wdr13-/0 SI / Wdr13+/0 SI
31982332	glutamine synthetase [Mus musculus]	2.14	1	1	0.334
31982300	hemoglobin, beta adult t chain [Mus musculus]	15.65	1	1	0.357
29789104	beta-soluble NSF attachment protein [Mus musculus]	4.70	1	1	**0.475**
6755967	voltage-dependent anion-selective channel protein 3 isoform 2 [Mus mus	3.53	2	1	0.584
30061327	histone H2A type 1-H [Mus musculus]	50.00	6	1	0.587
110626109	tyrosine-protein phosphatase non-receptor type substrate 1 isoform 1 pre	2.36	2	1	0.695
448261637	dynamin-1-like protein isoform d [Mus musculus]	1.80	4	1	0.708
55741460	protein DJ-1 [Mus musculus]	10.05	1	1	0.791
6753138	sodium/potassium-transporting ATPase subunit beta-1 [Mus musculus]	3.62	1	1	0.791
225735584	hexokinase-1 isoform HK1 [Mus musculus]	2.94	2	2	0.797
651166428	serine/threonine-protein phosphatase 2B catalytic subunit alpha isoform i	2.94	4	1	0.813
33859560	rab GDP dissociation inhibitor alpha [Mus musculus]	6.71	1	2	0.829
22267442	cytochrome b-c1 complex subunit 2, mitochondrial precursor [Mus muscu	4.42	1	1	0.835
40556608	heat shock protein HSP 90-beta [Mus musculus]	0.97	1	1	0.846
30089714	histone H4 [Mus musculus]	11.65	1	1	0.848
21450053	protein FAM49B [Mus musculus]	3.40	1	1	0.850
68341935	microtubule-associated protein 2 isoform 2 [Mus musculus]	3.00	2	1	0.862
28916677	calcium/calmodulin-dependent protein kinase type II subunit alpha isofor	12.13	16	6	0.867
45597447	superoxide dismutase [Cu-Zn] [Mus musculus]	8.44	1	1	0.879
19705578	V-type proton ATPase subunit B, brain isoform [Mus musculus]	3.33	2	1	0.893
163838648	ATP synthase subunit gamma, mitochondrial isoform b [Mus musculus]	4.38	2	1	0.911
33469051	tubulin polymerization-promoting protein [Mus musculus]	11.47	1	2	0.921
33563240	actin, alpha skeletal muscle [Mus musculus]	17.77	4	7	0.925
165972305	syntaxin-binding protein 1 isoform b [Mus musculus]	3.37	2	1	0.934
148747424	ADP/ATP translocase 1 [Mus musculus]	11.41	2	3	0.969
28316760	histone H2B type 1-B [Mus musculus]	16.67	12	2	0.972
6681095	cytochrome c, somatic [Mus musculus]	10.48	1	1	0.983
31981304	V-type proton ATPase subunit d 1 [Mus musculus]	3.42	1	1	0.986
295821212	sideroflexin-3 isoform 3 [Mus musculus]	6.76	4	1	0.994
6680988	cytochrome c oxidase subunit 6A1, mitochondrial [Mus musculus]	18.75	1	1	0.996
134031994	proteasome subunit alpha type-2 [Mus musculus]	8.97	1	1	1.003
18700024	isocitrate dehydrogenase [NAD] subunit beta, mitochondrial [Mus muscul	7.55	1	2	1.015
6754524	L-lactate dehydrogenase A chain isoform 1 [Mus musculus]	8.13	2	2	1.016
21746161	tubulin beta-2B chain [Mus musculus]	21.12	4	2	1.022
148539957	alpha-internexin [Mus musculus]	2.59	1	1	1.024
171543853	microtubule-associated protein 1B [Mus musculus]	0.41	3	1	1.024
29243942	eukaryotic translation initiation factor 5A-2 [Mus musculus]	7.84	2	1	1.033
31543976	14-3-3 protein gamma [Mus musculus]	3.64	1	1	1.038
33563256	guanine nucleotide-binding protein G(k) subunit alpha [Mus musculus]	10.73	12	3	1.044
31543349	vesicle-fusing ATPase [Mus musculus]	6.05	1	3	1.046

62234487	plasma membrane calcium-transporting ATPase 1 [Mus musculus]	0.57	1	1	1.047
31560731	V-type proton ATPase catalytic subunit A [Mus musculus]	2.43	1	1	1.051
172072590	neurochondrin [Mus musculus]	2.33	1	1	1.052
359385700	14-3-3 protein zeta/delta isoform 2 [Mus musculus]	2.68	5	1	1.052
114326546	phosphoglycerate mutase 1 [Mus musculus]	11.42	2	2	1.055
157951604	adenylyl cyclase-associated protein 1 [Mus musculus]	4.01	1	1	1.056
19526818	phosphate carrier protein, mitochondrial precursor [Mus musculus]	2.80	1	1	1.058
51491845	clathrin heavy chain 1 [Mus musculus]	5.25	1	7	1.059
20070412	ATP synthase subunit O, mitochondrial precursor [Mus musculus]	5.16	1	1	1.059
6679937	glyceraldehyde-3-phosphate dehydrogenase isoform 2 [Mus musculus]	18.32	4	4	1.064
6678469	tubulin alpha-1C chain [Mus musculus]	20.27	3	1	1.064
160298209	aspartate aminotransferase, cytoplasmic [Mus musculus]	3.63	1	1	1.065
13937391	guanine nucleotide-binding protein G(I)/G(S)/G(T) subunit beta-2 [Mus n	3.24	3	1	1.069
684179332	dynamin-1 isoform 2 [Mus musculus]	8.93	8	5	1.070
6679439	peptidyl-prolyl cis-trans isomerase A [Mus musculus]	3.66	5	1	1.072
594190942	sodium/potassium-transporting ATPase subunit alpha-3 [Mus musculus]	10.86	7	9	1.073
54607098	succinate dehydrogenase [ubiquinone] flavoprotein subunit, mitochondria	4.22	1	2	1.083
34740335	tubulin alpha-1B chain [Mus musculus]	20.18	1	1	1.085
226958349	triosephosphate isomerase [Mus musculus]	10.70	1	2	1.086
283483966	4-aminobutyrate aminotransferase, mitochondrial isoform 2 precursor [M	1.58	2	1	1.086
6680159	histone H3.3 [Mus musculus]	28.68	3	2	1.096
69885073	myelin basic protein isoform 6 [Mus musculus]	17.19	8	2	1.097
112363107	neurofilament medium polypeptide [Mus musculus]	0.71	1	1	1.097
161086984	AP-2 complex subunit sigma [Mus musculus]	8.45	1	1	1.099
6671539	fructose-bisphosphate aldolase A isoform 2 [Mus musculus]	5.49	2	1	1.100
254692859	NADH dehydrogenase [ubiquinone] 1 alpha subcomplex subunit 9, mitocl	5.04	1	1	1.105
31981690	heat shock cognate 71 kDa protein [Mus musculus]	1.55	1	1	1.107
40254595	dihydropyrimidinase-related protein 2 [Mus musculus]	6.64	6	4	1.109
6678359	transketolase [Mus musculus]	0.96	1	1	1.118
45598372	brain acid soluble protein 1 [Mus musculus]	6.19	1	1	1.118
15809030	beta-synuclein [Mus musculus]	10.53	1	1	1.123
160707903	synapsin-1 isoform b [Mus musculus]	3.58	2	2	1.127
13385942	citrate synthase, mitochondrial precursor [Mus musculus]	2.59	1	1	1.131
31980648	ATP synthase subunit beta, mitochondrial precursor [Mus musculus]	15.12	1	5	1.132
40254361	heat shock 70 kDa protein 4L [Mus musculus]	1.67	3	1	1.136
31982030	rho GDP-dissociation inhibitor 1 [Mus musculus]	12.25	1	1	1.139
31982186	malate dehydrogenase, mitochondrial precursor [Mus musculus]	23.37	1	5	1.149
254553344	ATP-dependent 6-phosphofructokinase, muscle type [Mus musculus]	3.85	1	2	1.150
22165384	tubulin beta-4B chain [Mus musculus]	19.10	4	1	1.151
158853992	alpha-enolase [Mus musculus]	8.53	4	2	1.159
126032329	elongation factor 1-alpha 1 [Mus musculus]	5.84	2	2	1.160

10946574	creatine kinase B-type [Mus musculus]	7.87	1	2	1.160
18079339	aconitate hydratase, mitochondrial precursor [Mus musculus]	1.15	1	1	1.183
926657659	high mobility group protein B1 [Mus musculus]	7.44	1	1	1.193
359807367	pyruvate kinase PKM isoform M1 [Mus musculus]	7.16	2	3	1.199
226423909	2',3'-cyclic-nucleotide 3'-phosphodiesterase isoform 1 [Mus musculus]	4.25	2	1	1.211
256773209	histone H2A.V [Mus musculus]	35.16	2	1	1.217
6753428	creatine kinase U-type, mitochondrial precursor [Mus musculus]	5.02	1	1	1.224
672424492	heterogeneous nuclear ribonucleoprotein K isoform 4 [Mus musculus]	3.87	4	1	1.236
27369581	calcium-binding mitochondrial carrier protein Aralar1 [Mus musculus]	2.51	1	1	1.240
254553458	glucose-6-phosphate isomerase [Mus musculus]	7.17	1	3	1.251
21704066	ras-related protein Rap-1A precursor [Mus musculus]	6.52	2	1	1.265
6680748	ATP synthase subunit alpha, mitochondrial precursor [Mus musculus]	1.81	1	1	1.268
10946578	thymosin beta-4 [Mus musculus]	13.64	2	1	1.273
742068536	peroxiredoxin-6 isoform 2 [Mus musculus]	13.50	2	2	1.277
226874906	14-3-3 protein epsilon [Mus musculus]	28.63	1	4	1.277
157951596	carbonic anhydrase 2 [Mus musculus]	6.15	1	1	1.290
183396771	60 kDa heat shock protein, mitochondrial [Mus musculus]	2.62	1	1	1.292
6681115	cytochrome P450 3A13 [Mus musculus]	3.58	1	1	1.295
24418919	glycogen phosphorylase, brain form [Mus musculus]	1.90	1	1	1.299
30089710	histone H2A type 2-C [Mus musculus]	49.61	4	1	1.309
13385260	acyl-coenzyme A thioesterase 13 [Mus musculus]	9.29	1	1	1.314
18087731	dynein light chain 2, cytoplasmic [Mus musculus]	12.36	2	1	1.314
6754036	aspartate aminotransferase, mitochondrial [Mus musculus]	7.44	1	2	1.323
255958286	succinyl-CoA ligase [ADP/GDP-forming] subunit alpha, mitochondrial prec	5.20	1	1	1.386
295054271	spectrin alpha chain, non-erythrocytic 1 isoform 3 [Mus musculus]	1.67	2	3	1.388
269914154	uncharacterized protein LOC239673 [Mus musculus]	2.23	1	1	1.392
269847199	RNA-binding protein 10 isoform 3 [Mus musculus]	1.99	3	1	1.398
80861454	plasma membrane calcium-transporting ATPase 2 [Mus musculus]	1.50	1	1	1.454
158508501	septin-5 [Mus musculus]	4.34	1	2	1.519
594542556	myelin proteolipid protein isoform 3 [Mus musculus]	9.88	3	2	1.572
6755114	peroxiredoxin-5, mitochondrial precursor [Mus musculus]	12.86	1	2	1.716
145301578	hemoglobin subunit alpha [Mus musculus]	11.27	1	1	1.970
11528518	synaptic vesicle glycoprotein 2A [Mus musculus]	3.37	1	1	2.030
498752597	hemoglobin subunit beta-1 [Mus musculus]	21.77	1	2	8.687
17647499	hemoglobin subunit beta-2 [Mus musculus]	15.65	1	1	11.557
